# Fully addressable designer superstructures assembled from one single modular DNA origami

**DOI:** 10.1038/s41467-025-56846-2

**Published:** 2025-02-12

**Authors:** Johann M. Weck, Amelie Heuer-Jungemann

**Affiliations:** Max Planck Institute of Biochemistry, Am Klopferspitz 18, 82152 Martinsried, Germany and Center for Nanoscience, Ludwig-Maximilians University, Munich, Germany

**Keywords:** DNA nanotechnology, DNA

## Abstract

DNA nanotechnology and especially the DNA origami method are primal tools to create precise nanoscale objects. For DNA origami, a long ssDNA scaffold strand is folded by a multitude of smaller staple strands into base-pair accurate shapes, allowing for precise modification and incorporation of guest molecules. However, DNA origami are limited in size, and thus is the area that can be controlled with nanoscale precision. Prior methods of creating larger assemblies were either costly or lacked structural control. Here, we incorporate two methods of modularity into one exemplary modular DNA origami (moDON). The modularity allows for the creation of over 50,000 diverse monomers and subsequently the assembly of a plethora of fully addressable designer superstructures while keeping the construction cost very low. The here-introduced methods for modularity in DNA origami design offer an efficient, cost-effective solution for constructing precisely organized, and fully addressable structures on a variety of scales.

## Abstract (web summary)

Methods for creating large DNA assemblies with low cost and enhanced structural control are of interest. Here, the authors report moDON (modular DNA origami nanostructure) that combines two approaches to modularity resulting in tens of thousands of different monomers and superstructures, constructed at biological time scales and minimal cost.

## Introduction

The concept of modularity is widely used in a plethora of scientific and engineering disciplines^1^. Modularity divides structures or systems into subunits, that can be independently addressed and changed, without affecting the rest of the structure or system. Subunits may even be reusable for different structures or systems. This allows for low-effort, cost-effective assembly of, e.g. buildings^2^, hardware (through standardization of subparts), machine code (also: object-oriented programming) or genetic code^3^. In nature, well-defined subunits of only a few nm in size^4^ assemble into higher-order structures^5,6^ with sizes ranging from a few hundred nanometers (nm) to several micrometers (µm). As synthetic biology progresses, researchers aim to mimic such structures, both on a molecular and on a cellular level. Therefore, the need for modular building blocks capable of controllably assembling and disassembling into and from higher-order structures on biologically relevant time scales has become more urgent.

Structural DNA nanotechnology has become a widely used construction approach in synthetic biology. Especially the DNA origami^7^ technique, where a long, circular scaffold strand, extracted from the M13mp18 bacteriophage, is folded into any desired 2D or 3D shape using short synthetic staple strands, presents an indispensable tool for nanoscale engineering. DNA origami nanostructures (DONs) ranging from just a few nm to over a hundred nm can be easily designed and synthesized, with freely available CAD software such as caDNAno^8^. To date a large variety of DONs have been published, ranging from the simplest 2D structures^7^ to functional DNA origami motors^9^. A very attractive feature of DONs is their complete addressability for guest molecule placement with base pair (bp) precision, enabling e.g. the formation of fluorescent nanorulers^10^, plasmonic nanosensors^11,12^, nm-sized force sensors^13,14^, or to study complex biological questions^15–17^.

Despite all of these advantages, a bottleneck in DON design is the overall final size limit, determined by the scaffold strand (the M13mp18 genome is made up of 7249 bases). Although different insertions have led to scaffold sizes of close to 9000 bases, the overall size increase in 3D DONs is marginal. Therefore, a variety of different strategies have been reported to increase DON sizes. These include the use of a lambda/M13 hybrid phage as a scaffold source^18^, the development of orthogonal scaffold strands^19^, the use of unscaffolded structures^20–22^, origami slats^23,24^, or hierarchical assembly into finite^25,26^ or periodic^26–28^ structures. Each of these methods has at least one major drawback: With the lambda/M13 hybrid phage, synthesis of scaffolds larger than 50 000 bases^18^, equivalent to ~six regular origami was achieved. A similar size was achieved with superstructures from orthogonal scaffolds, reaching sizes equivalent to five regular origami. However, both approaches have similar bottlenecks: Firstly, the effort for scaffold production is increased tremendously and secondly, the unique scaffold sequence requires the same amount of unique staples to be designed and synthesized individually, increasing effort and monetary cost proportionally to superstructure size. The latter problem also affects unscaffolded nanostructures employing single-stranded tile (SST) assembly, where every ssDNA tile needs to be designed and synthesized individually. Nonetheless, using 10,000 unique ssDNA tiles, superstructures in the Gigadalton (GDa) scale have been constructed^22^. The largest superstructures reported to date were constructed using so-called DNA origami slats^24^, through connection of several long 6 or 12 helix bundles (HBs) via well-positioned, complementary ssDNA handles. These structures reached several µm and GDa in size and their synthesis is comparably cost effective. However, assembly is so far limited to planar 2D structures with low degrees of complexity in assembly. Yet it is the most efficient strategy reported to date for these kinds of large, flat and fully addressable superstructures. Another approach is the construction of superstructures with ssDNA connectors^29–32^. With this approach, different 3D shapes of superstructures can be constructed, but each connection requires many unique ssDNA connectors, making it susceptible to unwanted interactions and incomplete connections. GDa sizes can also be reached through hierarchical assembly, where successively larger structures are assembled in multiple subsequent steps. This approach has led to structures measuring 0.5 µm (2D), depicting a μm-sized image of the Mona Lisa^33^, while 3D structures could reach up to the GDa scale^25^. The high structural control and potential size make it an excellent approach for the formation of superstructures. Nevertheless, design and construction are faced with different challenges.

For the hierarchical assembly of superstructures, connectivity always faces one of two problems: On the one hand, assemblies of self-complementary DONs lead to repetitive, homomultimeric finite^25^ or periodic^26,27^ superstructures. This approach is simple and cost-effective, as in principle only one type of monomer is required. However, it has the great disadvantage of an overall loss of structural control of the superstructure beyond the repetitive subunit, as only form and size of the subunit is controlled, yet superstructure formation occurs without control over the final structure. Consequently, it is impossible to controllably retain the site-specific addressability of the structure beyond the single monomer or subunit. On the other hand, assemblies made up of many different DONs, each complementary to one another, but not themselves, yield finite, heteromultimeric superstructures^9,19^. With this approach the structural control over the superstructure is high and the site-specific addressability is retained. However, the downsides are the requirement for a multitude of different DONs, leading to high design effort and increasing costs, proportional to superstructure size, and/or the need to be assembled in several steps, resulting in a tremendous decrease in overall yield.

Therefore, currently, concepts combining both low cost and high structural versatility are missing. Such concepts could alleviate the issues associated with traditional hierarchical assembly and allow for the controlled assembly of large superstructures, or a multitude of orthogonal smaller structures, with retained site-specific addressability across the entire superstructure.

In response to this challenge, we developed two methods for modularity and combined them in one structure, the moDON, a single modular DNA origami formed from one set of staples, but capable of folding into tens of thousands of different connection configurations. The large number of orthogonal connection sites allows us to increase the number of monomers in one-pot assemblies, circumventing the extraction and purification of intermediates as generally required in traditional hierarchical assembly. We were able to controllably assemble and disassemble manifolds of moDONs into large superstructures as well as smaller, more complex structures, in the xy- as well as in the z-plane.

We engineered modularity into two different connection methods. The first method of modularity, in xy-direction, was created for shape-matching connection sites^25,26^. We achieved the multitude of connection sites by engineering a re-routable scaffold. Until now DNA origami was designed under the paradigm of creating one single, fixed scaffold routing for each structure. With this method alone, we were able to construct hundreds of individual moDON monomers with different shape-matching connection sites.

The second method of modularity is a three-strand connection system in the z-direction. Here we engineered orthogonal binding sites with well-positioned, directional, and optimally sized handles, circumventing the problems occurring in other three-strand-systems, lacking rigidity, losing control over connection stoichiometry, and/or the impractical use of many different unique strands for each connection. The z-connections were engineered to be directional, non-branching, not self-passivating, removable, rigid, orthogonal, with a low number of duplexes per connection, and only made up of one single DNA sequence per connection site. Together with the xy-connections, this allowed for the construction of > 50 000 unique moDON monomers.

We demonstrate the construction of arbitrary finite superstructures with connections in three dimensions. Additionally, we created periodic structures with monomeric and multimeric repetitive subunits reaching more than 1 GDa in size. Superstructures could be assembled in one-step reactions from monomers, without the need for purification and isolation of intermediate products, which is required in traditional hierarchical assembly. This greatly simplifies construction and simultaneously increases the overall yield. Further, we show that xy- and z-connections are fully orthogonal allowing for parallel and selective assembly and disassembly of each individual connection using different orthogonal triggers (see Figure S1 for overview). Finally, we show that the site-specific addressability of each monomer in the superstructure is fully retained by placing gold nanoparticles (Au NPs) at specific positions in the superstructures, demonstrating a highly controllable, efficient, and cost-effective strategy for the formation of large and orthogonal superstructures.

## Results

### Design & Characterization of the moDON

**Fig. 1:**
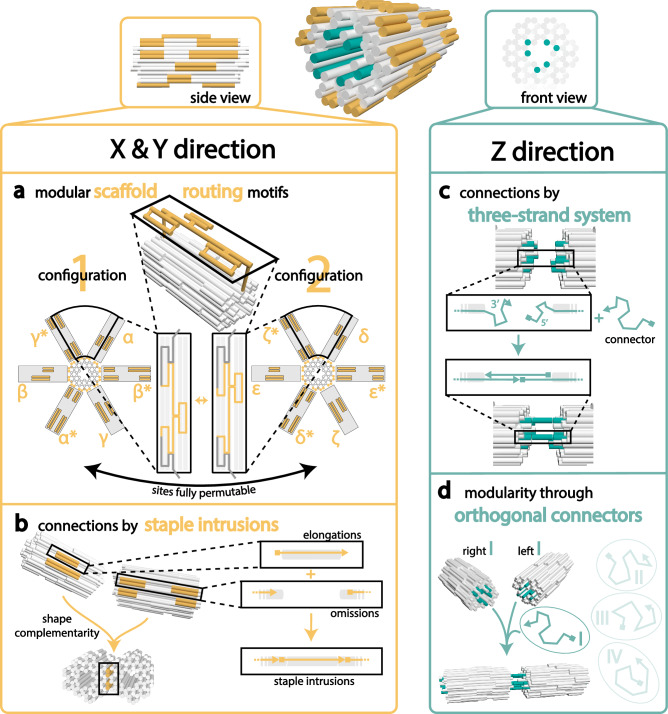
Connectivity and modularity of the moDON. Connectivity and modularity of the moDON is achieved by employing two orthogonal connection strategies, each with a unique modularity. **a** The xy-connections become modular through modular scaffold motifs, secluding the modular shell from the stable core structure: The scaffold performs a cross-over from the rigid core structure to the HH (yellow) on the helix ends. Whereas the scaffold cross-over from the HH to the connection sites (yellow) loop out from the middle of the helix. The ends remain stable (grey), and the middle part of the connection site is configurable with just a few staples. **b** In the xy-direction the connection is achieved by shape complementary sites and stabilized by short staple intrusions. The protruding four yellow helices in the left moDON are shape-complementary to the indentations of the right moDON, where elongated staples of one moDON hybridize to staple omissions on the scaffold of the second moDON. Complementarity of connection site shapes in combination with staple intrusions yield specificity. **c** The connection in z-direction (teal) is achieved via a three-strand-system, with ssDNA handles protruding from each monomer, each complementary to one half an external connector strand. Handles on the right side of the moDON are elongated at the 3’ end, and handles at the left side are elongated at the 5’ end, making the connection directional. **d** A set of four orthogonal connectors and handles on the moDON achieves modularity in the z-direction.

The basic design of the moDON is a 78 HB, based on the honeycomb lattice design (see Figure S2 for caDNAno layout). As can be seen in Fig. 1, the moDON monomer consists of one large core section (indicated in white), containing six extended helices on the left and right side of the structure (indicated in teal in Fig. 1), as well as a modular shell (indicated in yellow). The scaffold in the core part was layered evenly to facilitate folding (see Figure S3). To counteract any residual twist in the structure^25,34^, a deletion was introduced every 126 bp, thus adjusting the twist to 34.56°/bp over the whole structure (see Figure S4). The unused 116 nt long scaffold loop was positioned in the middle of the structure, pointing into the 6 HB cavities, to prevent any undesired interactions.

The different approaches to modularity and assembly connectivity of the moDON in both the xy- and the z-direction are illustrated in Fig. 1. Connection in the xy-direction was achieved by accurately fitting modularly changeable protrusions and indentations of helix duplexes (see yellow helices in Fig. 1a, b), while the connection in the z-direction was achieved by employing a three-strand system, including a connector-strand, continuing the ssDNA scaffold route in the design (see teal helices in Fig. 1c, d).

### Modularity and Assembly in xy-Direction

Modularity in the xy-direction was introduced by re-routing the scaffold on the outer shell of the moDON (yellow helices in Fig. 1a), without changing the core structure (white helices in Fig. 1). To minimize the number of nt in the core structure involved in re-routing of the shell, certain helices were chosen as so-called hinge helices (HH) between the stable core and the modular shell (see Figures S3, S5, S6). The modular shell section of the moDON consists of helices extending out from the HHs. To each side of one HH, two additional helices (four in total), extend out, forming patterns of protrusions or indentations that make up an xy-connection site as depicted in yellow in Fig. 1a (and Figure S3c). For the design of these connection sites, rules outlined in refs. ^26,35^ were adapted (*i.e*. a minimum length of 21 nt, as well as no staple/scaffold cross-overs close to end positions). Special attention was paid to avoid point symmetries in the design of the connection sites, which could lead to specific, but 180° turned connections. The twelve different active xy-connection sites that can possibly be formed were designated as α-ζ and α*-ζ* for the respective complementary sites. For ease of illustration, two different sets of configurations are depicted in Fig. 1a, with configuration 1 displaying connection sites α, β and γ as well as their complements α*- γ*, while configuration 2 displays connection sites δ, ε and ζ as well as their complements. It is important to note that a moDON can have a combination of connection sites from both configurations. However, α of the first configuration and δ of the second configuration share the same position in the moDON, thus not allowing them to be present on the same structure. The same holds true for β and ε, as well as for γ and ζ and all respective complements (an overview is given in Figure S7). Mutual exclusivity was ensured by checking all possible permutations of each connection site in all configurations. In order to obtain the different configurations, the scaffold routing in the HH and the adjacent helix loops needed to be adjusted from traditional DNA origami scaffold routing designs (see Fig. 1a, and S5).

As the scaffold is circular by default, and in order for only the shell, but not the core, to be modular, a complex scaffold routing approach was devised. In traditional DNA origami design, the scaffold cross-overs, connecting two adjacent helices, are generally placed at alternating positions between the ends of the helices and towards the middle of the helices (*cf*. Figure S3a). Constraints from the design of the modular shell and the requirement for simplicity in scaffold layering required a deviation from this traditional routing. To allow for modularity, the shell was designed from closed scaffold loops, adjacent to and protruding from each HH, forming two short, parallel helices (illustrated in Figures S3b, S3c, and S5). The scaffold cross-overs between these two helices in the loop were placed at the ends of the helices, while the cross-over to the HH was placed towards the middle (Figure S3b). Connection of the HH to the core structure therefore again was required to be at the helical ends, interrupting the alternating middle-end-middle scaffold cross-overs from helix 0 (H0) to H22 (for helix numeration see Figure S6a). By design, the cross-over between H22 and H23 was required to be placed in the middle of the structure, as H22 connects to HH75 at the helix ends. This was adjusted for all hinge motifs, allowing free modularity for all connections in the xy-direction, through addition of the appropriate staple subset (see Fig. 1a and S7). In order to correct for rounding errors when determining helical cross-over positions in these modular parts, insertions or deletions were added into the design as required (Figure S2, S4, and S6b).

Connection sites were designed to be orthogonal across both configurations. Since connection sites are complementary to the respective opposite connection site on the moDON, the position of the changeable section is positioned not point-symmetrically, but mirror-symmetrically (see Figure S7). Consequently, this leads to an uneven distribution of the number of configurations across the hinge helices, as HH65 only contains one configuration, whereas HH50 contains four configurations, of all permutations between the adjacent connection sites γ* or ζ* and α or δ. A total of 13 connection site mixes is therefore required to form all scaffold configurations (see Table S1). OxDNA simulations of moDONs in configurations 1 and 2 were used to predict correct folding and stability (Figure S8 and S9), which was further confirmed experimentally by agarose gel electrophoresis (AGE) and transmission electron microscopy (TEM) (Figures S10–S11). The AGE analysis revealed a quantitative yield of moDON monomers.

**Fig. 2:**
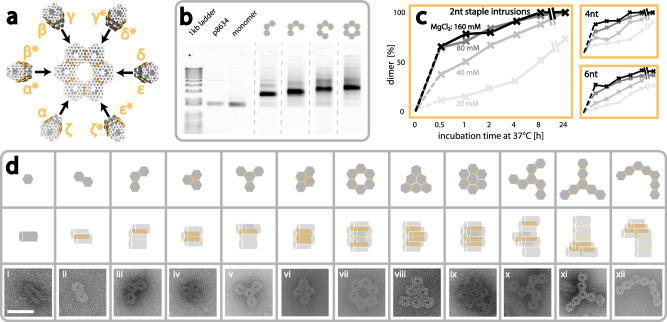
Superstructures in xy-direction. Superstructures in the xy-direction are constructed from a multitude of moDON monomers. Active connection sites are indicated in yellow. **a** Exemplary construction of a hexameric ring superstructure from 6 different moDON configurations (βγ, γ*δ*, δε, ε*ζ*, ζα, α*β*) as one-step-reaction. moDONs γ*δ* and ζα are cross configuration structures. **b** Gel image of hexameric ring construction and intermediate multimers. The monomer runs faster than the scaffold, p8634. Higher-order multimeric structures run slower the more monomers are assembled. **c** Fraction of moDON dimerized in xy-direction over incubation time. Longer incubation times and high MgCl_2_ concentration yielded a larger fraction of dimerized moDONs. For 40 mM and higher MgCl_2_ concentrations, ~100 % dimerization was achieved after 24 h. Increase from 80 to 160 mM MgCl_2_ did not yield better dimerization. Staple intrusion length did not influence dimerization rates. **d** Exemplary xy-direction superstructures made from various moDONs in one-step-reactions. Pictograms indicate designed structure. Scale bar is 50 nm and holds for all TEM micrographs.

Next, to ensure even more control over xy-connected assemblies, in addition to shape complementarity in the connection sites, we also introduced staple intrusions. Staples from one connection site were elongated by a few nucleotides to intrude into the complementary connection site, and hybridize to the scaffold (see Fig. 1b and S4). Furthermore, connection sites could also be de-activated/passivated by inclusion of 5 Thymine (T) staple overhangs (Figure S4). Together, this would allow us to mix a manifold of different moDON monomers, with specific connections sites, which could assemble into distinct superstructures (see Fig. 2a). To experimentally test our modularity and connectivity in the xy-direction, we initially formed dimers and trimers from all possible complementary monomers (α-ζ and α*-ζ*). Analysis by AGE revealed a clear shift in electrophoretic mobility for dimers and trimers, compared to monomers, proportional to superstructure size, suggesting successful assembly (Figures S12, S13). This was further confirmed by TEM imaging, which showed well-assembled dimers and trimers as illustrated in Fig. 2d(ii) and (iii) (and Figure S14b, c). The assembly yield, as determined from the gel band intensities in Figures S12 & S13 was found to be near quantitative (dimers: 94.8 %, trimers (iii): 91.7 %), demonstrating the effectiveness of our assembly strategy. Corresponding analysis of TEM images showed a still high, but somewhat reduced yield compared to values from gel band intensities (see Figures S15, S16). We attribute this decrease in yield to the additional mechanical stress exerted during blotting. This hypothesis is further supported when comparing yields of the different trimers. The assembly yield of trimer (iv), (cf. Figure 2d and S17a) as determined from gel band intensities was found to be 87.6 % (Figure S18a), comparable to that found for trimer (iii), but analysis of the corresponding TEM micrographs showed a much higher yield for trimer (iv), (see Figure S19). The difference between both structures is the additional connection site of trimer (iv) and the slightly decreased radius, which may lead to decreased shearing forces on connection sites.

After the successful assembly of dimers and trimers, we next sought to form larger structures and therefore constructed tetramers, hexamers, and heptamers (concept see Fig. 2a) in one-pot reactions. Both tetramers (v) and (vi) in Fig. 2d (and S17b, c) as well as hexamers (Fig. 2d and S23a, b) formed with high yields as determined by AGE (tetramer (v): 76.8 % (Figure S20); tetramer (vi): 66.1 % (Figures S18b); hexamer (vii): 91.7 % (Fig. 2b and S24); hexamer (viii): 80.8 % (Figure S18c)). TEM yields for all structures were again lower (see Figures S21, 22 and S25, 26). We further constructed the heptameric assemblies (ix)-(xii) in Fig. 2d (and S23c and S27). Due to a high abundance of incomplete assemblies, which migrated too similarly through the gel (*cf*. Figure S18d–f), we were unable to confidently extract a yield from AGE. TEM analysis, however, revealed low yields for each heptamer (see Figures S28–S31). We attribute the low yields to the combination of heptamers having a large radius, prone to shearing forces, and each monomer in the heptamer only being stabilized by one connection site in the main structure. In general, we reason, that the yield is proportional to the connection strength and negatively proportional to structure size, where larger structures are more prone to shearing forces (see also Figure S32). We further constructed periodic, homo-multimeric xy-lattices with a unit cell of 2 moDONs, resulting in 2-dimensional structures spanning more than 36 000 nm^2^ (Figure S33) made up of 46 monomers. We summarize, that one-step connections of a total of seven unique moDON monomers (from six unique connections) at the same time are possible. Modularity allows for the doubling of connections in each direction and thus increases the configuration space for superstructures 8-fold.

After successful assembly of a great variety of different higher order structures, we next sought to investigate the influence of different effects and design choices on superstructure assembly in the xy-direction in more detail. In brief, we examined a parameter tensor of (i) incubation times from 0.5 to 24 h, (ii) MgCl_2_ concentrations from 20 to 160 mM, and (iii) the length of intrusion of the staple of 2, 4, and 6 nt respectively. Analysis by AGE revealed that both the incubation time as well as the MgCl_2_ concentration in the buffer had a large influence on the dimerization ratio for all lengths of staple intrusions (see Figures S34, 35). Analyses of the corresponding band intensities (Fig. 2c) revealed that longer incubation times as well as higher concentrations of MgCl_2_ resulted in an increase in the yield of dimerized moDONs. At 20 mM MgCl_2_ the dimerization yield was only ~10 % after 0.5 h and ultimately only rose to 70 % after 24 h. It was thus deemed to not be sufficient to connect moDONs within a reasonable amount of time. Dimerization yields were improved by increasing the MgCl_2_ concentration to 40 mM, reaching ~100% dimerization after 24 h of incubation. It should be noted that a MgCl_2_ concentration of 160 mM did not further increase the speed of dimerization, but led to increased smearing of the bands in the gel (Figures S34, S35). We also note, that even this high concentration of MgCl_2_ did not lead to undesired multimerization, which we attribute to the compact design and thorough passivation. As can be seen from Fig. 2c, the abovementioned observations hold true for all lengths of staple intrusions, suggesting that their length does not play a vital role in the assembly process. We finally also investigated the influence of temperature (4 °C, 20 °C, and 37 °C) on assembly speed, but did not find it to have any significant influence (see Figure S36). Summarizing, we can identify a high MgCl_2_ concentration as the main trigger for assembly in the xy-direction.

### Modularity and Assembly in z-Direction

After successful assembly of moDON superstructures in the xy-direction, we next tested the connection strategy for the z-direction. Orthogonal to the xy-connectivity, using staple intrusions, connections in the z-direction were designed with a three-strand system. For this, left and right parts of the moDON were functionalized independently, resulting in the ability to connect to two different monomers at a time as illustrated in Fig. 1c, d. In order to allow for directional assembly, six helices were designated as connection sites, depicted in teal in Fig. 1. Staples of connector helices on the left side of the structure were elongated by an additional 10 nt at their 5’ end and staples of connector helices on the right side of the structure were elongated by 11 nt at their 3’ end. Upon the addition of a 21 nt long, complementary connector strand, a connection would be formed resulting in two full helical turns between monomers, continuing the helical pacing in the respective moDON monomers. To ensure stability and rigidity, each connection site was designed to consist of six handles of the same sequence. We specifically chose short lengths of extending staples of only 10/11 nt in order to prevent unintentional passivation of the connection site, enabling incompletely paired connectors to detach from the handle. To further minimize undesired hybridization, a three-letter alphabet of Cytosin, Adenin, and Thymine was used in the connector design. In total, we designed four different connectors: I, II, III, and IV (see Fig. 1d and Table S15). Their mutual orthogonality, as well as the orthogonality towards all moDON connection sites were verified in silico by NUPACK analysis (Figure S37).

**Fig. 3:**
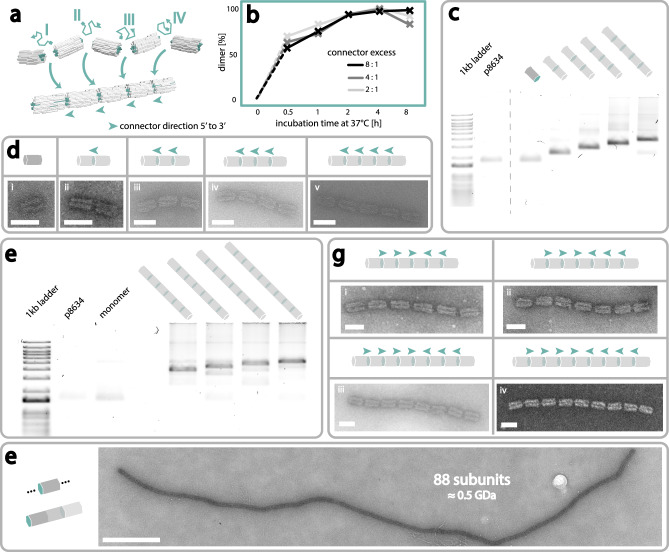
Superstructures in z-direction. Superstructures in the z-direction are constructed by connecting moDON monomers with a connector strand, partially complementary to a ssDNA handle on the moDON. **a** Construction of a pentamer from moDON monomers. The respective connector strand (I, II, III, or IV) hybridizes to specific ssDNA handles on a moDON. ssDNA handles on the left side of the moDON end with the 5’ whereas on the right side they end with the 3’ end, the direction of the connector is thus opposite, and marked with a small teal arrow. **b** Fraction of moDON dimerized in z-direction over incubation time. In each case more than 50 % moDONs were dimerized after 0.5 h. Increased connector access did not yield faster dimerization. **c** Gel image of z-directional connections forming superstructures of various sizes. Gel migration speed is reduced proportional to superstructure size. **d** Exemplary z-direction superstructures made from various moDONs in a one-step-reaction. Pictogram indicates designed structure; teal indicates connection sites. **e** Gel image of hexamers, heptamer, octamers, and nonamers from symmetric z-assemblies, harnessing the directionality of the connectors. Electrophoretic mobility reduces proportional to superstructure size. **f** Exemplary symmetrically assembled z-direction superstructures made from various moDONs in a one-step-reaction. **g** Assembly of a periodic tube of moDON in z-direction with a monomeric subunit. Scale bars in **d** and **f** are 50 nm, scale bar in **g** is 500 nm.

Similar to the assembly in the xy-direction, we initially experimentally investigated the formation of dimers with all possible permutations. AGE revealed successful dimer formation only upon addition of the correct connector strand, confirming mutual orthogonality of the connectors (Figure S38). Subsequent analysis of structures revealed a near quantitative dimer yield (AGE: 93.2 %, see Fig. 3c), TEM: 97.7 %, see Figure S39). To further showcase the orthogonality and efficacy of the three-strand-connecting system, we next constructed a multitude of different structures ranging from dimers to pentamers with one specific connector strand for each connection (see Fig. 3c, d and S40, S41). As can be seen from the AGE analysis in Fig. 3c, all structures formed successfully and with high yield, from one set of orthogonal moDONs. Higher-order structures displayed proportionally slower electrophoretic mobilities, as desired. Yield for trimers was 93.2 % (AGE) and 82.4 % (TEM), for tetramers 87.5 % (AGE) and 78.3 % (TEM), and for pentamers 87.3 % (AGE) and 65.9 % (TEM) as seen in Fig. 3c (and S42–S44).

We further engineered the moDON to carry every permutation of 3’ and 5’ handles, both left and right. This allowed us to construct larger finite superstructures with the same number of connections through symmetric assemblies. Here, a central monomer or dimer was constructed, with the same connector ends extending from both sides. To these ends two dimers, trimers or tetramers connect, resulting in even larger, but still finite superstructures (see Figure S45a). As can be seen in Fig. 3e, f (and S45b–S47) we successfully formed hexamers with a yield of 70.4 % (AGE) and 55.0 % (TEM, Figure S48), heptamers with a yield of 61.0 % (AGE) and 48.7 % (TEM, Figure S49), octamers with a yield of 56.8 % (AGE) and 31.2 % (TEM, Figure S50), and nonamers with a yield of 60.9 % (AGE) and 34.2 % (TEM, Figure S51). Our results revealed a slight negative proportionality of structure size and yield. A similar trend was seen for xy-connections, but for z-connections the overall yield, and especially in TEM analysis is comparably higher. We attribute this to a larger number of nucleotides (6 connectors at 21 nt = 126 nt) for each z-connection, compared to the xy-connections (4 x 2 nt = 8 nt), and thus more robust connections, outweighing the increased susceptibility to shearing forces through the larger radius.

After having successfully assembled finite structures, we consequently used our approach to construct periodic tubes from monomeric subunits. As can be seen in Fig. 3g (and S52), these tubes reached lengths of up to 88 monomers measuring almost 5 µm in length, corresponding to a molecular weight of ~0.5 GDa. With this we were further able to showcase the efficiency and orthogonality of our assembly approach in the z-direction.

Interestingly, when testing various connector handle to connector ratios, we found that connection was achieved even with a 1:1 ratio as determined by AGE (see Figure S53a). A more detailed analysis of these structures by TEM, however, revealed that the connection between single moDONs appeared to be bent (Figure S53b). Contrastingly, when the ratio was increased to 1:4 or higher, dimers were observed to be linear (Figure S39). We attribute this to the decreased probability of successfully forming all of the six possible connections if connector concentrations are too low, which in return leads to an inclination of the z-connections. Additionally, we did not observe any branching, which often is an unwanted side-reaction in similar connection strategies.

We next sought to investigate the interrelation of connection speed with connector and MgCl_2_ concentration. In the two-strand staple intrusion-based xy-assembly, reaction time and MgCl_2_ concentration were found to have a significant impact on dimer/superstructure formation, while intrusion length did not. For z-connections we thus investigated the influence of (i) incubation times, from 0.5 to 8 h, and (ii) connector to handle ratios from 2 to 8, (iii) temperature (from 4 to 37 °C), and (iv) MgCl_2_ concentrations from 5 to 40 mM. Analysis by AGE revealed the dimerization ratio to be very fast, reaching complete dimerization already after 2 h (Fig. 3b, S54a) at 37 °C independent of the connector excess. Interestingly, compared to the assembly kinetics in the xy-direction, connection in the z-direction was nearly twice as fast. We attribute this to the increased accessibility of the connection sites, at the ends of the moDON, compared to the slightly obstructed staple intrusions. Further, we suspect the repetitiveness of the connection sites to play a major role, as each connector has six potential, equal connection handles on the moDON. Structures incubated at lower temperature, however, showed much slower assembly kinetics with structures incubated at 4 °C only reaching a dimer yield of ~ 60 % after 8 h of incubation (Figure S55a, c). We attribute this to the diffusion speed of the connectors increasing with temperature, increasing interaction probability of connectors with handles. We additionally found that higher MgCl_2_ concentrations (40 mM) resulted in even faster dimerization kinetics, reaching full dimerization after only 1 h at 37 °C. However, after 8 h, all MgCl_2_ concentrations of 10 mM and higher resulted in full dimer formation (see Figure S54b, c). We thus conclude, that, as could be expected, the assembly process is dominantly triggered by the presence of the connector strand, while temperature and MgCl_2_ concentrations only played a role in assembly speed. Importantly, these findings confirm that the assembly trigger for z-directional connections (connector strands) is fully orthogonal to that of xy-directional assembly (high MgCl_2_ concentrations).

### Selective Assembly and Disassembly

We thus far showed that higher-order structures can be reliably formed in both xy- and z-directions, with high yields. However, control of size and structure of superstructures in xy- and z-direction constitute only one feature of the moDON. In the following we show assembly of structures combining xy- and z-connections, and the selective disassembly thereof, as well as investigating the underlying dynamics.

**Fig. 4:**
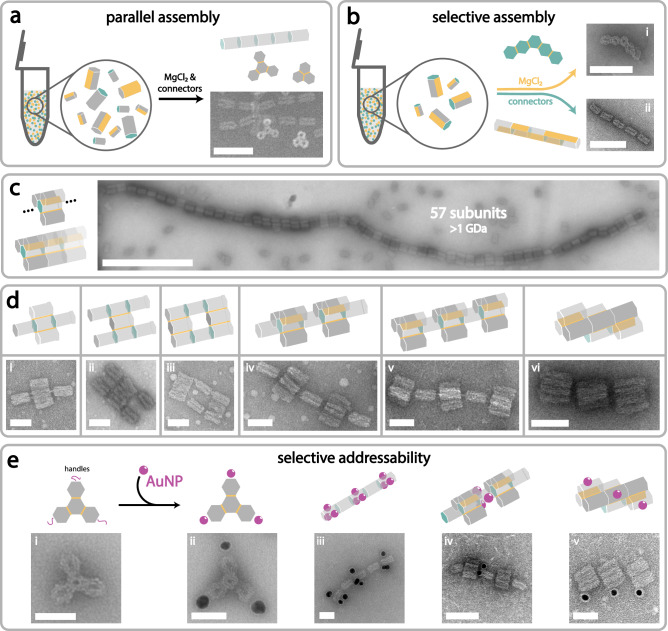
Parallel and selective assembly with retained monomer addressability. **a** The orthogonality between each xy- and z-connection allows for parallel formation of several different structures in the same tube. xy-connection sites are indicated in yellow, z-connection sites in teal. **b** Structures with both, xy- and z-connections are built, which selectively form either a distinct xy-pentamer or a z-pentamer, depending on the nature of the added assembly trigger (MgCl_2_ for xy-connections, connector strands for z-connections). **c** Large structures connected in three dimensions are formed by combining xy- and z-connections. The tubes are constructed with a tetrameric subunit in xy-direction that can infinitively stack in the z-direction. The multimeric tubes yield sizes of up to 57 subunits, corresponding to a calculated cumulative mass of 1.28 GDa. However, it should be noted that a small fraction of subunits are not fully tetrameric. **d** Exemplary xyz-direction superstructures made from various moDONs in one-step-reactions (i-v) or hierarchically assembled (vi). Pictogram indicates designed structure; yellow and teal indicate connection sites. **e** Addressability in moDON superstructures is retained beyond a single monomer. ssDNA handles (purple) are positioned exclusively on (ii) the outmost positions of the xy-tetramer, (iii) middle and ends of the z-pentamer, (iv) middle of the xyz-undecamer, and (v) at one monomer of each trimer in the twisted trimeric trimer, therefore, controlling gold nanoparticle (Au NP, purple) arrangement within the superstructure. Scale bars are 100 nm for **a** and **b**, 500 nm for **c**, and 50 nm for **d** and **e**.

As all xy- and z-connections are orthogonal to one another, we hypothesized that we would be able to assemble different superstructures from orthogonal, preformed monomers in parallel, at the same time, in the same tube. To demonstrate parallel assembly, we formed simultaneously (i) a trimer from moDONs with active α, β, and γ sides, (ii) a tetramer from moDONs with active δ, ε, and ζ sides and (iii) a pentamer in z-direction, in a one-step reaction. After addition of the respective triggers (MgCl_2_ for xy-assembly and connector strands for z-assembly) structures were analyzed by AGE and TEM. Encouragingly, species with three different electrophoretic mobilities could be observed in the gel, corresponding to trimers, tetramers, and pentamers as desired. This was further confirmed by TEM analysis, where many structures of all three species could be seen (see Fig. 4a and S56). This not only illustrates the amount of unique connection sites and their respective orthogonality, but also powerfully demonstrates the trigger-dependent selectivity of the assembly process, which we now explore further.

After having successfully established that our approach can yield several desired structures in parallel, we next sought to achieve selective assembly of different structures from the same moDONs. For this, we constructed five moDONs, each with active xy- and z-connection sites capable of forming a pentamer either in the xy- or the z-direction, depending on the respective trigger (MgCl_2_ or connectors) added. Encouragingly, we indeed found that almost exclusively one or the other trigger-specific pentamer was constructed from the same set of moDONs. As can be seen in Fig. 4b (and S57) an increase in MgCl_2_ concentration resulted in xy-pentamers, whereas an addition of the connector strands resulted in z-pentamers. This further demonstrated the orthogonality and thus specificity of the connections, constituting the foundation for assembling very large structures.

Until now we only showed the controlled, finite assembly of superstructures. However, due to the xy-z orthogonality, a combination of both assembly methods can be used to achieve even larger-sized superstructures. To demonstrate this, we aimed to assemble periodic structures in the z-direction with varying xy-directional subunits (trimers or tetramers) in a single assembly step. Note that only one of the moDONs in the trimeric or tetrameric subunit was designed to contain z-directional connection sites at both ends for periodic assembly (indicated in teal in Fig. 4c). Most previously reported large hierarchically assembled superstructures were formed in multi-step assemblies, achieving larger and larger structures with every assembly step^25,33^. Here, trimeric or tetrameric subunits and subsequent infinite periodic structures thereof, were all successfully formed in a single step, evidenced by TEM analysis (Fig. 4c, and S58, 59). The representative micrograph of periodic tetrameric subunit assemblies in Fig. 4c, displays the largest structures we observed, which were made up of 57 tetrameric subunits, with lengths reaching almost 3 µm and a calculated molecular weight of over 1 GDa. To further analyze the properties of our periodic structures we extracted the persistence length L_p_ from fitting the worm-like-chain (WLC) model to datasets of contour length and end-to-end distance of N > 50 structures each (see Figures S52d, S58d, and S59d). For structures with a monomeric subunit (Figure S52) we found L_p_ = 0.93 ± 0.16 µm, which further increased to L_p_ = 1.10 ± 0.15 µm for a trimeric subunit, and even further to 1.76 ± 0.25 µm for a tetrameric subunit. The L_p_ obtained for our structures are comparable or even larger than the L_p_ reported for scaffolded DNA origami 6HB, suggesting that our connection strategy results in comparably rigid connections. Although only assemblies from monomeric, trimeric and tetrameric subunits were shown, the approach can easily be extended to any other possible xy-assembly, making it a highly versatile method. To further demonstrate the possibility of combining xy- and z-assemblies we constructed additional finite structures. As can be seen in Fig. 4d(i) to (v) (and Figures S60, S61) we were able to construct complex finite structures from up to eight unique moDON monomers in simple one-pot reactions (see Figures S62–67 for yield analyses).

To demonstrate that our approach can also be included in a hierarchical assembly, we constructed a twisted nonamer (trimeric twisted trimer) from three trimers in a two-step assembly, without intermediate purification, shown in Fig. 4d(vi) (and S68). All trimers used the same xy-connections for the first assembly step, but carried different z-connections, used in the second assembly step. Expectedly, the yield of the trimeric twisted trimer (68.5 % in AGE, and 43.5 % in TEM, see Figure S69) is approximately the product of the yield of three trimers in Fig. 2d(iv) and S19.

In addition to construction of arbitrarily shaped superstructures, another desirable goal in hierarchical assembly is to retain the ability of modifying each point in the superstructure selectively. This is often not possible in traditional hierarchical assembly approaches. Therefore, to validate that using the moDON approach, targeted addressability of single moDONs within a superstructure can be retained, we selectively conjugated DNA-coated Au NPs to specific sections of the assembled structures. Specifically, we designed an xy-tetramer (ii), a z-pentamer (iii), a xyz-undecamer (iv), as well as the hierarchically assembled twisted trimeric trimer (v) where only specific monomers displayed anti-handles complementary to the DNA-Au NPs. Schematic illustrations of superstructure and anti-handle placement are shown in Fig. 4e. Analysis of the assembled structures by TEM clearly shows that Au NPs only attached to those monomers displaying the anti-handle, while all other moDON monomers in the superstructure remained bare (see Fig. 4e, and S70–S73). However, unlike in the GDa-sized structures in ref. ^24^, addressability of our infinite GDa-sized structures is not retained beyond the (multimeric) subunit. Nevertheless, we were able to confirm that indeed, using the moDON, site-specific addressability of each specific monomer in a finite superstructure is fully retained and can be used for the selective modification with functional guest molecules such as NPs or proteins. This could enable further functionality for synthetic cell design allowing e.g. the selective attachment and transport of cargo along an artificial cytoskeleton^36^. Although not demonstrated here, the modular design of the moDON could also allow for different modifications (e.g. with different proteins) at pre-designed positions within the same superstructure.

**Fig. 5:**
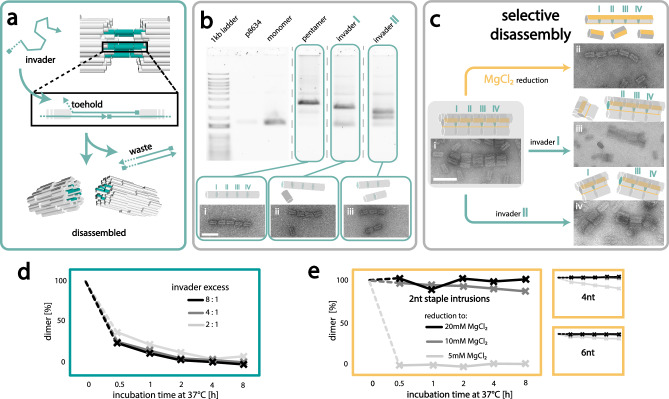
Disassembly of moDON superstructures. **a** Schematic of z-directional disassembly: Connector strands are elongated by a short ssDNA toehold. An invader strand, fully complementary to the elongated connector is able to hybridize first to the toehold, and then detach the connector from the ssDNA handles on the moDONs. This results in z-directional disassembly. **b** Selective disassembly of z-connections. The unique sequence of each connector allows for selective disassembly of single bonds in the superstructure. Shown here is the invasion of connector I or connector II, by their respective invaders, resulting in disassembly into tetramers and monomers (ii), or dimers and trimers (iii) respectively. **c** Selective disassembly of a 20-mer xyz-superstructure: Reduction of MgCl_2_ leads to disassembly of xy-connections, leaving only z-directional pentamer and monomers intact (ii). Addition of invader strands I or II, leads to selective disassembly of connections I or II, leaving tetramers and 16-mers (iii) or octamers and dodecamers (iv), respectively. **d** Disassembly speed of z-connections is marginally depending on invader excess. 2-fold excess of invader strands over connector strands in the solution shows slightly slower disconnection than 4- or 8-fold excess. **e** Disassembly speed of xy-connections in low MgCl_2_ buffer is highly dependent on staple intrusion length. xy-connections with only 2 nt staple intrusions dissociate fast, in low MgCl_2_ buffer. Longer staple intrusions of 4 nt or 6 nt stabilize dimers, proportional to the staple intrusion length. For 6 nt staple intrusions the proportion of dimers is still ~90 % after 8 h. Scale bars are 100 nm.

In nature, functional protein complexes or microtubules are trigger-dependently assembled and disassembled. Thus, after demonstrating controlled and trigger-specific assembly of superstructures as well as their retained addressability, we lastly investigated, if such superstructures could analogously be selectively disassembled, mimicking cellular systems. We initially tested disassembly for the z-connection. Due to the connector strand-mediated assembly of moDONs in the z-direction, removal of the connector by toehold-mediated strand displacement poses an excellent tool. For this, the connectors were extended to display a connector specific, short ssDNA toehold region of 7 nt. The addition of fully complementary invader strands could then selectively remove the connector strand (see NUPACK simulations in Figures S74, S75 and Table S15) and thus result in superstructure disassembly as schematically depicted in Fig. 5a. To illustrate the specificity of the connector-invader pairs, as well as their orthogonality towards the other pairs, we investigated the selective partial disassembly of a z-pentamer. Depending on the sequence of the invaders added, pentamers should selectively disassemble into various ensembles ranging from tetramers to monomers. Encouragingly, analysis by AGE and TEM showed that the programmed disassembly process is indeed highly selective and highly effective, disassembling the pentamers into the desired substructures of tetramers, trimers, dimers, and monomers as observable in Fig. 5b (and S76–S79). To further showcase the effectiveness of this methodology, we next demonstrated the selective disassembly of periodic tubes made up from either monomeric or dimeric subunits in the z-direction. Once more, AGE revealed the highly effective and selective disassembly into monomeric (Figure S80a, b) or dimeric subunits (Figure S80c, d) as desired. Further examining the influence of different conditions on z-disassembly, we observed that neither the amount of invader strand added (2-, 4-, or 8-fold excess over connector strands), nor the concentration of MgCl_2_ (in a range of 5 – 20 mM) had any significant effect on disassembly kinetics or yield with almost all structures being fully disassembled after ~2 h if the correct invader was added (see Fig. 5d and S81a). However, incubation temperature was found to play a role. Z-connections incubated at 37 °C were fully disassembled after 4 h (Figure S55b, d). Z-connections incubated at 20 °C or 4 °C still appeared to be dimerized (> 50%), even after 8 h of incubation. Analogously to the assembly of z-connections, we attribute this effect to the diffusion speed of the invaders increasing with temperature and thus the increasing interaction probability and strand-displacement velocity. This confirms that the main trigger for z-disassembly is the type of invader strand added, while incubation temperature also impacts disassembly speed. The concentration of MgCl_2_ in the buffer, however, played no significant role.

While the three-strand connection system in the z-direction excellently lent itself for disassembly by toehold-mediated strand displacement, xy-assemblies could not easily be disassembled using the same method. We therefore investigated other parameters to achieve selective disassembly in the xy-direction. We hypothesized that if short lengths of the staple intrusions were utilized, disassembly could be mediated by reducing the MgCl_2_ concentration, as had been reported in the literature^26^. To test this hypothesis, dimeric structures containing staple intrusions of 2, 4, or 6 nt were exposed to different MgCl_2_ concentrations for different reaction times at 37 °C. While for assembly kinetics, reaction time and MgCl_2_ concentration played an important role, as discussed earlier, for the disassembly kinetics the number of nt in the staple intrusions were found to be crucially important. As can be seen in Fig. 5e (and S82), no dimer disassembly could be observed in 10 or 20 mM MgCl_2_ supplemented buffer for dimers formed with 4 and 6 nt staple intrusions. However, for the shortest system of only 2 nt staple intrusion length, a small number of monomers with higher electrophoretic mobility could be observed. Full disassembly of dimers into the respective monomers was only achieved for structures formed with 2 nt intrusions if the MgCl_2_ concentration in the buffer was reduced to 5 mM. As can be seen in Fig. 5e (and S82), these dimers were fully disassembled after only 30 min, while all other dimers formed with longer staple intrusions did not fully disassemble even after 8 h under these conditions. This suggests that structural integrity of xy-connections is highly dependent on staple intrusion length, and MgCl_2_ concentration, which can be explained with the lower free energy funnel from longer staple intrusions and decreased shielding of backbone repulsion from lower MgCl_2_ concentrations. Consequently, less stable xy-connections, with only 2 nt staple intrusions could be used for selective disassembly.

We thus far showed that both xy- and z-directional assemblies can be selectively assembled and disassembled. We further showed that not only the triggers for inducing assembly (*i.e*. connector strand- and MgCl_2_-induced hybridization), but also the triggers of disassembly (*i.e*. addition of invader strands or reduction of MgCl_2_ concentration) are fully orthogonal. This should allow for selective disassembly of large superstructures into any desired subunit, breaking either xy or z-connections. To test this, we designed a 20-mer, consisting of five tetrameric subunits in xy-direction, which further assembled into a pentamer of tetramers in the z-direction. The successful formation of the tetrameric pentamers was confirmed by TEM analysis (Fig. 5c(i) and S83). Subsequently, we exposed the assembled structure to the different disassembly triggers, *i.e*. low MgCl_2_ concentrations or invader strands in order to achieve either monomeric pentamers or tetramers of defined lengths (monomeric, dimeric etc.). Supporting our hypothesis, analysis by TEM revealed that dependent on the trigger added, structures could selectively be disassembled into the desired subunits: When the MgCl_2_ concentration was reduced to 5 mM, only the xy-connections disassembled, resulting in a z-directional pentamer and a large number of monomers (Fig. 5c(ii) and Figure S84). Conversely, addition of invader I resulted in monomeric tetramers as well as tetrameric tetramers, (Fig. 5c(iii) and Figure S85), suggesting that only the first z-connection was released, as desired. Analogously, the addition of invader II resulted in disassembly of the second z-connection, keeping all other z-connections intact and thus the tetrameric pentamer was split into dimeric and trimeric tetramers (Fig. 5c(iv) and Figure S86). With this we finally showed that the moDON can be assembled and disassembled with complete xy-z orthogonality giving absolute and complete control over the process.

## Discussion

In summary, we developed re-routable scaffolds as design paradigm for DNA origami to introduce modularity to shape-matching connections and developed a modular three-strand-system that circumvents the loss over structural control, dynamic convertibility, and the need for many unique connectors, which were previously problematic for these connections. We showcased the power of modularity in DNA origami design by engineering one moDON, with tens of thousands of different connection site configurations. The large number of orthogonal connections allows one-step assemblies into a diverse variety of superstructures. These can be finite (super-)structures with fully retained site-specific addressability, or periodic structures reaching large scales of µm and GDa.

We introduced modularity to two connection strategies^26,27,35^. Modularity in xy-direction was achieved via scaffold re-routing, subverting the traditional way of DNA origami design of rigid scaffold routings. Modularity in z-direction was achieved with well-positioned, directional, and optimally sized handles for an orthogonal three-strand-system, which circumvents all issues of this kind of connection: lack of rigid, unintended passivation, branching, non-removable connections, lack of directionality, and/or the need for many unique connections strands for each connection. With six positions in the xy-direction, each being able to form one of two connection sites (α-ζ) or be passive, and two opposite positions in the z-direction being able to form one of eight connection sites or be passive, the total number of unique monomers that can be formed through the modularity of just one single moDON is:

$$	 {(2\,{{\rm{xy}}}\; {{\rm{configs}}}.+{{\rm{passive}}})}^{6{{\rm{sites}}}} \, * \,{(8\,{{\rm{z}}}\; {{\rm{configs}}}.+{{\rm{passive}}})}^{2 \, {{\rm{sites}}}} \\ 	=59049 \, {{\rm{unique\; monomers}}}$$

With 59 049 unique monomers possible from a single moDON set of staples, our method significantly reduces cost (total number of staples needed are only ~ 2.1 × the number needed for a single structure (Table S18)), and design time compared to individual design of each DNA origami. To change the configuration of one connection site only ~ 2.5 % of the staples need to be exchanged.

The modular construction of DNA origami significantly decreases the cost and effort for superstructure design whilst having a large versatility in possible assemblies. However, the modularity alone does not introduce fundamentally different kinds of DNA-DNA connections and is not able to fully solve existing issues with respective connection strategies. Connections based on protrusions and indentations have a low stability, limited by the number of base pairs in each connection. An increase of bp in the connection, on the other hand, could undermine structural stability or facilitate the formation of topological traps. Extending the number of DNA layers in the connections from two to four^28^ could also increase the stability but would increase the complexity of the modular design. Protrusions and indentations are also limited by the amount of helix configurations, while still avoiding symmetries and similarities of connection sites, as well as the formation of overhanging scaffold loops, prone to off-target interactions. The rigidity of z-connections is prone to missing connector strands. In general, unintended and unpredictable interactions between ssDNA strands become more likely with an increasing number of nt involved. Unfortunately, this presents a universal problem for all DNA-DNA connections, and an efficient way to overcome these limitations is yet to be found.

However, compared to other methods of superstructure assembly, the modular design excels in versatility, cost-effectiveness, and/or ease of handling. By increasing the number of connection sites and configurations thereof, we were able to increase the number of monomers in one-step reactions, increasing the size of superstructures constructed in one-pot assemblies. Further, the cost for the superstructures is decreased tremendously, since each of the diverse origami configurations is a variation of the same moDON. Both factors combined alleviate the problems of high cost, lack of versatility, and yield loss through repeated extraction and purification of intermediates in traditional hierarchical assembly. We showed further, that the moDON superstructures can also be hierarchically assembled, enabling the construction of even larger, more complex structures. However, 3D SST assemblies^20–22^ exceed all other published structures to date in versatility and addressability, including ours, as every tile is separately adjustable and addressable. The largest fully addressable SST structures reached ~ 0.5 GDa^22^, ~ 10 times larger than the fully addressable structures here, but the monetary cost, on the other hand, was far beyond $ 100,000. Here, modular-designed origami are much more cost-effective and easier to handle than the SST structures from 30,000 ssDNA oligomers. The largest fully addressable structures achieved to date are origami crisscross assemblies^24^, reaching several µm and several GDa for structures in 2D. The size and weight of our fully addressable assemblies is far below those. Here, however, the moDON assemblies excel in the variety of scales and forms they can be assembled into (e.g. twisted and curved structures), and also disassembled from. Other three-strand systems^27,29–32^ (to the here presented z-connections) were able to connect origami to various forms. But those connection strategies need a large number of unique, long ssDNA strands, and/or do not have control over the connection properties, like stoichiometry or rigidity, whereas the here presented z-connections only require *one* single kind of 21nt-short ssDNA strand for each stable, directional, orthogonal, and dynamically controllable connection, decreasing the risk of unwanted interactions and increasing the number of orthogonal connections designable. Using only one unique z-connector per connection site, to control the dynamic assembly and disassembly process makes this approach also feasible for incorporation into strand displacement networks, unlike structures needing dozens of unique sequences. Further, the small number of ssDNA nucleotides involved in each modular xy-connection in combination with the structurally orthogonal indentations and protrusions makes this design strategy immune to off-target interactions. Additionally, the here constructed moDON folds with quantitative yield, which leads to a comparably higher *effective yield* of the superstructures, a trait that is often overlooked and a prominent issue in many structures reported thus far for (hierarchical) assembly.

In its capacity to assemble and disassemble into and from a large variety of shapes and scales on biologically relevant size- and timescales, the moDON could have great potential in synthetic biology. For example, the long tubular superstructures (Figs. 3g and 4c) could lend themselves as cytoskeleton-mimicking structures, where assembly and disassembly could be controlled by DNA-strand displacement circuits^36,37^. Especially the z-connections with their minimized number of connection strands, and still retained structural control are suited for the dynamic assembly and disassembly in structures. Compared to DNA nanotubes, mostly used to mimic the natural cytoskeleton in synthetic biology, the here constructed periodic tubes have a very large subunit, predisposing them for the incorporation of more complex approaches to multifunctionality, e.g. molecular motors^9^ that could alternatively also be used for the construction of artificial flagella in synthetic biology, or hinged elements^38^.

The here presented methods of modularity should prove a powerful addition in the toolbox of DNA origami construction. We expect the xy-z orthogonal modularity and connections design to be adaptable to any other DNA origami structure, not only the moDON, potentially even allowing for further expansions of orthogonal connection sites, and presenting itself as an addition or even alternative to hierarchical assembly. All in all, these features present the modular approach to DON superstructure construction as a promising tool for the construction of large superstructures with high control, suitable for synthetic biology applications.

## Methods

### DNA Origami Design, Synthesis and Purification

DNA origami structures were designed with caDNAno^8^ version 2.4.10 and simulated with oxDNA^39–42^ version 3.5.0 with 100 000 000 iterations at standard settings, after relaxation with 5000 CPU and 1 000 000 GPU iterations. DNA sequences for the three-strand-system were analyzed with NUPACK^43^ version 2.2.

Unmodified DNA oligos were purchased from IDT, desalted and dispersed in IDTE pH 8.0 at 100 µM. The respective sequences can be found in the Supplementary Information. Scaffold DNA was produced as described in ref. ^44^. DNA was stored at −20 °C until further use. Folding was performed on a Biometric TRIO thermocycler from Analytic Jena, or a MJ research PTC-200. If not further specified, the folding buffer consisted of 1× TAE (40 mM Tris, 20 mM acetic acid, 1 mM EDTA, pH 8.2) supplemented with 15 mM MgCl_2_. Scaffold and staples (see Figure S87a), were added to the folding buffer, denatured for 5 min at 65 °C, folded isothermally for 3 h at 50.5 °C and then held at 4 °C until further use (see Figure S87b). Purification of folded structures was performed with Amicon centrifugal filters (Merck Millipore, cat.no.: UFC5100) and TAE buffer with 5 mM MgCl_2_ in five washing steps at 8 000 × g for 4 min. The purified structures were stored at −20 °C until further use.

Assemblies of moDONs to superstructures were performed, if not further specified, by addition of MgCl_2_ to 40 mM for xy-connections or 5-fold excess of connectors over handles for z-connections to a moDON ensemble. Disassembly of superstructures was performed, if not further specified, by addition of 5-fold excess of invader strands over connector strands. If not further specified, z-assembly and disassembly were performed at 20 mM MgCl_2_. Each ensemble of moDONs was, if not further specified, equimolar with respect to the different monomers (usually 1 or 2 nM), in TAE (40 mM Tris, 20 mM acetic acid, 1 mM EDTA, pH 8.2) and incubated at 37 °C over-night.

### Agarose Gel Electrophoresis

AGE was performed, unless further specified, with 1 % agarose gels, TAE running buffer (40 mM Tris, 20 mM acetic acid, 1 mM EDTA, pH 8.2) supplemented with 11 mM MgCl_2_ and SYBR safe (invitrogen, cat.no.: S33102) at 70 V, for 90 min and on ice. Gels were imaged on a typhoon FLA 9000 laserscanner. AGE yield was analyzed with the built-in gel analysis tool from Fiji^45^ version 2.9.0 (−2.14.0) and extracted as the fraction of surface area from gel plots.

### TEM

Carbon-coated copper grids (Plano GmbH, cat.no.: S162-3) were treated for 30 s with oxygen plasma, 10 fmol of the respective sample was incubated on the grid for 5 min, the solution removed with filter paper, and subsequently stained with 5 µl of a 2 % uranylformate solution for 10 s, which was again removed with filter paper. The grid was air-dried before imaging. Imaging was performed on a Jeol-JEM-1230 transmission electron microscope, operating at an acceleration voltage of 80 kV. Micrographs were analyzed with Fiji^45^ version 2.9.0 (and later) and yields analyzed by counting DNA origami monomers and calculating the fraction in the desired superstructure. MATLAB R2020b was used to fit the persistence length via Eq. (1)^46^ with R being the end-to-end distance, ${l}$ the contour length, and ${L}_{p}$ the persistence length:

1 $$ < \, R \, { > }_{2D}^{2}=4\,l\,{L}_{p}\left[1-\frac{2\,{L}_{p}}{l}\left(1\,-\,{e}^{-\frac{l}{2{L}_{p}}}\right)\right]$$

### Au NP Synthesis and moDON Modification

Au NP were synthesized as described in ref. ^47^. In brief, 50 mL of 1 mM hydrogen tetrachloroaurate in milliQ water was heated in aqua-regia-cleaned glassware to a rolling simmer, stirring vigorously with a magnetic stir bar. Then 400 µl of a (also simmering) 2 % trisodium citrate solution was added. After a color change to dark red, the heat was turned off, and the solution stirred for an additional 15 min, and then put aside to cool to room temperature. The solution was then concentrated by centrifugation at 10 000 × g for 10 min and re-dispersion of the pellet in milliQ water. The concentration was determined from absorption measurements taken with a Nanodrop 1000. Samples were stored at 4 °C until further use.

Au NPs were subsequently functionalized with thiolated DNA oligos (biomers.net, HPLC purified, resuspended in milliQ water). For this, thiolated DNA was added to the Au NPs in 12 500-fold excess and subsequently frozen at −20 °C. Functionalized Au NP were purified via four steps of centrifugation at 20 000 × g for 10 min, subsequent supernatant removal and addition of the same amount of milliQ water. Concentrations were measured from absorption measurements taken with a Nanodrop 1000. Samples were stored at 4 °C until further use.

moDON-Au NP constructs were formed by addition of moDON to DNA-coated-Au NPs while simultaneously vortexing at a low level, followed by 1 h of incubation at room temperature. DNA-Au NP constructs were added with at least 5x excess over handles on the moDON. Constructs were optionally purified by AGE, with the above specified parameters.

## Supplementary information

Supplementary Information

Transparent Peer Review file

## Source data

Source Data

## Data Availability

All data supporting the findings of this study are available within the main text, supplementary information files, source data files, and from the corresponding author upon request. caDNAno files of the structure are also available on nanobase.org/structure/263. Source data are provided with this paper.
