# Author Correction: Fully addressable designer superstructures assembled from one single modular DNA origami

**DOI:** 10.1038/s41467-025-57186-x

**Published:** 2025-02-21

**Authors:** Johann M. Weck, Amelie Heuer-Jungemann

**Affiliations:** Max Planck Institute of Biochemistry, Am Klopferspitz 18, 82152 Martinsried, Germany and Center for Nanoscience, Ludwig-Maximilians University, Munich, Germany

**Keywords:** DNA nanotechnology, DNA

Correction to: *Nature Communications* 10.1038/s41467-025-56846-2, published online 12 February 2025

In the version of the article initially published, the origami structures in Fig. 1b were a duplicate of those in Fig. 1d and have now been corrected in the HTML and PDF versions of the article.

