# Supplementary material for: Fully addressable designer superstructures assembled from one single modular DNA origami
Source: Nat Commun. 2025 Feb 12;16:1556. doi: 10.1038/s41467-025-56846-2 (PMC11814417; doi:10.1038/s41467-025-56846-2)
Supplement: Supplementary file 1 — Supplementary Information [file 41467_2025_56846_MOESM1_ESM.pdf]

Supplementary Information for

# Fully addressable designer superstructures assembled from a single modular DNA origami

*Johann M. Weck[1,*], and Amelie Heuer-Jungemann[1,*]*

[1]Max Planck Institute of Biochemistry, Am Klopferspitz 18, 82152 Martinsried, Germany and Center for Nanoscience, Ludwig-Maximilians University, Munich, Germany

## Table of Figures


**Figure S1: connectivity overview** ... 6
**Figure S2: layouts of the moDON in configurations 1 and 2** ... 7
**Figure S3: Scaffold routing in core and shell** ... 8
**Figure S4: Staple routing and positioning of corrections** ... 9
**Figure S5: caDNAno scaffold routing** ... 10
**Figure S6: Helix numeration and scaffold loop corrections** ... 11
**Figure S7: Overview over connection sites and configurations** ... 12
**Figure S8: oxDNA simulation of the moDON in configuration 1 (αβγ)** ... 13
**Figure S9: oxDNA simulation of the moDON in configuration 2 (δεζ)** ... 14
**Figure S10: AGE analysis of folded monomers with $MgCl_2$ titration** ... 15
**Figure S11: Analysis of moDON monomers** ... 16
**Figure S12: AGE gel shift assay of dimer/trimer permutations 1.** ... 17
**Figure S13: AGE gel shift assay of dimer/trimer permutations 2.** ... 18
**Figure S14: close-up view on xy-structures 1** ... 19
**Figure S15: Wide-field TEM micrograph and statistics of xy-dimer formation** ... 20
**Figure S16: Wide-field TEM micrograph and statistics of xy-trimer formation 1** ... 21
**Figure S17: close-up view on xy-structures 2** ... 22
**Figure S18: AGE shift assay of xy-structures** ... 23
**Figure S19: Wide-field TEM micrograph and statistics of xy-trimer formation 2** ... 24
**Figure S20: AGE gel shift assay of tetramers** ... 25
**Figure S21: Wide-field TEM micrograph and statistics of xy-tetramer formation 1** ... 26
**Figure S22: Wide-field TEM micrograph and statistics of xy-trimer formation 2** ... 27
**Figure S23: close-up view on xy-structures 3** ... 28
**Figure S24: AGE gel shift assay of hexamers.** ... 29
**Figure S25: Wide-field TEM micrograph and statistics of xy-hexamer formation 1** ... 30
**Figure S26: Wide-field TEM micrograph and statistics of xy-hexamer formation 2** ... 31
**Figure S27: close-up view on xy-structures 4** ... 32
**Figure S28: Wide-field TEM micrograph and statistics of xy-heptamer formation 1** ... 33
**Figure S29: Wide-field TEM micrograph and statistics of xy-heptamer formation 2** ... 34
**Figure S30: Wide-field TEM micrograph and statistics of xy-heptamer formation 3** ... 35
**Figure S31: Wide-field TEM micrograph and statistics of xy-heptamer formation 4** ... 36
**Figure S32: Yield overview of AGE and TEM yields** ... 37
**Figure S33: Infinite xy-structures** ... 38
**Figure S34: AGE shift assays of xy-assembly 1** ... 39
**Figure S35: AGE shift assays of xy-assembly 2** ... 40
**Figure S36: AGE shift assay on temperature influence on xy-assembly** ... 41
**Figure S37: NUPACK analysis of z-connector orthogonality** ... 42
**Figure S38: z-assembly permutations** ... 43
**Figure S39: Wide-field TEM micrograph and statistics of z-dimer formation** ... 44
**Figure S40: close-up view on z-structures 1** ... 45
**Figure S41: close-up view on z-structures 2** ... 46

**Figure S42: Wide-field TEM micrograph and statistics of z-trimer formation** ........ 47
**Figure S43: Wide-field TEM micrograph and statistics of z-tetramer formation** ........ 48
**Figure S44: Wide-field TEM micrograph and statistics of z-pentamer formation** ........ 49
**Figure S45: symmetrically assembled z-structures** ........ 50
**Figure S46: close-up view on z-structures 3** ........ 51
**Figure S47: close-up view on z-structures 4** ........ 52
**Figure S48: Wide-field TEM micrograph and statistics of z-hexamer formation** ........ 53
**Figure S49: Wide-field TEM micrograph and statistics of z-heptamer formation** ........ 54
**Figure S50: Wide-field TEM micrograph and statistics of z-octamer formation** ........ 55
**Figure S51: Wide-field TEM micrograph and statistics of z-nonamer formation** ........ 56
**Figure S52: Infinite tube with monomeric subunit** ........ 57
**Figure S53: z-connections with various connector concentrations** ........ 58
**Figure S54: AGE shift assays of z-assembly** ........ 59
**Figure S55: AGE shift assay on temperature influence on z-assembly and disassembly** ........ 60
**Figure S56: Parallel assembly** ........ 61
**Figure S57: Selective assembly** ........ 62
**Figure S58: Infinite tubes with trimeric subunits** ........ 63
**Figure S59: Infinite tubes with tetrameric subunits** ........ 64
**Figure S60: close-up view on xyz-structures 1** ........ 65
**Figure S61: close-up view on xyz-structures 2** ........ 66
**Figure S62: AGE analysis of xyz-structures** ........ 67
**Figure S63: Wide-field TEM micrograph and statistics of xyz-pentamer formation** ........ 68
**Figure S64: Wide-field TEM micrograph and statistics of xyz-heptamer formation** ........ 69
**Figure S65: Wide-field TEM micrograph and statistics of xyz-octamer formation.** ........ 70
**Figure S66: Wide-field TEM micrograph and statistics of xyz-undecamer formation** ........ 71
**Figure S67: Wide-field TEM micrograph and statistics of xyz-14mer formation.** ........ 72
**Figure S68: hierachical xyz-nonamer "twisted trimeric trimer" assembly** ........ 73
**Figure S69: Wide-field TEM micrograph and statistics of xyz-nonamer.** ........ 74
**Figure S70: xy-tetramer with selectively placed Au NPs.** ........ 75
**Figure S71: z-pentamer with selectively placed Au NPs** ........ 76
**Figure S72: xyz-undecamer with selectively placed Au NPs** ........ 77
**Figure S73: xyz-nonamer "twisted trimeric trimer" with selectively placed Au NPs** ........ 78
**Figure S74: NUPACK analysis of z-connector orthogonality with toeholds** ........ 79
**Figure S75: NUPACK analysis of z-connectors in presence of invader strands** ........ 80
**Figure S76: z-disassembly permutations** ........ 81
**Figure S77: TEM micrographs of selective z-disassembly 1** ........ 82
**Figure S78: TEM micrographs of selective z-disassembly 2** ........ 83
**Figure S79: TEM micrographs of selective z-disassembly 3** ........ 84
**Figure S80: Assembly & disassembly of periodic z-directional superstructures** ........ 85
**Figure S81: AGE shift assay of z-disassembly** ........ 86
**Figure S82: AGE shift assay of xy-disassembly** ........ 87
**Figure S83: TEM micrographs of tetrameric pentamer assembly .** ........ 88
**Figure S84: TEM micrographs of tetrameric pentamer disassembly 1** ........ 89
**Figure S85: TEM micrographs of tetrameric pentamer disassembly 2** ........ 90

**Figure S86: TEM micrographs of tetrameric pentamer disassembly 3**......91
**Figure S87: folding conditions for the moDON monomers** ......92

## Table of tables

**Table S1: Staple connection sites and the respective staple mixes** ........ 93
**Table S2: Core staples** ........ 94
**Table S3: Staples for HH50 modular part in configuration 1**. ........ 97
**Table S4: Staples for HH55 modular part in configuration 1** ........ 98
**Table S5: Staples for HH60 modular part in configuration 1** ........ 99
**Table S6: Staples for HH65 modular part in configuration 1** ........ 100
**Table S7: Staples for HH70 modular part in configuration 1** ........ 101
**Table S8: Staples for HH75 modular part in configuration 1** ........ 102
**Table S9: Staples for HH50 modular part in configuration 2** ........ 103
**Table S10: Staples for HH55 modular part in configuration 2** ........ 104
**Table S11: Staples for HH60 modular part in configuration 2** ........ 105
**Table S12: Staples for HH70 modular part in configuration 2** ........ 106
**Table S13: Staples for HH75 modular part in configuration 2** ........ 107
**Table S14: Staples for cross-configurations of HH50** ........ 108
**Table S15: z-connections staples** ........ 109
**Table S16: 4 nt and 6 nt overlap connections** ........ 112
**Table S17: Au NP handle sequences** ........ 113
**Table S18: Number of nt for specific configurations** ........ 114

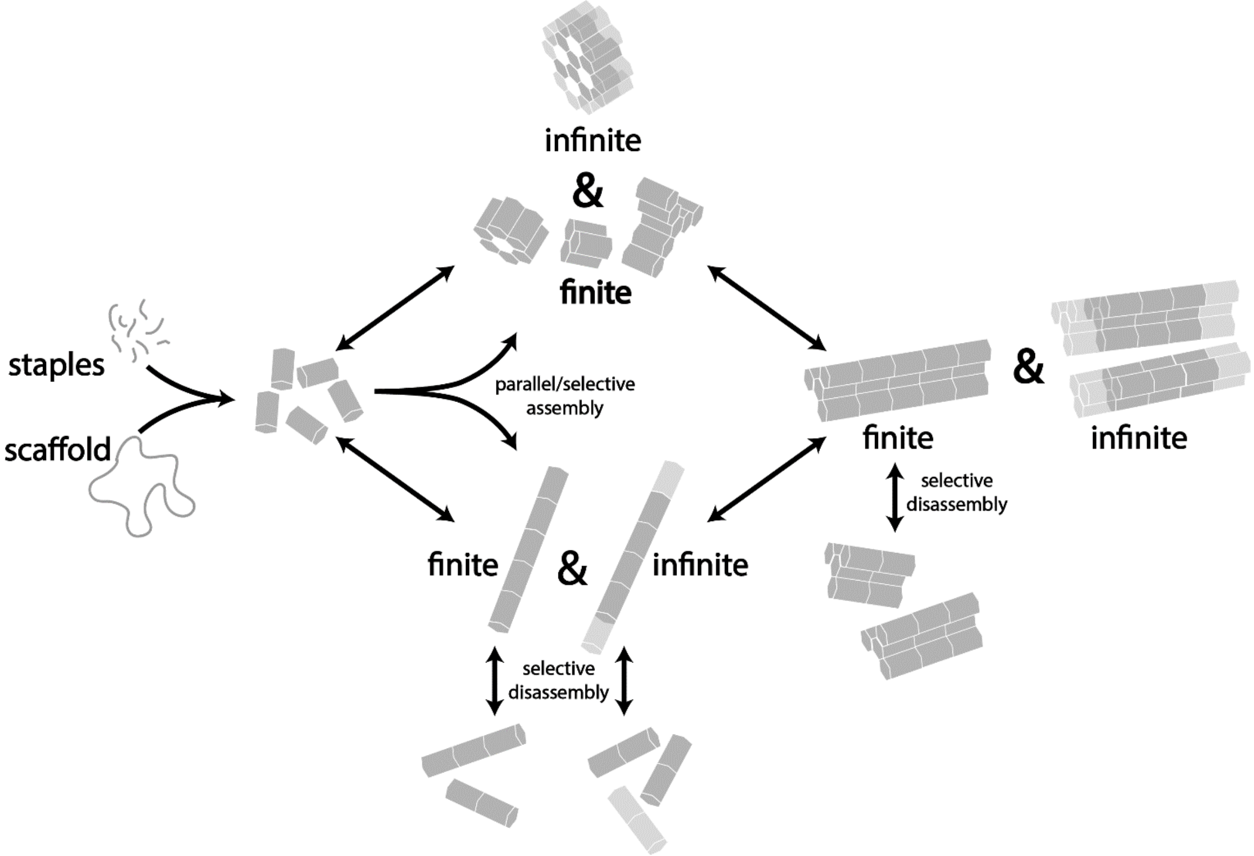


**Figure S1: connectivity overview**: different moDON monomers are folded separately. Specific superstructures are designed by a combination of moDONs with complimentary connection sites. In the xy-direction, perpendicular to the helical direction, finite and infinite structures are assembled by increasing the $MgCl_2$ concentration. In the z-direction, the helical direction, finite and infinite structures are formed by addition of connectors strands. Connections are orthogonal to each other, as well as to the connection strategies themselves. This enables *parallel* assembly of different xy-directional and z-directional structures in one reaction vessel. This also enables *selective* assembly of either xy- or z-structures, from moDONs carrying both connection sites for xy- and z- structures, depending on which trigger is added. Both approaches can be combined to form large structures in all directions. Conversely, a decrease in $MgCl_2$ concentration leads to disassembly of xy-connections. The addition of invader strands leads to selective disassembly of z-connections through toehold-mediated strand displacement. Both disassembly strategies are also orthogonal towards each other. In case of z-disassembly, the sequence specificity of the connectors and invaders also leads to orthogonality of the single connection sites towards each other.

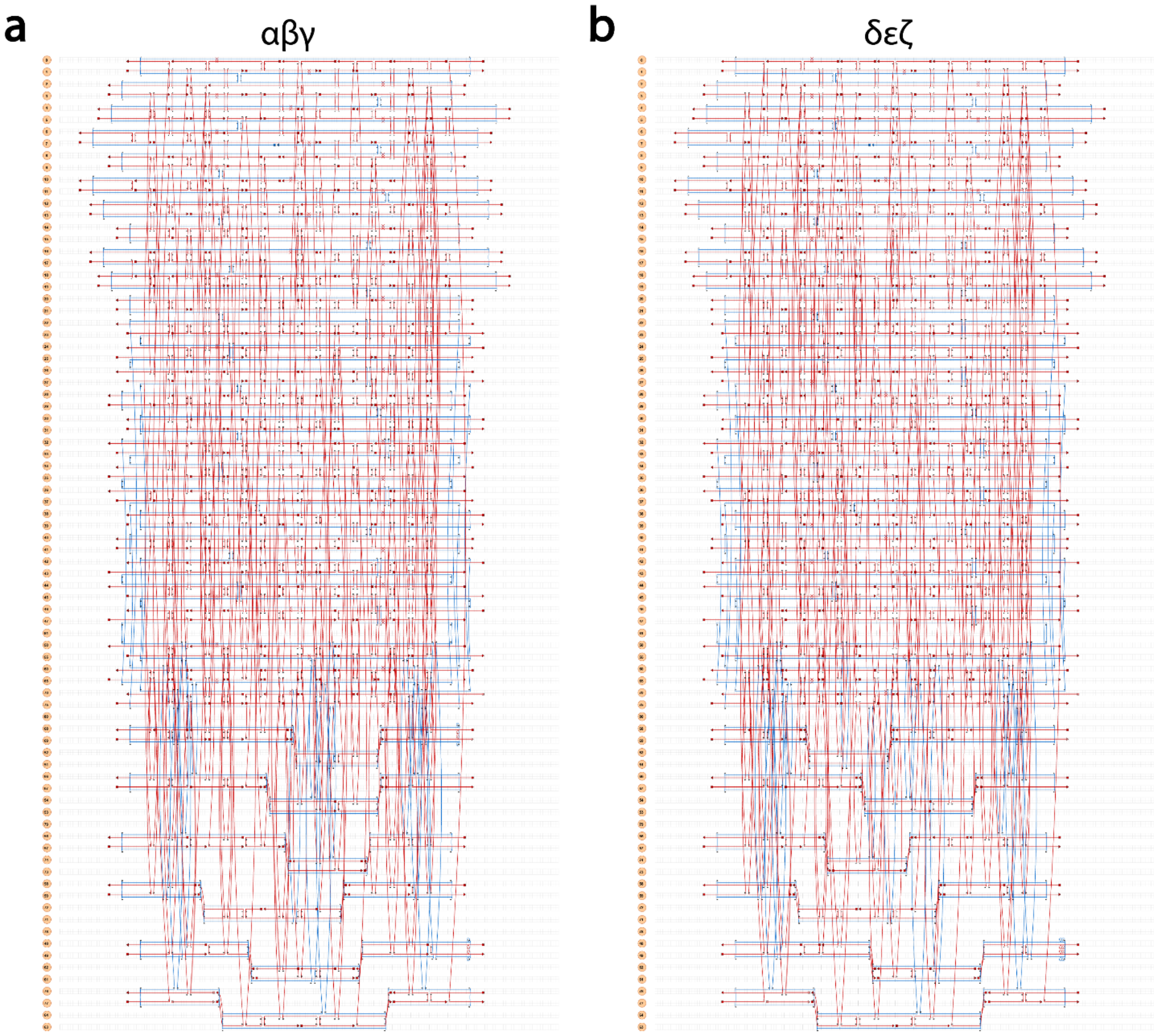


**Figure S2: layouts of the moDON in configurations 1 and 2**. Staple routing is indicated in red, scaffold routing indicated in blue. (**a**) Layout of configuration 1 and (**b**) layout of configuration 2 only differ in the size and/or position of the respective protrusions and indentations. Details of the design are described in the following figures: for schematic scaffold layering see S3, for staple motifs see S4, for connection sites see S5 and S7, and for side view, enumeration and deletions see S6. For oxDNA simulations of both configurations here see S8-S9. For staple sequences see Tables S2-S15.

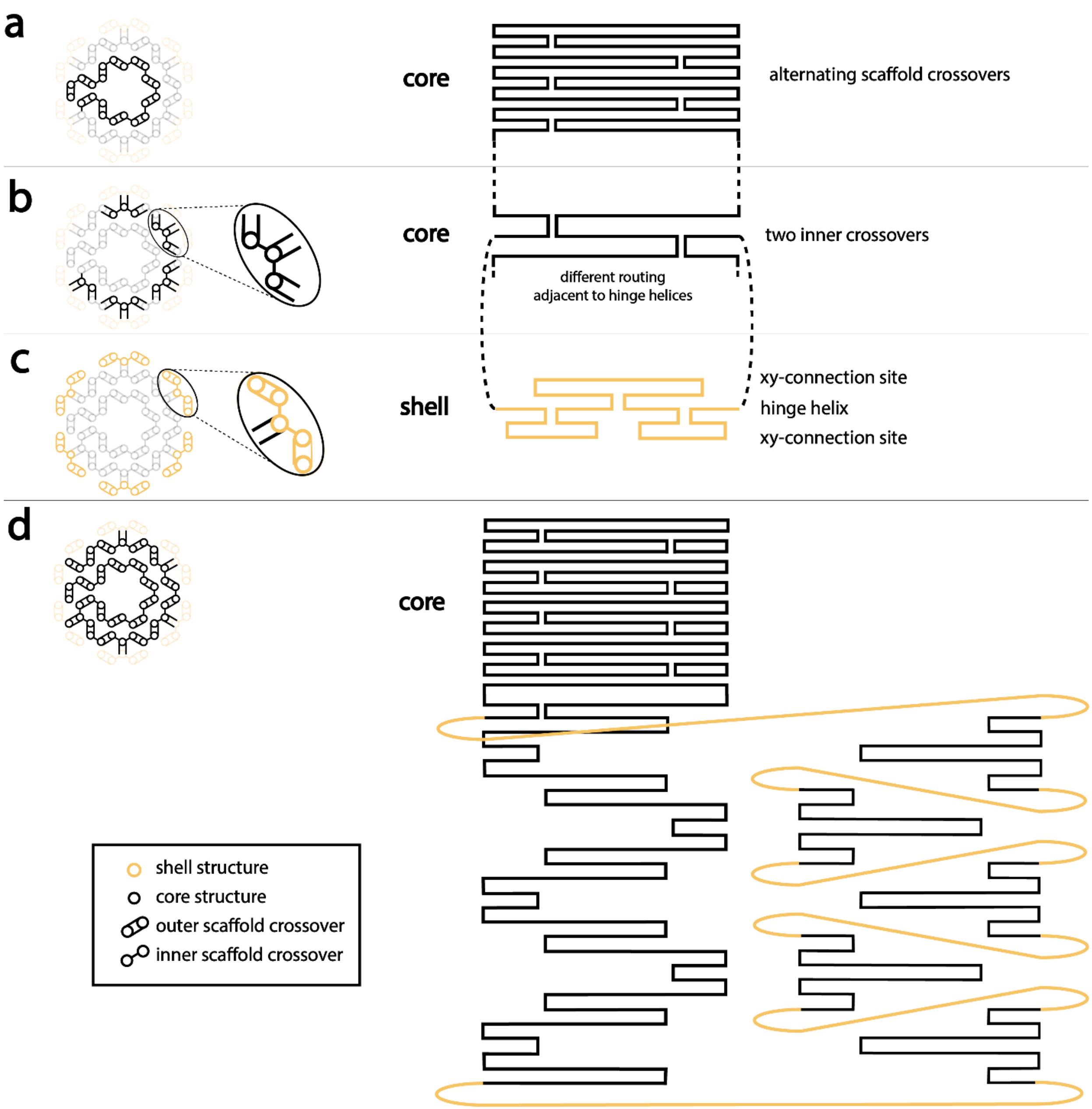


**Figure S3: Scaffold routing in core and shell:** (**a**) the inner core part (black) of the moDON is layered evenly, with alternating scaffold cross-overs at the helix ends and in the helix middle. This was done to ease the folding pathway. The positions of the scaffold cross-overs in the helix middle are varied, to avoid the introduction of an artificial breaking point. (**b**) The outer core part (black) of the moDON was not able to be layered with alternating scaffold cross-over positions. Every fifth helix has three cross-overs, to connect with a hinge helix (yellow) (**c**). From the hinge helix the xy-connection sites (yellow) loop out. The connection sites are modular and their routing is changed by exchange of a few staples. The hinge helix connects with scaffold crossovers at the helix ends back to the core structure. The connections from this adjacent helix to the second and third helix is then done with scaffold cross-overs in the middle of the helix. This leads to the scaffold routing pattern shown in (**d**), with the connection sites shown as curved yellow lines.

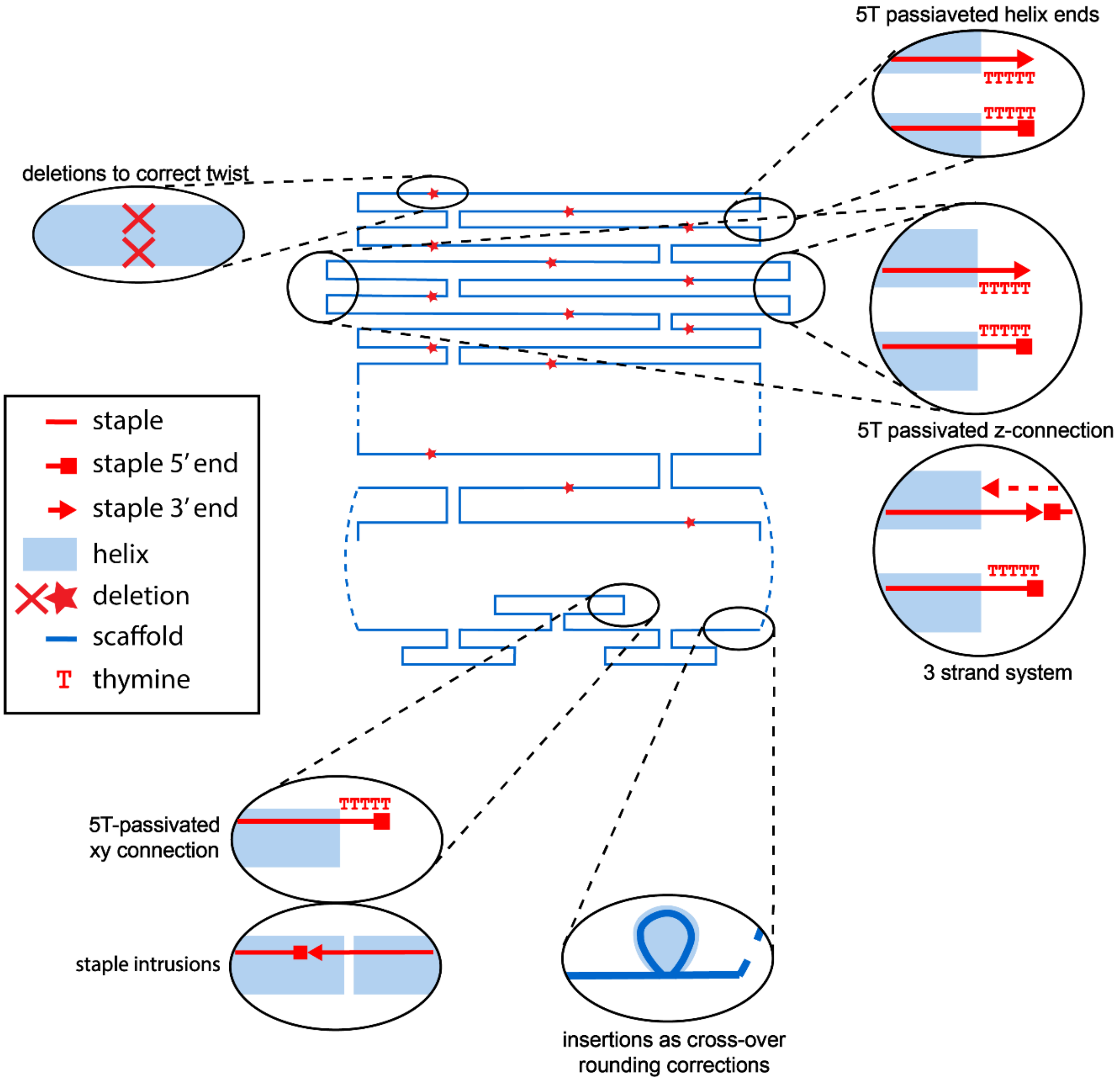


**Figure S4: Staple routing and positioning of corrections:** the residual twist of the honeycomb lattice was corrected with deletions every 126 bp and the crossover rounding errors were corrected with insertions at the connection site ends (see also Figure S6b), to keep the number of bp in the modular shell even. To prevent unwanted blunt-end interactions of the moDON, the helical ends were passivated with 5 Thymine (T) bases. The same strategy was used to passivate both connections sites of xy- and z-connections if desired. Connections in xy-direction were stabilized by staple intrusions, of elongated staples from one moDON to short staple omission in the complementary structure. Connections in z-direction were constructed with a three-strand-system. Here the 5’ ends on the moDONs left and the 3’ ends on the moDONs right were elongated by 10 nt or 11 nt, respectively, both constituting handles, complementary to each a half of a connector strand. Assigning 5’ elongations to the left and 3’ elongations to the right end of the moDON, achieved directionality of the z-connection. For staple sequences see Tables S2-S15.

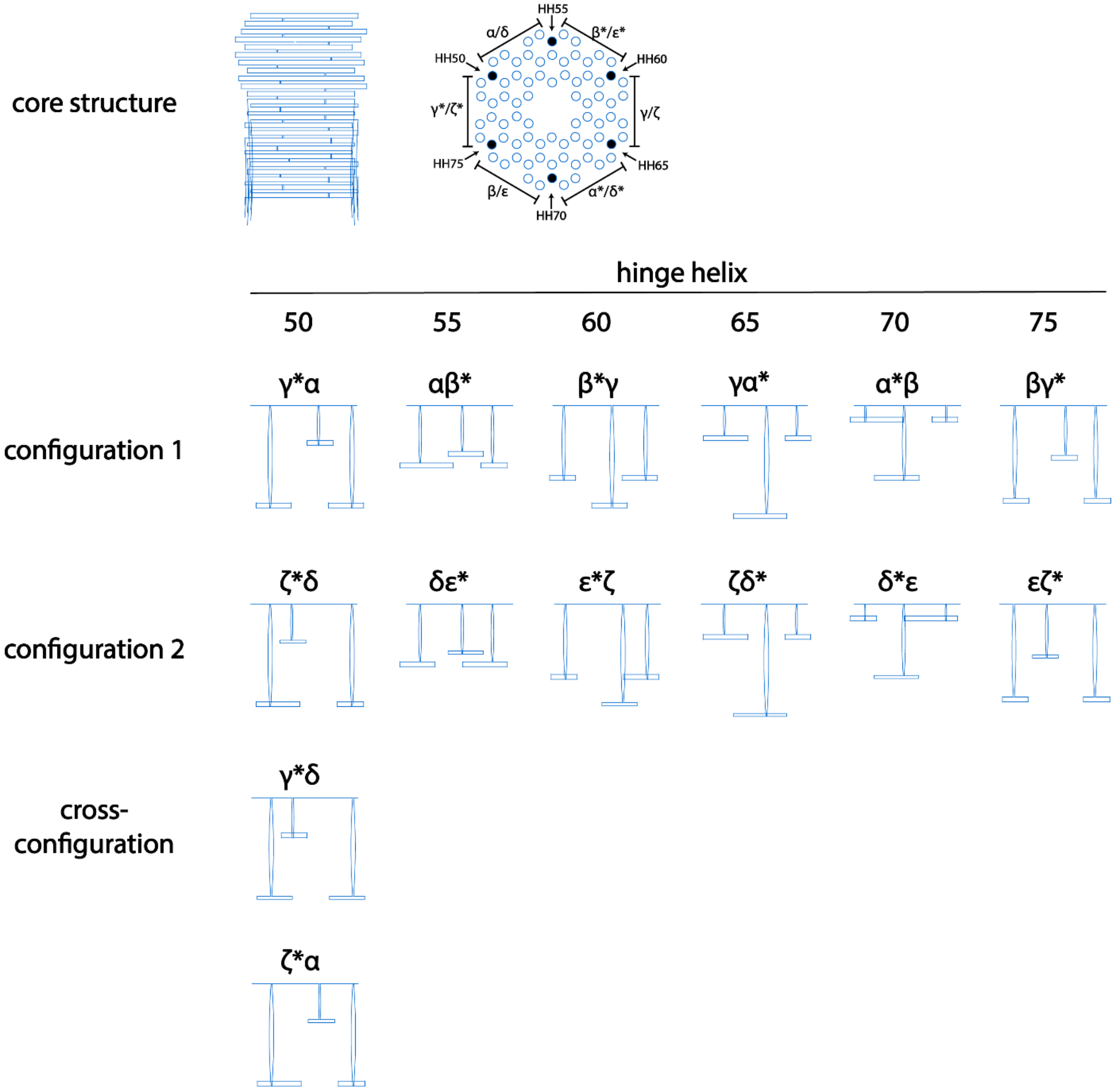


**Figure S5: caDNAno scaffold routing** paths of the different xy-connection sites. The core routing (top left image) always remains the same, while the shell is modular. All different configurations are arranged with respect to their configuration, and their hinge helix (HH). Note here, that HH50 has four possible configurations, while HH65 only has one. All other HH have two configurations. Also compare to Table S1. For staple sequences see Tables S2-S15.

**a**

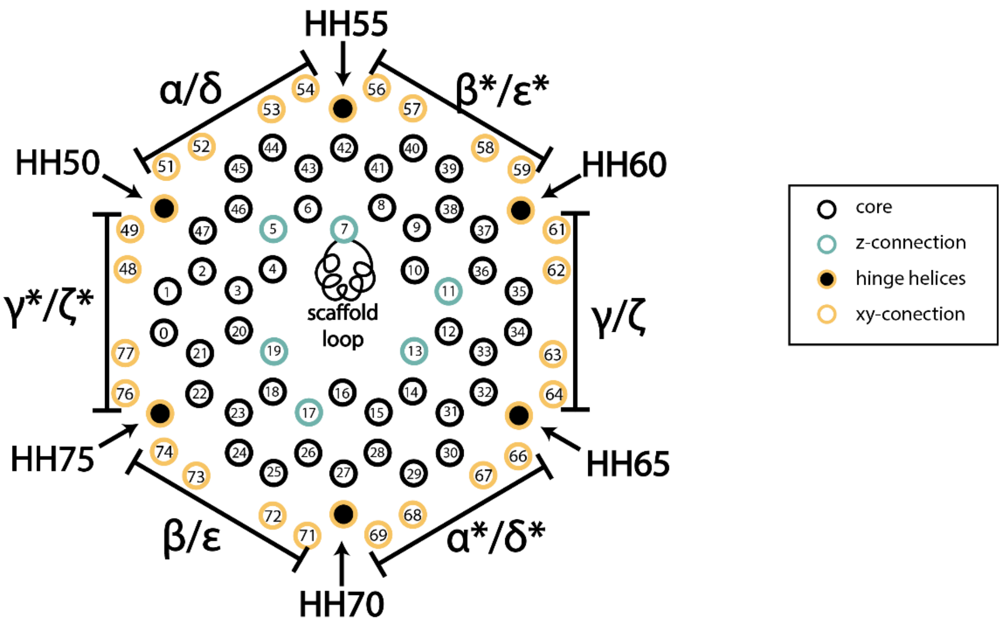


**b**

| hinge helix # | number of nt in scaffold loop αβγ | δεζ | γ*δ | ζ*α | final |
|---|---|---|---|---|---|
| 50 | 358 +2 | 356 +4 | 356 +4 | 358 +2 | 360 |
| 55 | 400 | 400 | 400 | 400 | 400 |
| 60 | 356 | 356 | 356 | 356 | 356 |
| 65 | 420 | 420 | 420 | 420 | 420 |
| 70 | 420 +2 | 422 | 422 | 422 | 422 |
| 75 | 314 | 314 | 314 | 314 | 314 |

**Figure S6: Helix numeration and scaffold loop corrections**: (**a**) Helix numeration of the moDON. Helices in yellow are part of the xy-connection sites, helices in turquois are z-connections sites, black helices are purely structural. Hinge helices are yellow with a black core. The scaffold loop was placed in the middle of helix 7, facing inwards into the moDON. (**b**) Since the literature value for one full helical turn in B-DNA configuration is not an integer, it is either rounded up or down in the caDNAno software to 11 or 10 bp. Changing the position and/or length of the modular parts from one configuration to the other leads to different amounts of nt in the scaffold of the modular parts. Those were corrected by small insertions. For staple sequences see Tables S2-S15.

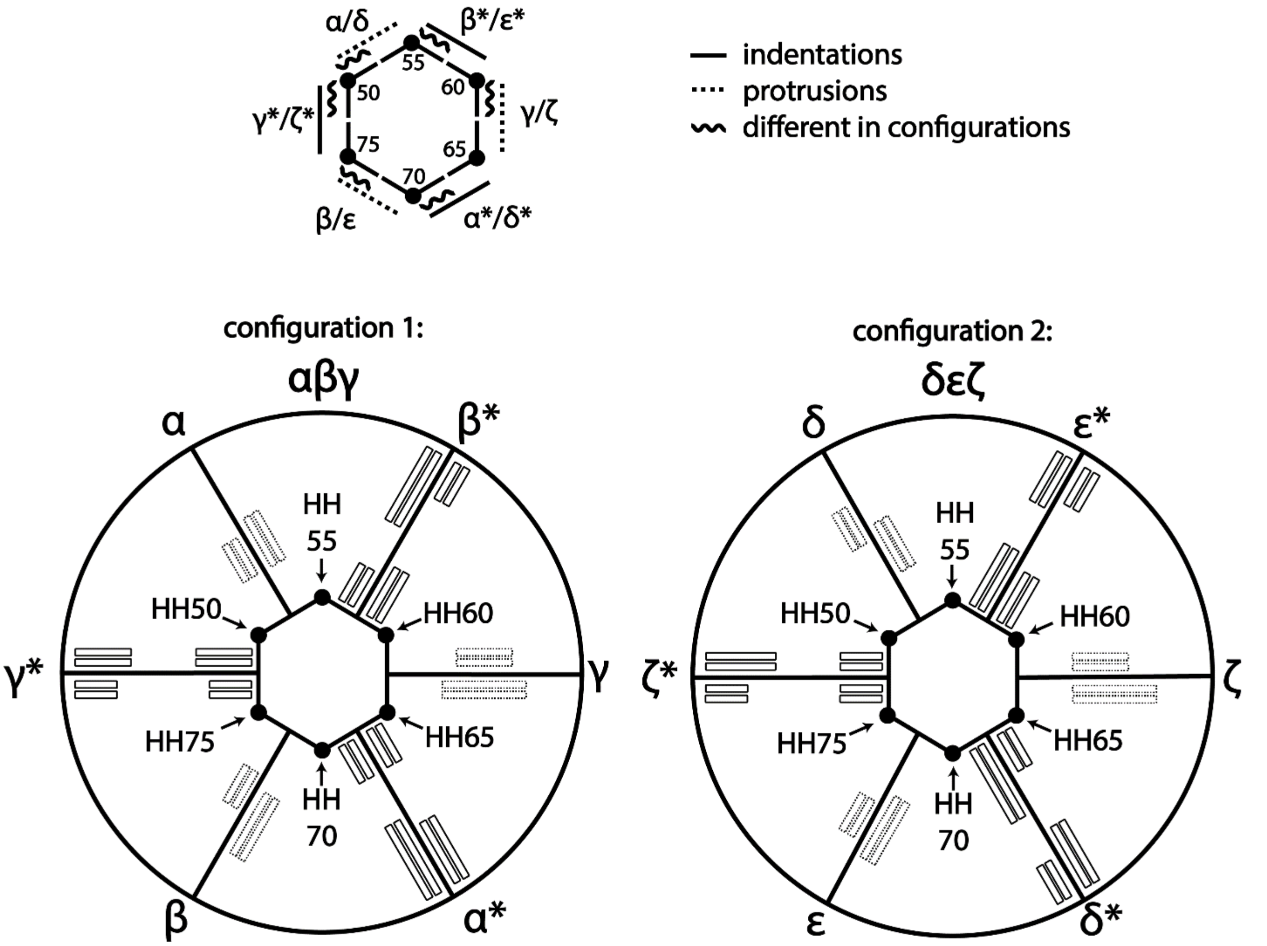


**Figure S7: Overview over connection sites and configurations**: All 12 connection sites are orthogonal to each other, across both configurations. Protrusions fit accurately into indentations, which are always denoted with the same letter and an asterisk. The position of connection sites cannot be changed in the moDON, but the connection site can be either from configuration 1 or configuration 2. Each connection site can be passivated. For staple sequences see Tables S2-S15.

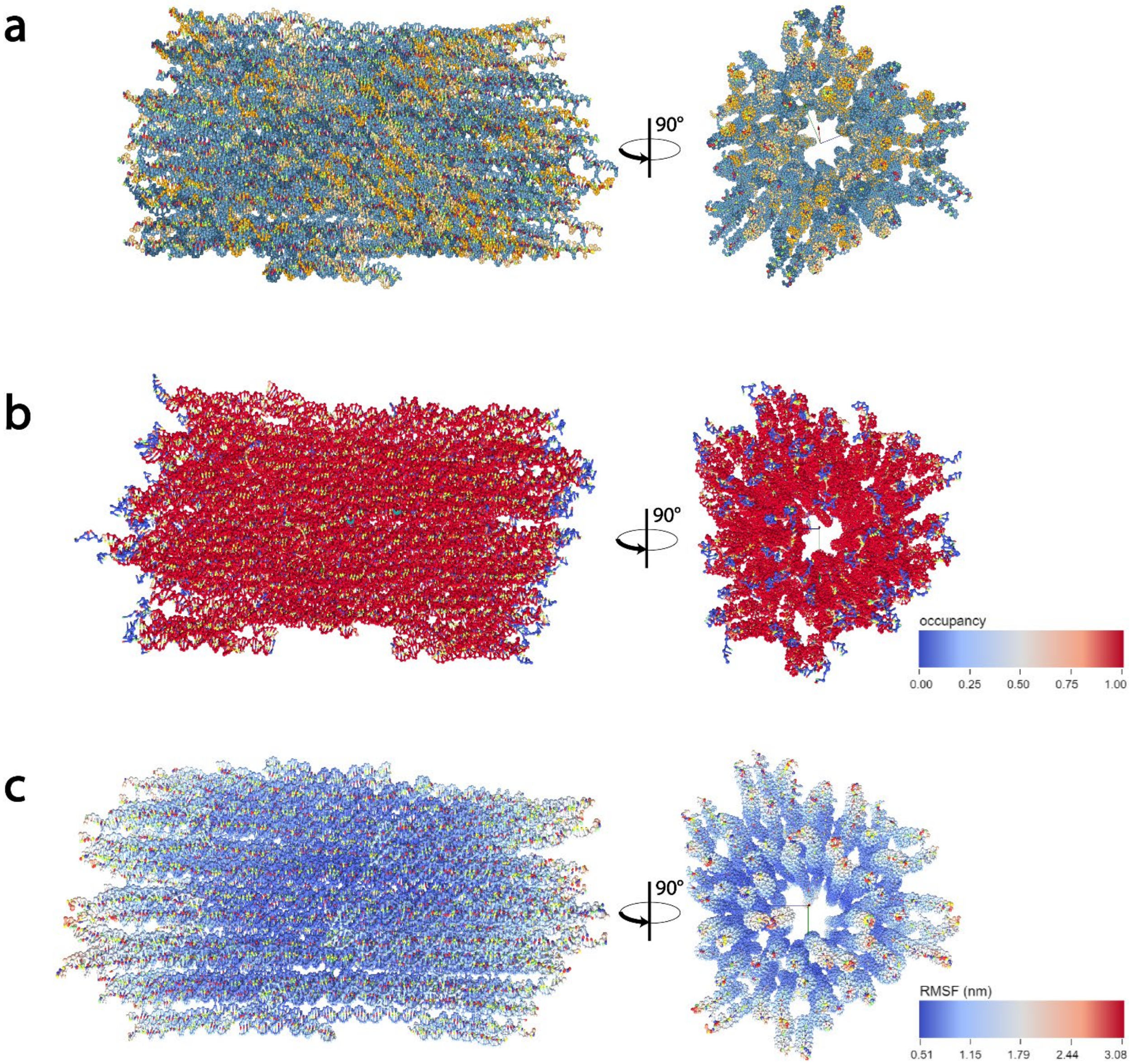


**Figure S8: oxDNA simulation of the moDON in configuration 1 (αβγ):** Side view to the left and top view on the right of (**a**) the averaged structure (**b**) the bond occupancy, indicating a high occupancy of the whole structure, except the 5T passivated helix ends (**c**) root mean squared fluctuation (RMSF) on the average structure, showing low flexibility across the sides, and marginally more towards the helix ends. Higher fluctuation is only seen for the 5T ssDNA passivation, explained by the lower persistence length of ssDNA compared to dsDNA. This oxDNA simulation, compared to the oxDNA simulation of the moDON in configuration 2 (Figure S9) suggests overall structural stability, even with changed shell structure.. The relaxation was performed with 5 000 CPU, and 1 000 000 GPU iterations, the simulation was performed with 100 000 000 iterations, all at 20°C, 1 M NaCl.

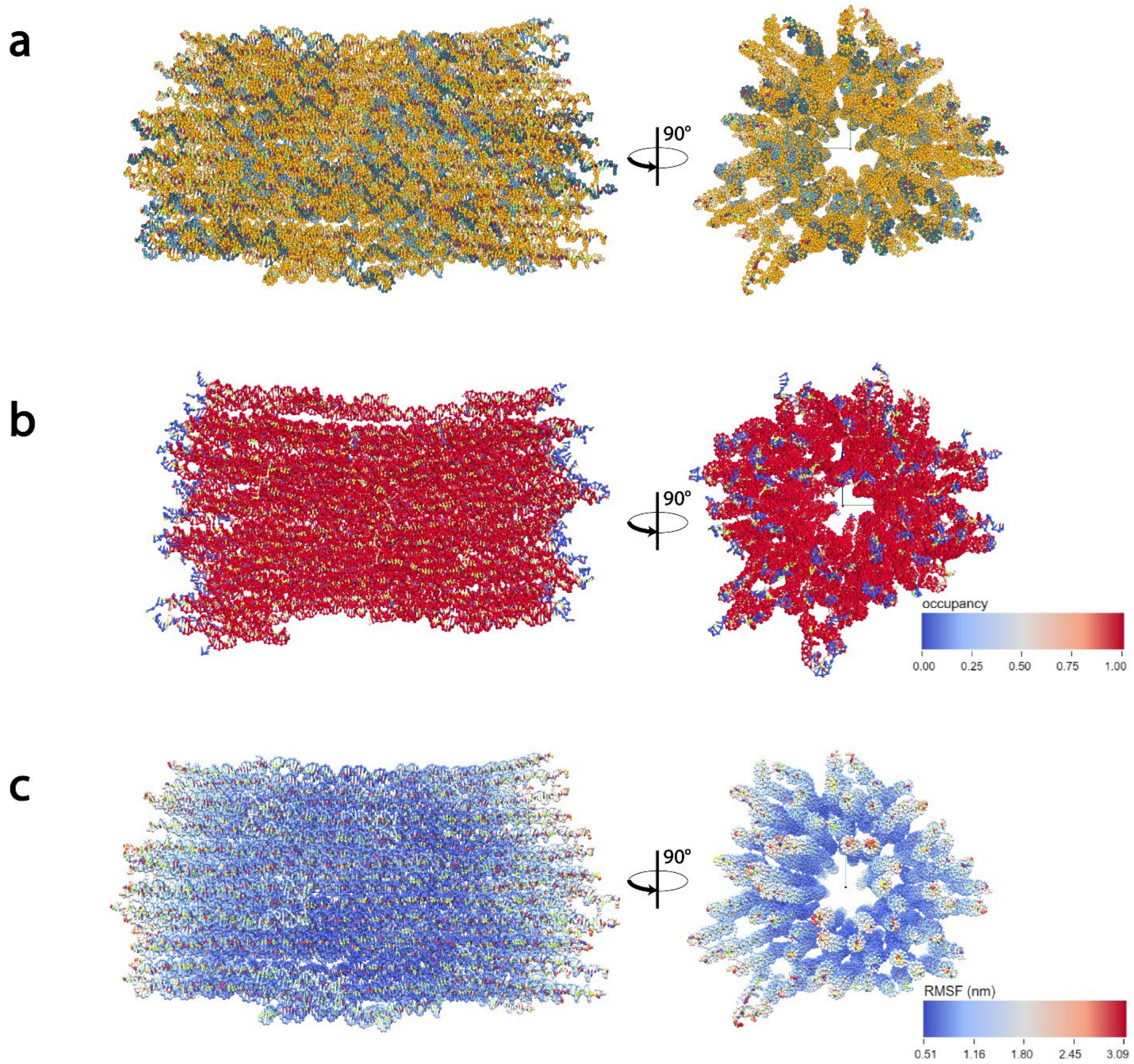


**Figure S9: oxDNA simulation of the moDON in configuration 2 (δεζ):** Side view to the left and top view on the right of (**a**) the averaged structure (**b**) the bond occupancy, indicating a high occupancy of the whole structure, except the 5T passivated helix ends (**c**) root mean squared fluctuation (RMSF) on the average structure, showing low flexibility across the sides, and marginally more towards the helix ends. Higher fluctuation is only seen for the 5T ssDNA passivation, explained by the lower persistence length of ssDNA compared to dsDNA. This oxDNA simulation, compared to the oxDNA simulation of the moDON in configuration 1 (Figure S8) suggests overall structural stability, even with changed shell structure. The relaxation was performed with 5 000 CPU, and 1 000 000 GPU iterations, the simulation was performed with 100 000 000 iterations, all at 20°C, 1 M NaCl.

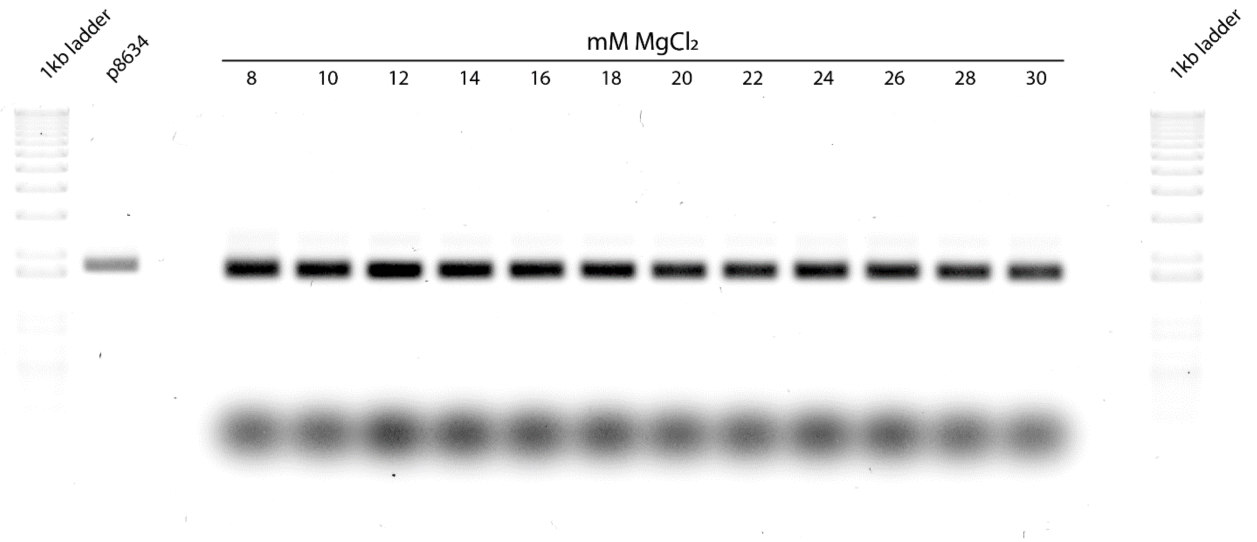


**Figure S10: AGE analysis of folded monomers with $MgCl_2$ titration** shows that the monomer fold with exceptional yield (97.5 %) over all tested amounts of salt. The monomer shows a slightly faster electrophoretic mobility than the scaffold p8634.

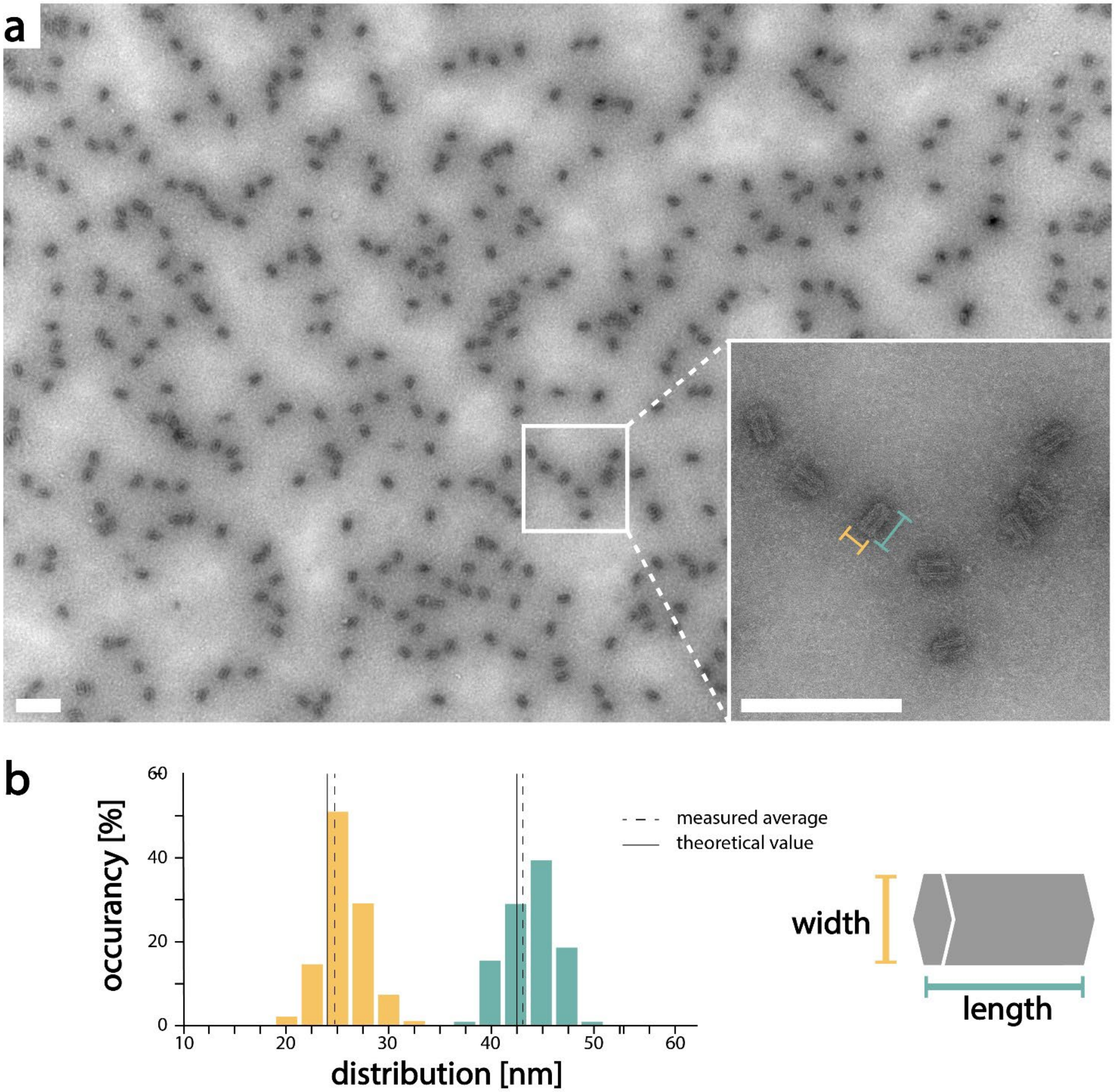


**Figure S11: Analysis of moDON monomers** (**a**) TEM micrographs of properly folded moDON monomers. (**b**) Size distribution of length (turquois) and width (yellow). Measured averages (length 42.98 nm, width 24.67 nm) is slightly larger than expected theoretical values (length 42.5 nm and width 24.0 nm) as calculated from number of bp in each helix of the core structure (125 bp) and number of helices at broadest point (12 helices). N > 100 individual monomers. Scale bars are 200 nm.

**a**

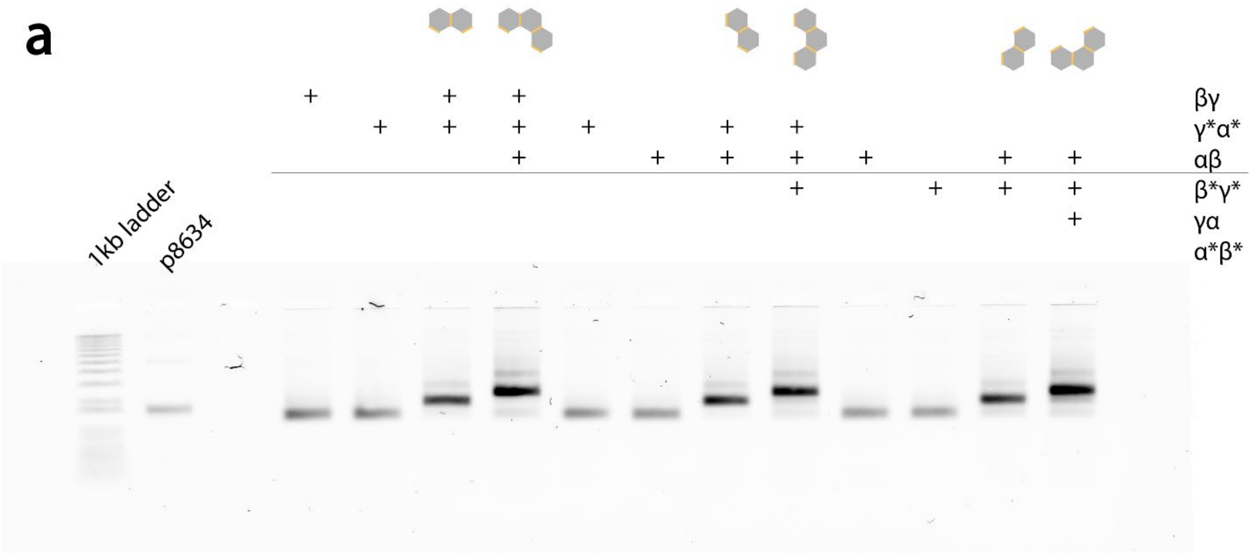


**b**

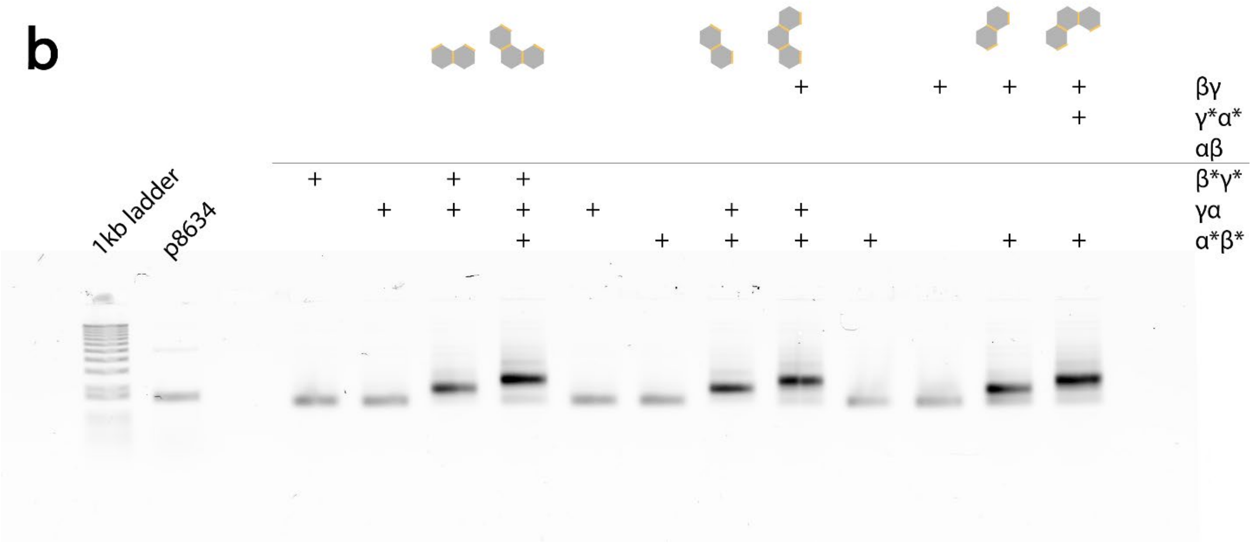


**Figure S12: AGE gel shift assay of dimer/trimer permutations 1:** moDONs in configuration 1. (**a**) and (**b**) show all permutations of dimers and trimers as constructed from the moDONs βγ, γ*α*, αβ, β*γ*, γα, and α*β*, as well as the respective monomers.

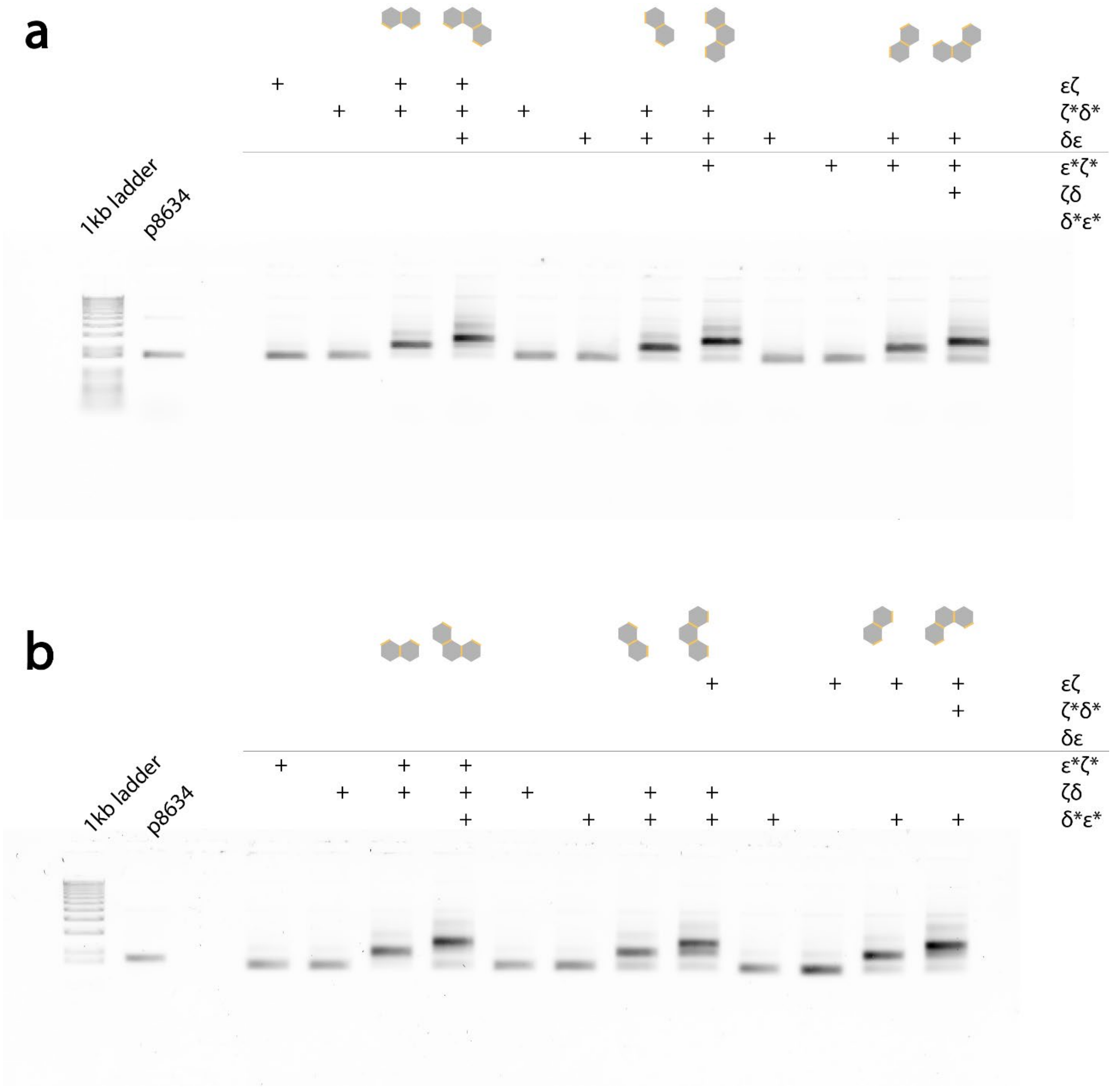


**Figure S13: AGE gel shift assay of dimer/trimer permutations 2:** moDONs in configuration 2. (**a**) and (**b**) show all permutations of dimers and trimers as constructed from the moDONs εζ, ζ*δ, δε, ε*ζ*, ζδ, and δ*ε*, as well as the respective monomers.

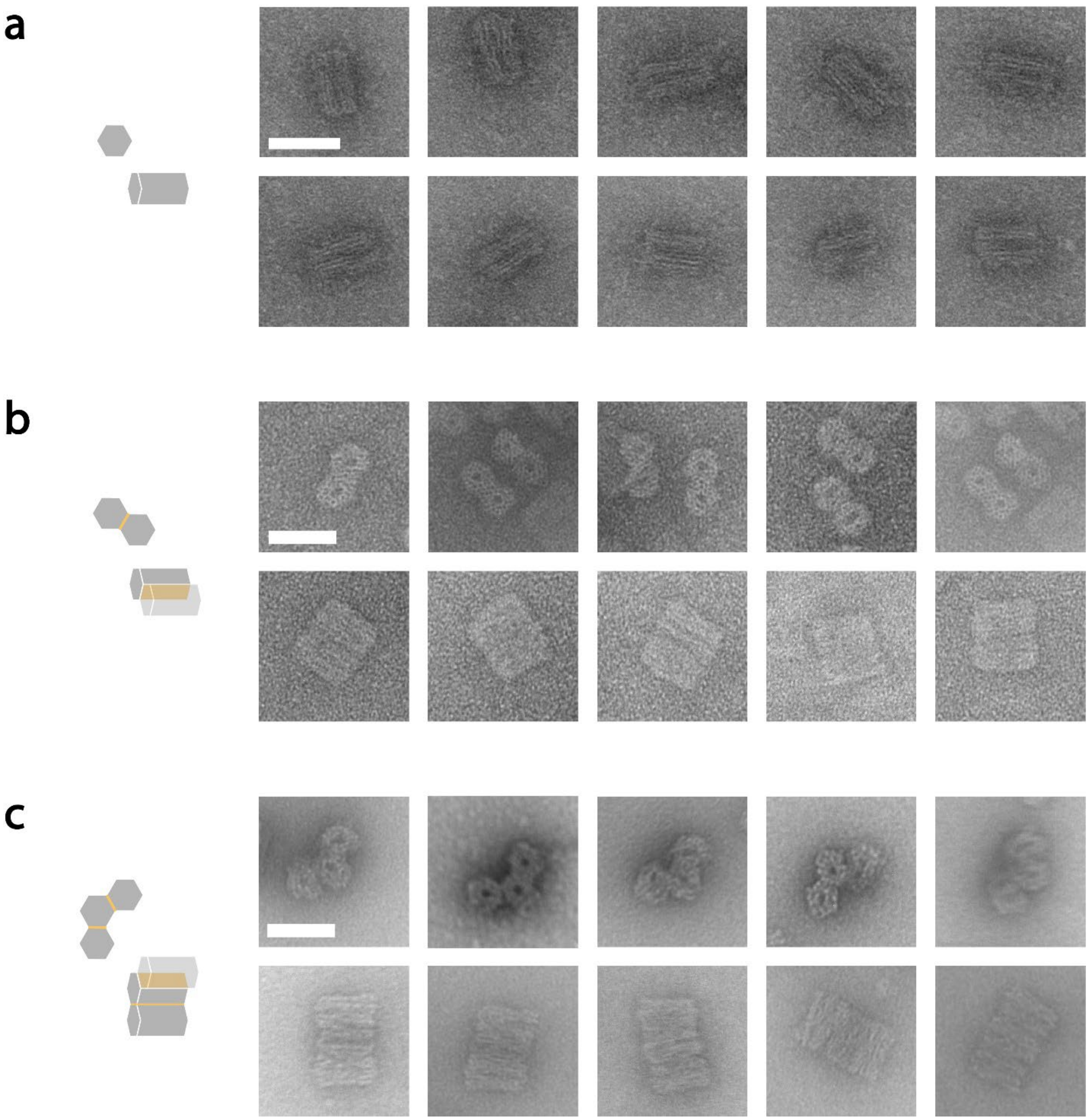


**Figure S14: close-up view on xy-structures 1:** Showing TEM micrographs of (**a**) monomers, (**b**) dimers, and (**c**) trimers. Data from the same experiment was also used in Figure 2d. Scale bars are 50 nm and hold for all micrographs of the respective structure.

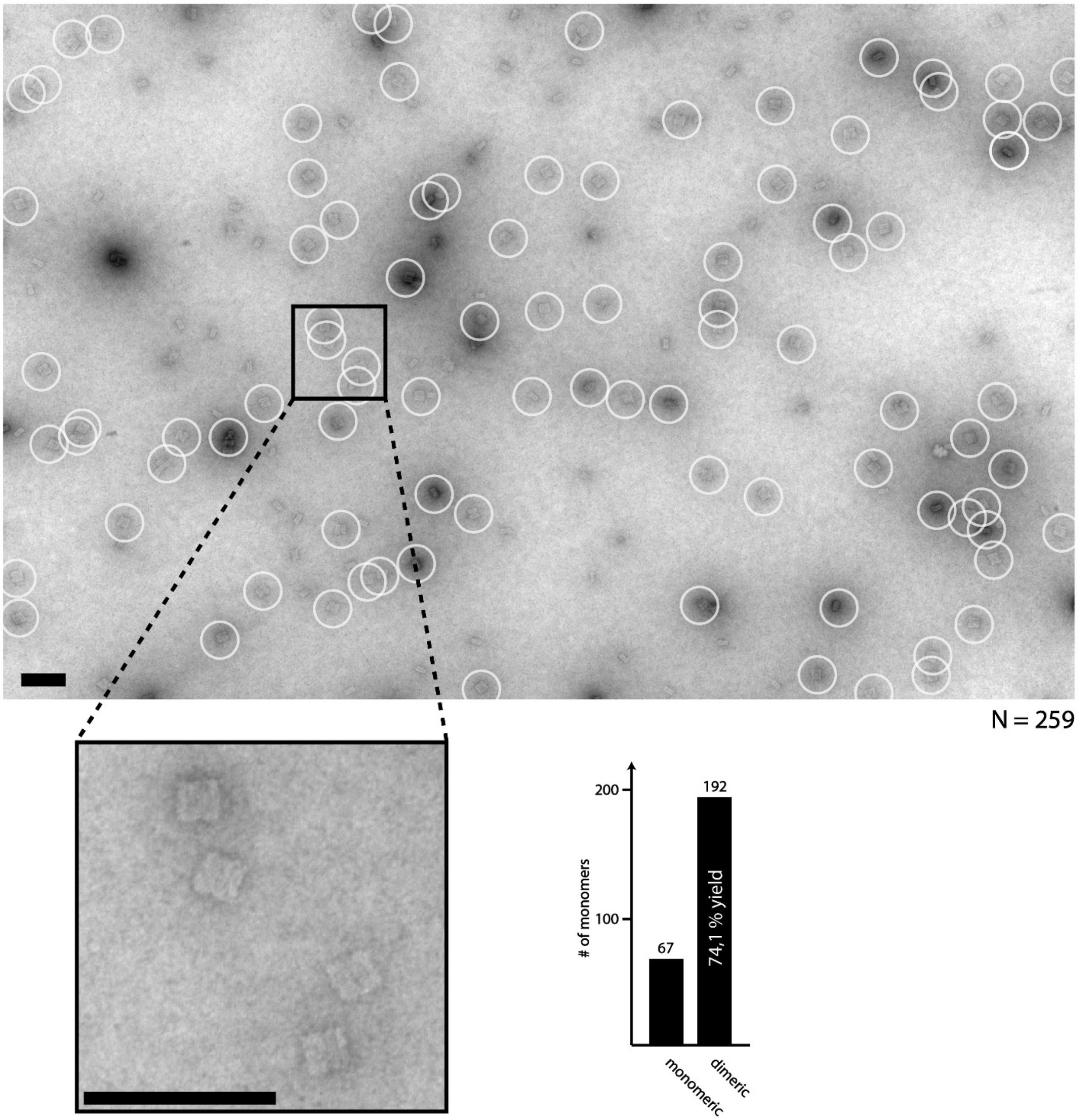


**Figure S15: Wide-field TEM micrograph and statistics of xy-dimer formation**. Analysis of the TEM micrograph shows a yield of 74.1 % of dimers, as calculated by the fraction of monomers in the desired superstructure ($N_{dimer}$ = 192) to the total amount of monomers ($N_{\Sigma}$ = 259). Scale bars are 200 nm.

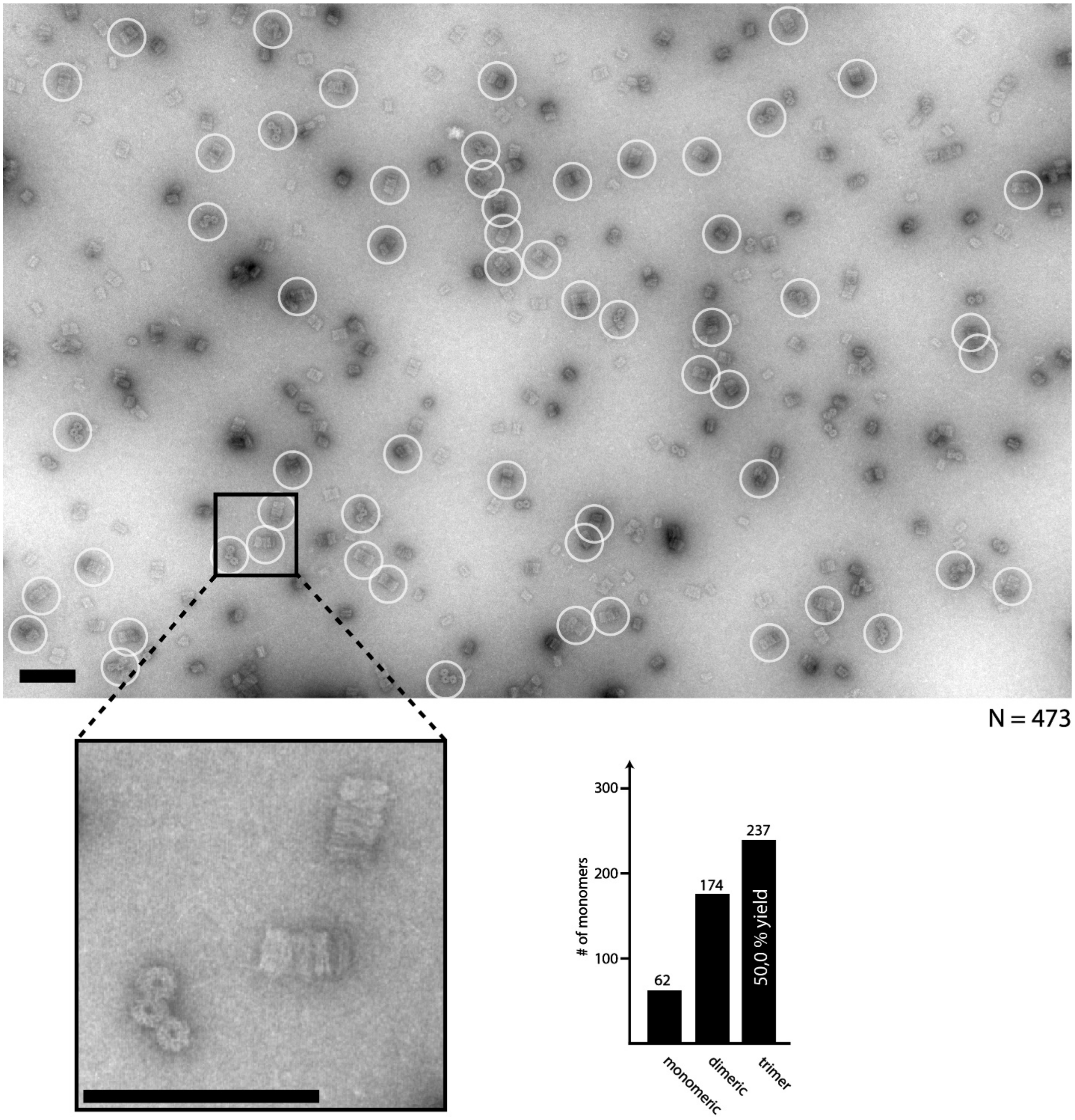


**Figure S16: Wide-field TEM micrograph and statistics of xy-trimer formation 1**. Analysis of the TEM micrograph shows a yield of 50.0 % of trimers, as calculated by the fraction of monomers in the desired superstructure ($N_{trimer}$ = 237) to the total amount of monomers ($N_{\Sigma}$ = 473). Scale bars are 200 nm.

a

b

c

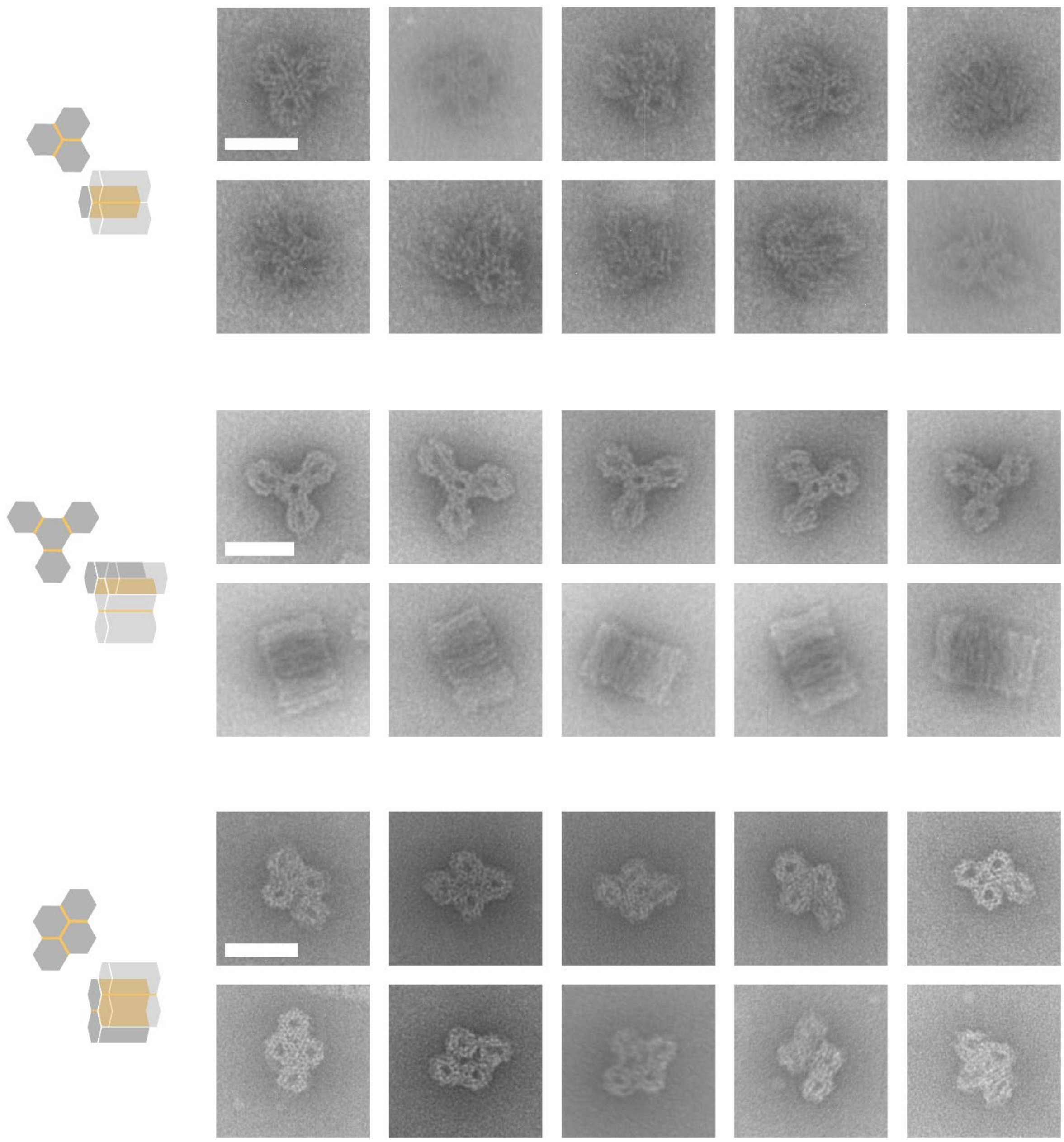

**Figure S17: close-up view on xy-structures 2**: Showing TEM micrographs of (**a**) trimers, (**b**), (**c**) different tetramers. Data from the same experiment was also used in Figure 2d. Scale bars are 50 nm and hold for all micrographs of the respective structure.

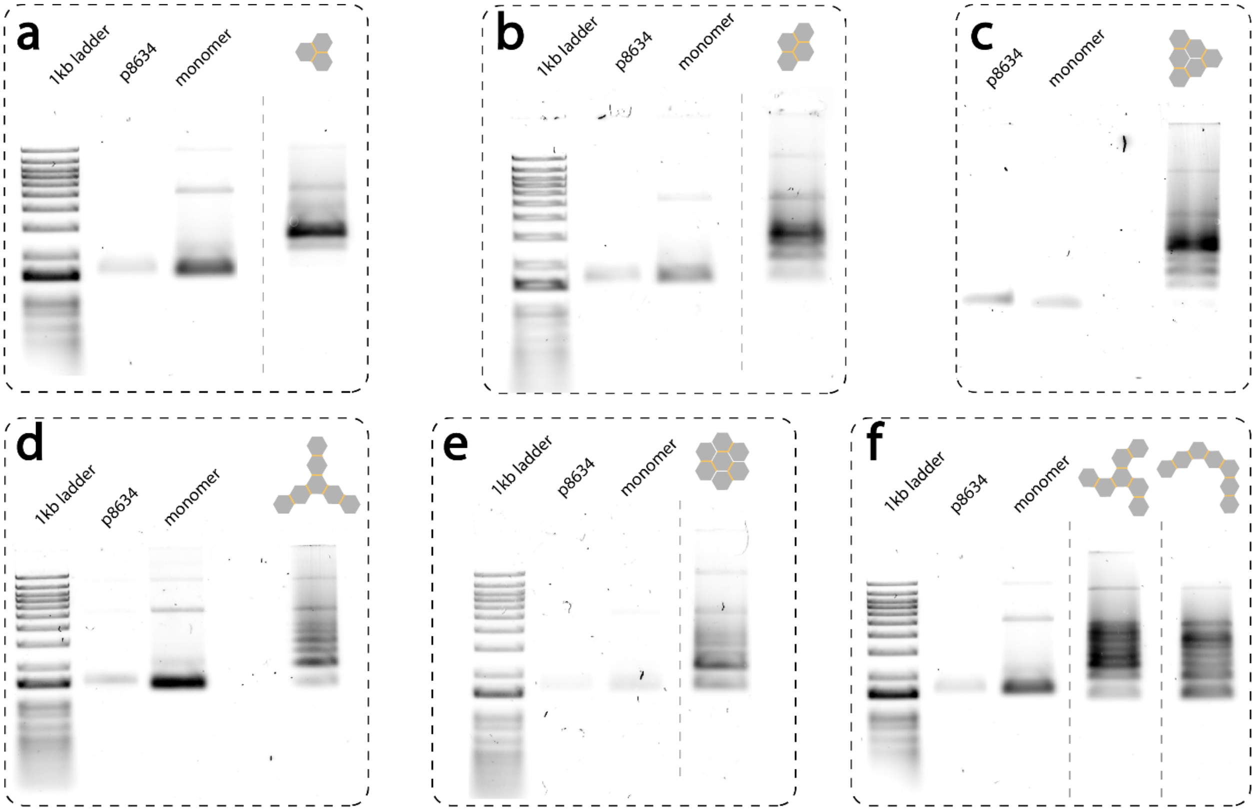


**Figure S18: AGE shift assay of xy-structures** made from various moDONs in a one-pot reaction. The trimer in (**a**) shows a yield of 87.60 %, the tetramer in (**b**) has a yield of 66.06 %, the hexamer in (**c**) shows a yield of 80.83 %. Yield of the heptamers in (**d**) – (**f**) was not measurable. Monomeric moDONs for the hexamer in (**c**) were added subsequently to circumvent the formation of equimolar parts of mutually exclusive tetramers or pentamers, not able to form hexamers.

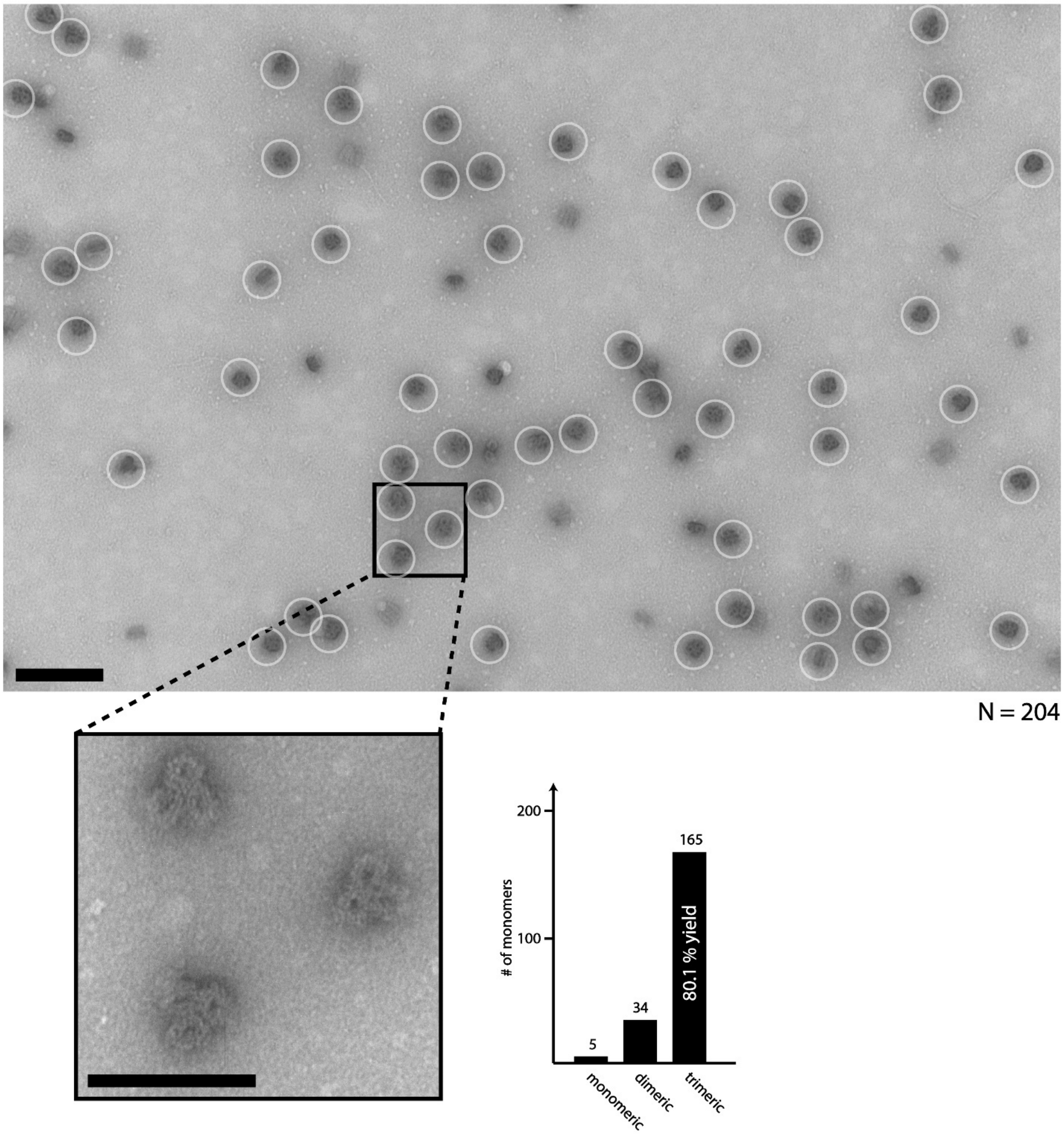


**Figure S19: Wide-field TEM micrograph and statistics of xy-trimer formation 2**. Analysis of the TEM micrograph shows a yield of 80.1 % of dimers, as calculated by the fraction of monomers in the desired superstructure ($N_{trimer}$ = 165) to the total amount of monomers ($N_{\Sigma}$ = 204). Scale bar of the wide-field image is 200 nm and of the zoom-in is 100 nm.

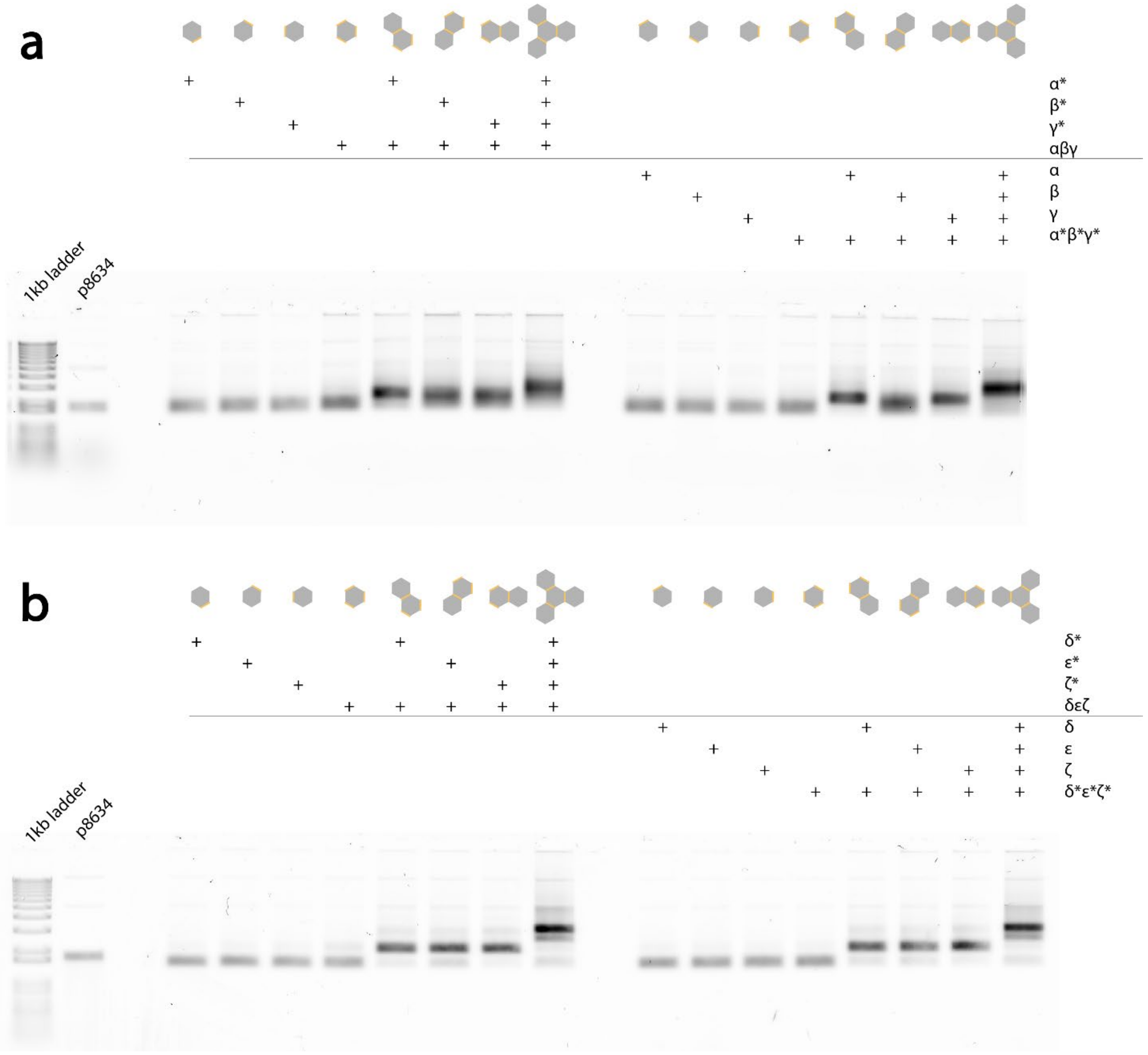


**Figure S20: AGE gel shift assay of tetramers** of moDONs in configurations 1 and 2. All monomers and dimers of the sub-structures, as well as the final tetramers are shown. (**a**) In configuration 1 α*, β*, γ*, and αβγ for the first tetramer and α, β, γ, and α*β*γ* for the second tetramer were used. (**b**) In configuration 2 δ*, ε*, ζ* and δεζ were used for the first tetramer and δ, ε, ζ, as well as δεζ were used for the second tetramer.

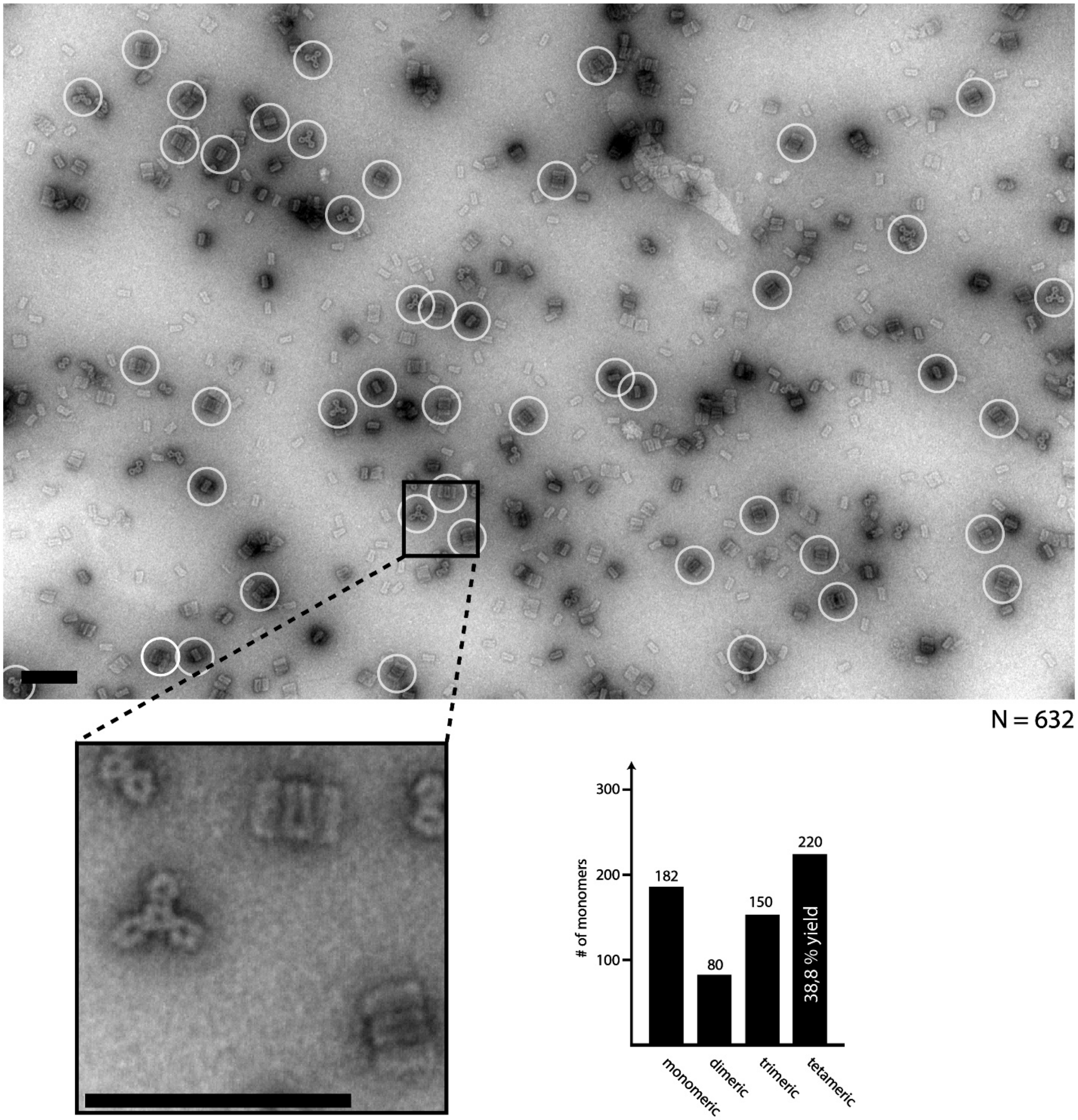


**Figure S21: Wide-field TEM micrograph and statistics of xy-tetramer formation 1**. Analysis of the TEM micrograph shows a yield of 38.8 % of tetramers, as calculated by the fraction of monomers in the desired superstructure ($N_{tetramer}$ = 220) to the total amount of monomers ($N_{\Sigma}$ = 632). Scale bars are 200 nm.

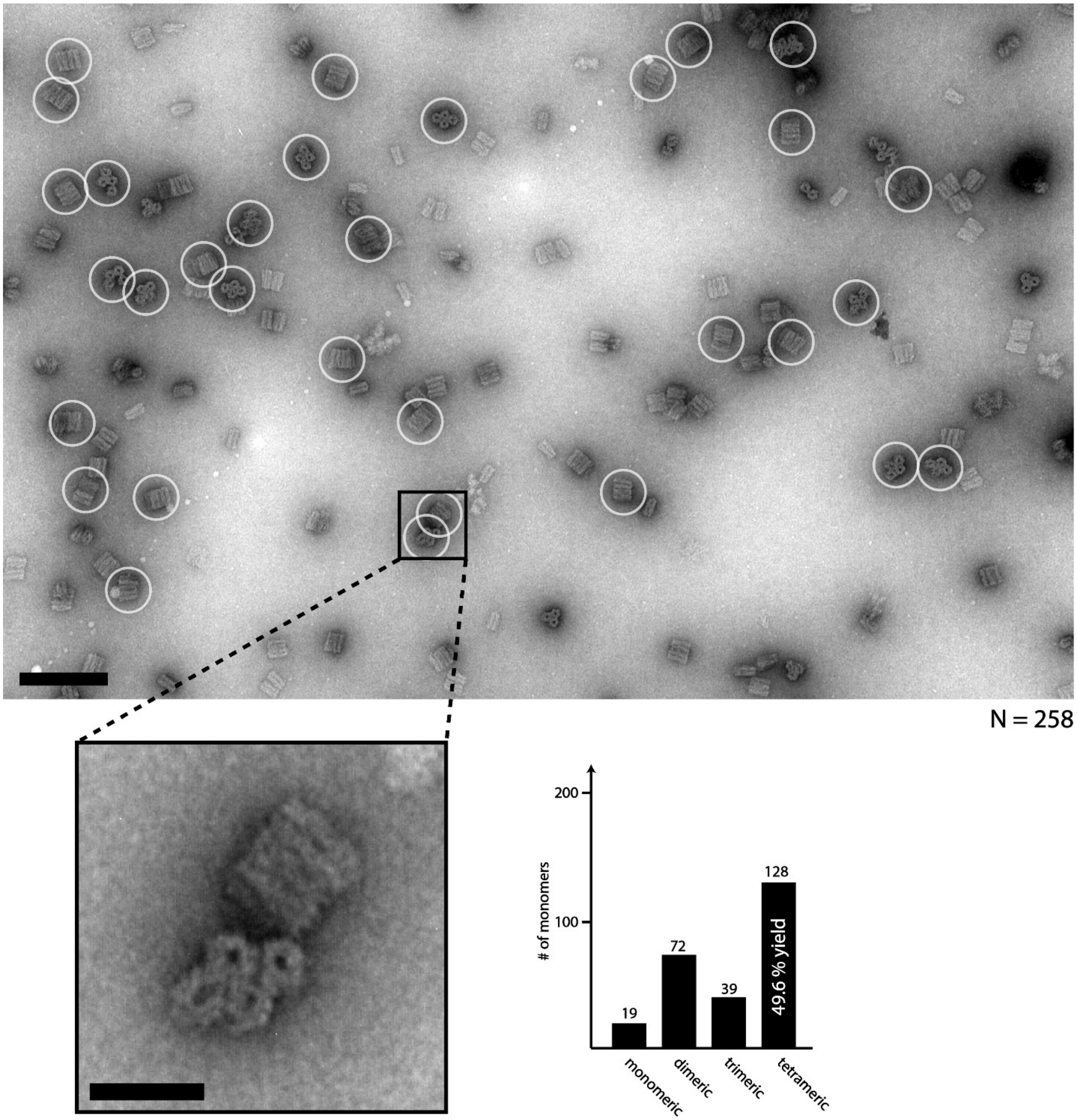


**Figure S22: Wide-field TEM micrograph and statistics of xy-trimer formation 2**. Analysis of the TEM micrograph shows a yield of 49.6 % of dimers, as calculated by the fraction of monomers in the desired superstructure ($N_{tetramer}$ = 128) to the total amount of monomers ($N_{\Sigma}$ = 258). Scale bar of the wide-field image is 200 nm and of the zoom-in is 50 nm.

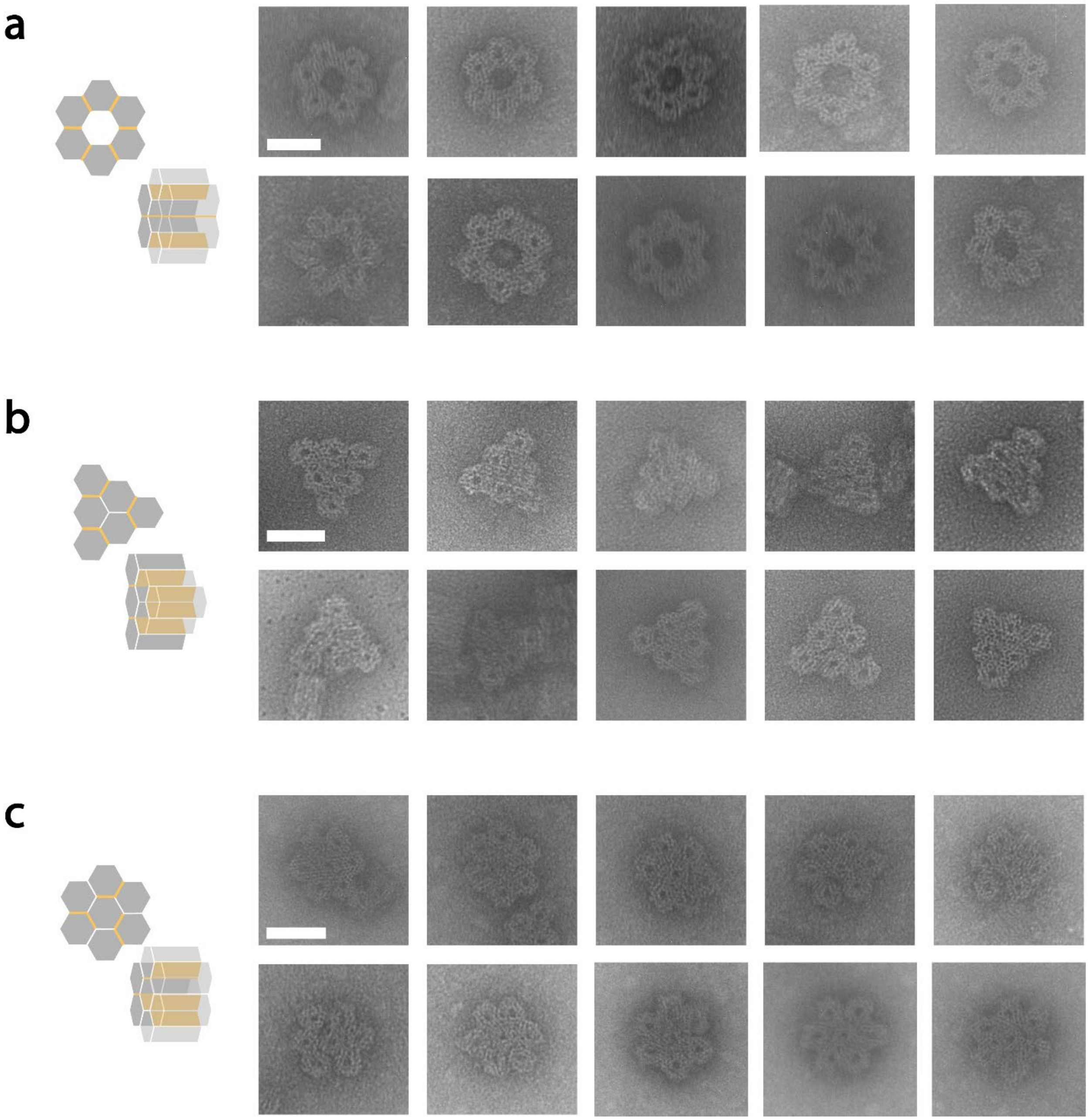


**Figure S23: close-up view on xy-structures 3:** Showing TEM micrographs of different (**a**), (**b**) hexamers, and (**c**) heptamers. Data from the same experiment was also used in Figure 2d. Scale bars are 50 nm and hold for all micrographs of the respective structure.

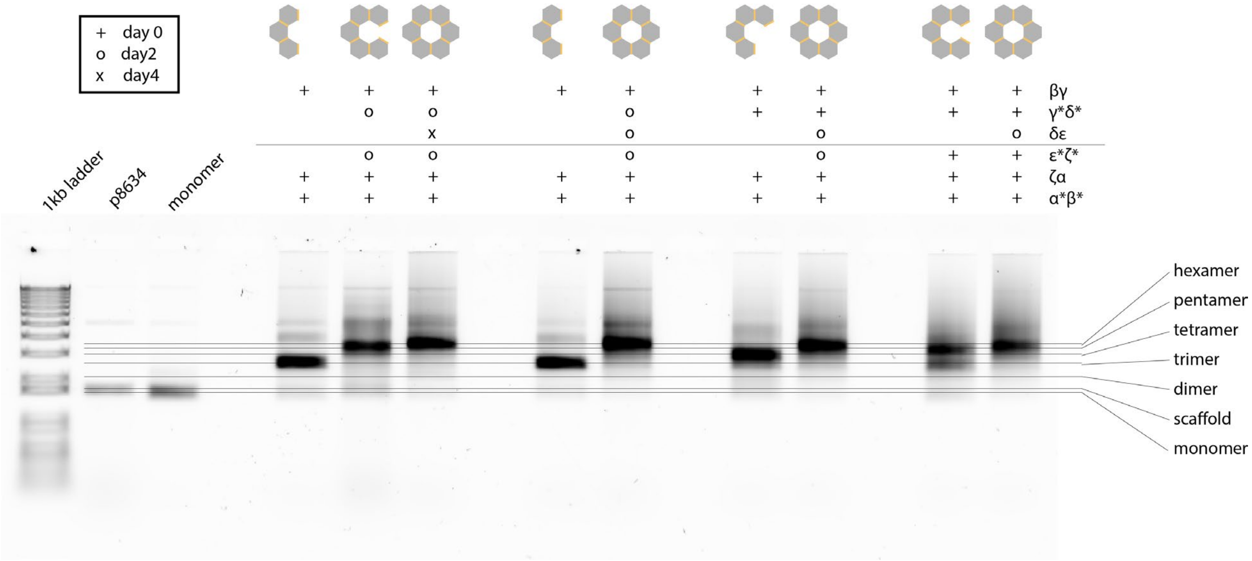


**Figure S24: AGE gel shift assay of hexamers** made from moDONs with connection sites from both configurations. Here, the moDONs α*β* and βγ only have connection sites from configuration 1; δε, and ε*ζ* only have connection sites from configuration 2. The connection sites of moDON monomers γ*δ* as well as ζα are a mixture from both configurations. moDONs were added subsequently to circumvent the formation of equimolar parts of mutually exclusive tetramers or pentamers, not able to form hexamers. Data from the same experiment was also used in Figure 2b.

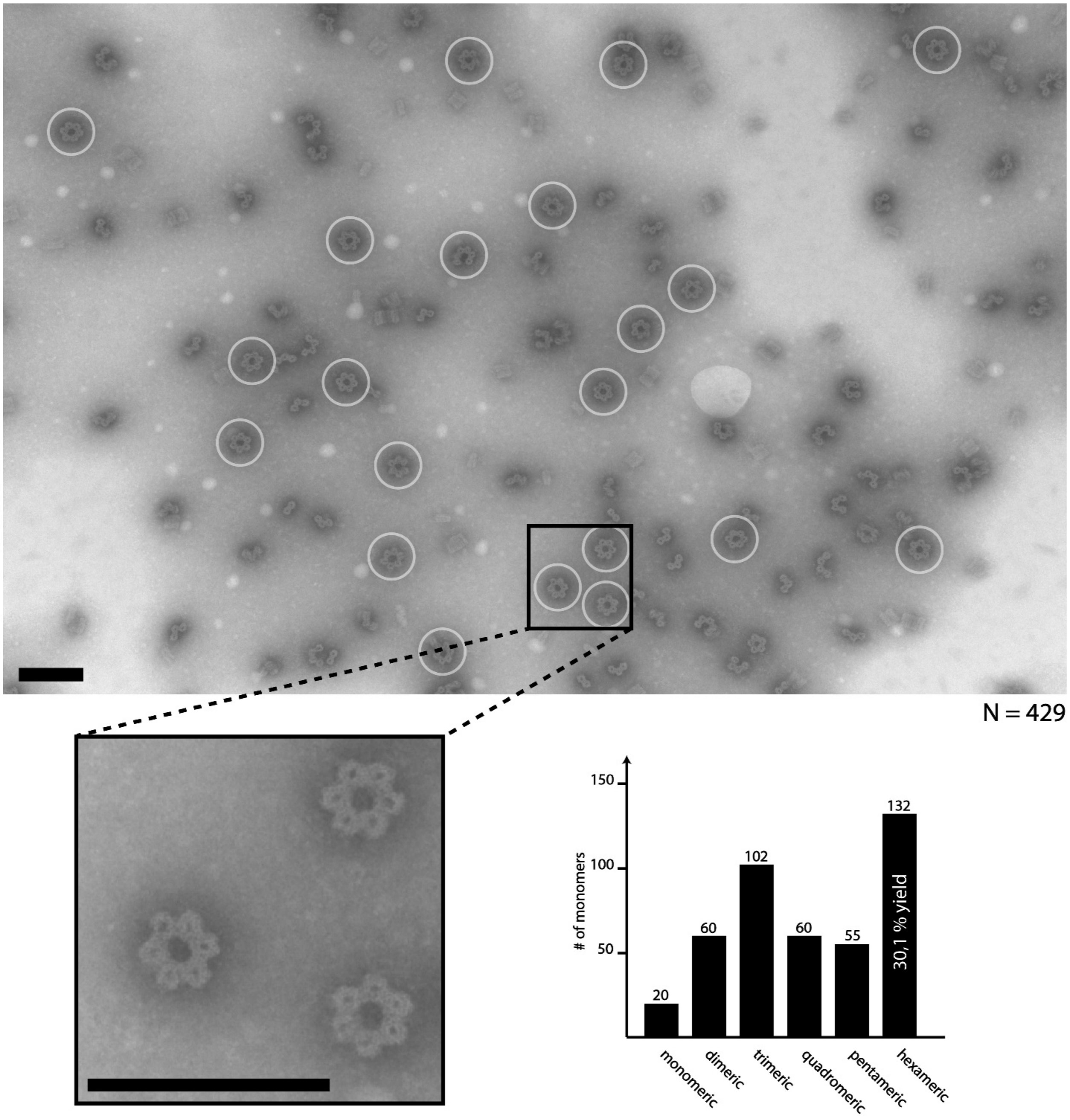


**Figure S25: Wide-field TEM micrograph and statistics of xy-hexamer formation 1**. Analysis of the TEM micrograph shows a yield of 30.1 % of dimers, as calculated by the fraction of monomers in the desired superstructure ($N_{hexamer}$ = 132) to the total amount of monomers ($N_{\Sigma}$ = 429). Scale bars are 200 nm.

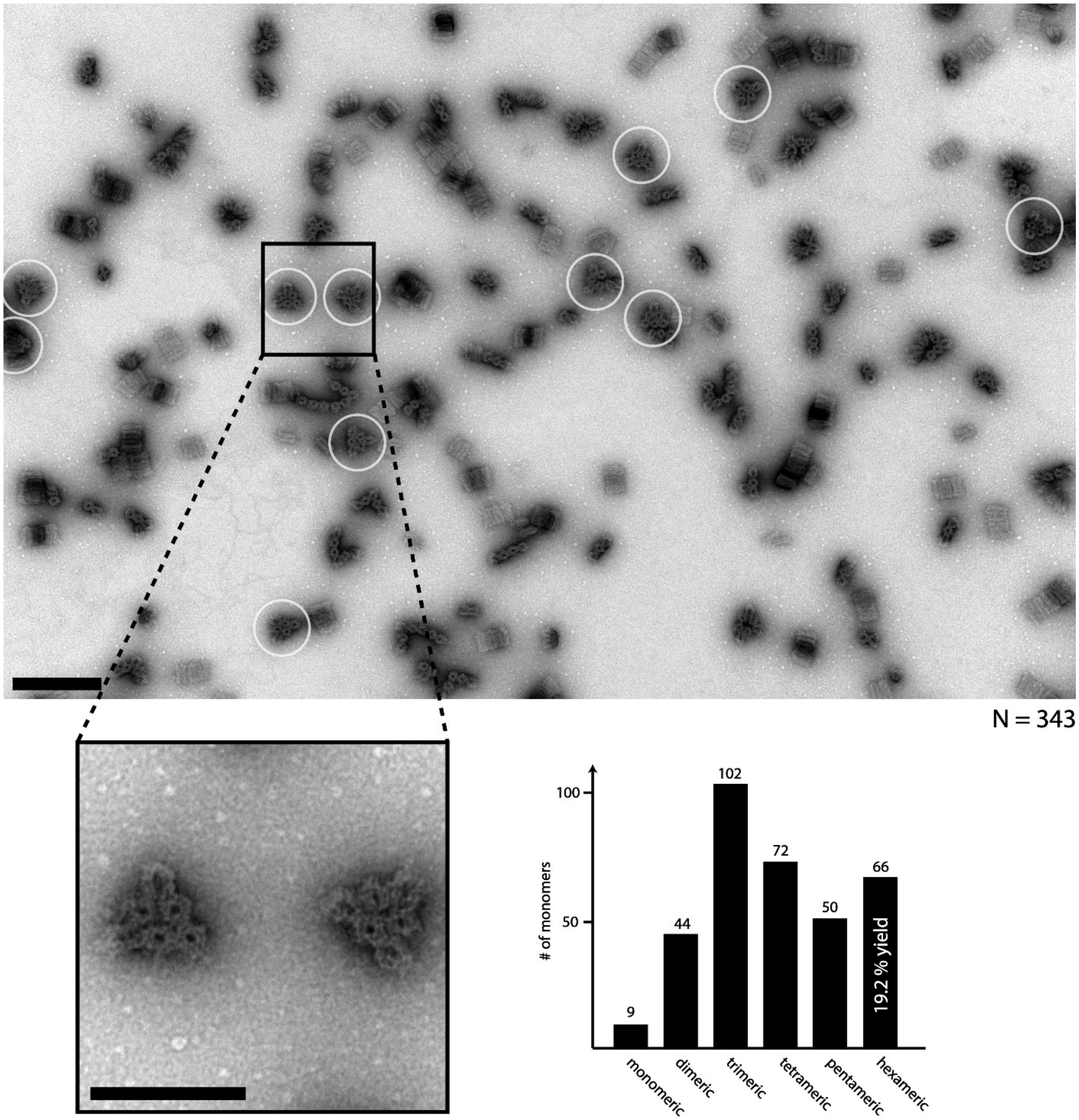


**Figure S26: Wide-field TEM micrograph and statistics of xy-hexamer formation 2**. Analysis of the TEM micrograph shows a yield of 19.2 % of dimers, as calculated by the fraction of monomers in the desired superstructure ($N_{hexamer}$ = 66) to the total amount of monomers ($N_{\Sigma}$ = 343). Scale bar of the wide-field image is 200 nm and of the zoom-in is 100 nm.

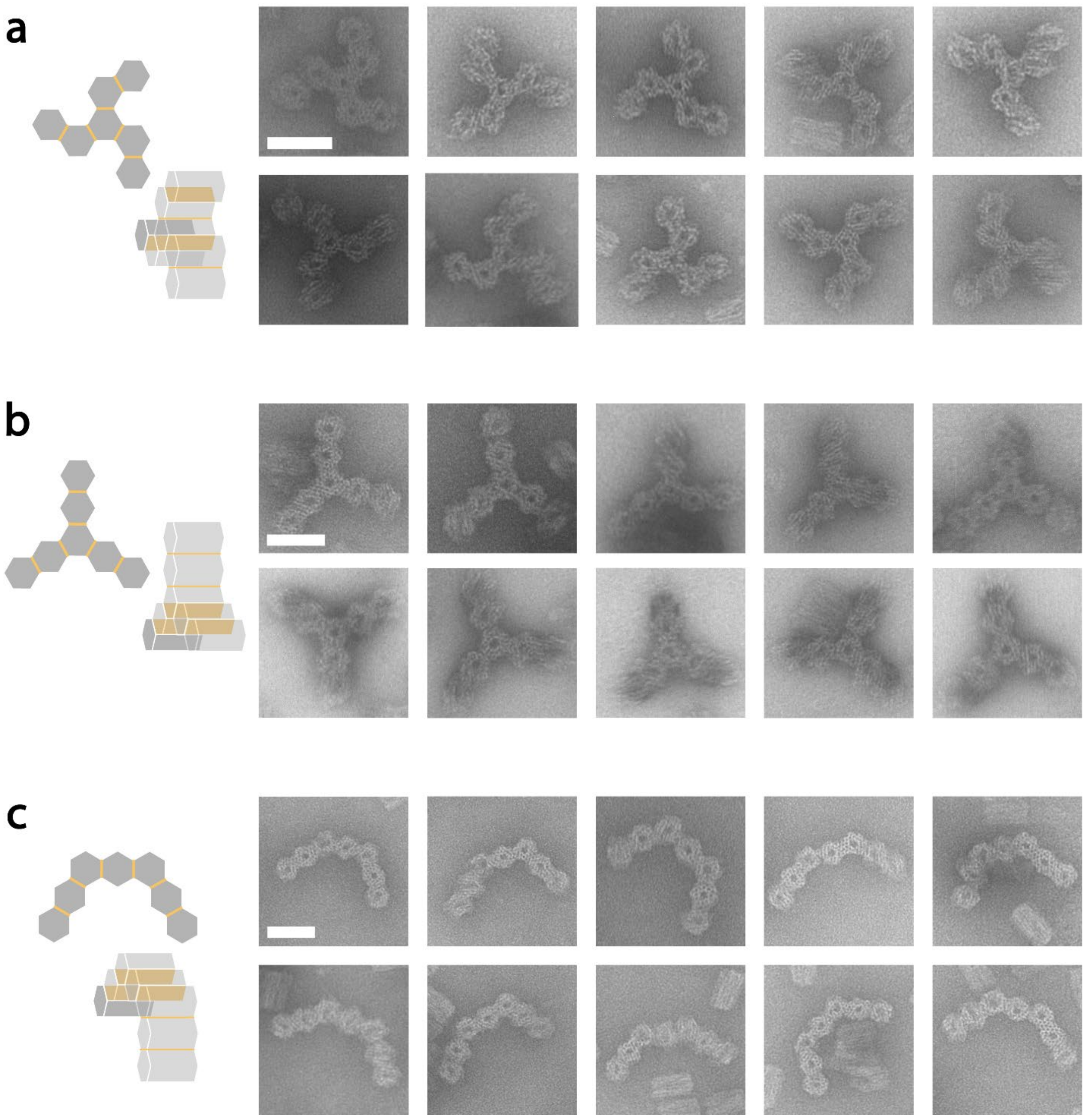


**Figure S27: close-up view on xy-structures 4** showing TEM micrographs of (**a**), (**b**), (**c**) different heptamers. Data from the same experiment was also used in Figure 2d. Scale bars are 50 nm and hold for all micrographs of the respective structure.

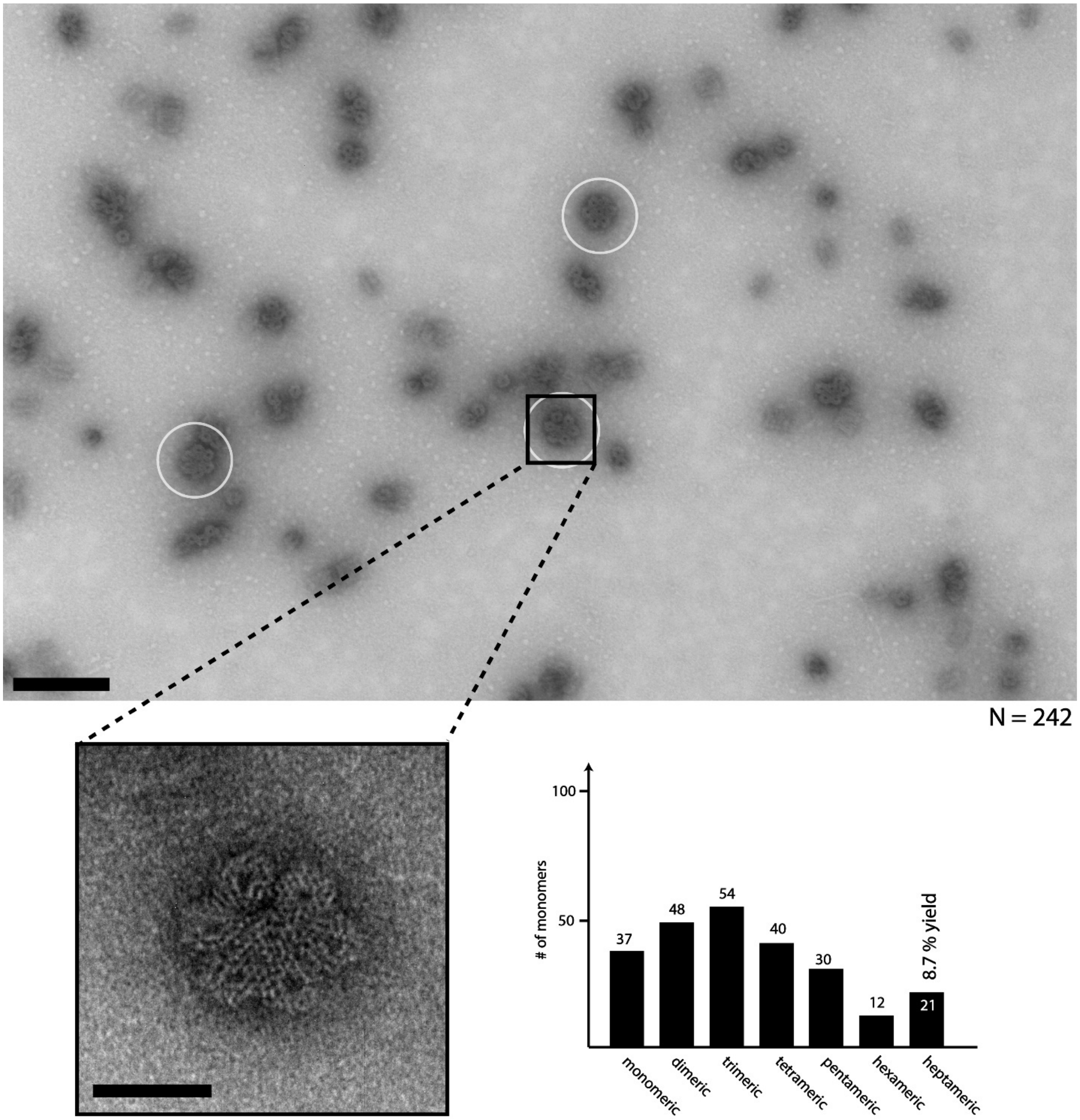


**Figure S28: Wide-field TEM micrograph and statistics of xy-heptamer formation 1**. Analysis of the TEM micrograph shows a yield of 8.7 % of dimers, as calculated by the fraction of monomers in the desired superstructure ($N_{heptamer}$ = 21) to the total amount of monomers ($N_{\Sigma}$ = 242). Scale bar of the wide-field image is 200 nm and of the zoom-in is 50 nm.

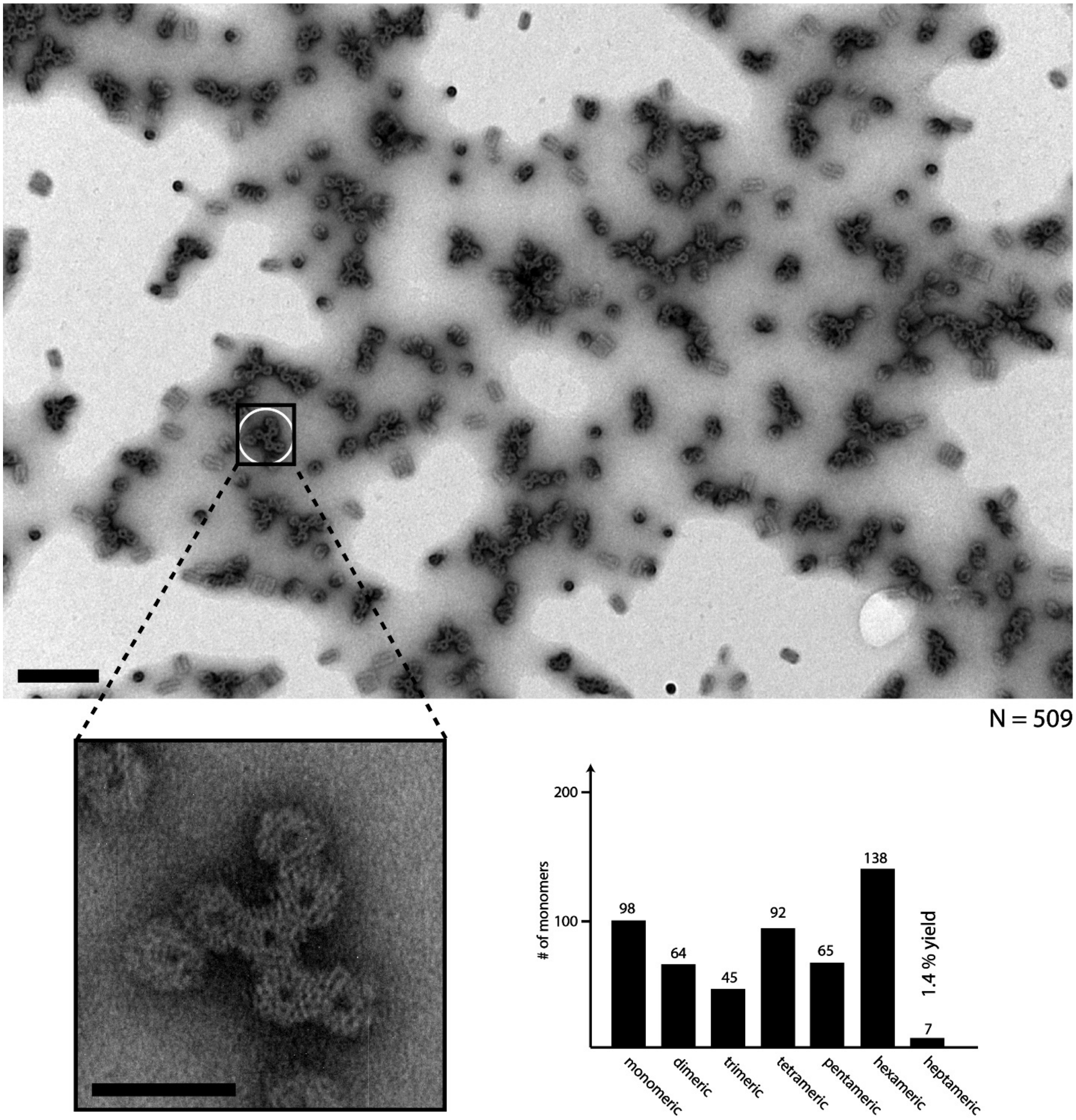


**Figure S29: Wide-field TEM micrograph and statistics of xy-heptamer formation 2**. Analysis of the TEM micrograph shows a yield of 1.4 % of dimers, as calculated by the fraction of monomers in the desired superstructure ($N_{heptamer}$ = 7) to the total amount of monomers ($N_{\Sigma}$ = 509). Scale bar of the wide-field image is 200 nm and of the zoom-in is 50 nm.

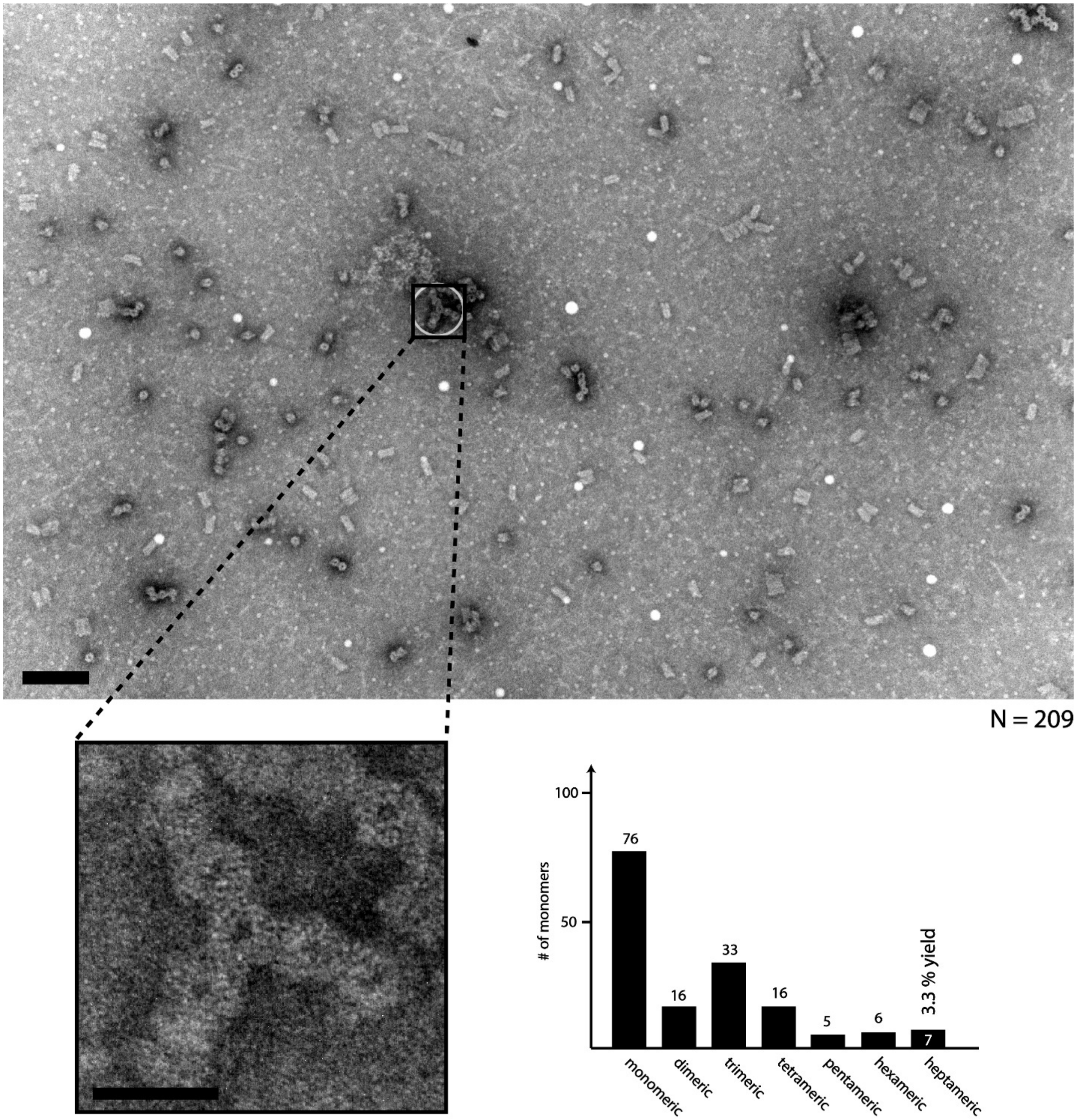


**Figure S30: Wide-field TEM micrograph and statistics of xy-heptamer formation 3**. Analysis of the TEM micrograph shows a yield of 3.3 % of dimers, as calculated by the fraction of monomers in the desired superstructure ($N_{heptamer}$ = 7) to the total amount of monomers ($N_{\Sigma}$ = 209). Scale bar of the wide-field image is 200 nm and of the zoom-in is 50 nm.

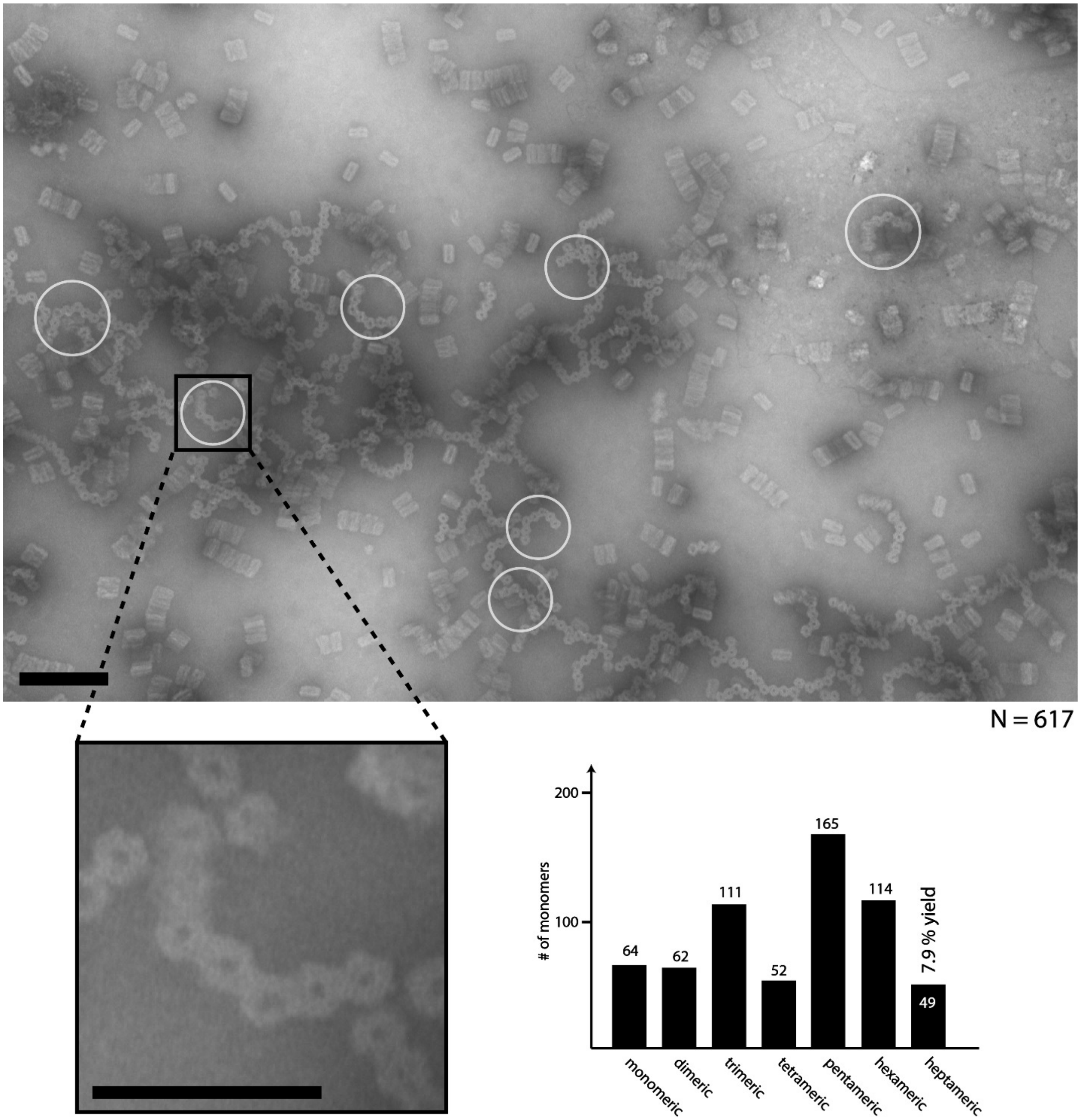


**Figure S31: Wide-field TEM micrograph and statistics of xy-heptamer formation 4**. Analysis of the TEM micrograph shows a yield of 7.9 % of dimers, as calculated by the fraction of monomers in the desired superstructure ($N_{heptamer}$ = 49) to the total amount of monomers ($N_{\Sigma}$ = 617). Scale bar of the wide-field image is 200 nm and of the zoom-in is 100 nm.

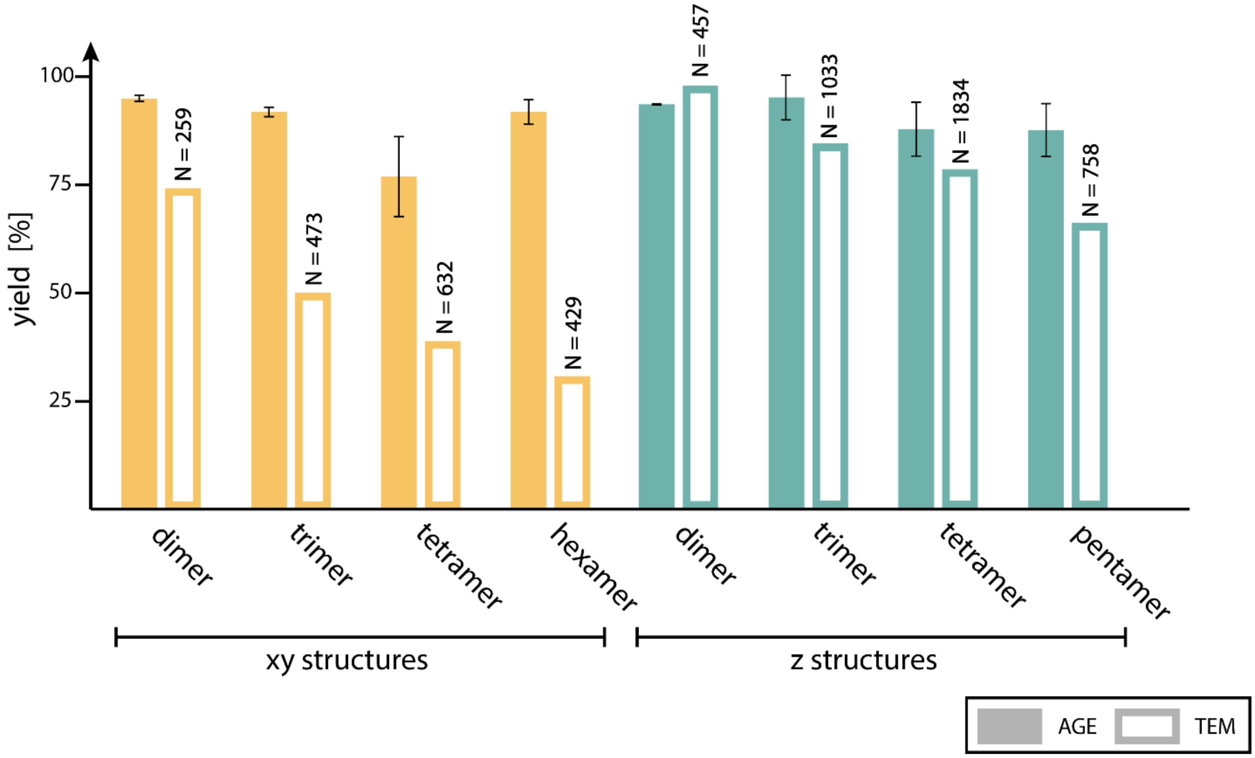


**Figure S32: Yield overview of AGE and TEM yields**: AGE yields were calculated as the mean of three independent experiments and are displayed with the respective standard deviation. xy-assemblies are shown in yellow, z-assmeblies in green. For xy-dimers: 94,8 ± 1.8 %, trimers: 91.7 ± 1.3 %, tetramers: 76.8 ± 9.4 %, and hexamers: 91.7 ± 3.0 %. For z-dimers: 93.2 ± 0.3 %, trimers: 94.8 ± 5.3 %, tetramers: 87.5 ± 6.4 %, and pentamers: 87.3 ± 6.3 %. Yields were extracted from gel images with the build-in gel analysis function in the image analysis software Fiji: Graphs of summed intensity were plotted and yield extracted as fraction of surface area. Peaks from gel impurities and/or remnants of scaffold impurities were disregarded. TEM yields were calculated as fraction of monomers in the desired superstructure from the total number N of moDONs (always N>250) in wide-field TEM micrographs. For xy-dimers 74.1 % (N=259), trimers: 50.0 % (N=473), tetramers: 38.8 % (N=632), and hexamers: 30.1 % (N=429). For z-dimers: 97.7 % (N=457), for trimers: 84.2 % (N=1033), for tetramers: 78.3 % (N=1834), and for pentamers 65.9 % (N=753). Detailed analysis and wide-field micrographs can be found in Figures S16-S19, S30, and S35-S37.

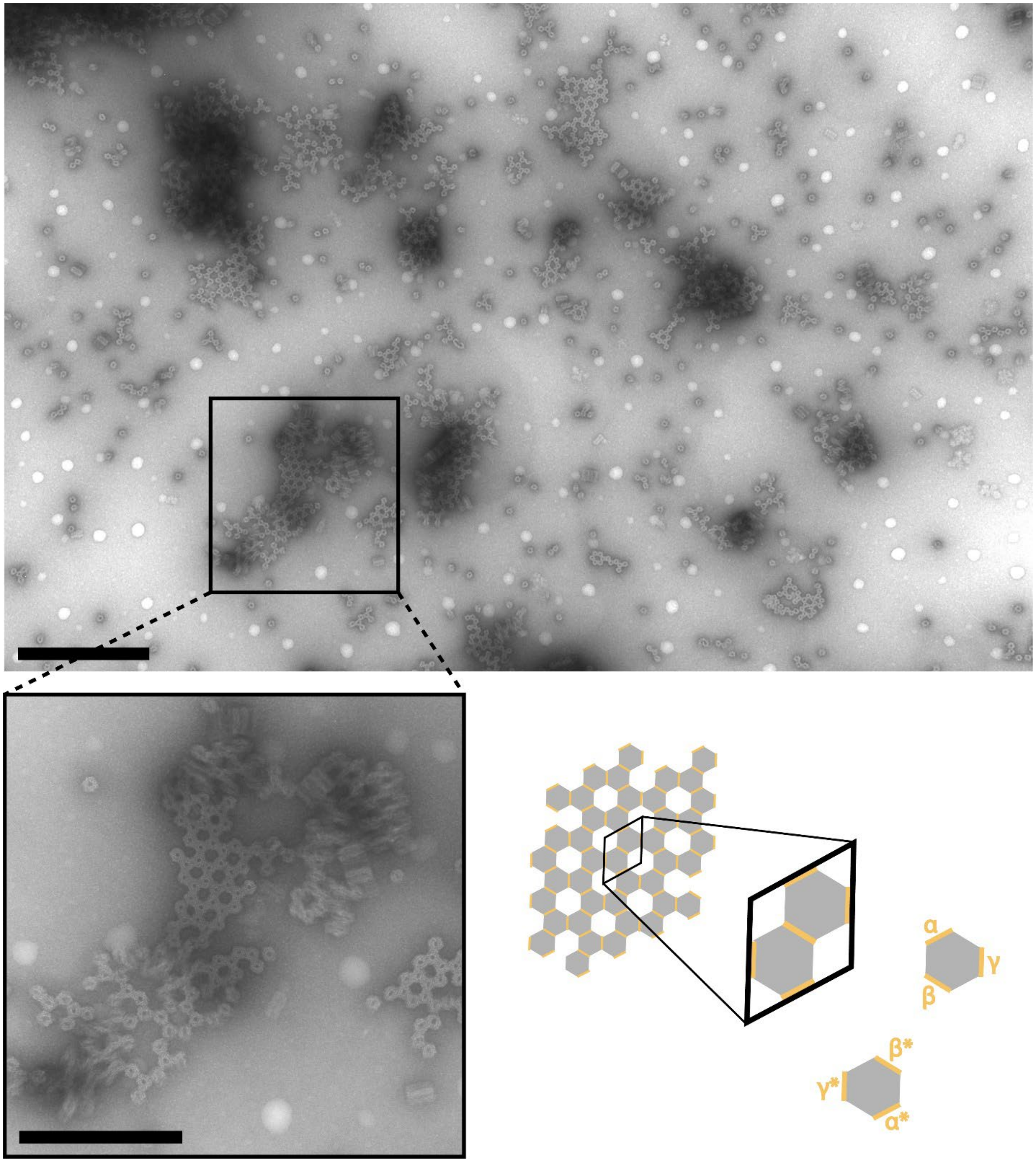


**Figure S33: Infinite xy-structures** were constructed by combining αβγ and α*β*γ* monomers. Together they form a unit cell of more than 1 500 $nm^2$. Enlarged is a piece of ~ 46 monomers spanning an area larger than 36 000 $nm^2$. Scale bar of the overview picture is 500 nm and 250 nm for the enlarged part.

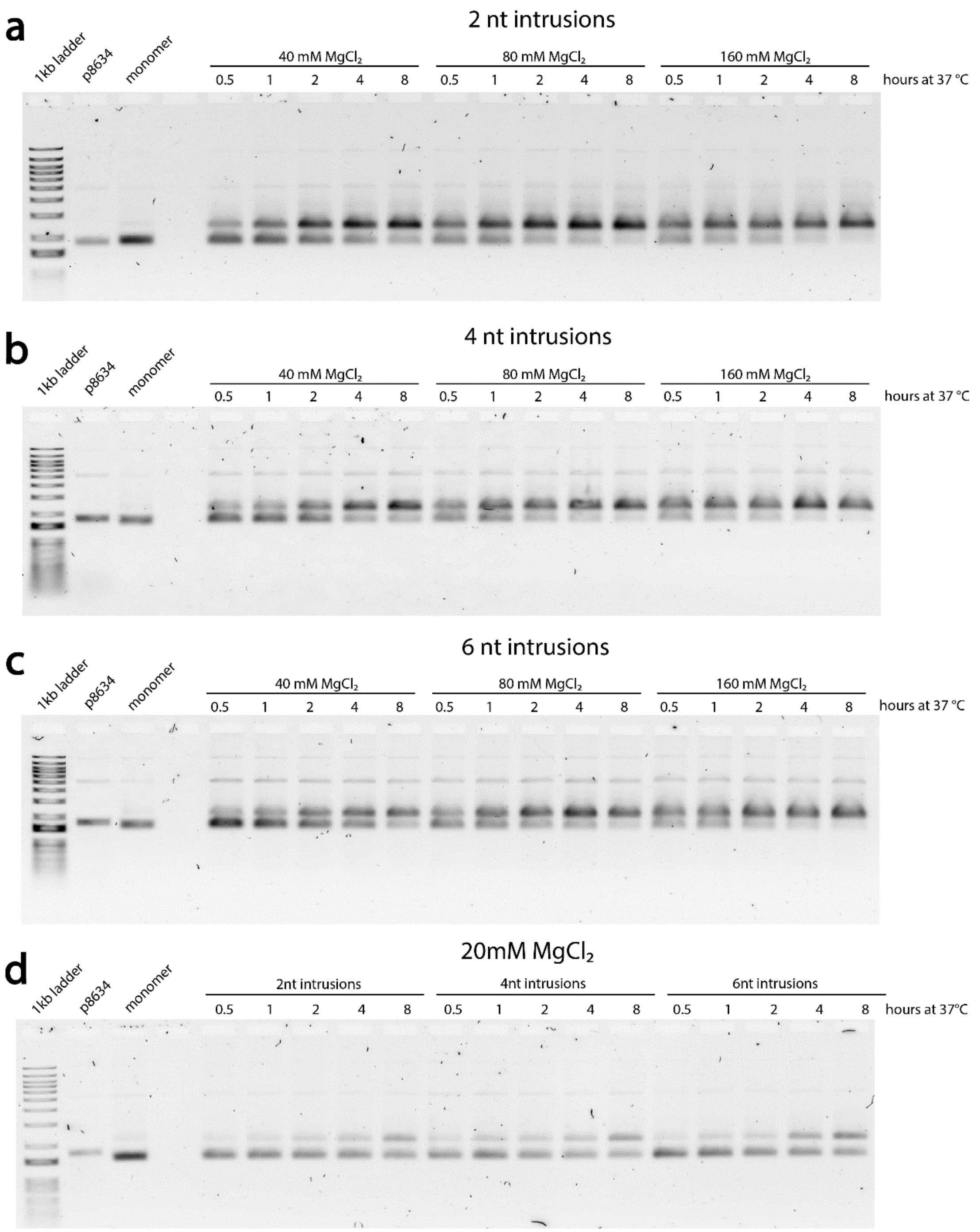


**Figure S34: AGE shift assays of xy-assembly 1:** complementary monomers with (**a**) 2 nt, (**b**) 4 nt, and (**c**) 6 nt staple intrusion were added to buffers containing a total $MgCl_2$ concentration of 40 mM, 80 mM, or 160 mM each, 8, 4, 2, 1, or 0.5 h before AGE was started. (**d**) Complementary monomers with 2 nt, 4 nt and 6 nt were added in a buffer containing 20 mM $MgCl_2$, at time points 8, 4, 2, 1, or 0.5 h before the samples were transferred to the gel. Data also used in Figure 2c.

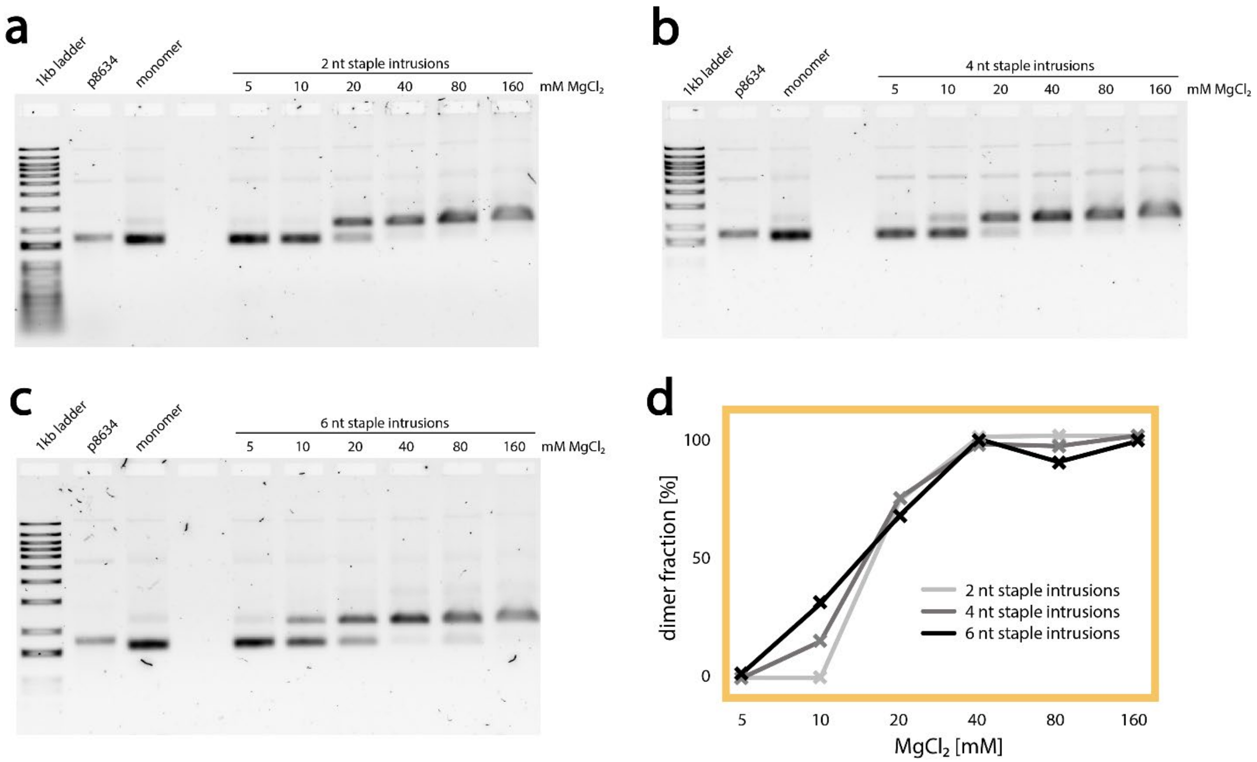


**Figure S35: AGE shift assays of xy-assembly 2**: Analyzing the dimerization after 24 h: complementary monomers with (**a**) 2 nt, (**b**) 4 nt, and (**c**) 6 nt staple intrusion were added to buffers containing a total $MgCl_2$ concentration of 5 mM, 10 mM, 20 mM, 40 mM, 80 mM, or 160 mM each, 24 h before AGE was started. Samples were incubated at 37 °C. Data also shown in Figure 2c. (**d**) Fraction of dimers at different $MgCL_2$ concentrations, as fraction of dimer band intensity to the sum of dimer and monomer band intensity. 100 % dimerization normalized to monomer band.

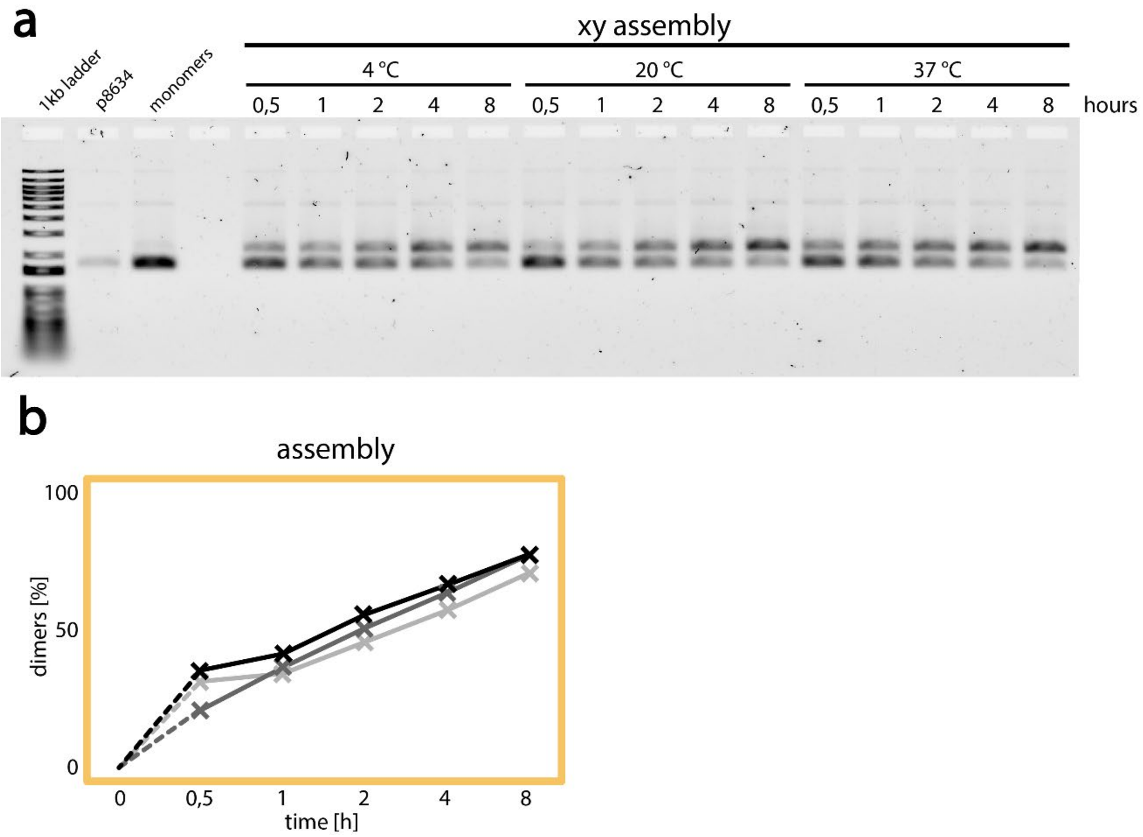


**Figure S36: AGE shift assay on temperature influence on xy-assembly:** (**a**) Complementary monomers with 2 nt overlaps were added in TAE supplemented with 40 mM $MgCl_2$ and incubated at different temperatures: 4 °C, 20 °C, and 37 °C. (**b**) Fraction of dimers at different time points, as fraction of dimer band intensity to the sum of dimer and monomer band intensity. 100 % dimerization normalized to monomer band.

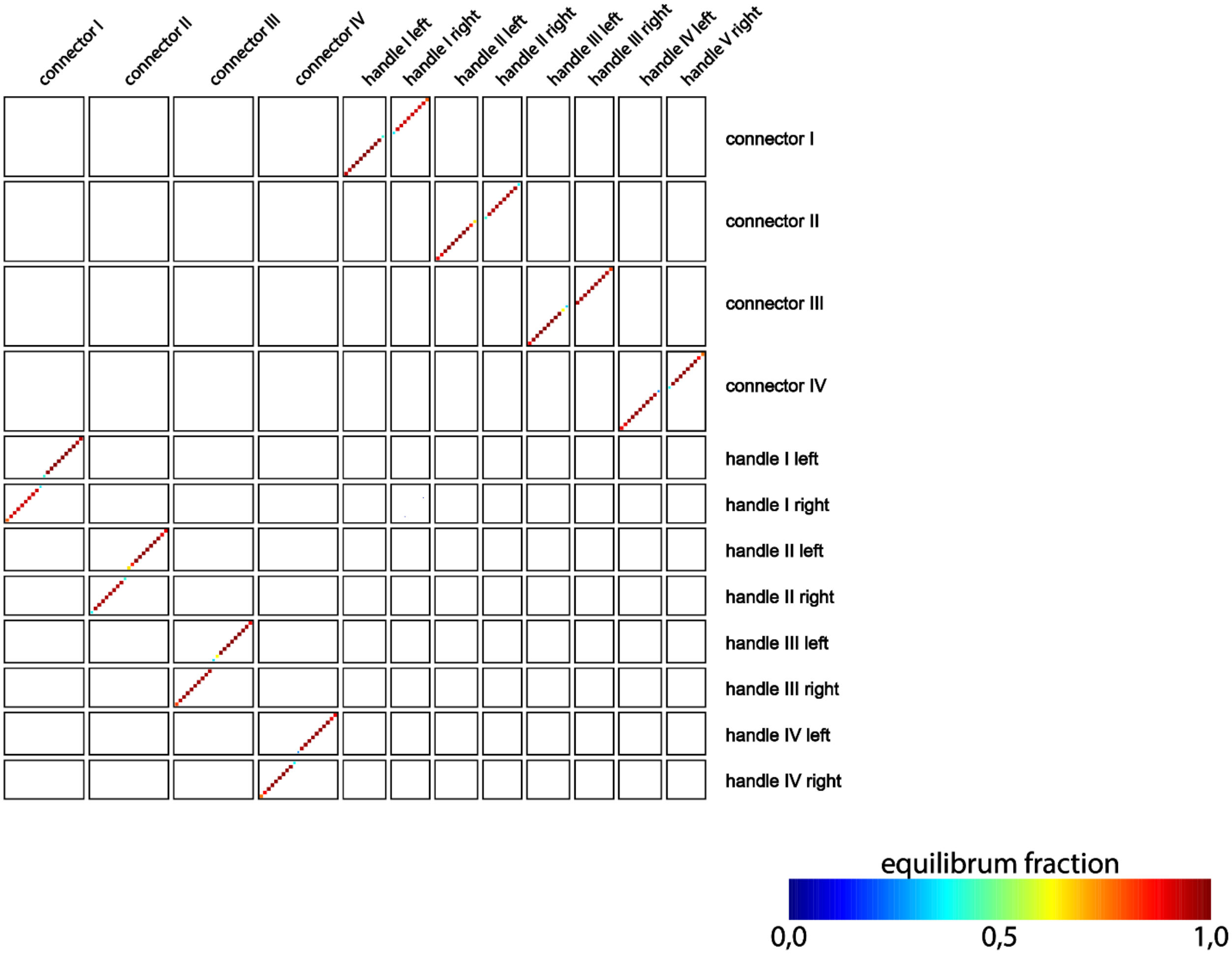


**Figure S37: NUPACK analysis of z-connector orthogonality:** Color scheme indicates complementary connectors and handles bind strongly and selectively with each other even in presence of the other handles and connectors. This shows mutual orthogonality. The simulation was conducted with NUPACK version 2.2 and the following options: 20°C, max. 3 strand complexes, 1 µM per strand, "Serra and Turner 1995", 1 M NaCl.

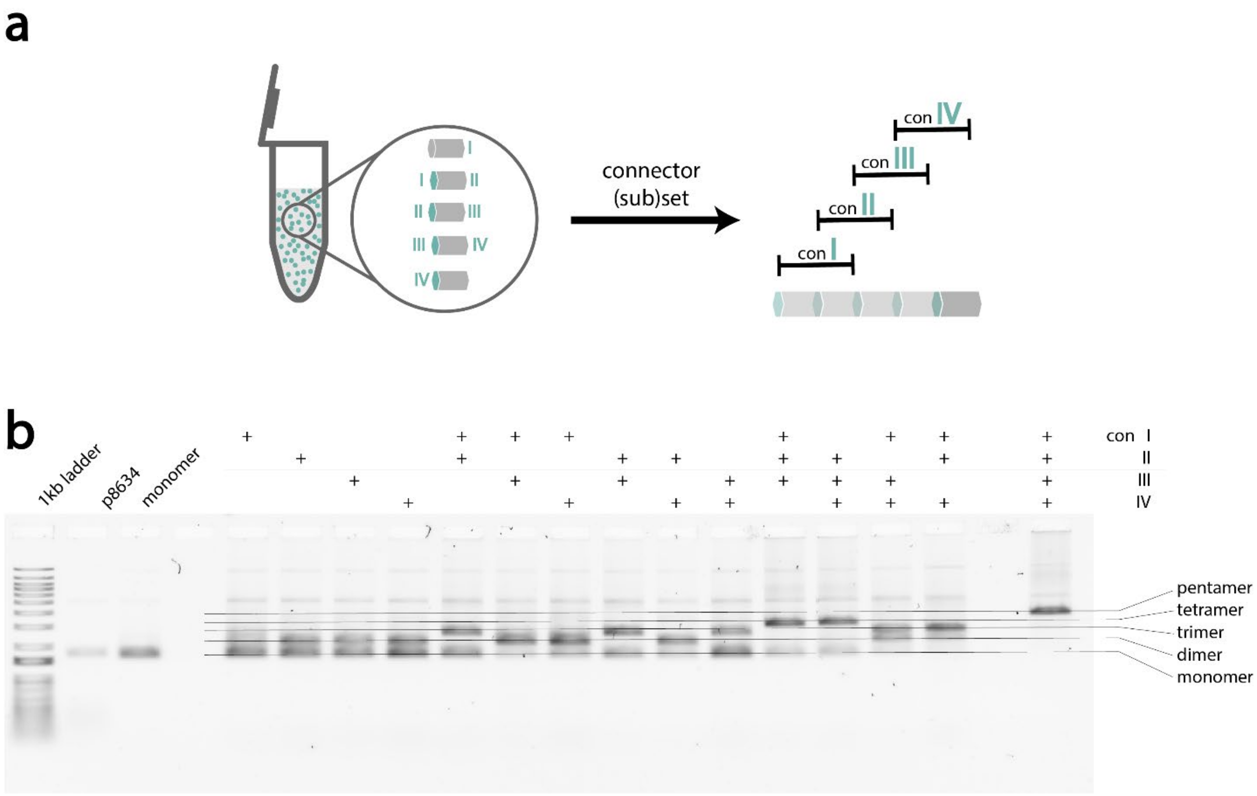


**Figure S38: z-assembly permutations:** (**a**) Schematic of the experiment: To an equimolar mixture of five moDON monomers (zI-right, zI-left/zII-right, zII-left/zIII-right, zIII-left/zIV-right, and zIV-left) subsets of connectors were added and a multitude of moDON multimers in z-direction were created. (**b**) AGE shift assay of z-assembly permutations showed the designed connections of subsets of the moDONs exceptionally well. Addition of single connectors resulted in formation of one part dimers with three parts monomer left unconnected (as only two types of monomers are able to form dimers). Addition of two connectors resulted either in one part trimer and two parts monomers (conI+conII, conII+conIII, or conIII+conIV) or in two parts dimer and one part monomer (conI+conIII, conI+conIV, or conII+conIV). Addition of three connectors resulted either in formation of one part tetramer and one part monomer (conI+conII+conIII or conII+conIII+conIV) or in one part trimer and one part dimer (conI+conIII+conIV or conI+conII+conIV). Addition of all four connector strands yields pentamers.

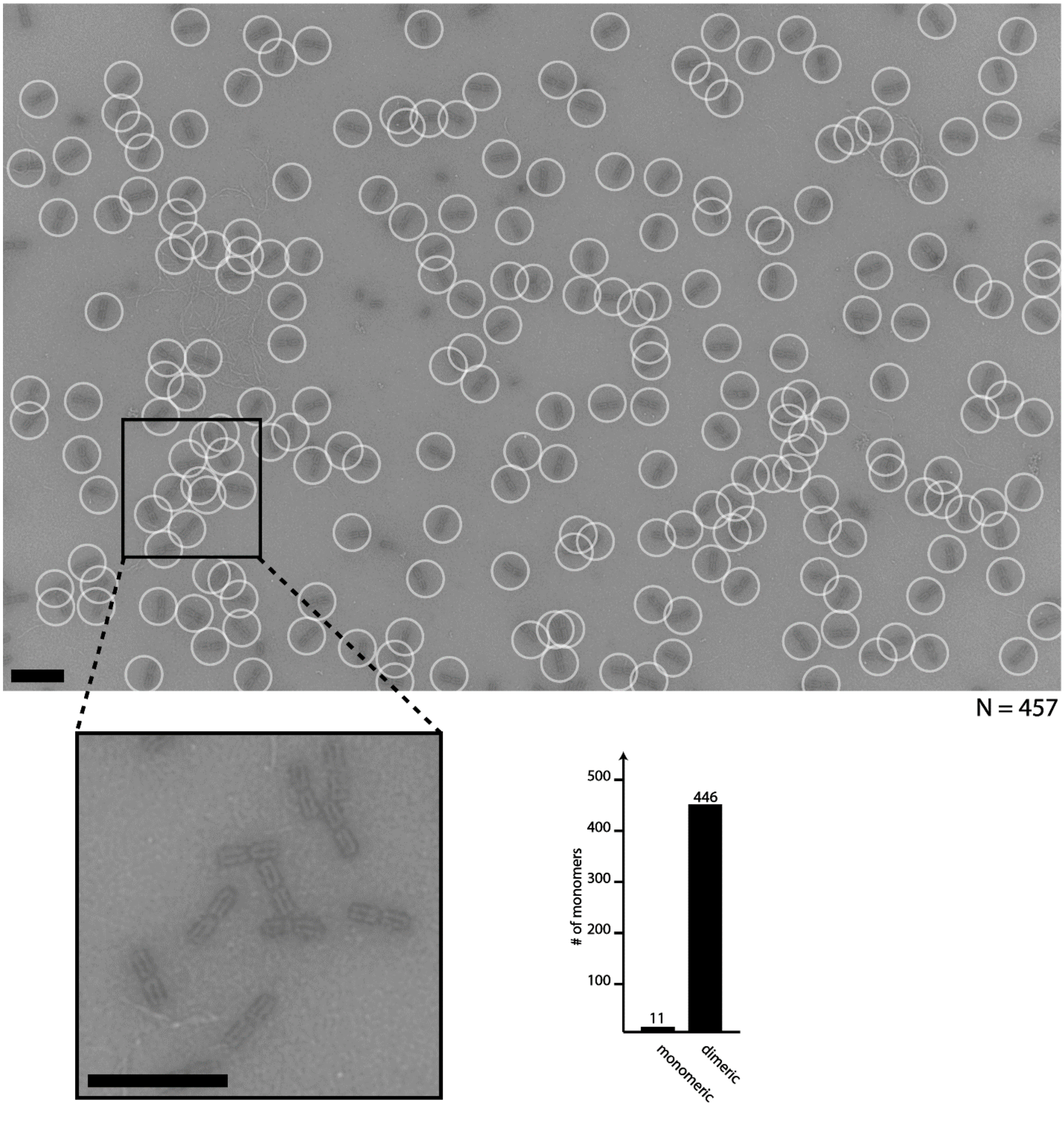


**Figure S39: Wide-field TEM micrograph and statistics of z-dimer formation**. Analysis of the TEM micrograph shows a yield of 97.6 % of dimers, as calculated by the fraction of monomers in the desired superstructure ($N_{dimer}$ = 446) to the total amount of monomers ($N_{\Sigma}$ = 457). Scale bars are 200 nm.

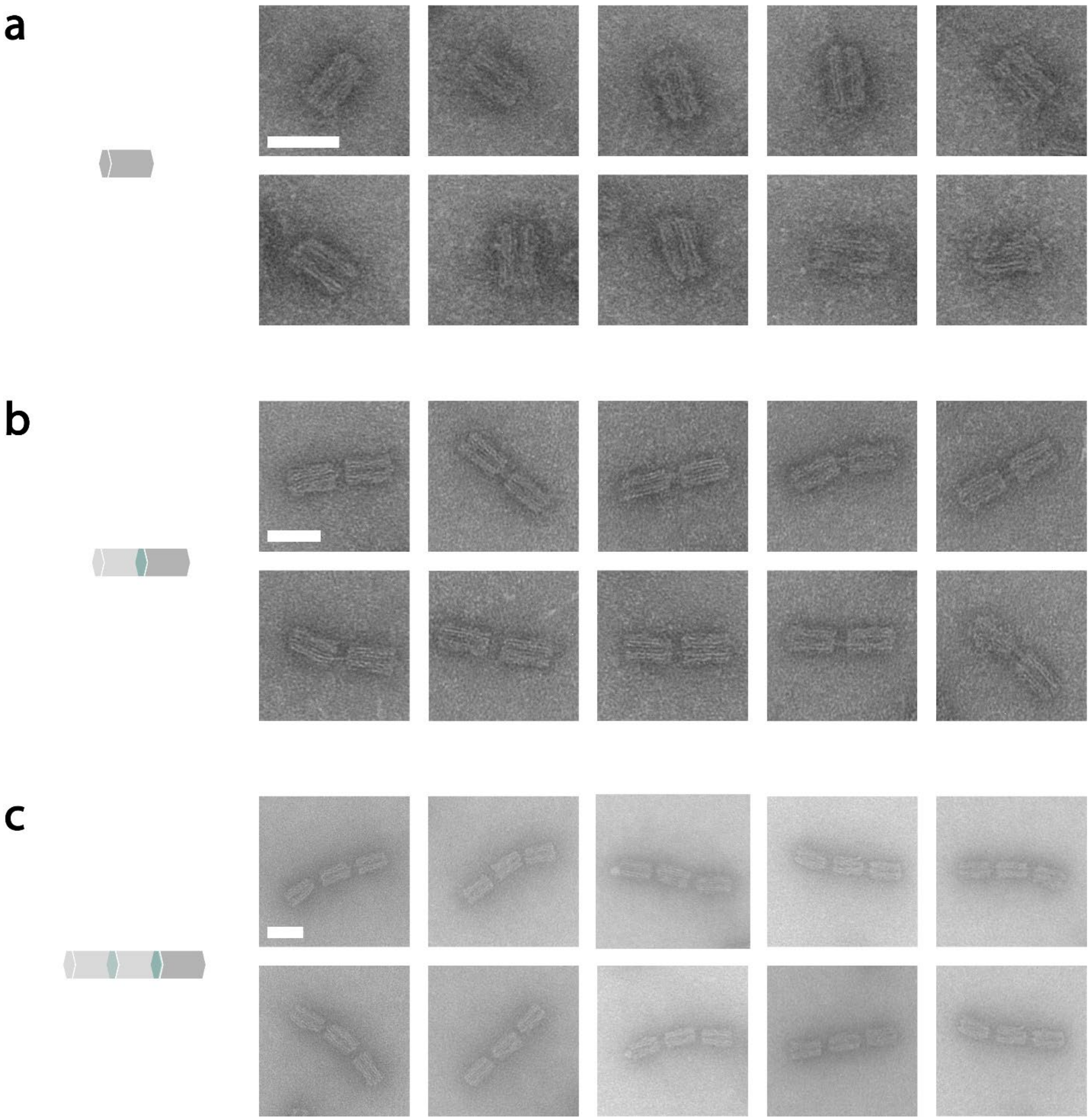

**Figure S40: close-up view on z-structures 1** showing TEM micrographs of (**a**) monomers (**b**) dimers and (**c**) trimers formed by addition of connectors in 5-fold excess to the moDON monomers. Data from the same experiment was also used in Figure 3d. Scale bars are 50 nm and hold for all micrographs of the respective structure.

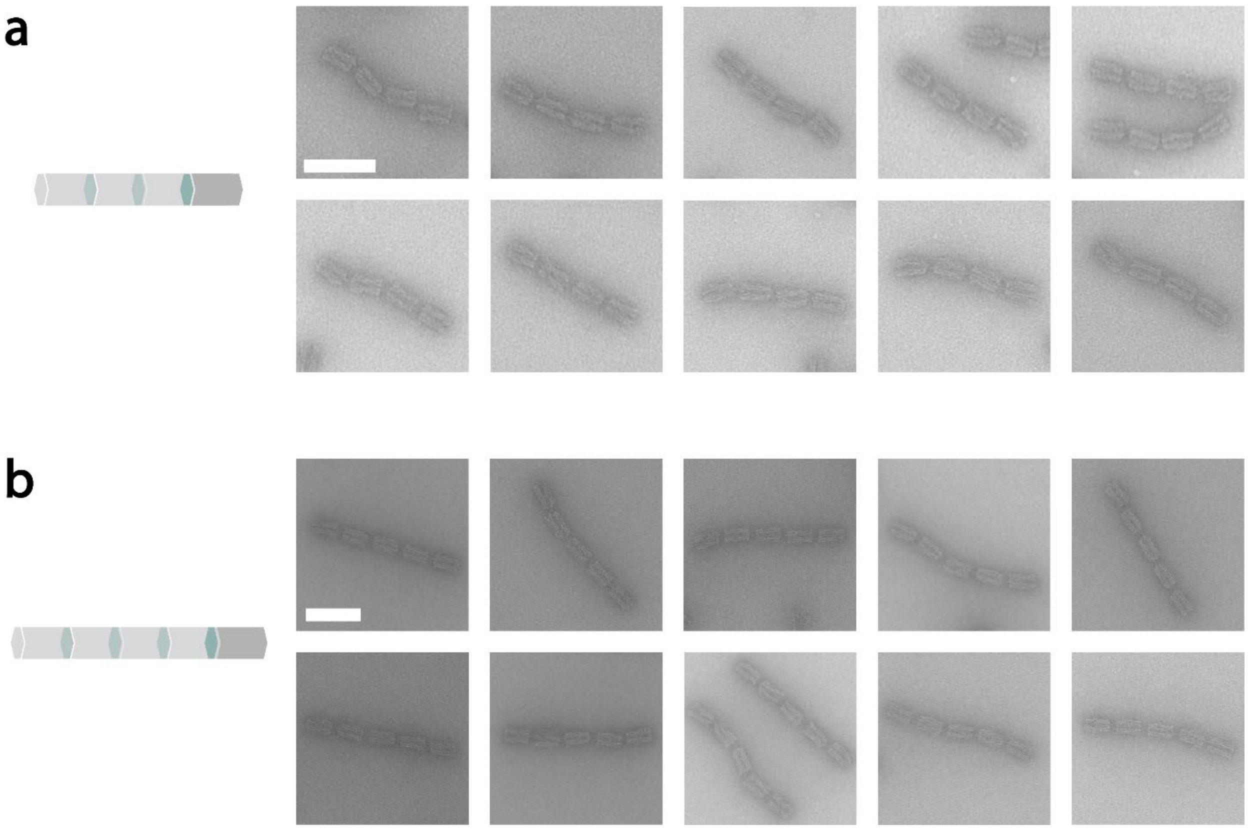


**Figure S41: close-up view on z-structures 2** showing TEM micrographs of (**a**) tetramers and (**b**) pentamers and formed by addition of connectors in 5-fold excess to the moDON monomers. Data from the same experiment was also used in Figure 3d. Scale bars are 100 nm and hold for all micrographs of the respective structure.

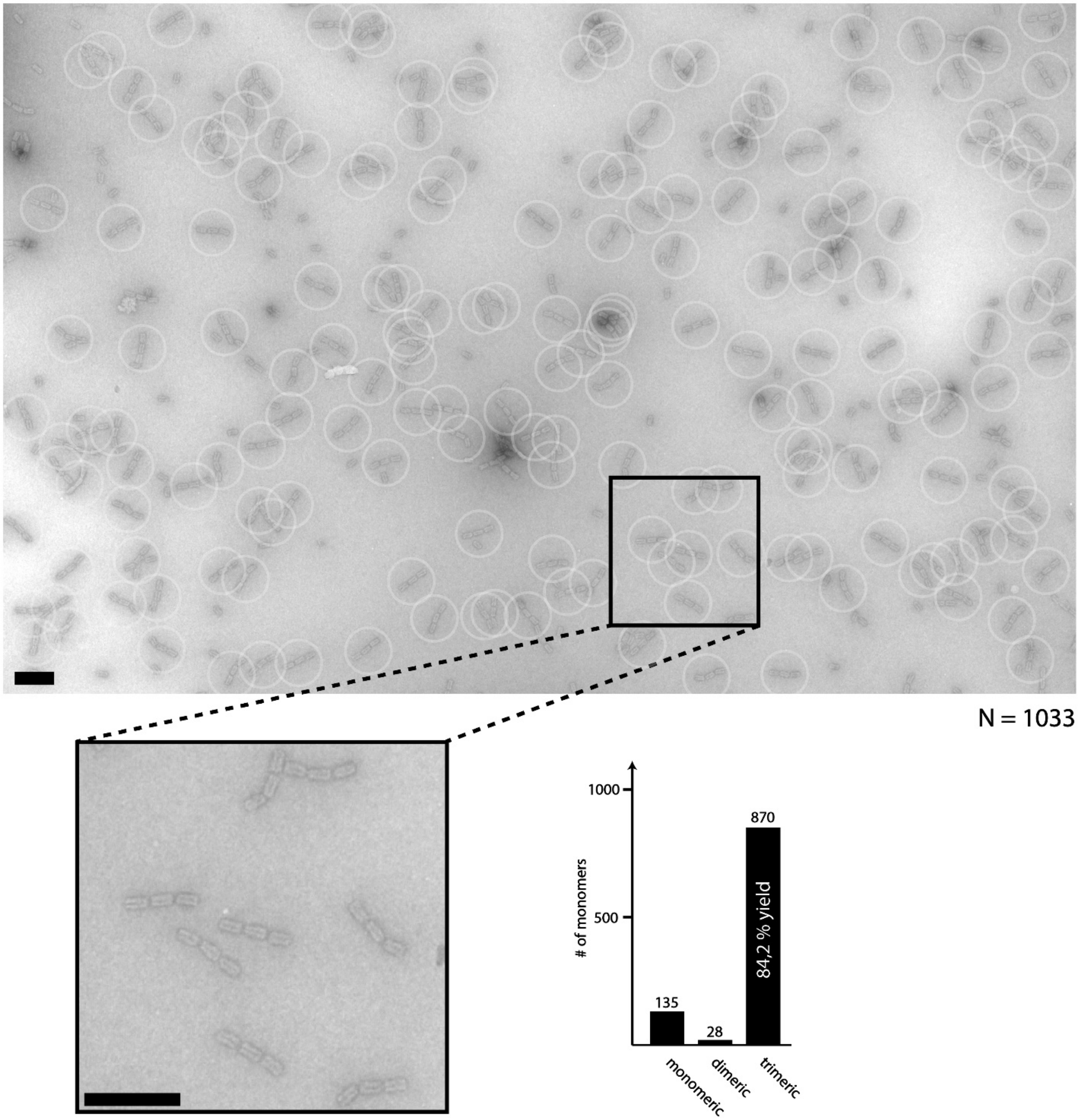


**Figure S42: Wide-field TEM micrograph and statistics of z-trimer formation**. Analysis of the TEM micrograph shows a yield of 84.2 % of trimers, as calculated by the fraction of monomers in the desired superstructure ($N_{trimer}$ = 870) to the total amount of monomers ($N_{\Sigma}$ = 1033). Scale bars are 200 nm.

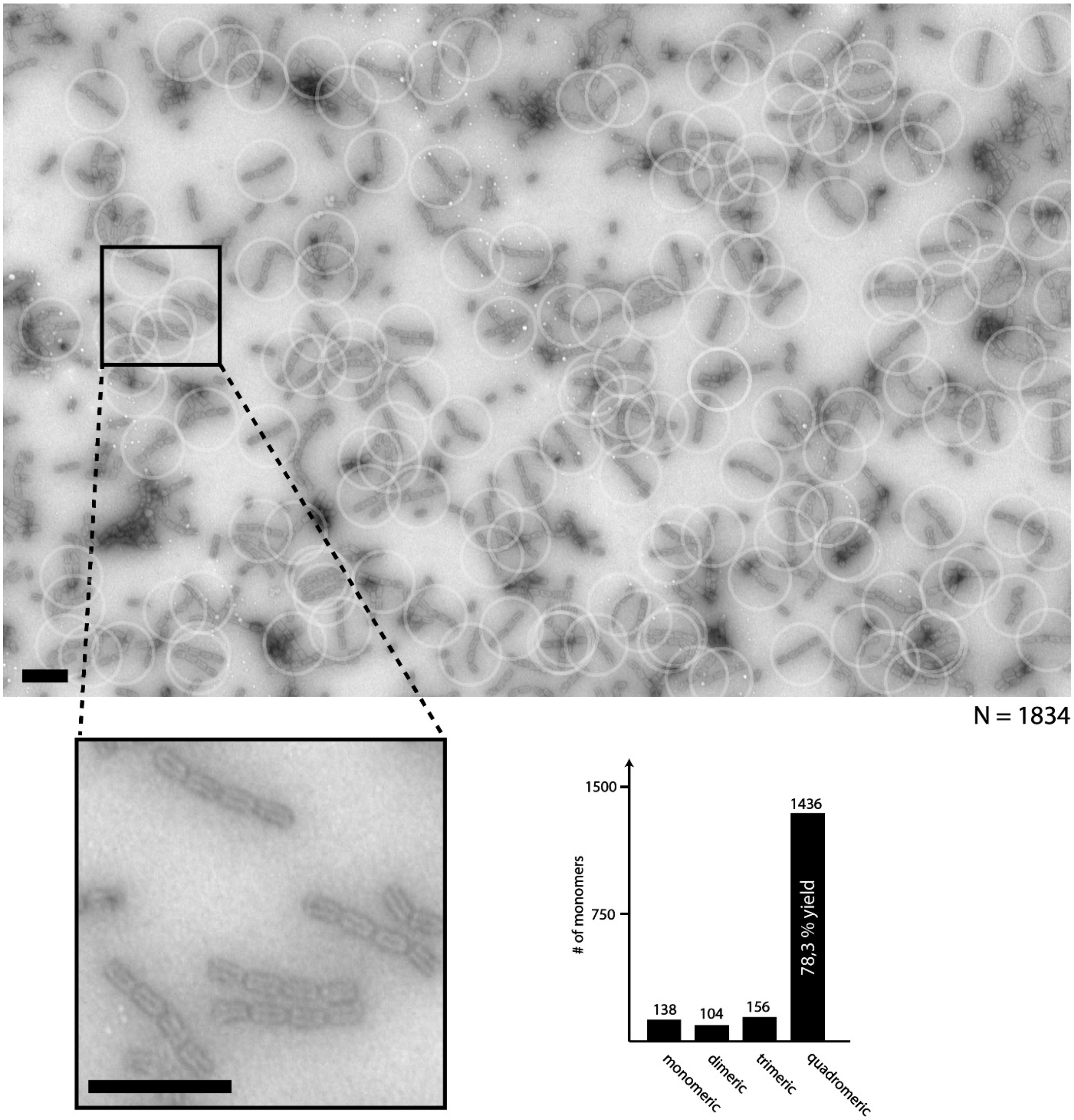


**Figure S43: Wide-field TEM micrograph and statistics of z-tetramer formation**. Analysis of the TEM micrograph shows a yield of 78.3 % of tetramers, as calculated by the fraction of monomers in the desired superstructure ($N_{tetramer}$ = 1436) to the total amount of monomers ($N_{\Sigma}$ = 1834). Scale bars are 200 nm.

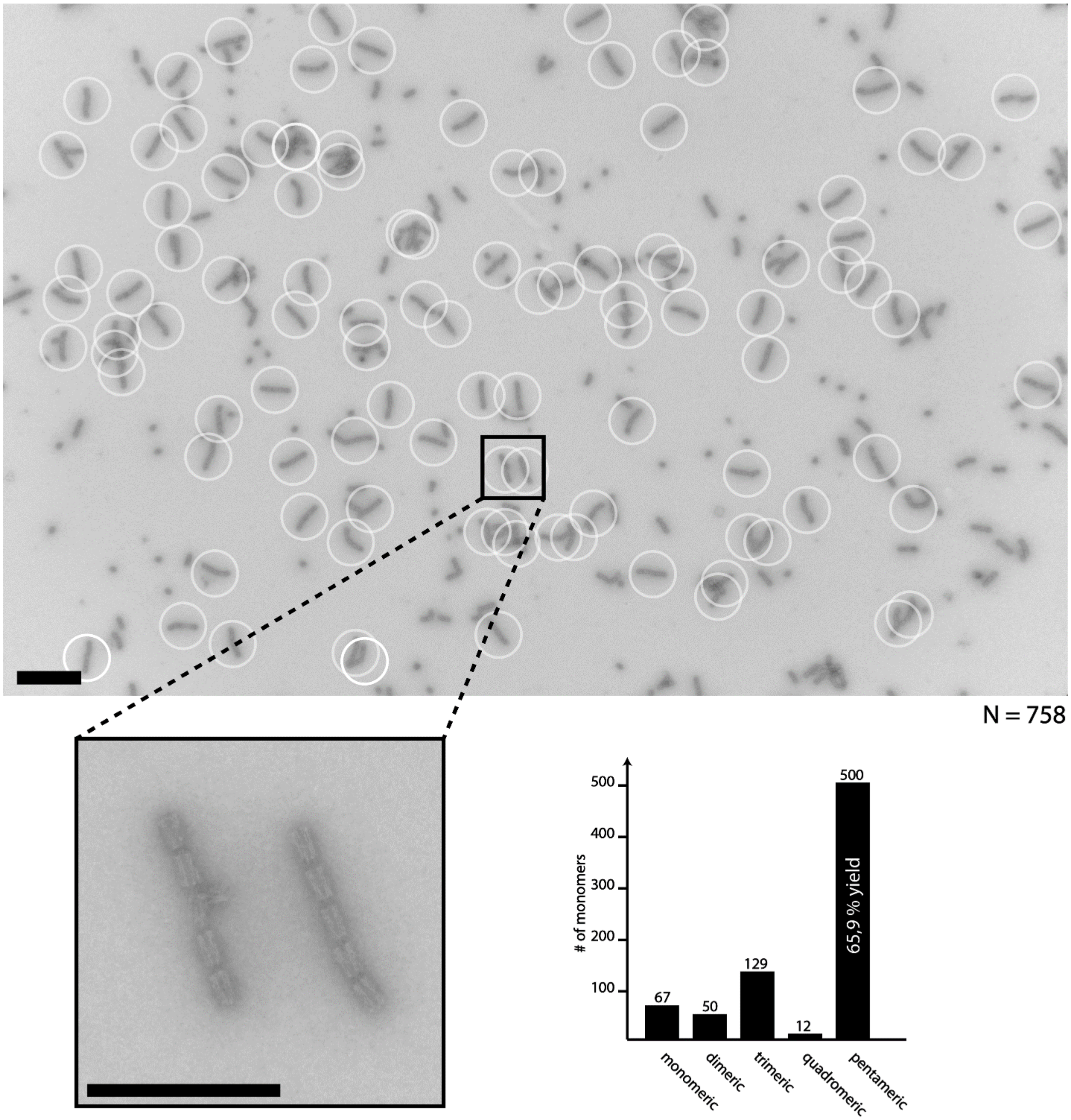


**Figure S44: Wide-field TEM micrograph and statistics of z-pentamer formation**. Analysis of the TEM micrograph shows a yield of 65.9 % of pentamers, as calculated by the fraction of monomers in the desired superstructure ($N_{pentamer}$ = 500) to the total amount of monomers ($N_{\Sigma}$ = 758). Scale bars are 200 nm.

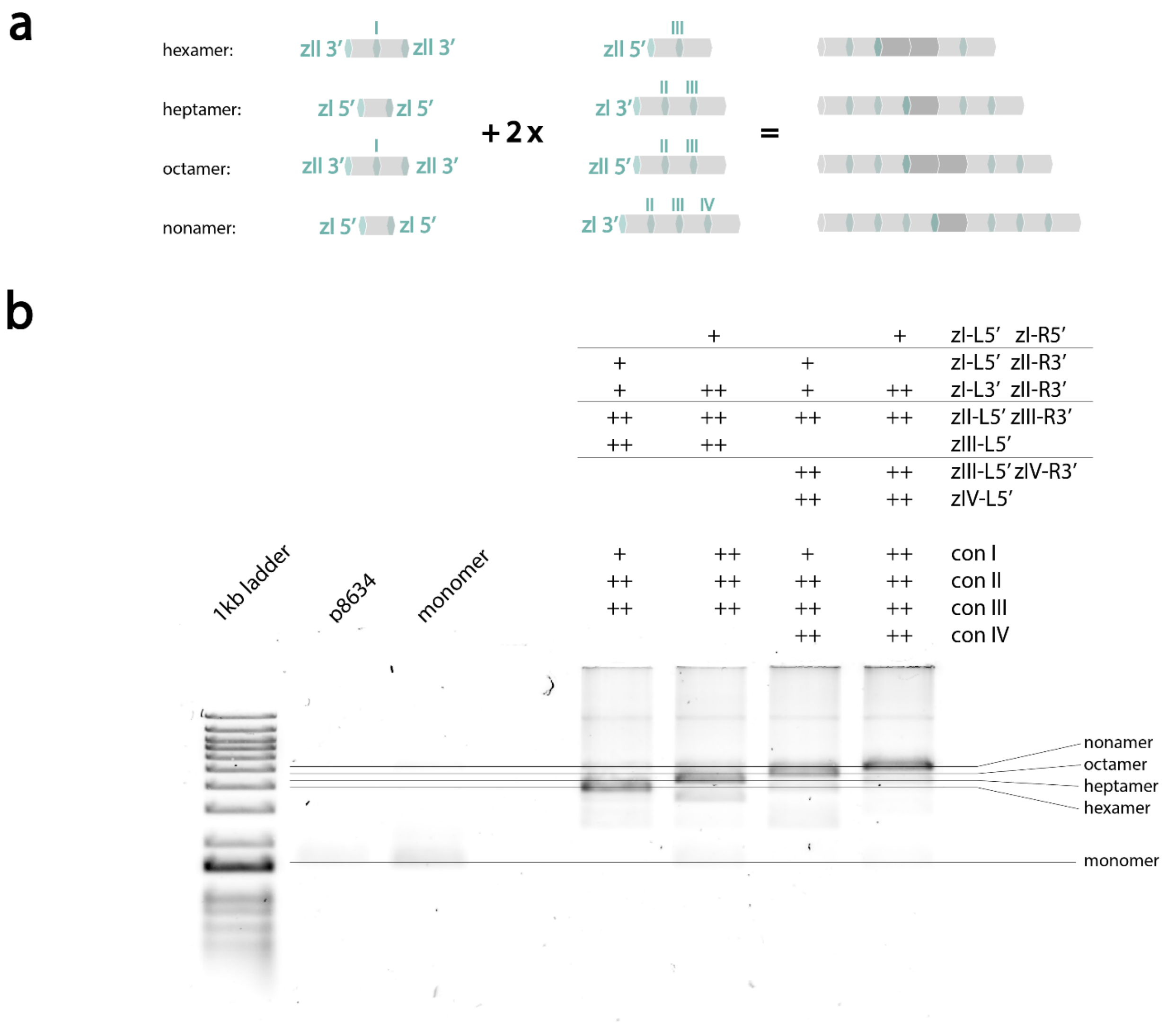


**Figure S45: symmetrically assembled z-structures** are made as shown in (**a**) by construction of a symmetric center piece, to whose ends two dimeric, trimeric or tetrameric pieces connect and make hexameric to nonameric finite structures in the end. (**b**) AGE of symmetrically assembled structures shows reduced electrophoretic mobility proportional to structure size. The respective yields were are the hexamer 70.4 %, for the heptamer 61.0 %, for the octamer 56.8 %, and for the nonamer 60.9 %. The same gel images is used in Figure 3e.

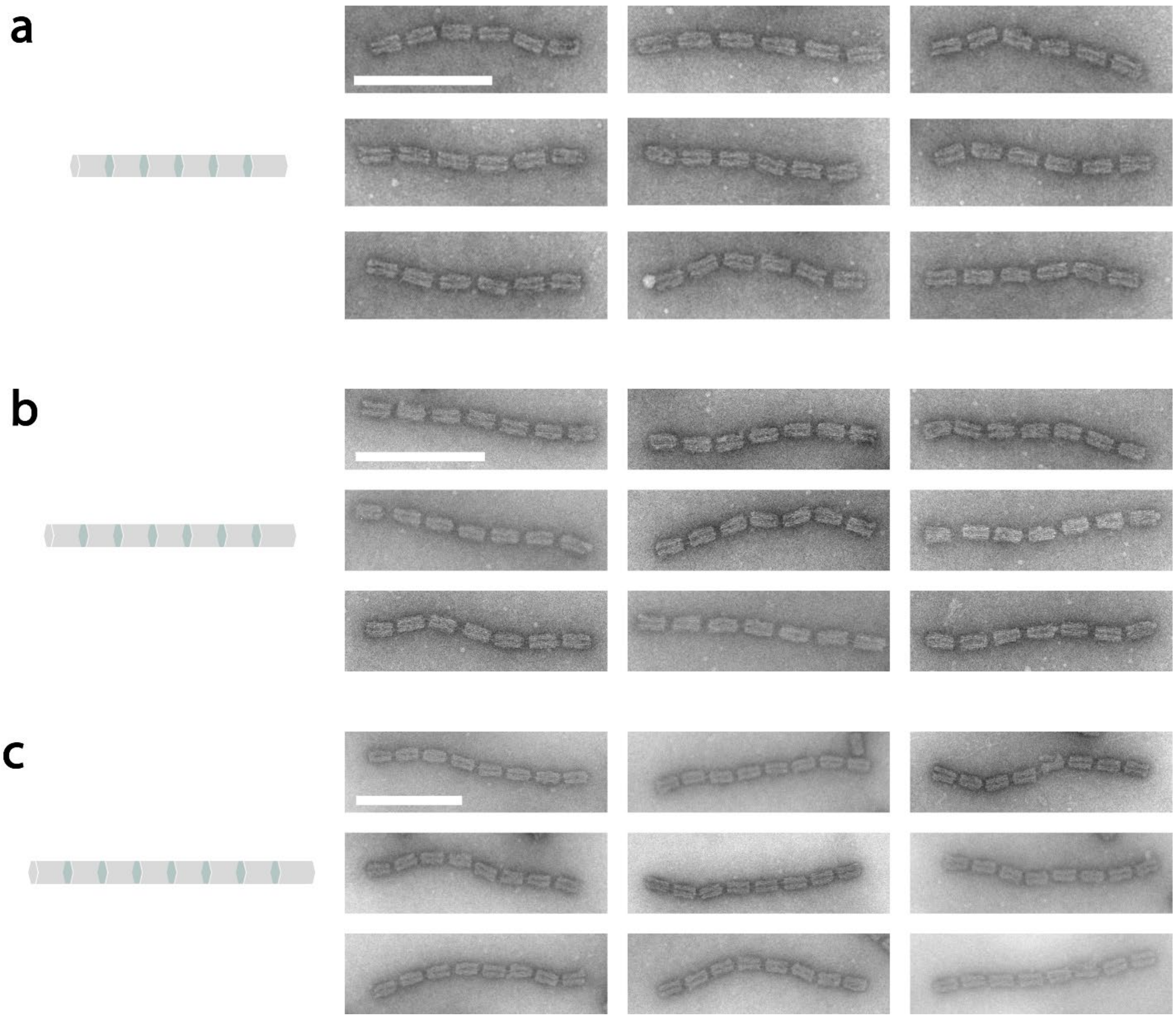

**Figure S46: close-up view on z-structures 3** showing TEM micrographs of (**a**) hexamers, (**b**) heptamers, and (**c**) octamers and formed by addition of connectors in 5-fold excess to the moDON monomers. Data from the same experiment was also used in Figure 3g. Scale bars are 200 nm and hold for all micrographs of the respective structure.

a

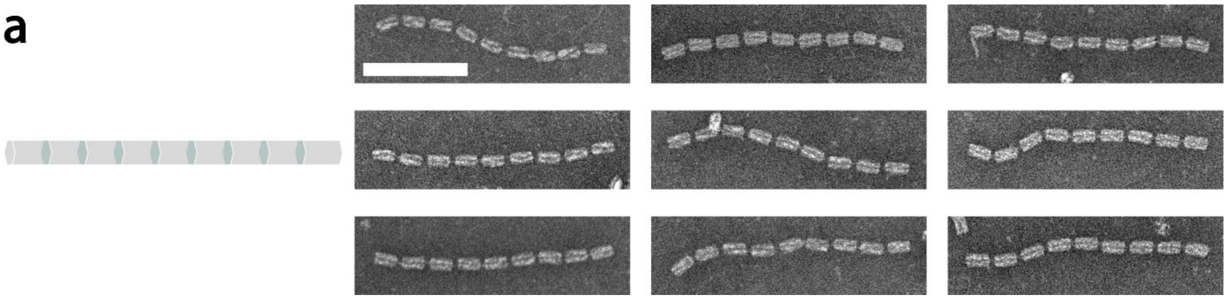

**Figure S47: close-up view on z-structures 4** showing TEM micrographs of (**a**) nonamers and formed by addition of connectors in 5-fold excess to the moDON monomers. Data from the same experiment was also used in Figure 3g. Scale bar is 200 nm and holds for all micrographs.

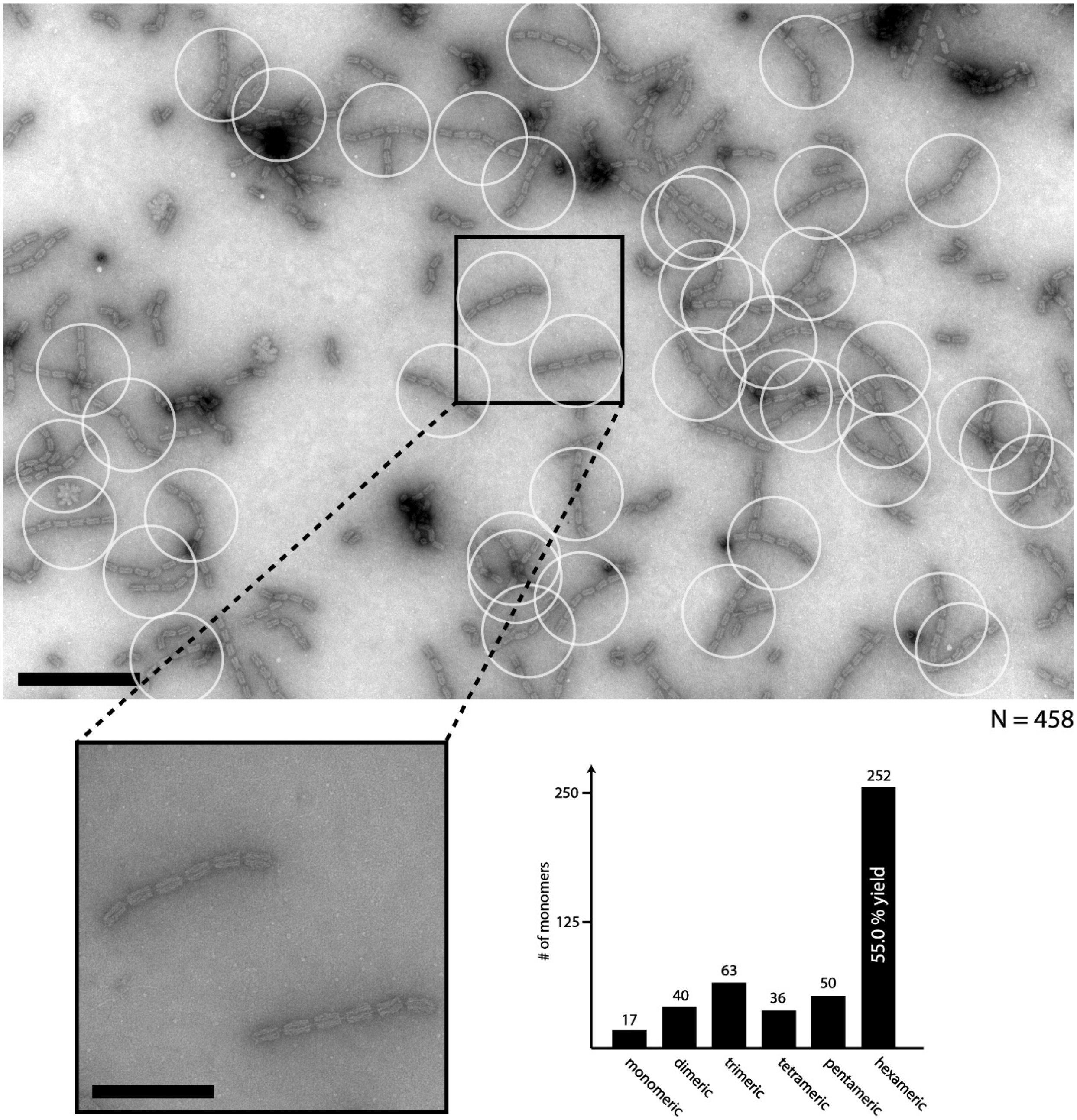


**Figure S48: Wide-field TEM micrograph and statistics of z-hexamer formation**. Analysis of the TEM micrograph shows a yield of 55.0 % of hexamers, as calculated by the fraction of monomers in the desired superstructure ($N_{hexamer}$ = 252) to the total amount of monomers ($N_{\Sigma}$ = 458). Scale bars are 200 nm.

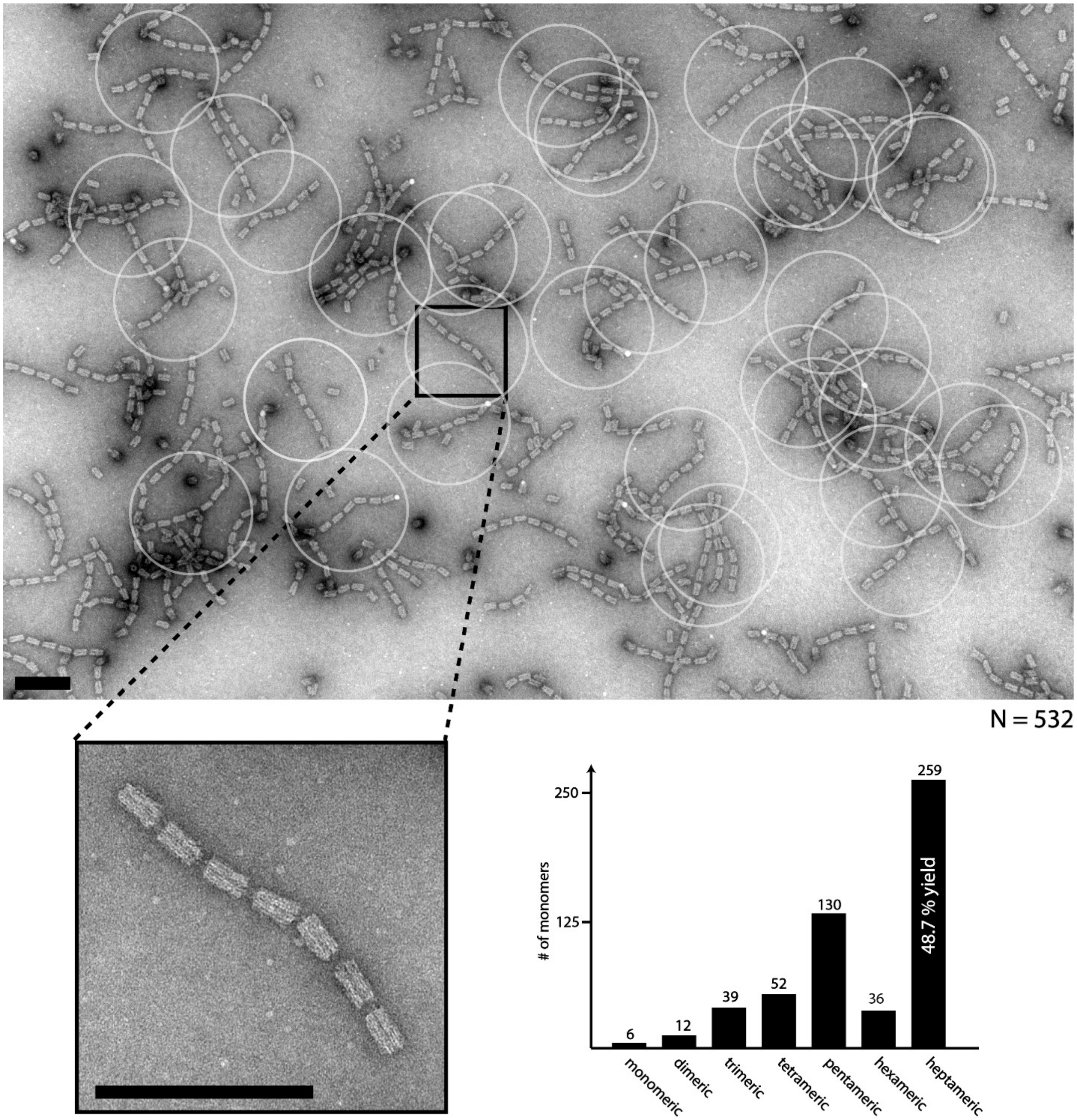


**Figure S49: Wide-field TEM micrograph and statistics of z-heptamer formation**. Analysis of the TEM micrograph shows a yield of 48.7 % of heptamers, as calculated by the fraction of monomers in the desired superstructure ($N_{heptamer}$ = 259) to the total amount of monomers ($N_{\Sigma}$ = 532). Scale bars are 200 nm.

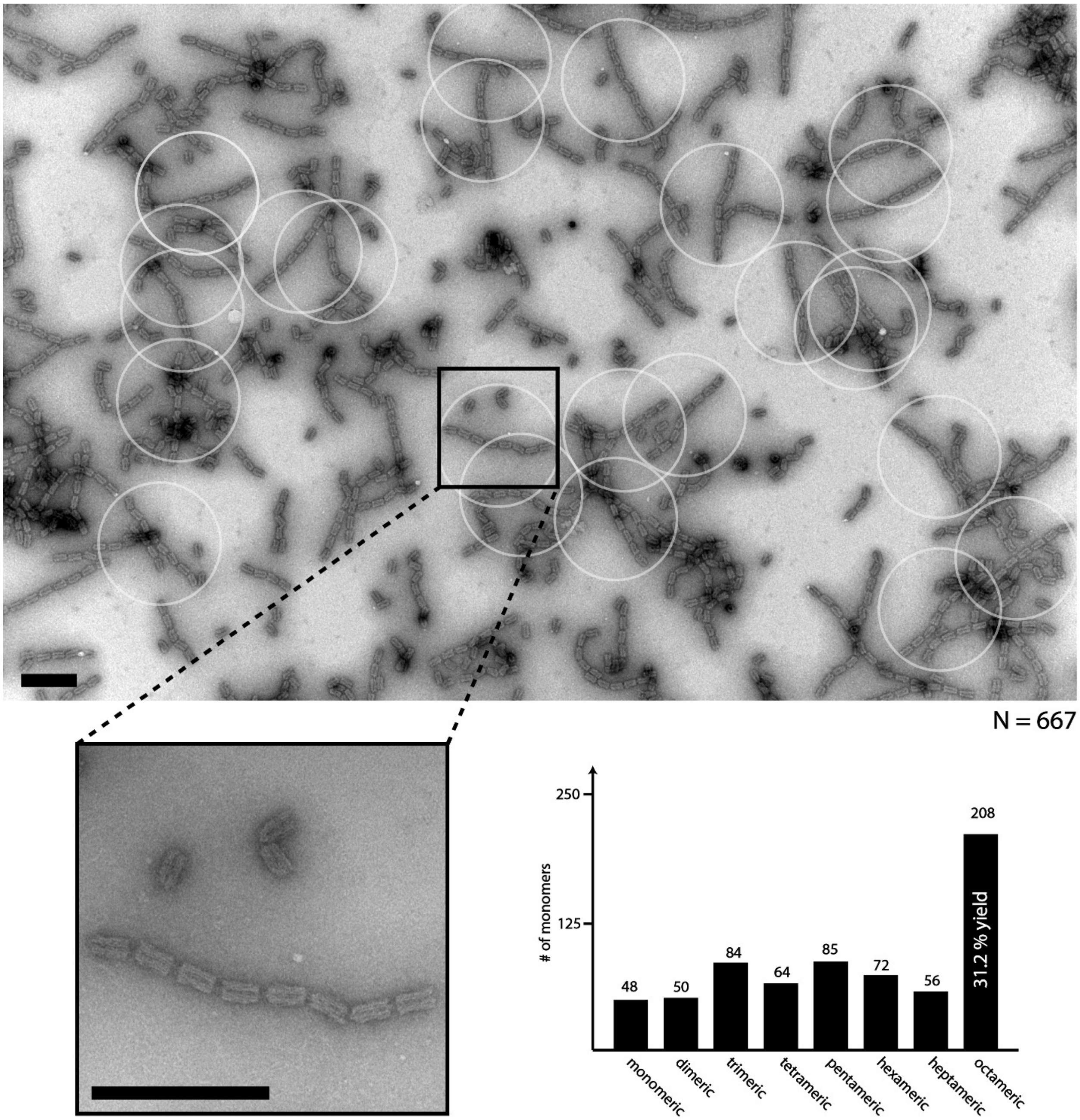


**Figure S50: Wide-field TEM micrograph and statistics of z-octamer formation**. Analysis of the TEM micrograph shows a yield of 31.2 % of octamers, as calculated by the fraction of monomers in the desired superstructure ($N_{octamer}$ = 208) to the total amount of monomers ($N_{\Sigma}$ = 457). Scale bars are 200 nm.

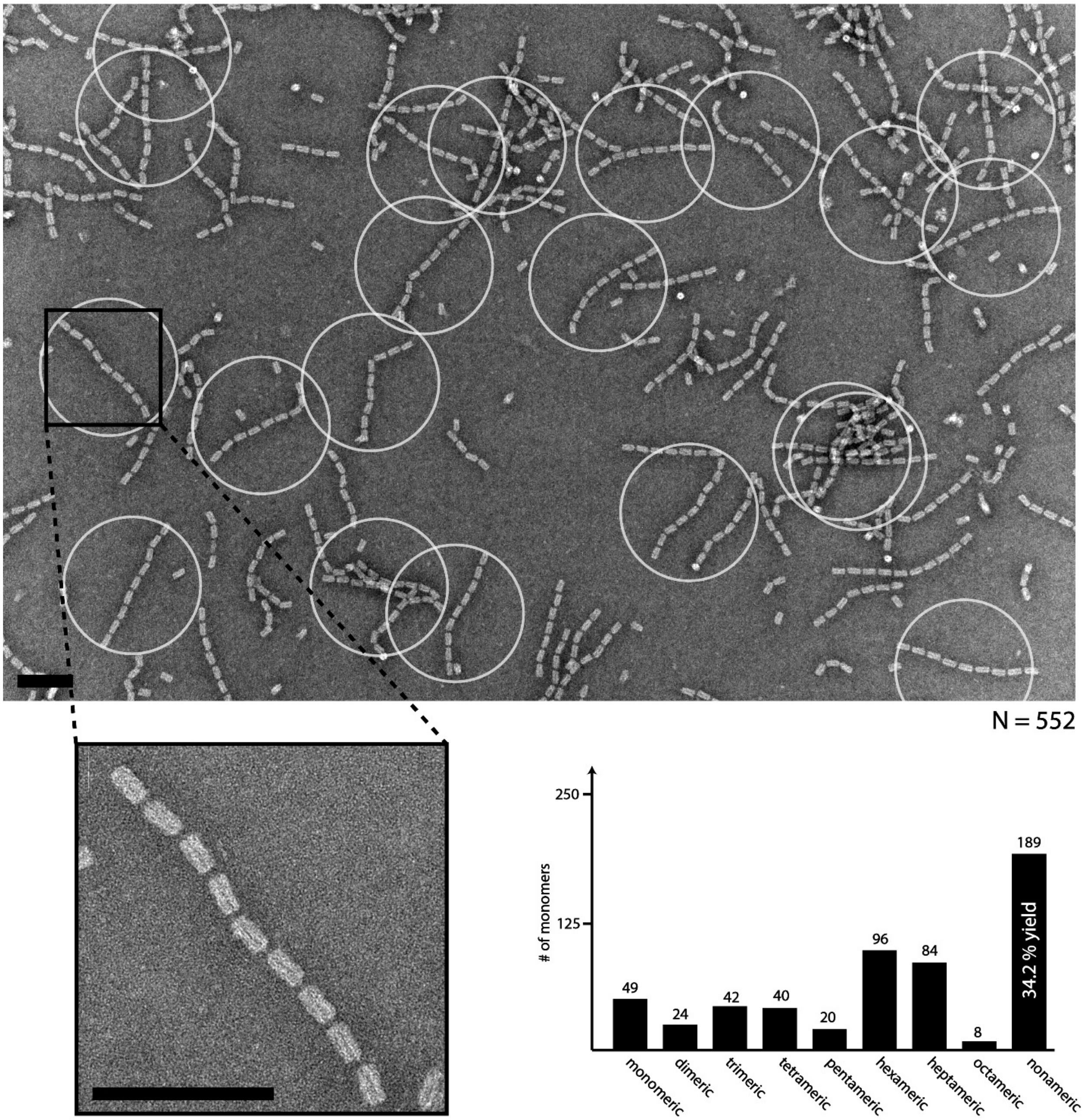


**Figure S51: Wide-field TEM micrograph and statistics of z-nonamer formation**. Analysis of the TEM micrograph shows a yield of 34.2 % of nonamers, as calculated by the fraction of monomers in the desired superstructure ($N_{nonamer}$ = 189) to the total amount of monomers ($N_{\Sigma}$ = 552). Scale bars are 200 nm.

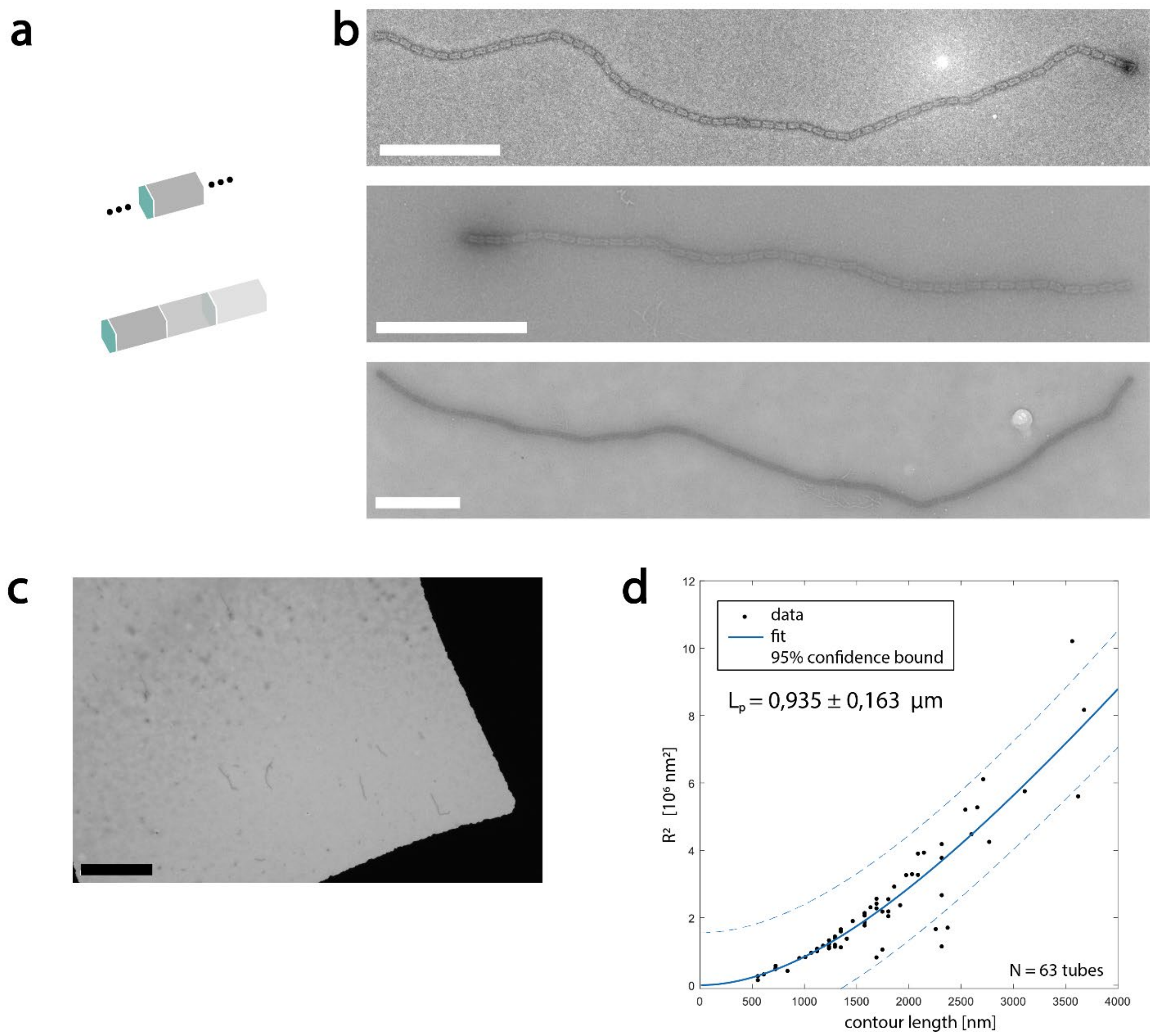


**Figure S52: Infinite tube with monomeric subunit** is constructed with the moDON zI-left/zI-right, the repetitive subunit is shown in (**a**). z-connections are indicated in turquois. (**b**) TEM micrographs of exemplary structures: Number of subunits (= monomers) is 64, 42, and 88 (from top to bottom), corresponding to approximately 3.6 µm, 2.4 µm, and 5.0 µm contour length, and 0.36 GDa, 0.24 GDa, and 0.49 GDa weight, respectively. Bottommost micrograph is also displayed in Figure 3e. (**c**) Wide-field TEM micrograph showing large periodic structure already visible at very low magnification. (**d**) Analysis of end-to-end distance (squared on y axis) and contour length (x axis) of N=63 structures revealed a persistence length of 0.935 ± 0.163 µm. Data of (**b**) is also shown in Figure 3e. Scale bars in (**b**) are 500 nm and in (**c**) 10 µm.

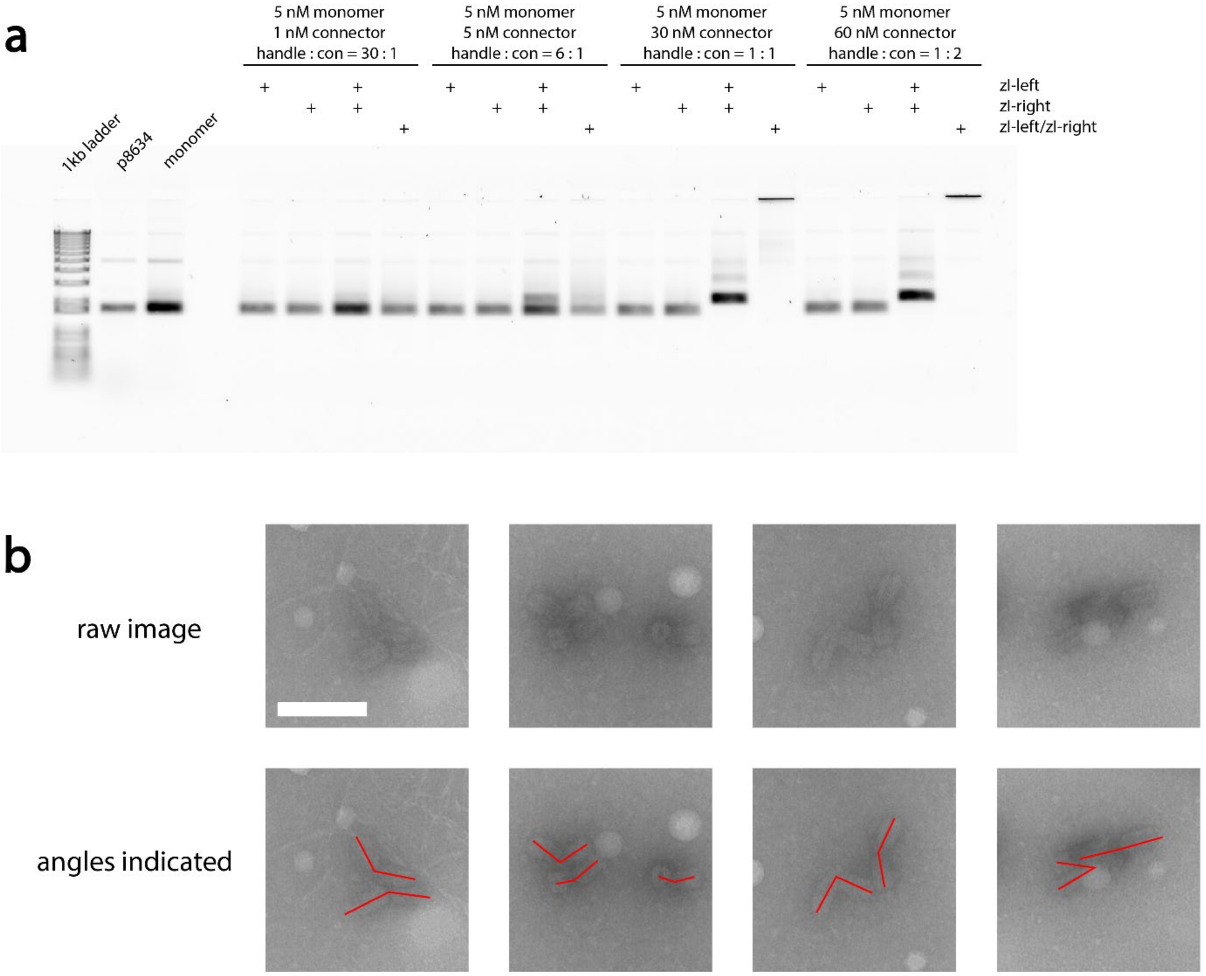


**Figure S53: z-connections with various connector concentrations** (**a**) were constructed from moDONs with handles at the left, the right or both end. Ratio of handles to connectors was varied from 30:1 to 1:2. Full dimerization, or multimerization (for moDONs with both connection sites) was reached with 1:1 handle : connector ratio. We also noted, that for the 1:1 ratio the infinitely multimerized superstructures did not migrate through the gel, indicating a very high degree of multimerization. The lower, 6:1 ratio of handles to connectors, yielded a low fraction of moDONs dimerized, and further division of connector concentration by a factor of five, did not yield dimers at all. (**b**) TEM micrographs of z-dimers with equimolar connector-handle ratio. Connections are mostly bent along the z-axis. Below the raw micrograph, the same image is duplicated, with the angles between both monomers indicated in red. Scale bar is 100 nm and holds for all micrographs.

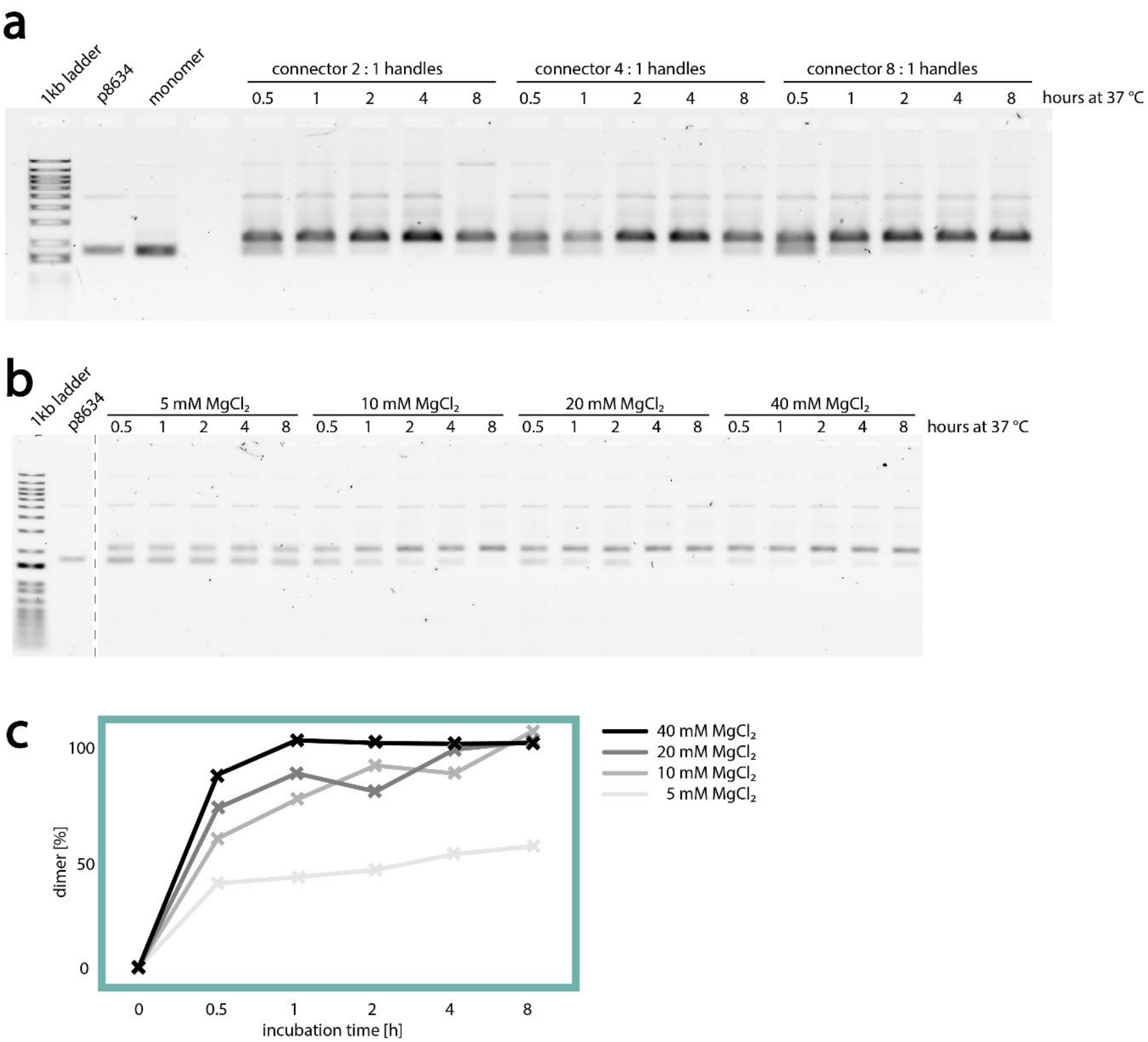


**Figure S54: AGE shift assays of z-assembly:** (**a**) Influence of connector excess: Dimers in z-direction were formed by addition of connector strands in 2-, 4-, or 8-fold excess to handles, each 8, 4, 2, 1, or 0.5 h before the samples were transferred to the gel. Data was also used in Figure 3b. (**b**) Influence of $MgCl_2$ concentration: z-directional dimers were formed by addition of connector strands in 5-fold excess to handles in TAE buffer containing 5, 10, 20, or 40 mM $MgCl_2$, each 8, 4, 2, 1, or 0.5 h before the samples were transferred to the gel and the AGE was started. (**c**) $MgCl_2$ dependency of dimerization over time (from analysis of gel bands: comparison of fraction of dimer band intensity to the sum of dimer and monomer band intensity). 100% dimerization normalized to monomer band. 0 h incubation time was set to 0 % dimerization manually.

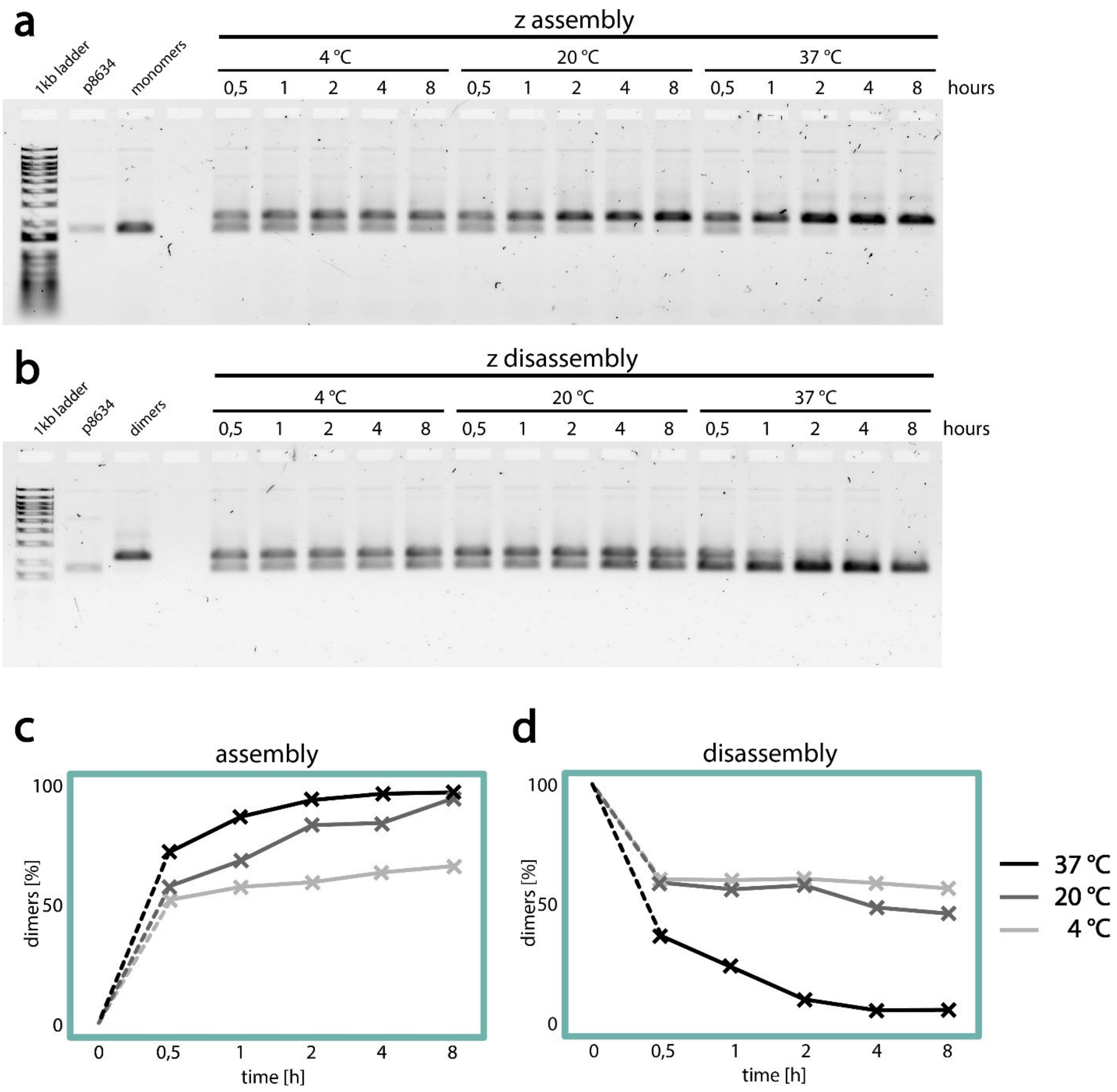


**Figure S55: AGE shift assay on temperature influence on z-assembly and disassembly:** (**a**) The connector strand as added to the respective monomers and incubated at different temperatures: 4 °C, 20 °C, and 37 °C. (**b**) To already assembled z-dimers the respective invader strand was added and incubated at 4 °C, 20 °C,or 37 °C. Addition happened 30 min, 1 h, 2 h, 4 h, or 8 h before AGE was started. (**c**), (**d**) Fraction of dimers at different time points, as fraction of dimer band intensity to the sum of dimer and monomer band intensity. 100 % dimerization normalized to monomer band in (**c**) and dimer band in (**d**).

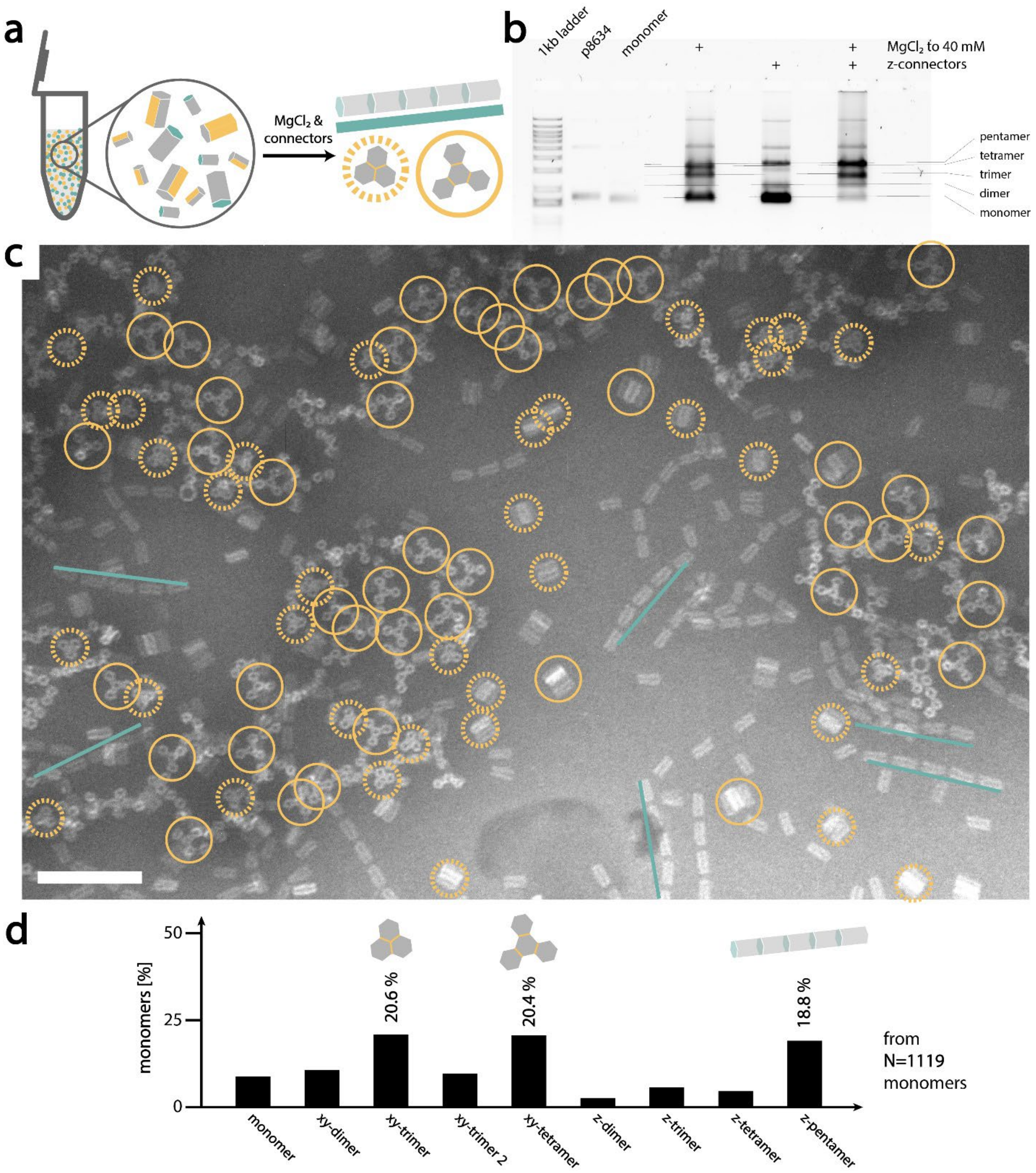


**Figure S56: Parallel assembly:** (**a**) Schematic of experimental setup: $MgCl_2$ and z-connectors were added to the same mixture of 12 moDON monomers, and three distinctly different structures formed parallelly through the mutual orthogonality of the connection sides. (**b**) AGE shift assay of parallel formation: Addition of $MgCl_2$ leads to formation of xy-trimers and xy-tetramers, addition of connectors with low $MgCl_2$ leads to formation of z-pentamers, leaving the uninvolved moDONs monomeric. Addition of both leads to parallel assembly of all three structures. (**c**) TEM micrograph of parallelly assembled moDON superstructures: xy-connections are indicated in yellow, z-connections in turquois. In the TEM micrograph. Data of the same experiment is shown in Figure 4a. (**d**) Staticstial evaluation of structures formed in parallel assembly. Scale bar in (**c**) is 200 nm.

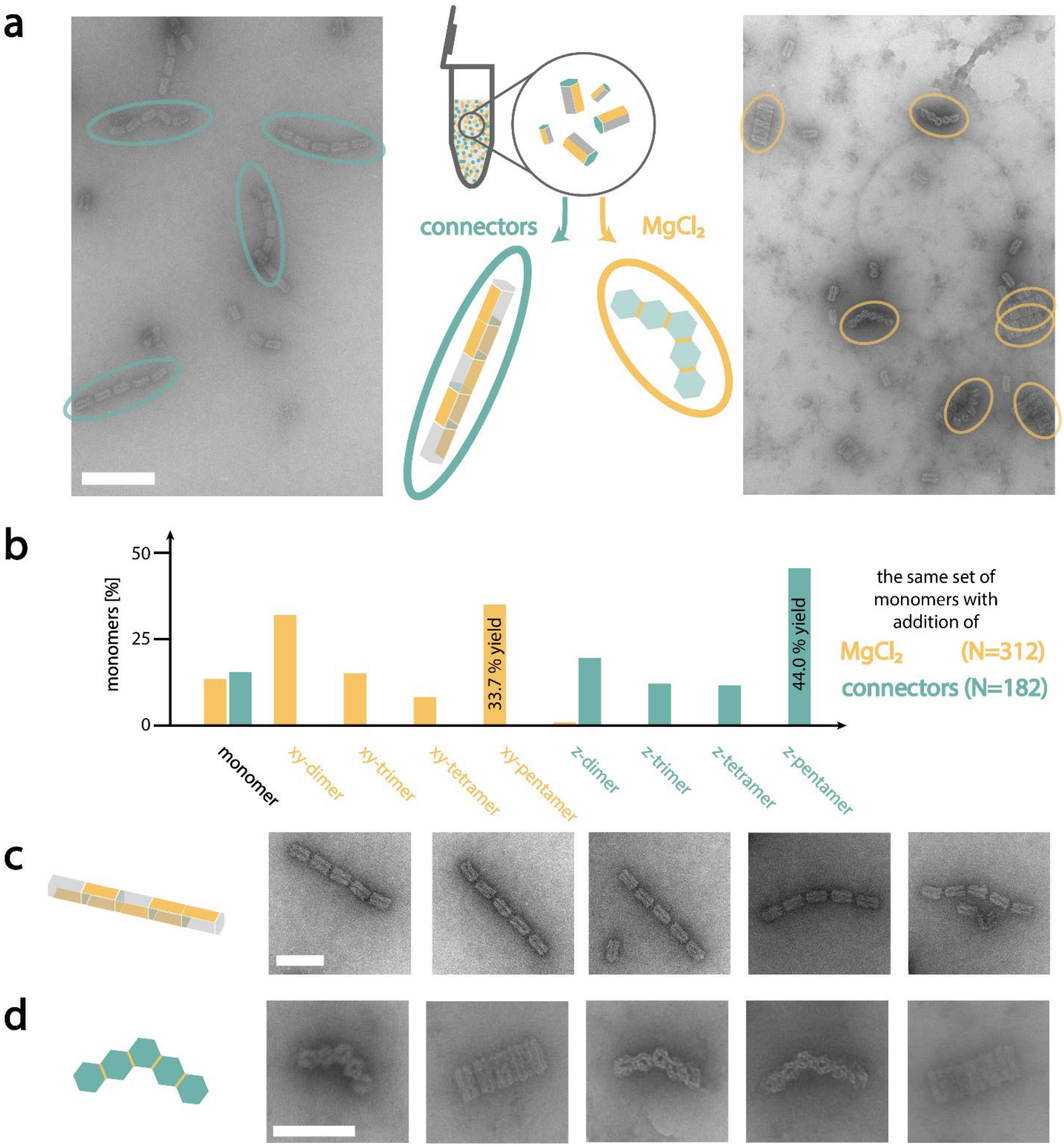


**Figure S57: Selective assembly** of different structures from the same set of moDONs: Schematic (**a**) shows that the resulting structure depends on whether z-connectors or $MgCl_2$ of 40 mM were added. xy-connections are indicated in yellow, z-connections in turquois. The moDONs used in this experiment are β*/zI-left/zII-right, βε*/zI-right, γε/zII-left/zIII-right, γ*ζ/zIV-left, and ζ/zIII-left/zIV-right. Data of the same experiment is shown in Figure 4b. Addition of only 40 mM $MgCl_2$ yields a pentamer in the xy-direction. Addition of only z-connectors yields a pentamer in z-direction. (**b**) statistical examination formed upon addition of different triggers to the same set of moDON monomers. (**c**) Close-up views of z-assembled pentamers, and (**d**) xy-assembled pentamers. Scale bar for (**a**) is 200 nm and holds for both micrographs, scale bar for (**c**) and (**d**) is 100 nm and holds for all micrographs of the respective structure.

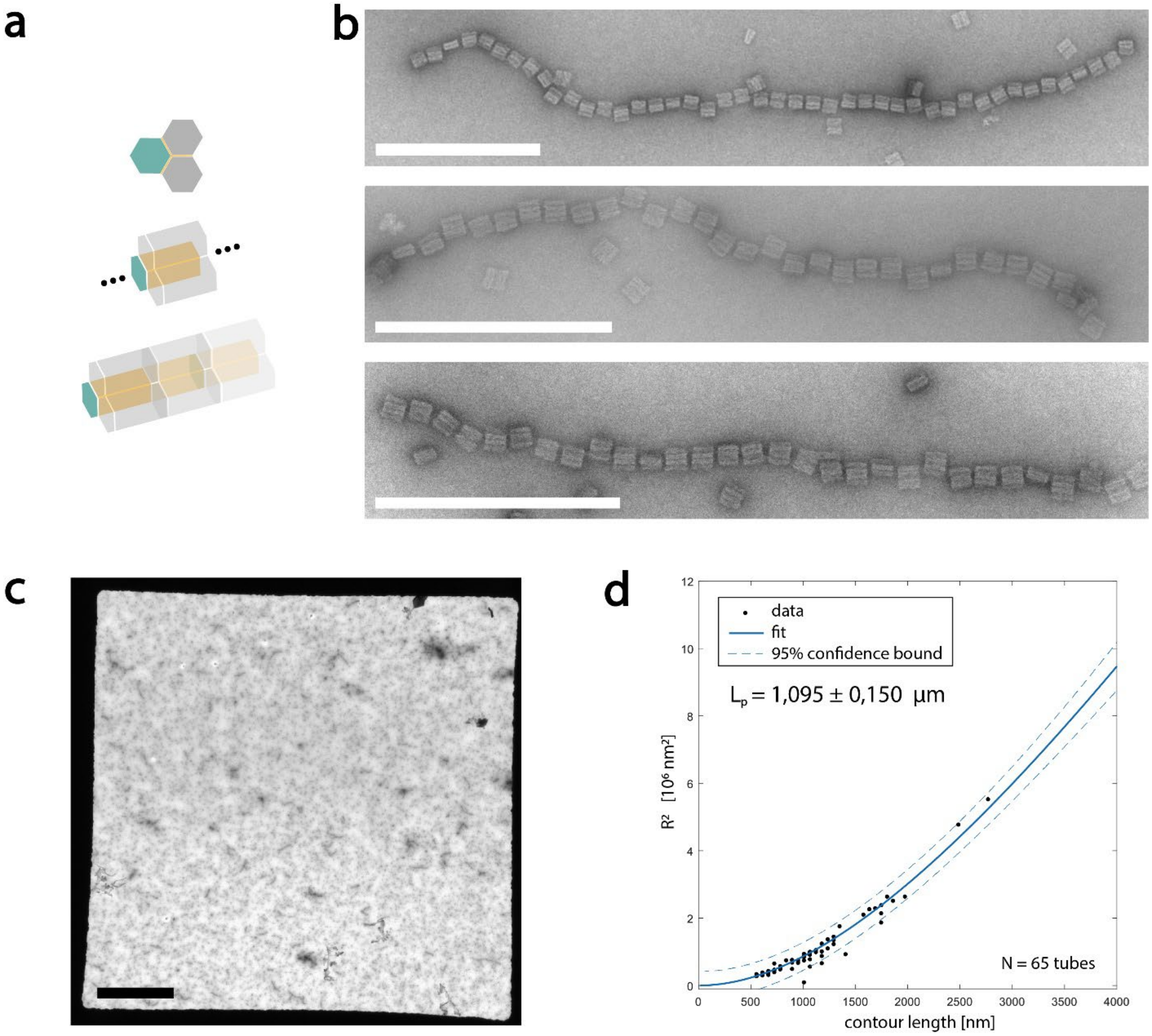


**Figure S58: Infinite tubes with trimeric subunits** are constructed with three moDONs: αβ*, βγ*, and α*γ/zI-left/zI-right and the repetitive subunit shown in (**a**). xy-connections are indicated in yellow, z-connections in turquois. (**b**) TEM micrographs of exemplary structures: Number of subunits (= monomers) is 44, 30, and 30 (from top to bottom), corresponding to approximately 2.5 µm, 1.7 µm, and 5.0 µm contour length, and 0.74 GDa, 0.51 GDa, and 0.51 GDa weight, respectively. (**c**) Wide-field TEM micrograph showing large periodic structure already visible at very low magnification. (**d**) Analysis of end-to-end distance (squared on y axis) and contour length (x axis) of N=65 structures revealed a persistence length of 1.095 ± 0.150 µm. Scale bars in (**b**) are 500 nm and in (**c**) 10 µm.

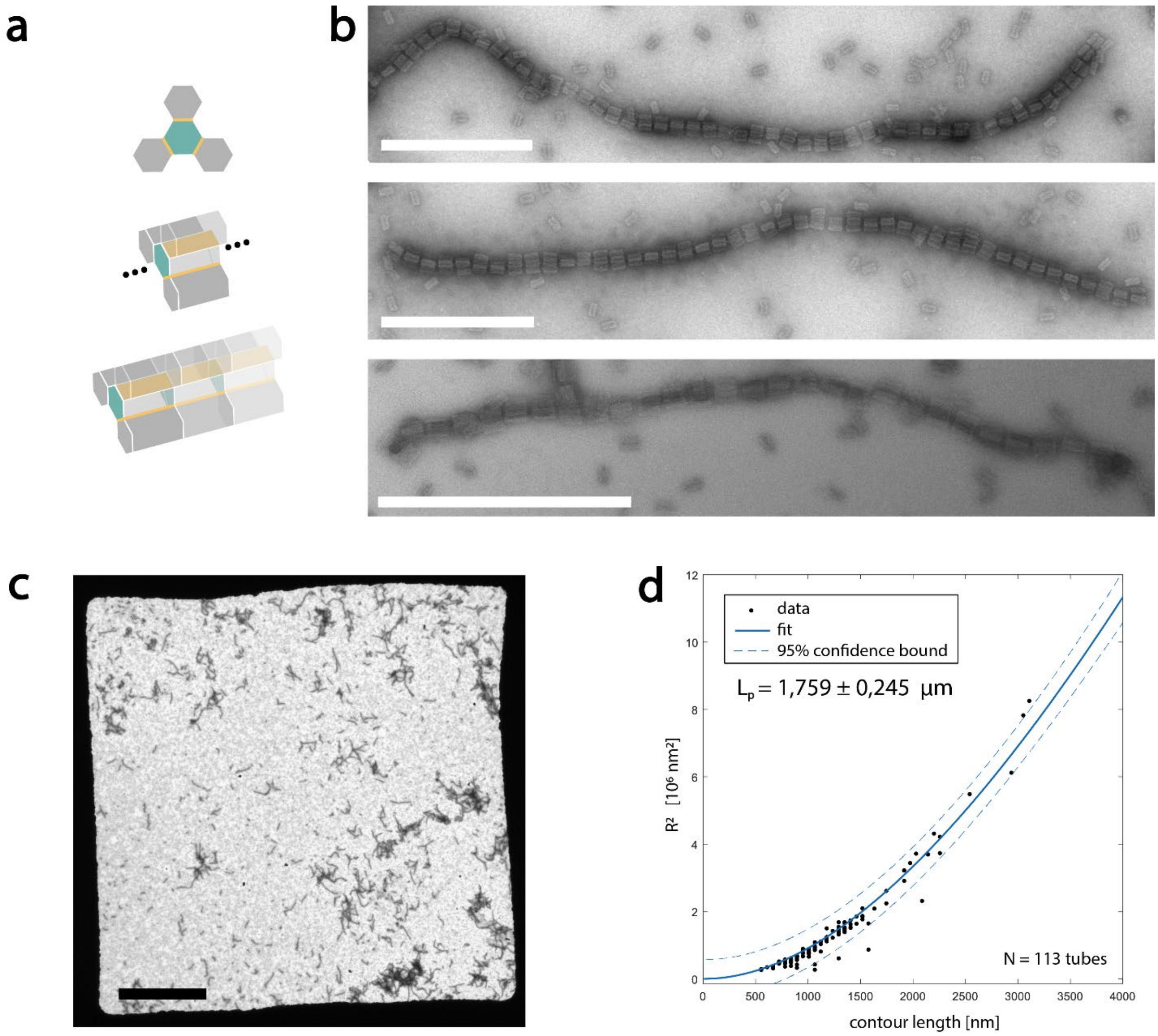


**Figure S59: Infinite tubes with tetrameric subunits** are constructed with four moDONs: δεζ/zII-left/zII-right, δ*, ε*, and ζ* and the repetitive subunit is shown in (a). xy-connections are indicated in yellow, z-connections in turquois. (**b**) TEM micrographs of exemplary structures: Number of subunits (= monomers) is 52, 45, and 28 (from top to bottom), corresponding to approximately 2.9 µm, 2.5 µm, and 1.6 µm contour length, and 1.17 GDa, 1.01 GDa, and 0.63 GDa weight, respectively. (**c**) Wide-field TEM micrograph showing large periodic structure already visible at very low magnification. (**d**) Analysis of end-to-end distance (squared on y axis) and contour length (x axis) of N=113 structures revealed a persistence length of 1.759 ± 0.245 µm. Data from the same experiment was also used in Figure 4c. Scale bars in (**b**) are 500 nm and in (**c**) 10 µm.

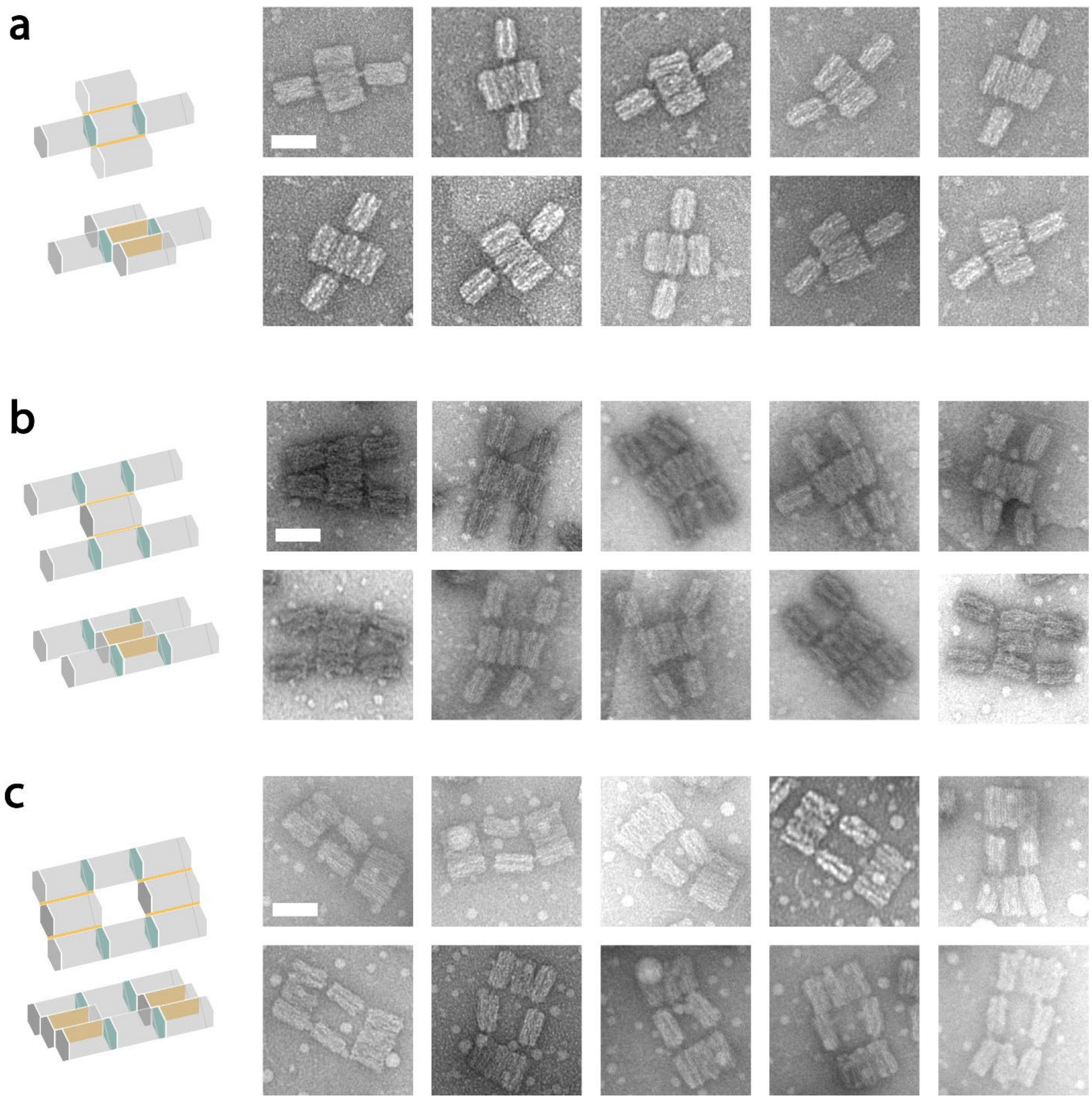


**Figure S60: close-up view on xyz-structures 1** showing TEM micrographs of (**a**) pentamers, (**b**) heptamers, and (**c**) octamers. Data from the same experiment was also used in Figure 4d. Scale bars are 50 nm and hold for all micrographs of the respective structure.

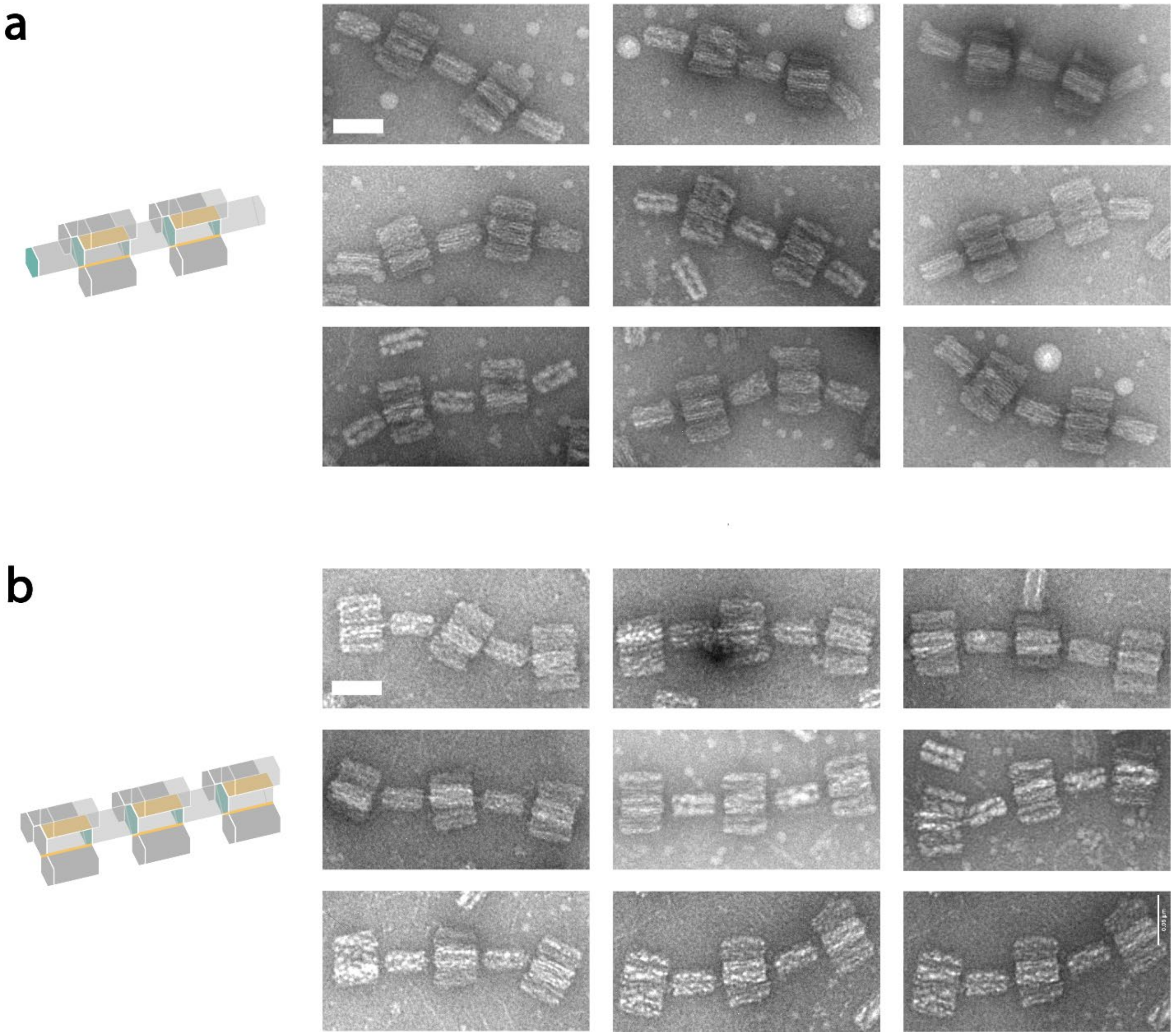


**Figure S61: close-up view on xyz-structures 2** showing TEM micrographs of (**a**) undecamers, and (**b**) tetradecamers. Data from the same experiment was also used in Figure 4d. Scale bars are 50 nm and hold for all micrographs of the respective structure.

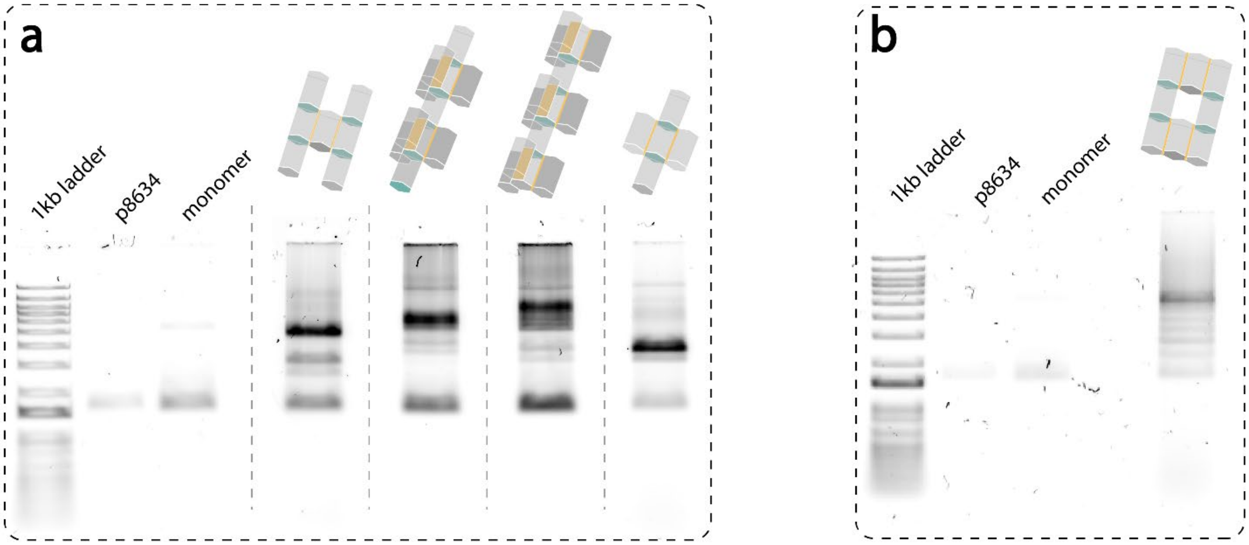


**Figure S62: AGE analysis of xyz-structures** shown in Figure 4d i-v. Increased structure sized yields proportionally less electrophoretic motility. From left to right: In (**a**) the heptamer shows a yield of 52.15 %, the undecamer a yield of 44.23 %, the 14mer a yield 17.31 %, and the pentamer a yield of 68.72 %. The octamer in (**b**) was assembled by gradually adding the connectors to the mixture and it yielded an assembly with 69.44 % yield.

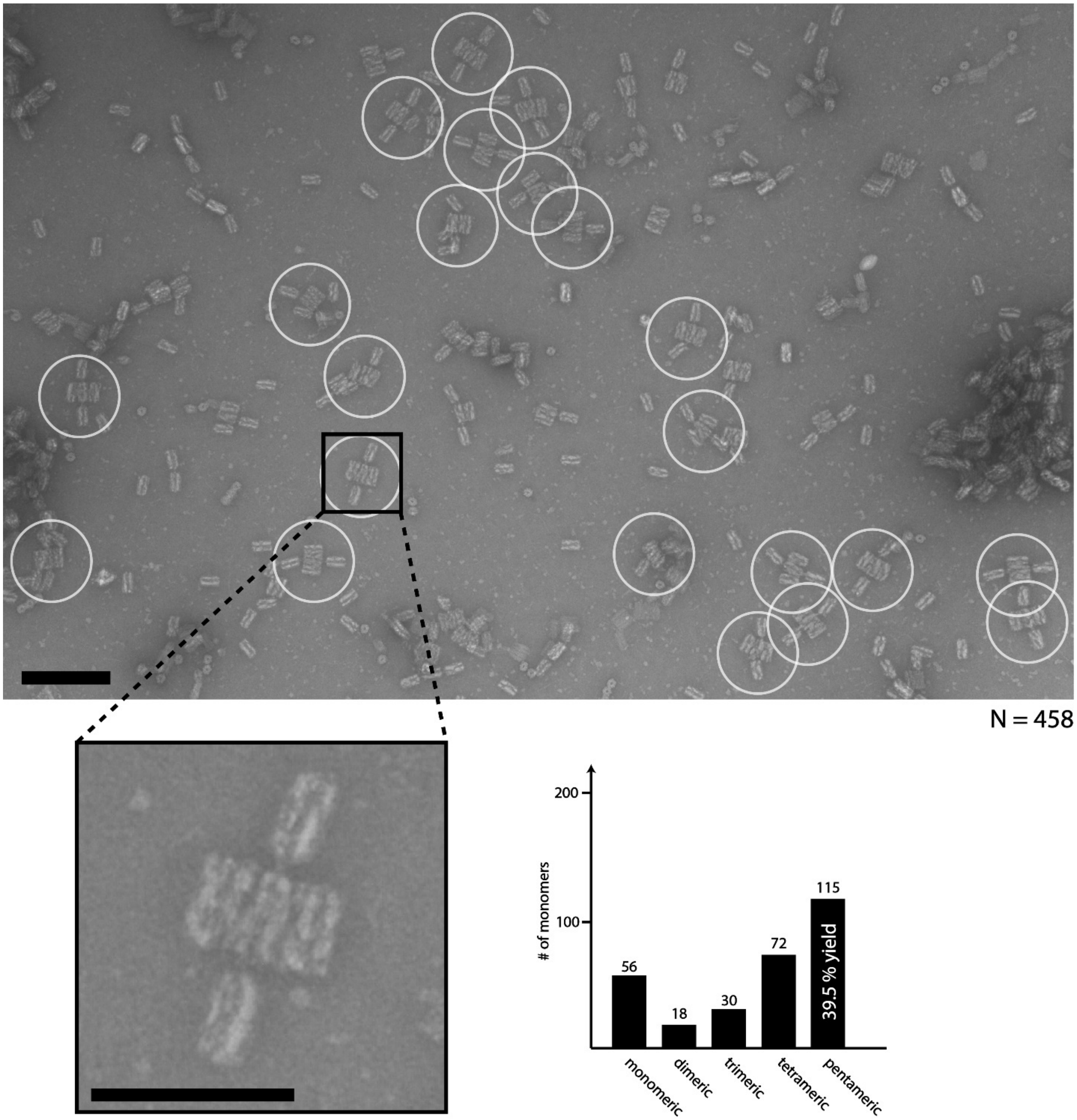


**Figure S63: Wide-field TEM micrograph and statistics of xyz-pentamer formation**. Analysis of the TEM micrograph shows a yield of 39.5 % of pentamers, as calculated by the fraction of monomers in the desired superstructure ($N_{pentamer}$ = 115) to the total amount of monomers ($N_{\Sigma}$ = 458). Scale bars are 200 nm.

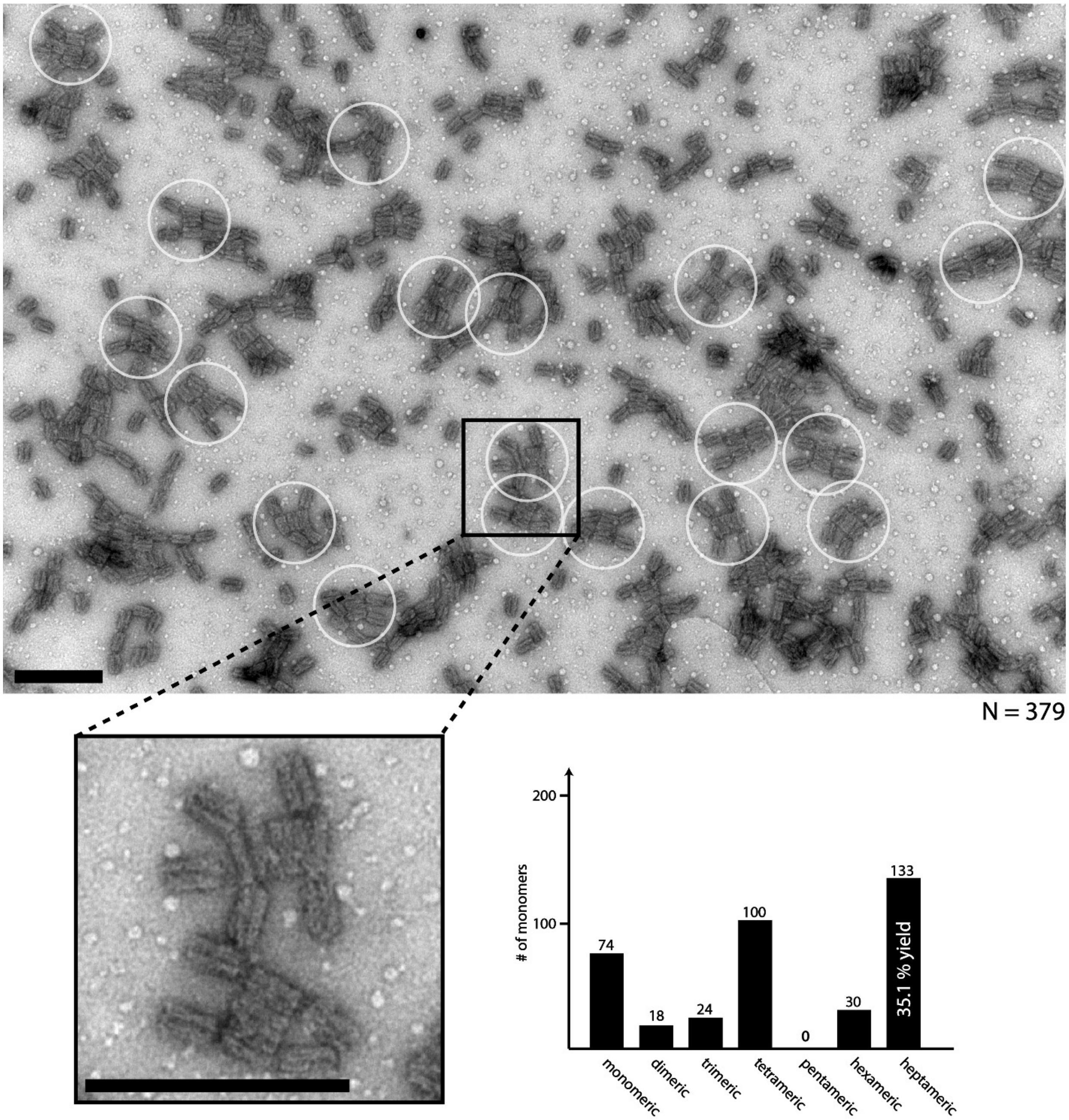


**Figure S64: Wide-field TEM micrograph and statistics of xyz-heptamer formation**. Analysis of the TEM micrograph shows a yield of 35.1 % of heptamers, as calculated by the fraction of monomers in the desired superstructure ($N_{heptamer}$ = 1133) to the total amount of monomers ($N_{\Sigma}$ = 379). Scale bars are 200 nm.

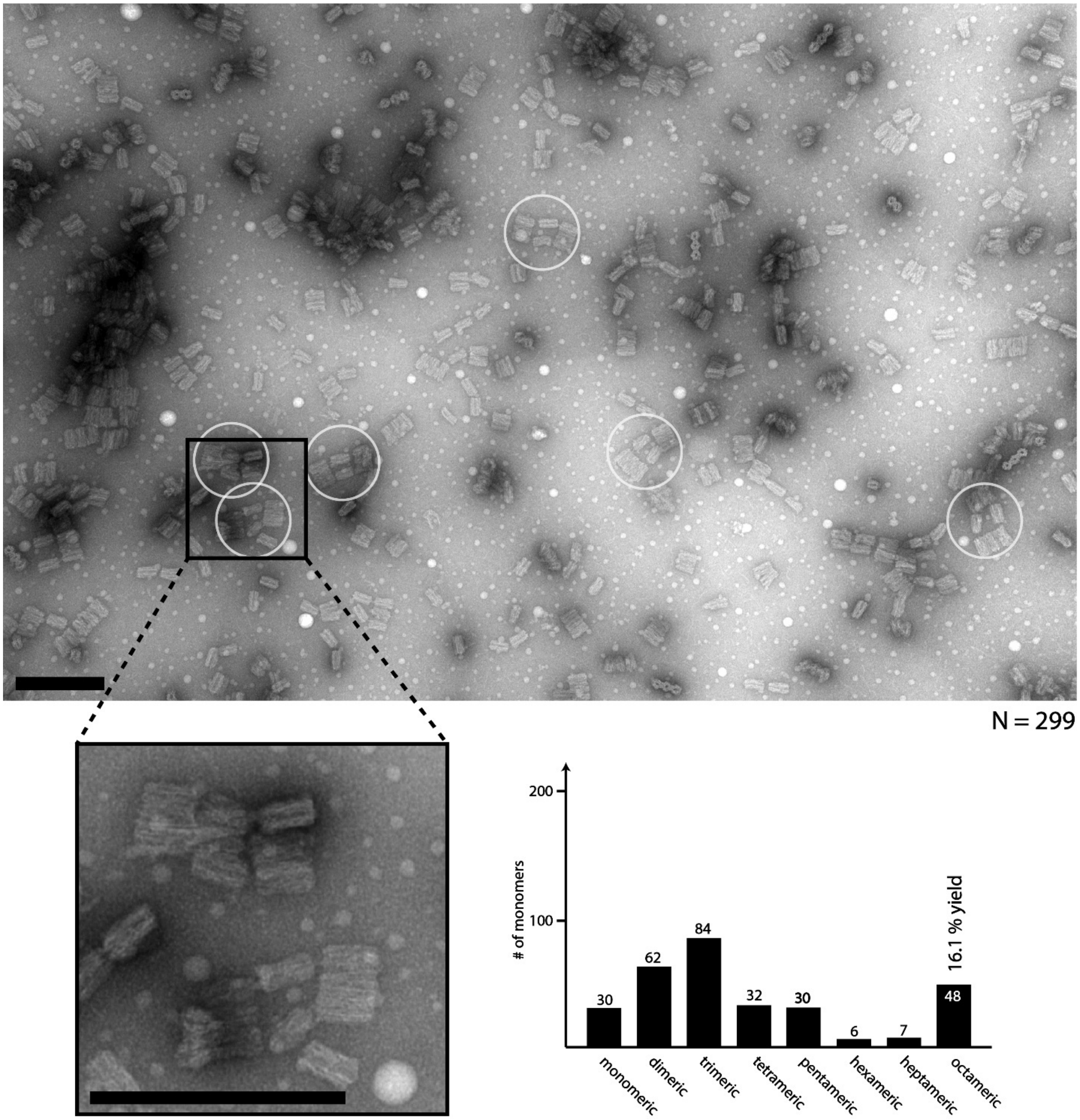


**Figure S65: Wide-field TEM micrograph and statistics of xyz-octamer formation**. Analysis of the TEM micrograph shows a yield of 16.1 % of octamers, as calculated by the fraction of monomers in the desired superstructure ($N_{octaamer}$ = 48) to the total amount of monomers ($N_{\Sigma}$ = 299). Scale bars are 200 nm.

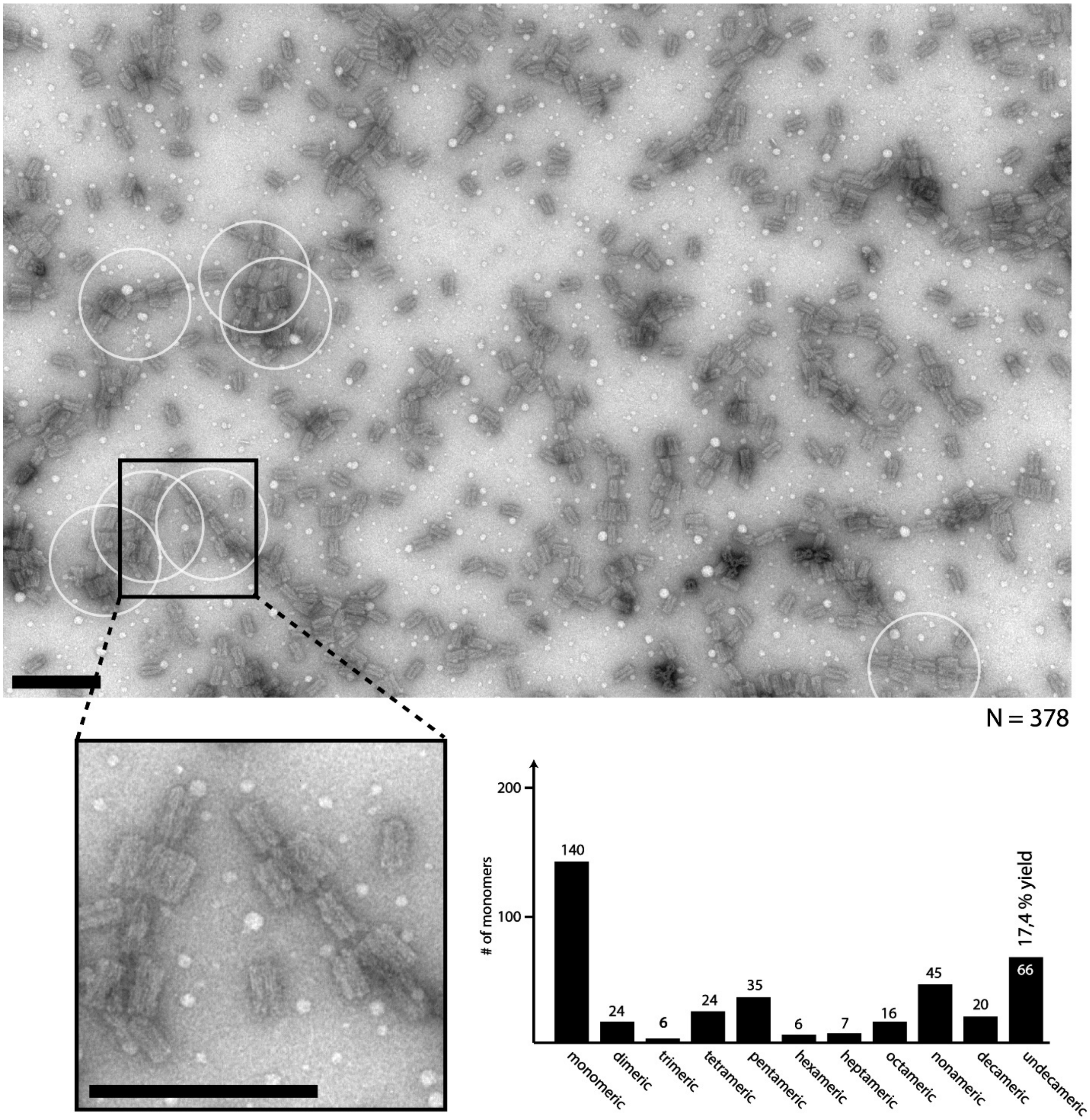


**Figure S66: Wide-field TEM micrograph and statistics of xyz-undecamer formation**. Analysis of the TEM micrograph shows a yield of 17.4 % of undecamers, as calculated by the fraction of monomers in the desired superstructure ($N_{undecamer}$ = 66) to the total amount of monomers ($N_{\Sigma}$ = 378). Scale bars are 200 nm.

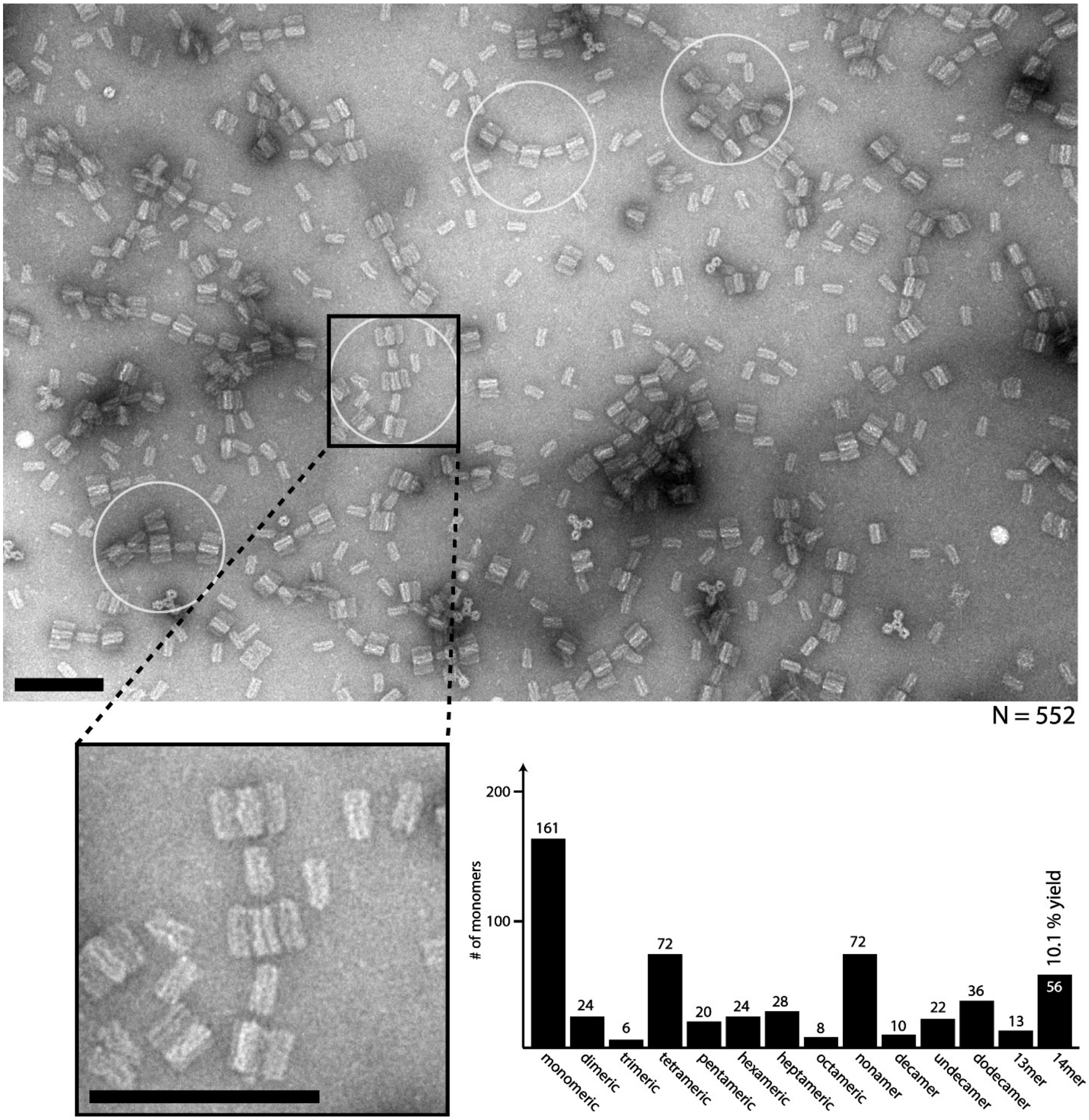


**Figure S67: Wide-field TEM micrograph and statistics of xyz-14mer formation**. Analysis of the TEM micrograph shows a yield of 10.1 % of 14mers, as calculated by the fraction of monomers in the desired superstructure ($N_{14mer}$ = 56) to the total amount of monomers ($N_{\Sigma}$ = 552). Scale bars are 200 nm.

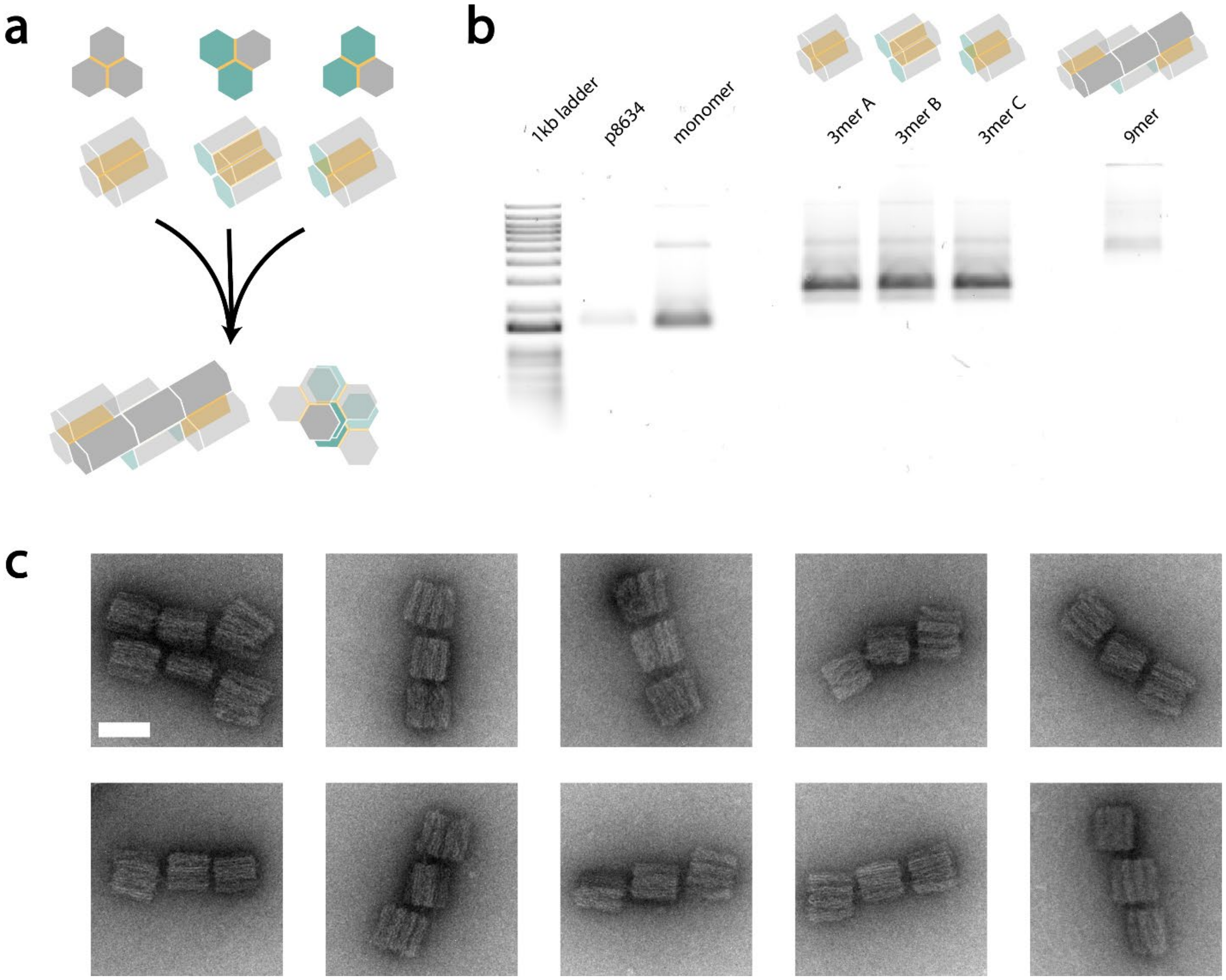


**Figure S68: hierachical xyz-nonamer "twisted trimeric trimer" assembly.** (**a**) Schematic of assembly strategy: three trimers are assembled independently, and then mixed equimolar to assemble a nonamer without intermediate purification. (**b**) AGE analysis of the hierachical assembly. Each trimer assembles with a yield of just below 90 %, the nonamer assembles with a yield of 68.51 %, which is approximately 0.9^3. (**c)** close-up view on **t**he formed nonamers. Scale bar is 50 nm and holds for all micrographs.

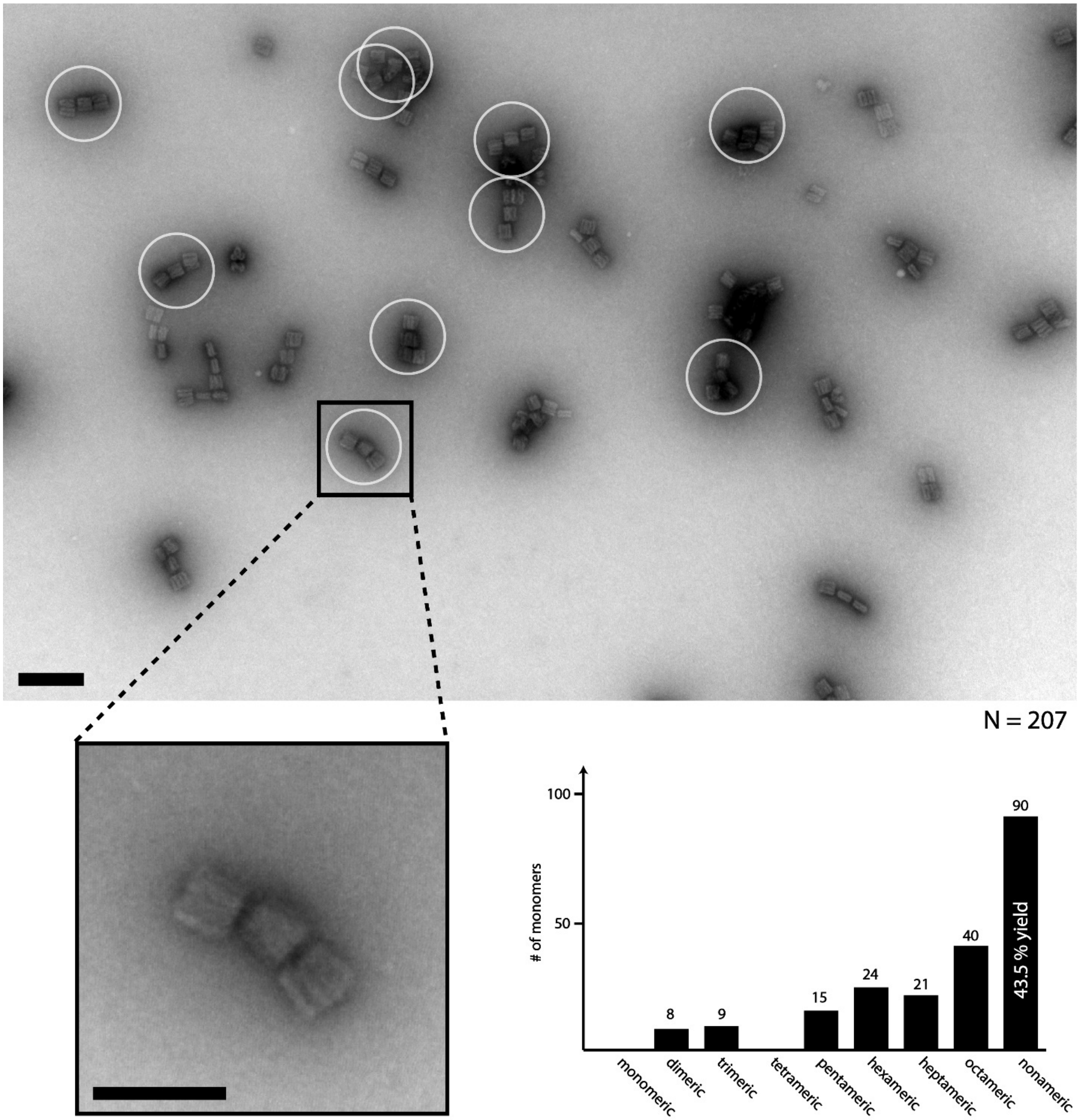


**Figure S69: Wide-field TEM micrograph and statistics of xyz-nonamer "twisted trimeric trimer" formation**. Analysis of the TEM micrograph shows a yield of 43.5 % of nonamers, as calculated by the fraction of monomers in the desired superstructure ($N_{nonamer}$ = 90) to the total amount of monomers ($N_{\Sigma}$ = 207). Scale bars are 200 nm.

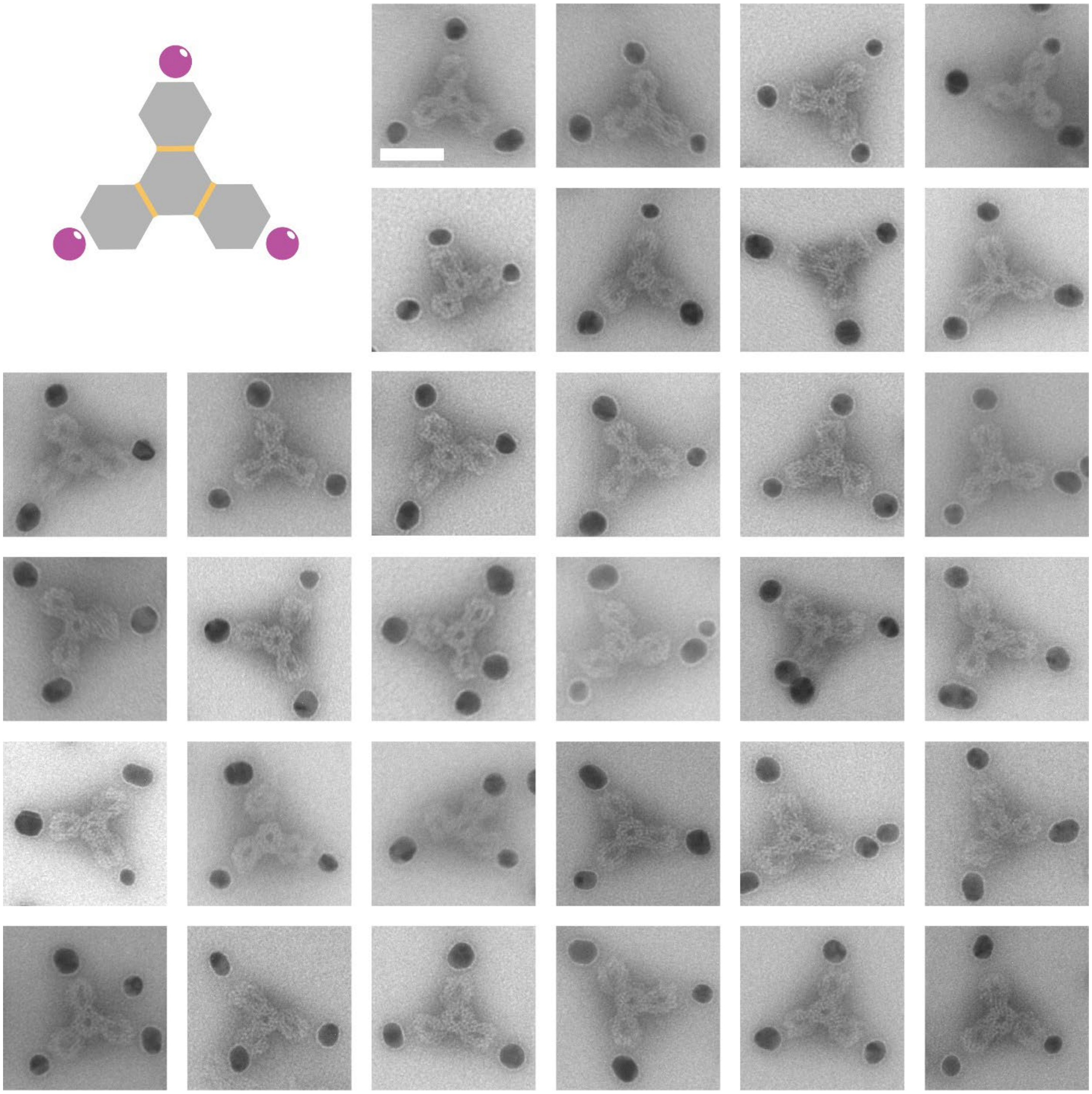

**Figure S70: xy-tetramer with selectively placed Au NPs** at the vertices. The central moDON has the connection sites δεζ, connecting to monomers δ* (Au NP handles in α direction), ε* (Au NP handles in β direction), and ζ* (Au NP handles in γ direction). Data of the same experiment is shown in Figure 4e. Scale bar is 50 nm and holds for all micrographs.

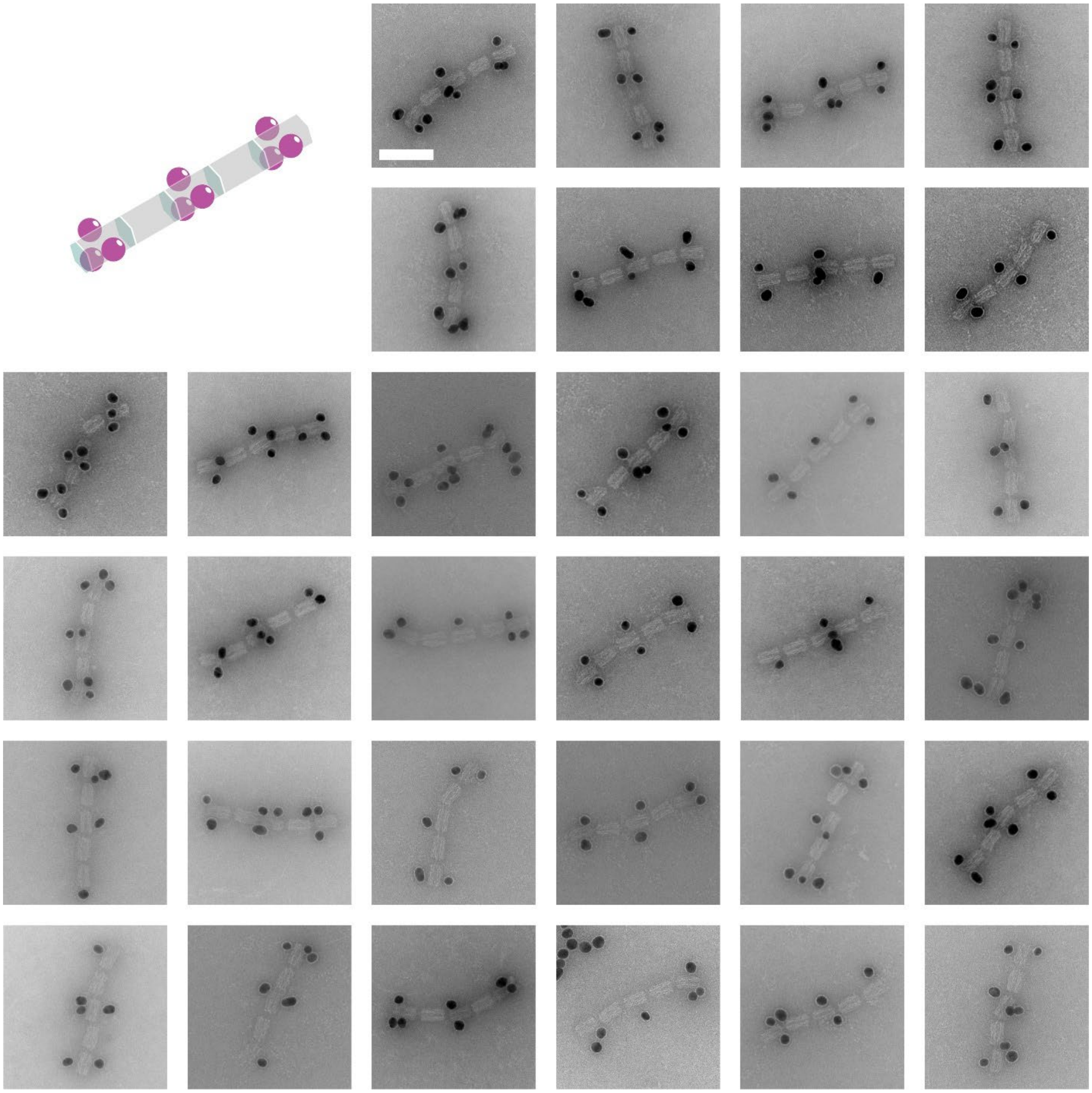

**Figure S71: z-pentamer with selectively placed Au NPs** on monomers one (zI-right), three (zII-left/zIII-right), and five (zIV-left) with three Au NP connection sites (modified αβγ connection sites). Data of the same experiment is also found in Figure 4e. Scale bar is 100 nm and holds or all micrographs.

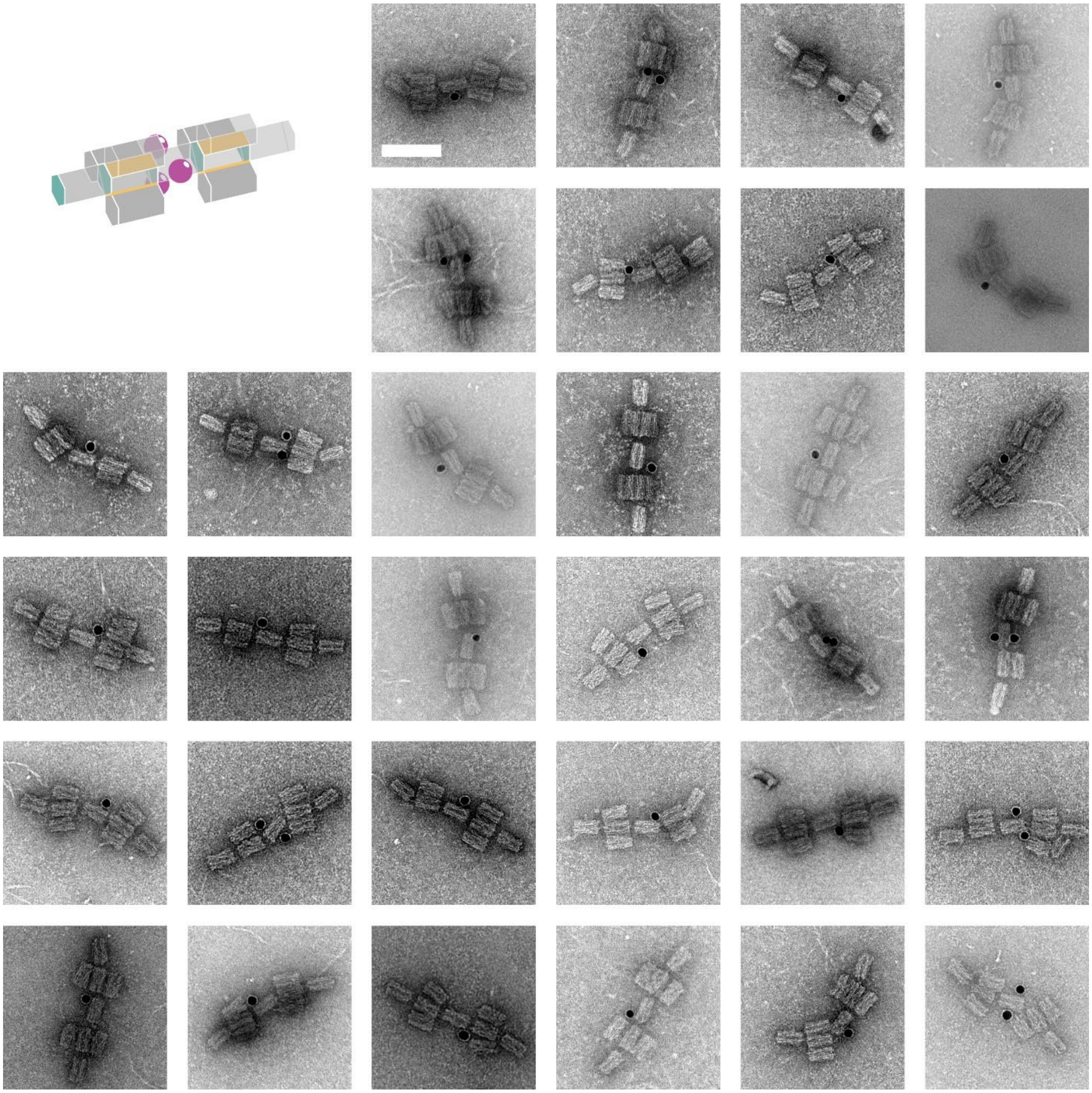

**Figure S72: xyz-undecamer with selectively placed Au NPs** on the middle monomers (zIIleft-zIIIright) with three Au NP connection sites (modified αβγ connection sites). Data of the same experiment is also found in Figure 4e. Scale bar is 100 nm and holds or all micrographs.

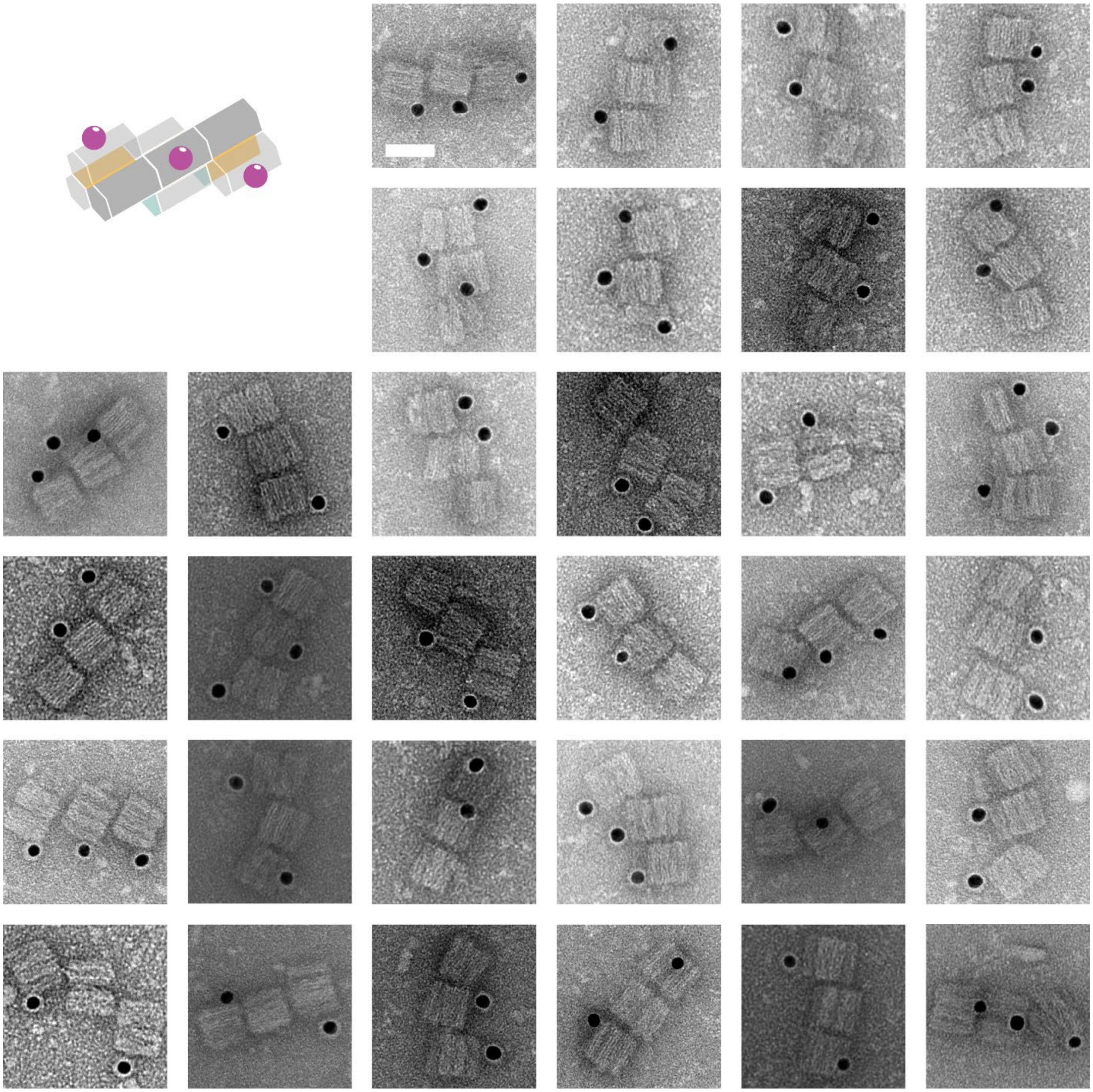

**Figure S73: xyz-nonamer "twisted trimeric trimer" with selectively placed Au NPs** on one monomer in each of the trimer assemblies. Data of the same experiment is also found in Figure 4e. Scale bar is 100 nm and holds or all micrographs.

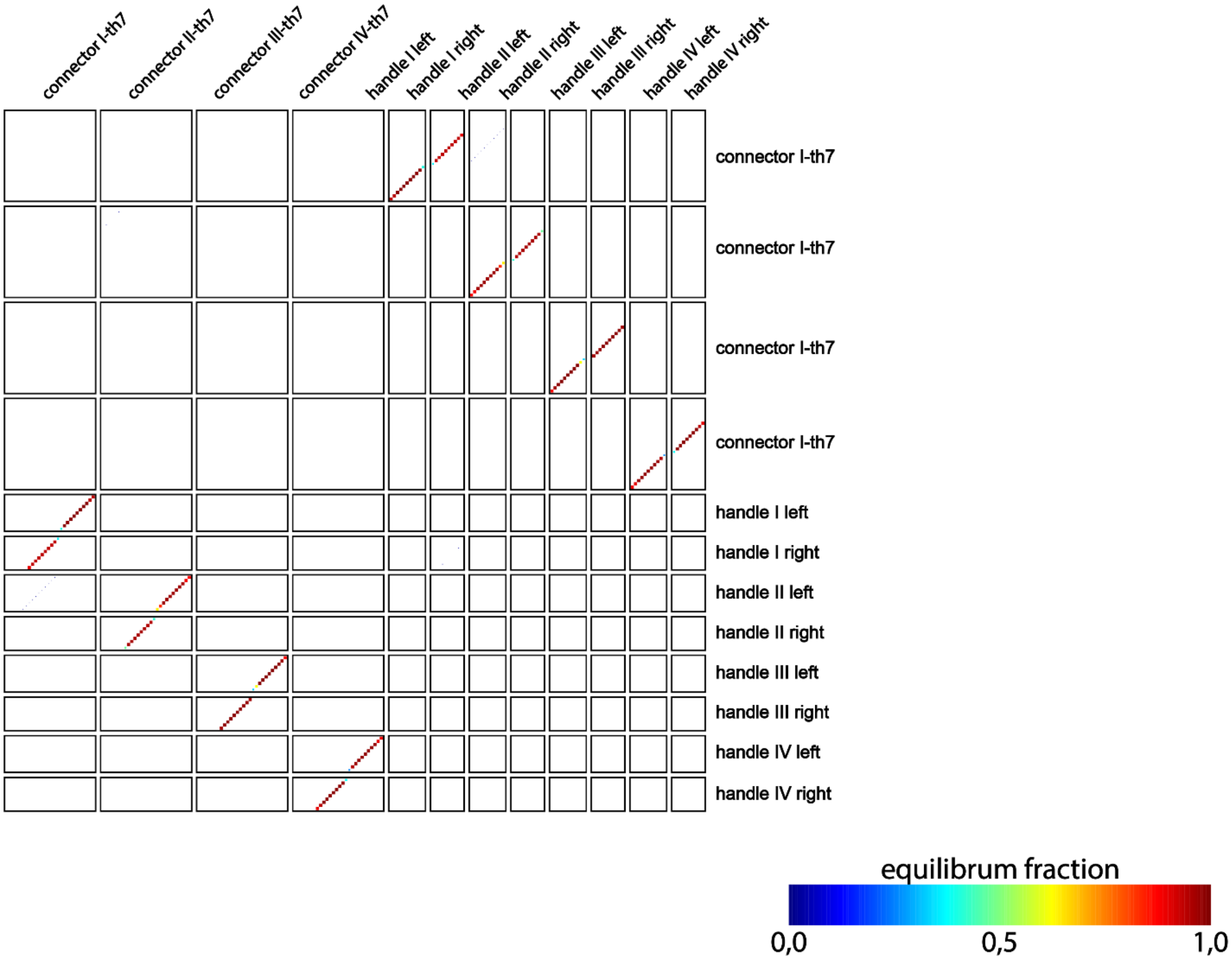


**Figure S74: NUPACK analysis of z-connector orthogonality with toeholds:** color scheme indicates complementary connectors and handles bind strongly and selectively with each other even in presence of the other handles and connectors. This shows mutual orthogonality. Connectors elongated with a toehold region were denoted with the respective Roman letter, indicating their sequence, and "-th7", indicating the addition of a 7 nt long toehold. The toehold does not seem to have a major influence on binding behavior (*cf*. Figure S28). The simulation was conducted with NUPACK version 2.2 and the following options: 20°C, max. 3 strand complexes, 1 µM per strand, "Serra and Turner 1995", 1 M NaCl. For respective sequences see Table S15.

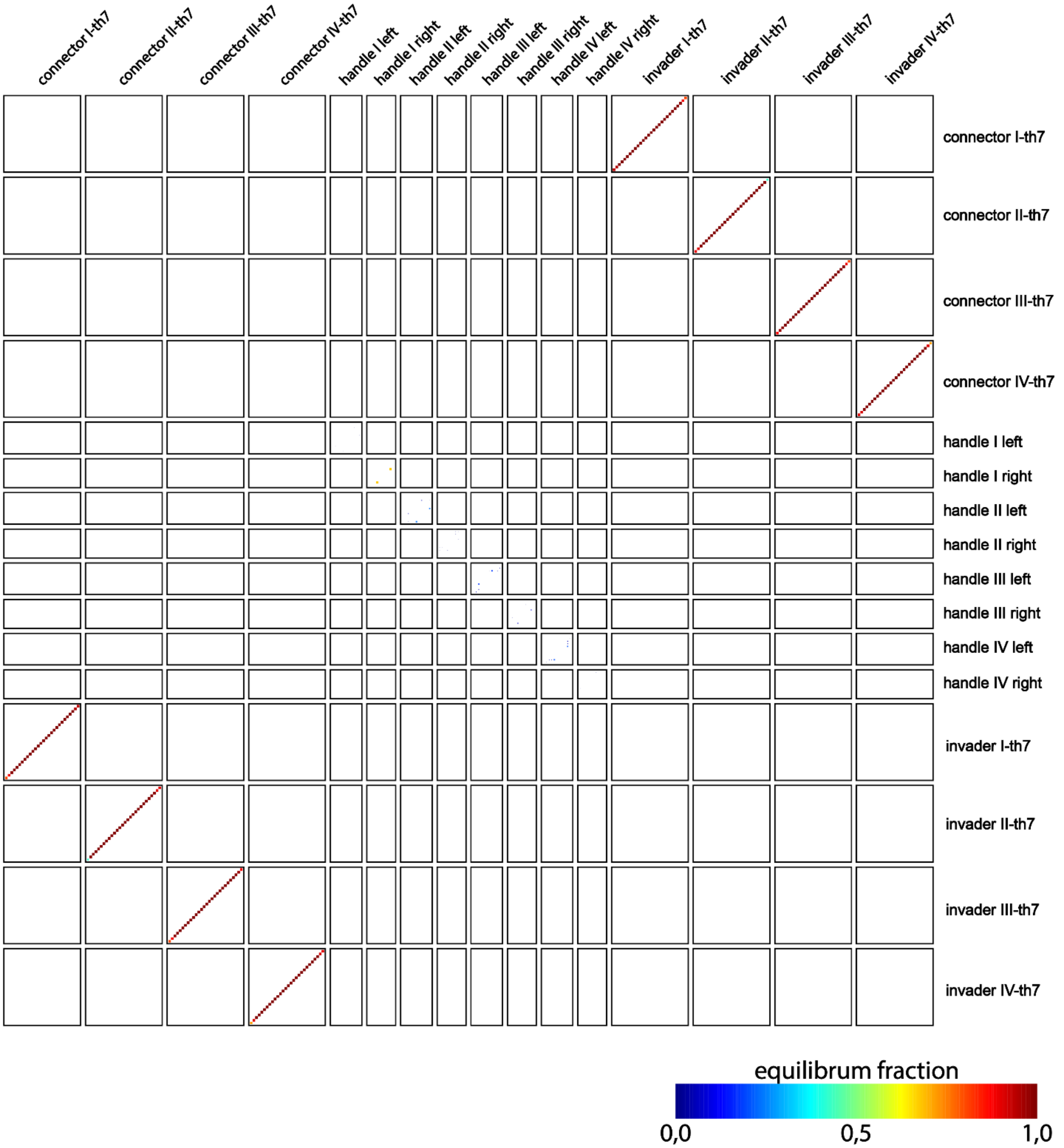


**Figure S75: NUPACK analysis of z-connectors in presence of invader strands:** color scheme indicates the binding of complementary connectors and invaders is favored over the binding of connectors to handles (*cf.* Figure S48). Further, crosstalk between non-complementary handles, connectors and invaders is vanishingly low, indicating retained orthogonality in the strand displacement process. Connectors and invaders elongated with a toehold region were denoted with their respective Roman letter, indicating their sequence, and "-th7", indicating a 7 nt long toehold. The simulation was conducted with NUPACK version 2.2 and the following options: 20°C, max. 3 strand complexes, 1 µM per strand, "Serra and Turner 1995", 1 M NaCl. For respective sequences see Table S15.

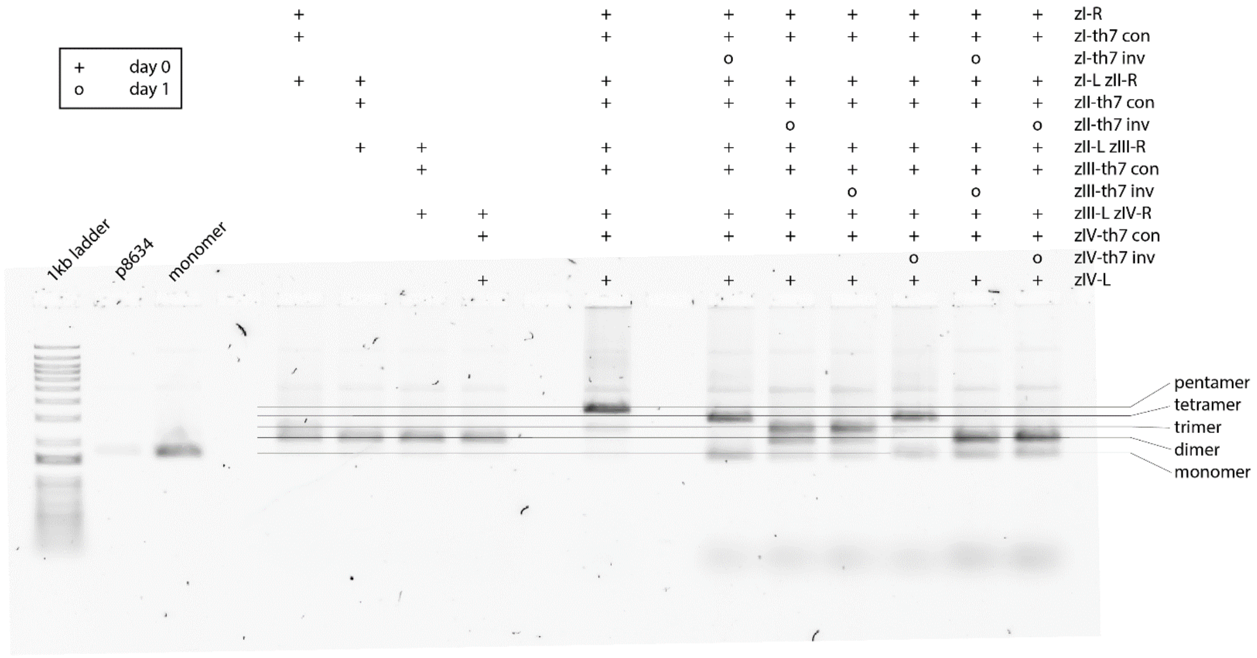


**Figure S76: z-disassembly permutations** of a pentamer, which was formed with 5-fold connector excess over handles over-night. Connectors and invaders elongated with a toehold region were denoted with their respective Roman letter, indicating their sequence, and "-th7" indicating the addition of a 7 nt long toehold. For sequences see Table S15. Invaders were added in 5-fold excess over connectors and again incubated over-night. Addition of invaders against connection zI or zIV resulted in disassembly to a tetramer and a monomer (see Figure S51), addition of zII or zIII invaders resulted in a dimer and a trimer (see Figure S52). Combination of zI and zIII invaders or zII and zIV invaders resulted in 2 dimers and one monomer (see Figure S53). Used moDON monomers: zI-right, zI-left/zII-right, zII-left/zIII-right, zIII-left/zIV-right, and zIV-left. Data from the same experiment was used in Figure 5b.

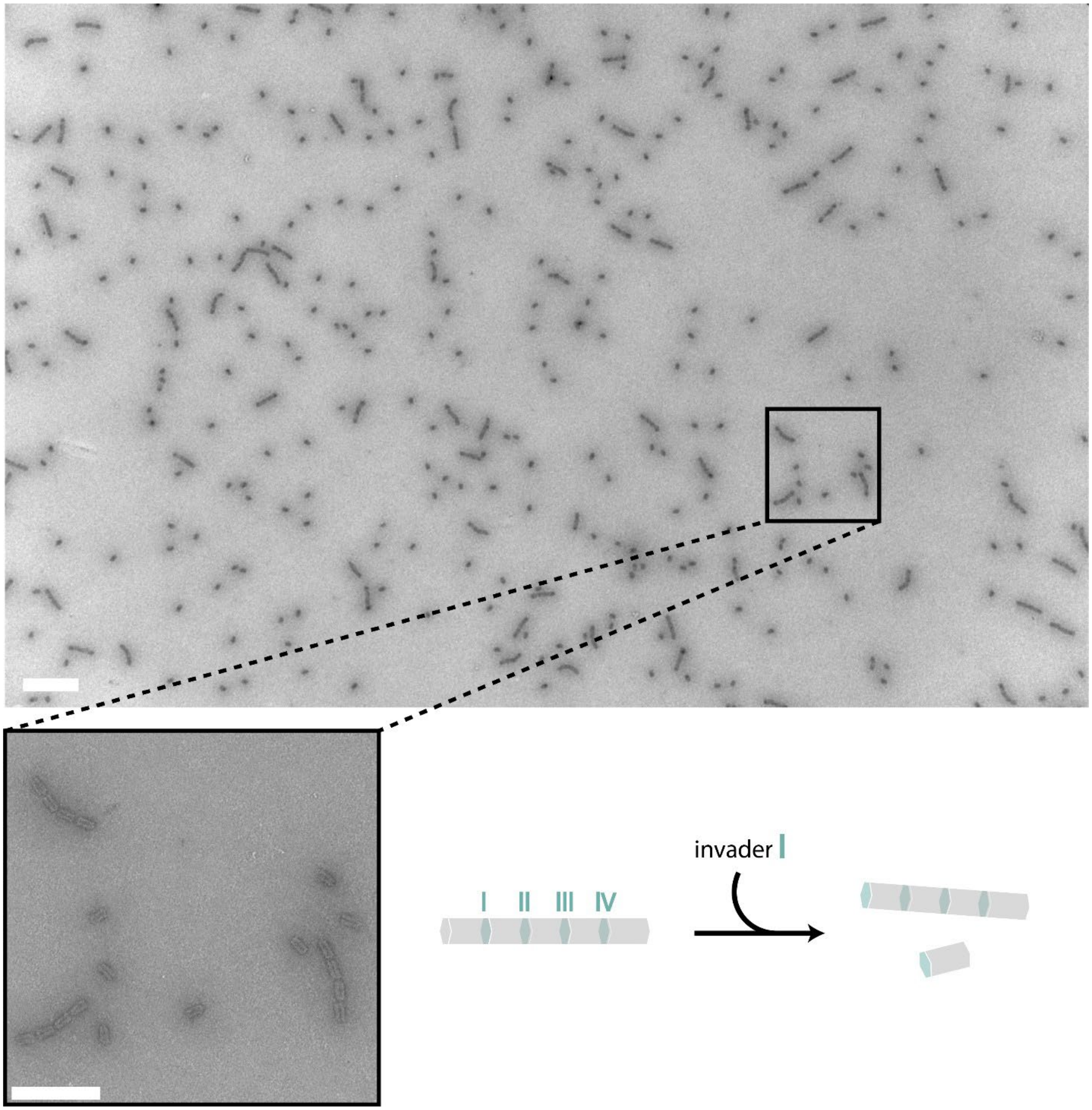


**Figure S77: TEM micrographs of selective z-disassembly 1:** To a fully formed pentamer of z-connections, invader strand I was added. Invader strand I disassembled the connections between the first and second moDON (from left to right) which resulted in a tetramer and a monomer. Scale bar is 500 nm for the overview picture and 200 nm for the enlarged part. Data from the same experiment was used in Figure 5b.

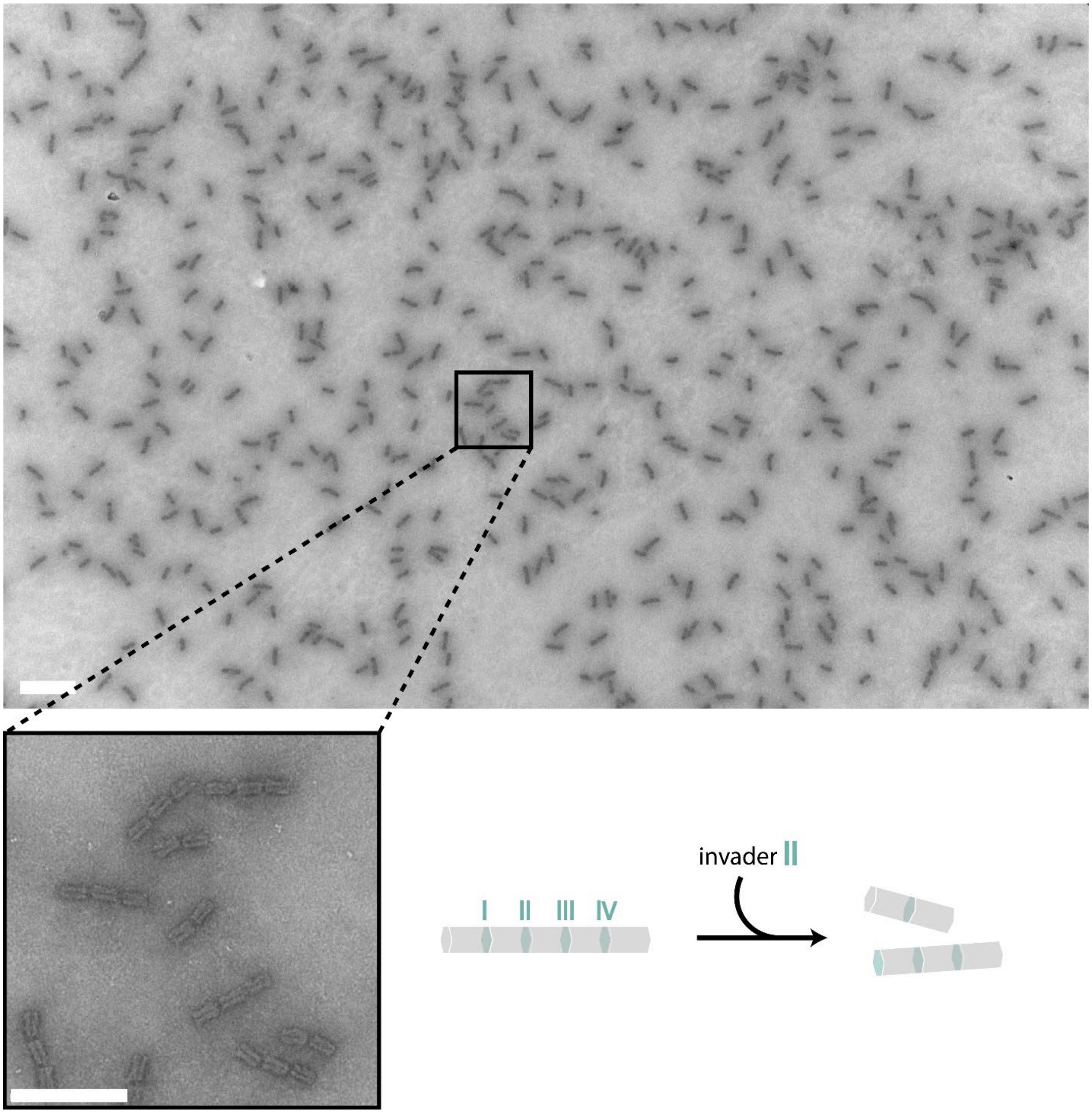


**Figure S78*: TEM micrographs of selective z-disassembly 2:*** To a fully formed pentamer of z-connections invader strand II was added. Invader strand II disassembled the connections between the second and third (from left to right) moDON, which resulted in dimers and trimers. Scale bars are 500 nm for the overview picture and 200 nm for the enlarged part. Data from the same experiment was used in Figure 5b.

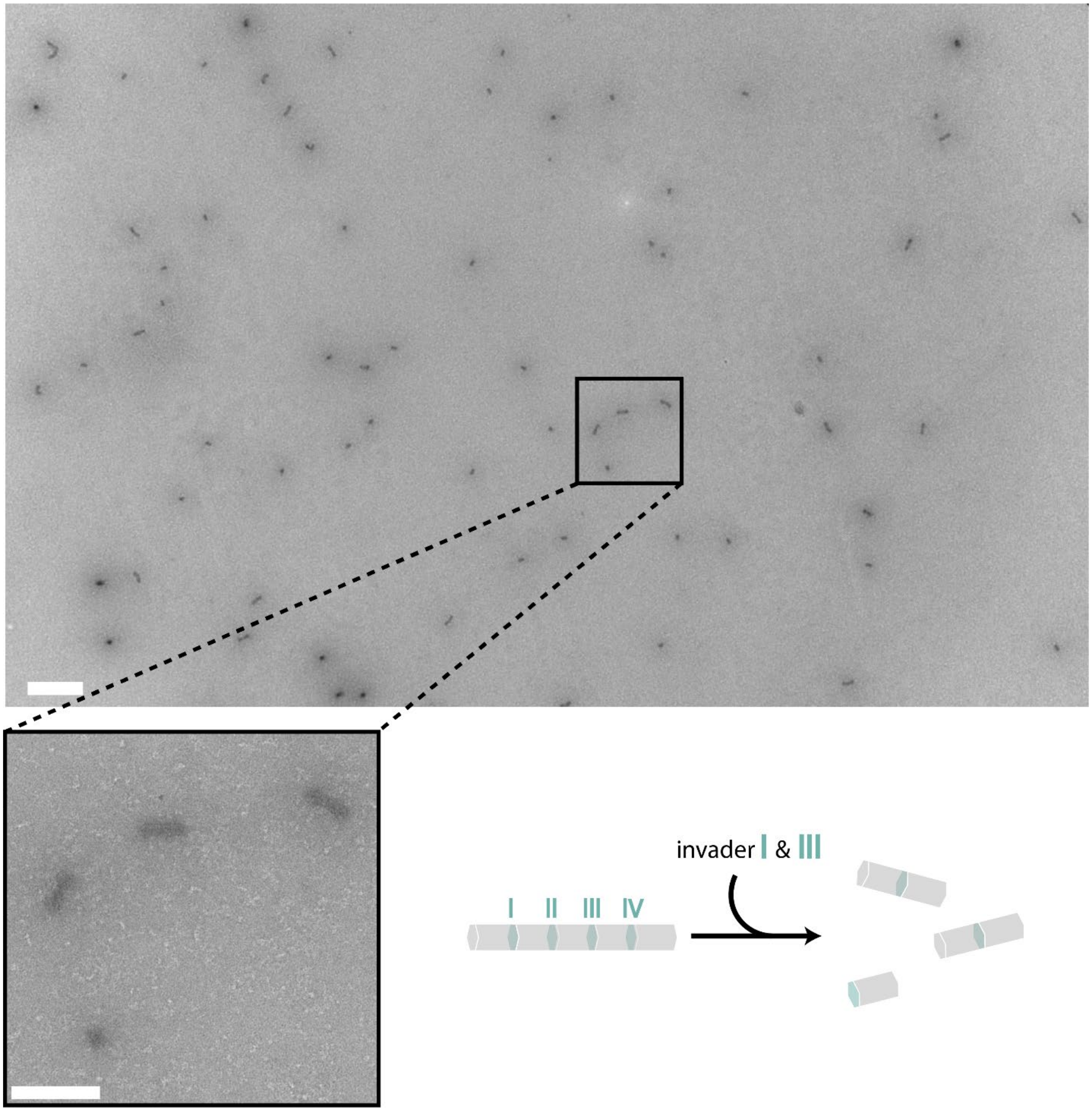


**Figure S79: TEM micrographs of selective z-disassembly 3:** To a fully formed pentamer of z-connections invader strands I and III were added. Invader strand I and III disassembled the connections between the first and second, as well as the third and fourth moDON (from left to right) which resulted in two dimers and one monomer, per pentamer. Scale bars are 500 nm for the overview picture and 200 nm for the enlarged part.

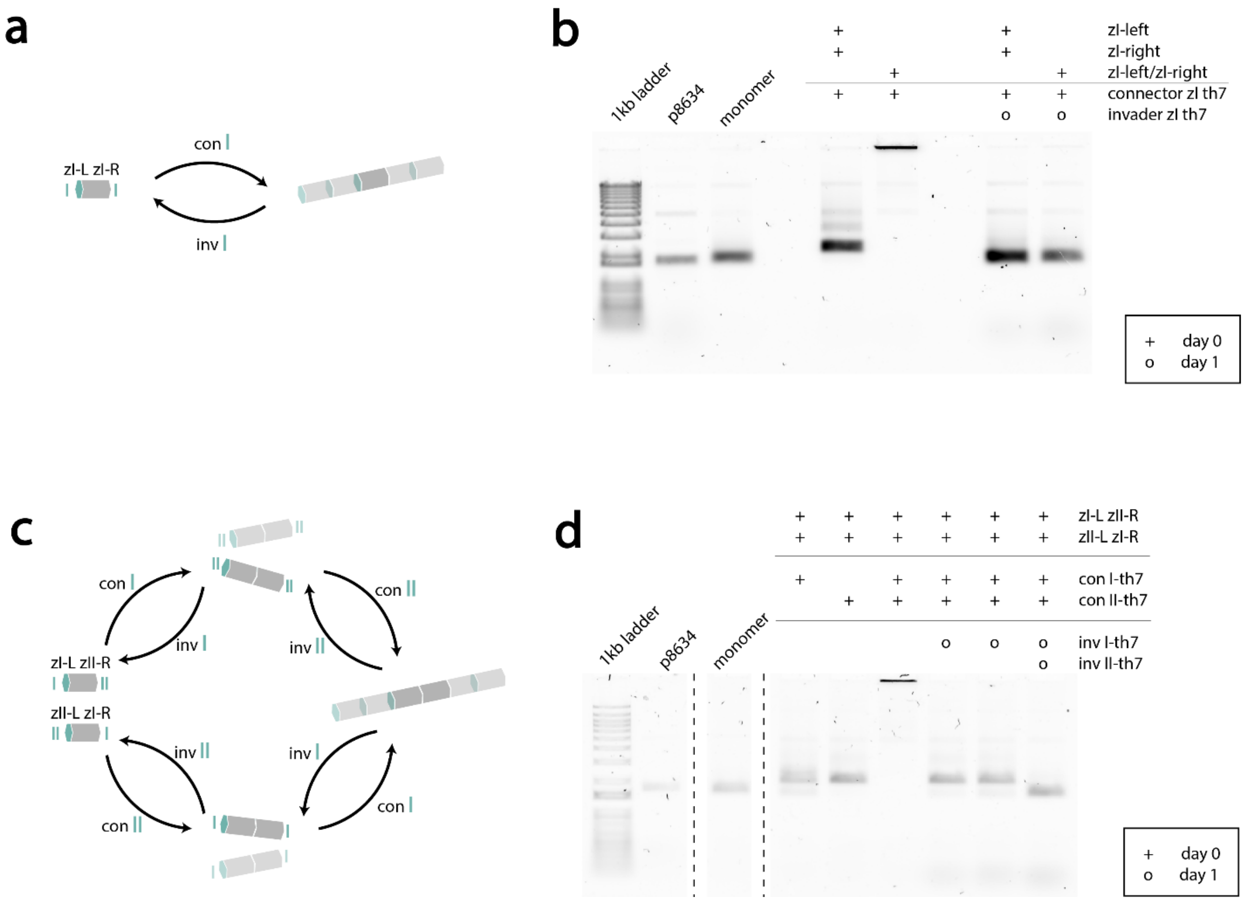


**Figure S80: Assembly & disassembly of periodic z-directional superstructures**: (**a**), (**b**) Homomultimeric or (**c**), (**d**) heteromultimeric. (**a**) Schematic showing assembly and disassembly pathway of the homomultimeric superstructure. (**b**) AGE shift assay of the assembly and disassembly: Addition of connector strand assembles the monomers into infinite superstructures, addition of the respective invader disassembles them again into monomers. (**c**) Schematic showing assembly and disassembly pathways of the heteromultimeric superstructure. (**d**) AGE shift assay shows assembly of both possible subunits through addition of the respective connector strand, and assembly of infinite tubes, when both connectors are added. The tubes can be disassembled into dimers or monomers, depending on whether only one or both invaders are added. Gels were imaged on day 2.

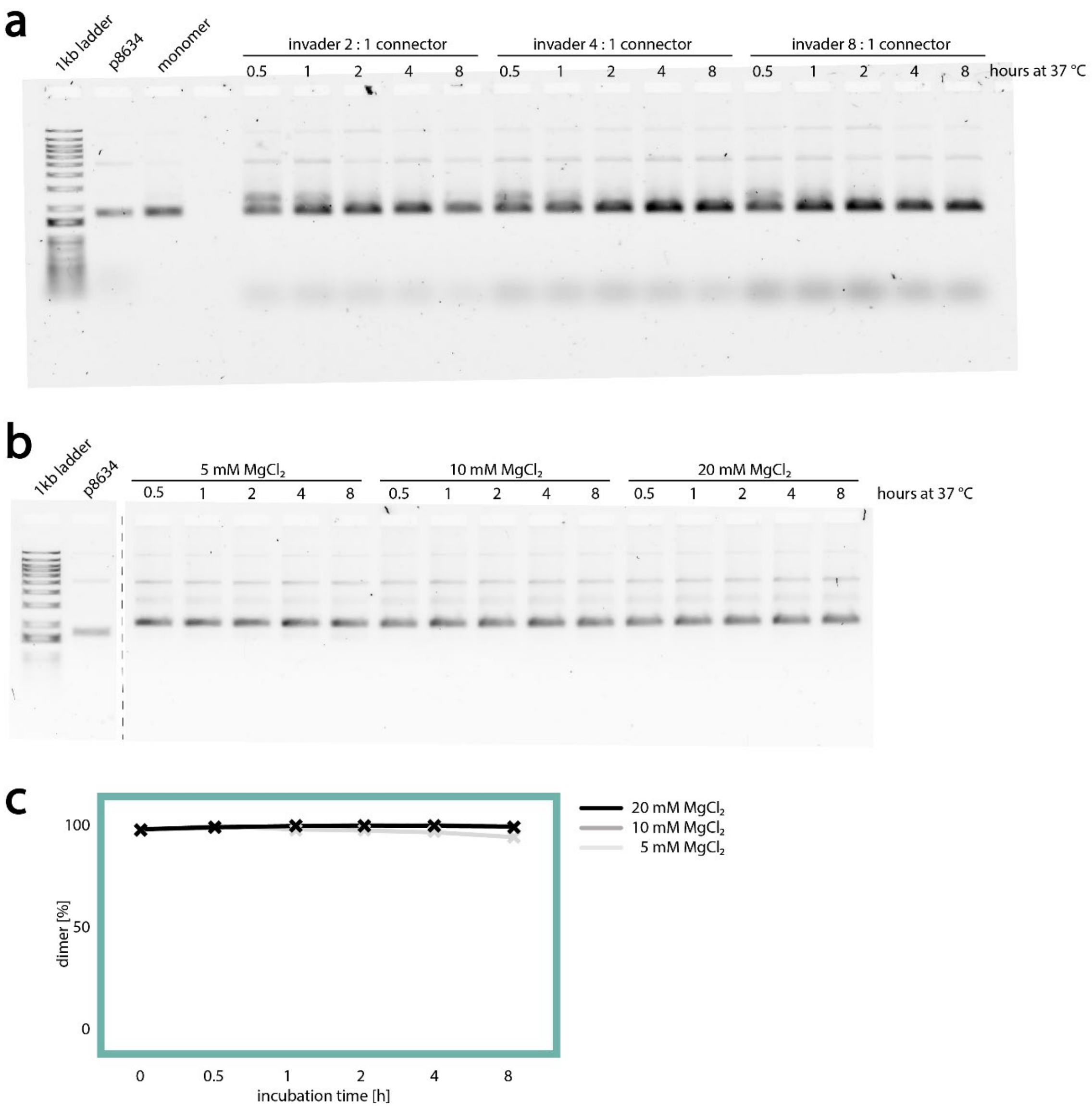


**Figure S81: AGE shift assay of z-disassembly:** (**a**) Influence of invader excess: z-directional dimers were formed through overnight, the next day invaders were added in 2-, 4-, or 8-fold excess over connector strands, each 8, 4, 2, 1, or 0.5 h before the samples were transferred to the gel. (**b**) Influence of $MgCl_2$ concentration: z-directional dimers were formed over-night. They were diluted in buffers with a total $MgCl_2$ concentration of 5 mM, 10 mM, or 20 mM each, 8, 4, 2, 1, or 0.5 h before the samples were transferred to the gel. (**c**) $MgCl_2$ dependency of dimer disassembly (based on analysis of gel band intensities: fraction of dimer band intensity to the sum of dimer and monomer band intensity). 100% dimerization normalized to monomer band.

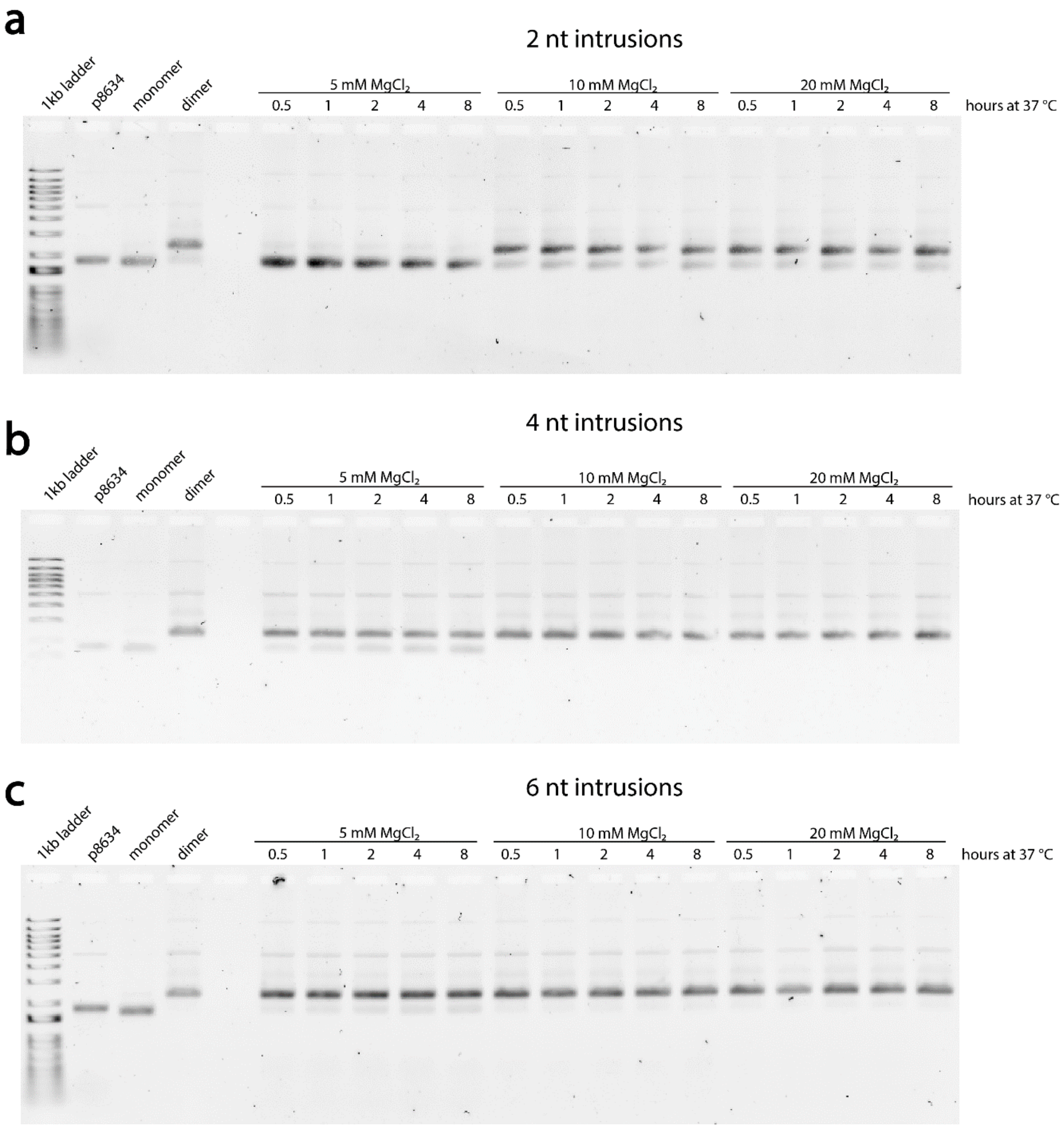


**Figure S82: AGE shift assay of xy-disassembly:** Dimers from xy-connections with (**a**) 2 nt, (**b**) 4 nt, and (**c**) 6 nt staple intrusions were formed over-night. They were diluted in buffers with a total $MgCl_2$ concentration of 5 mM, 10 mM, or 20 mM each, 8, 4, 2, 1, or 0.5 h before the samples were transferred to the gel. Data also used in figure 5e.

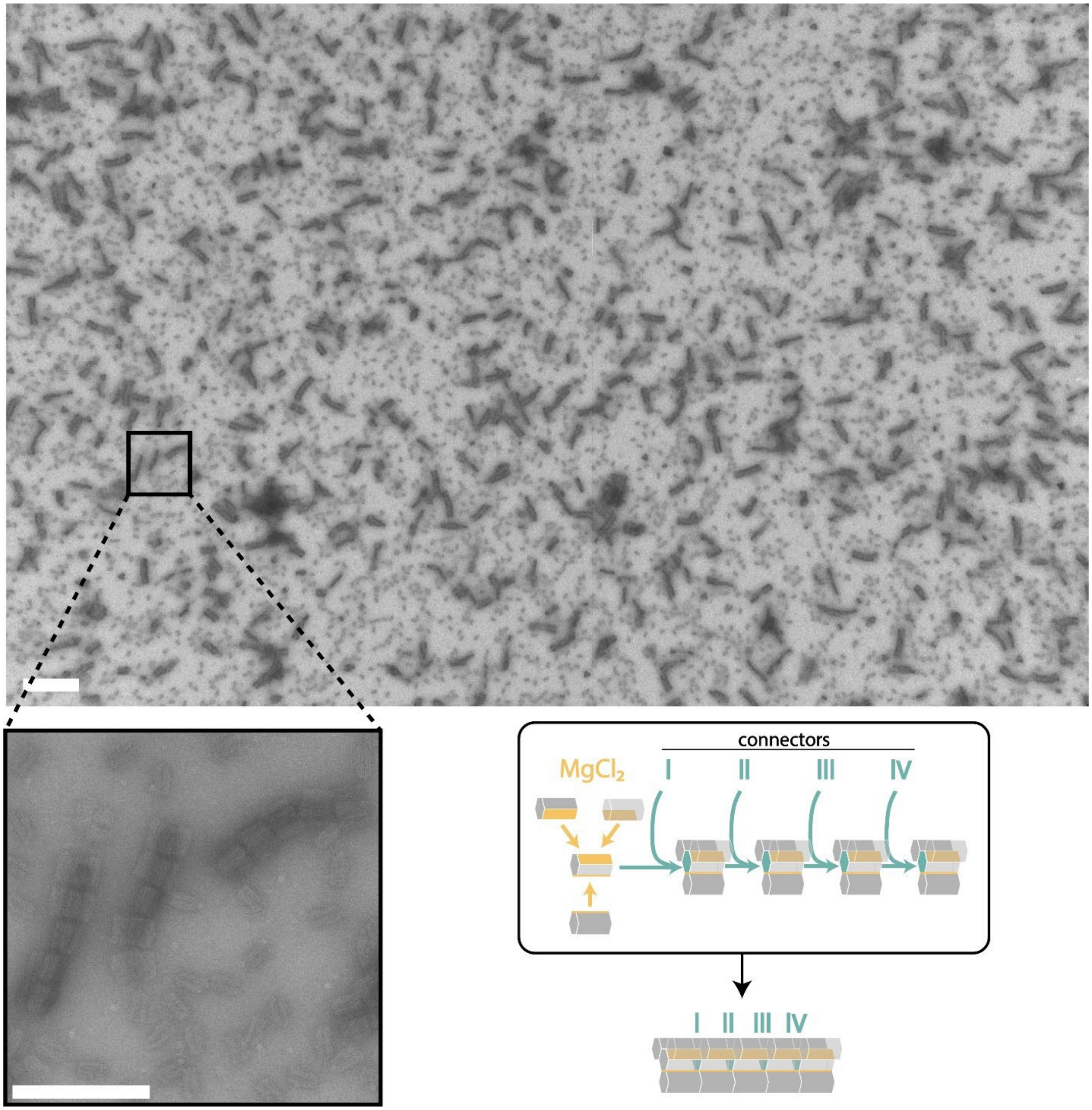


**Figure S83: TEM micrographs of tetrameric pentamer assembly** from xy- and z-connections. The central moDON monomers carry the same xy-connections: δεζ, connecting to moDONs with connection sites δ*, ε*, or ζ*, respectively. At the same time, the central moDONs have different z-connection handles, namely zI-right, zI-left/zII-right, zII-left/zIII-right, zIII-left/zIV-right, or zIV-left. Addition of respective connectors forms a pentamer of tetramers. Scale bar is 500 nm for the overview picture and 200 nm for the enlarged part.

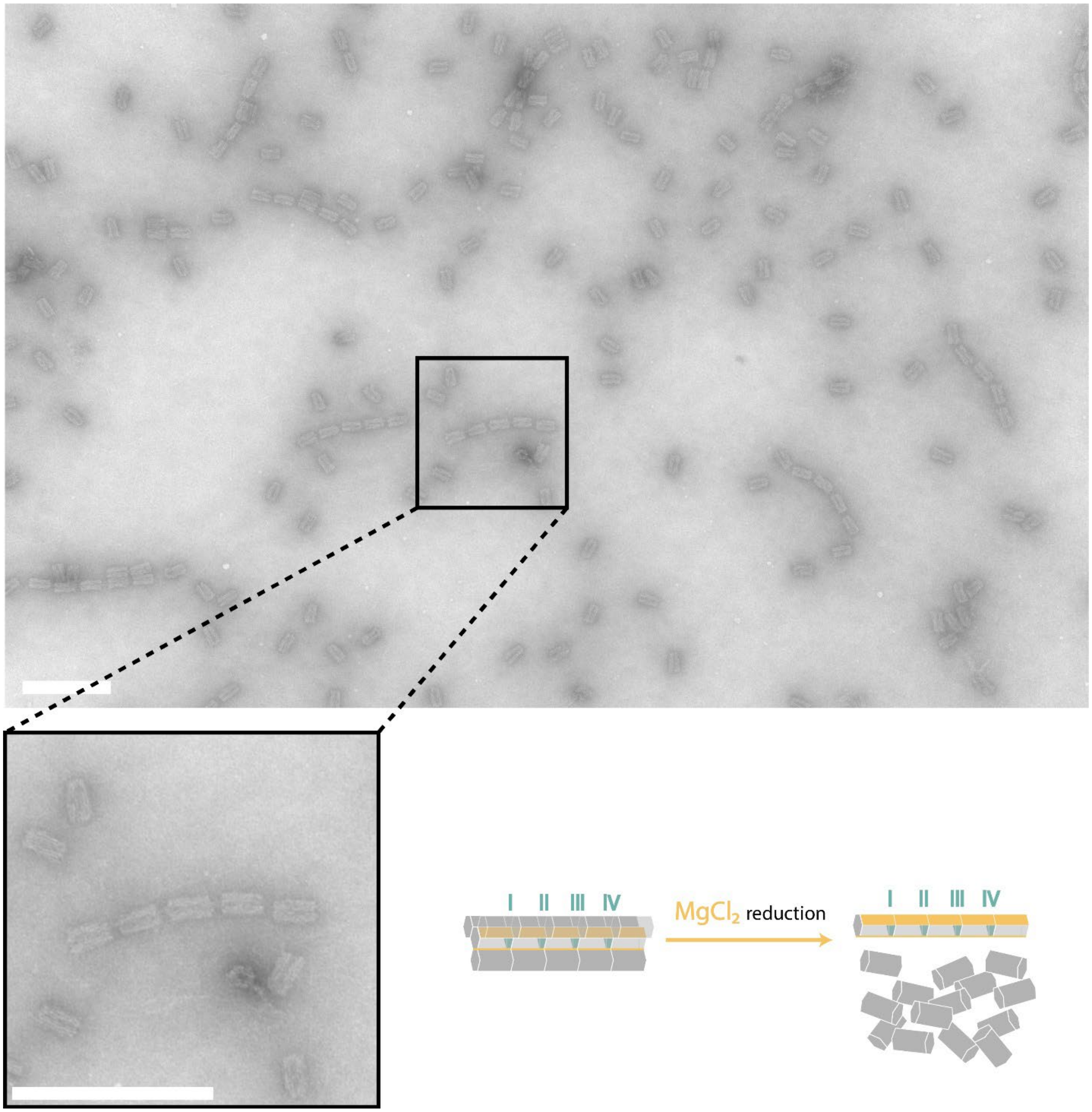


**Figure S84: TEM micrographs of tetrameric pentamer disassembly 1.** Reduction of $MgCl_2$ in the buffer solution leads to disassembly of xy-connections only, resulting in a z-pentamer and monomers. Scale bar is 500 nm for the overview picture and 200 nm for the enlarged part.

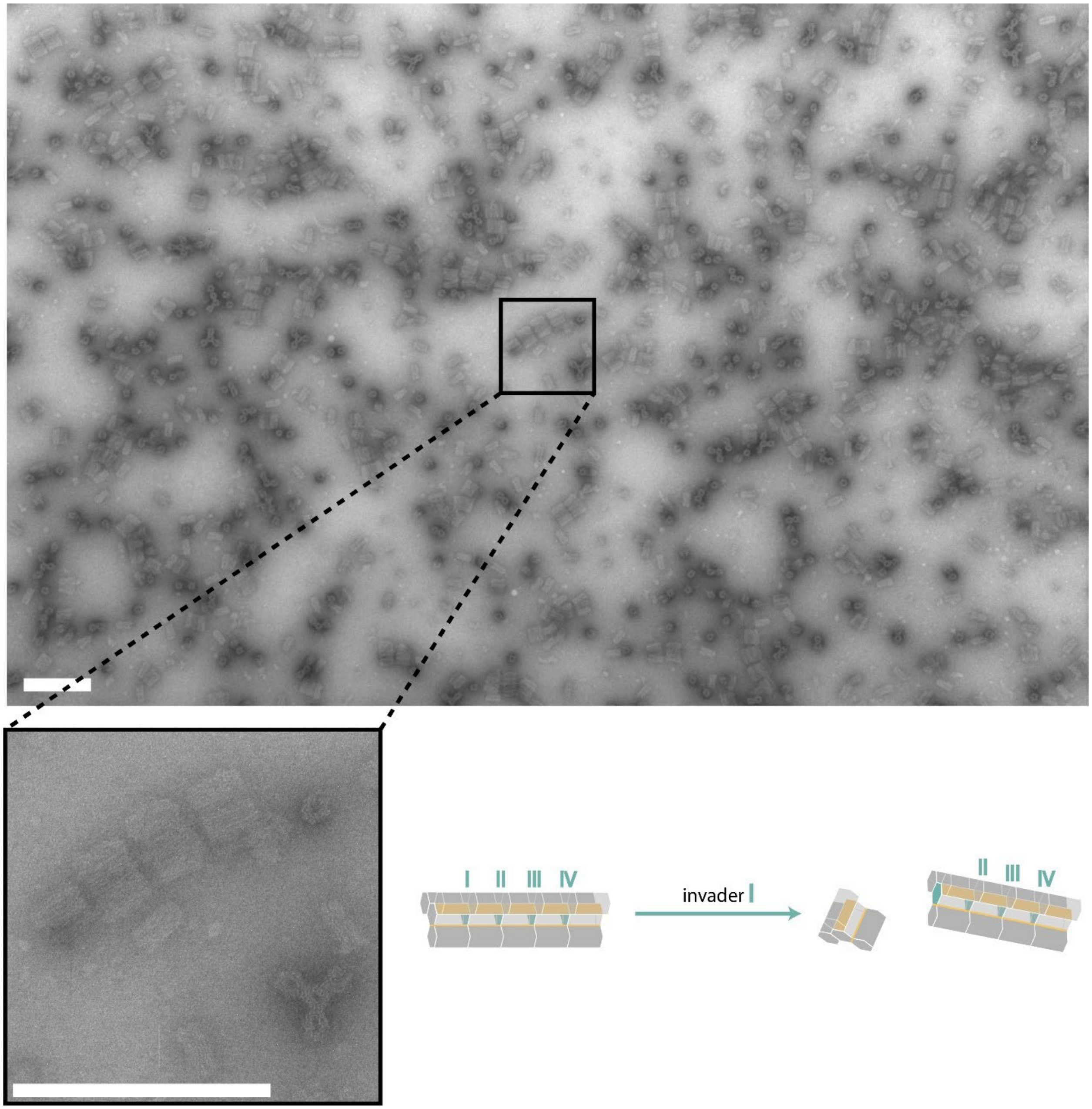


**Figure S85: TEM micrographs of tetrameric pentamer disassembly 2.** The addition of invader I leads to disassembly of the z-connection between the first and the second tetramer, resulting in a tetramer and a tetramer of tetramers. Scale bar is 500 nm for the overview picture and 200 nm for the enlarged part.

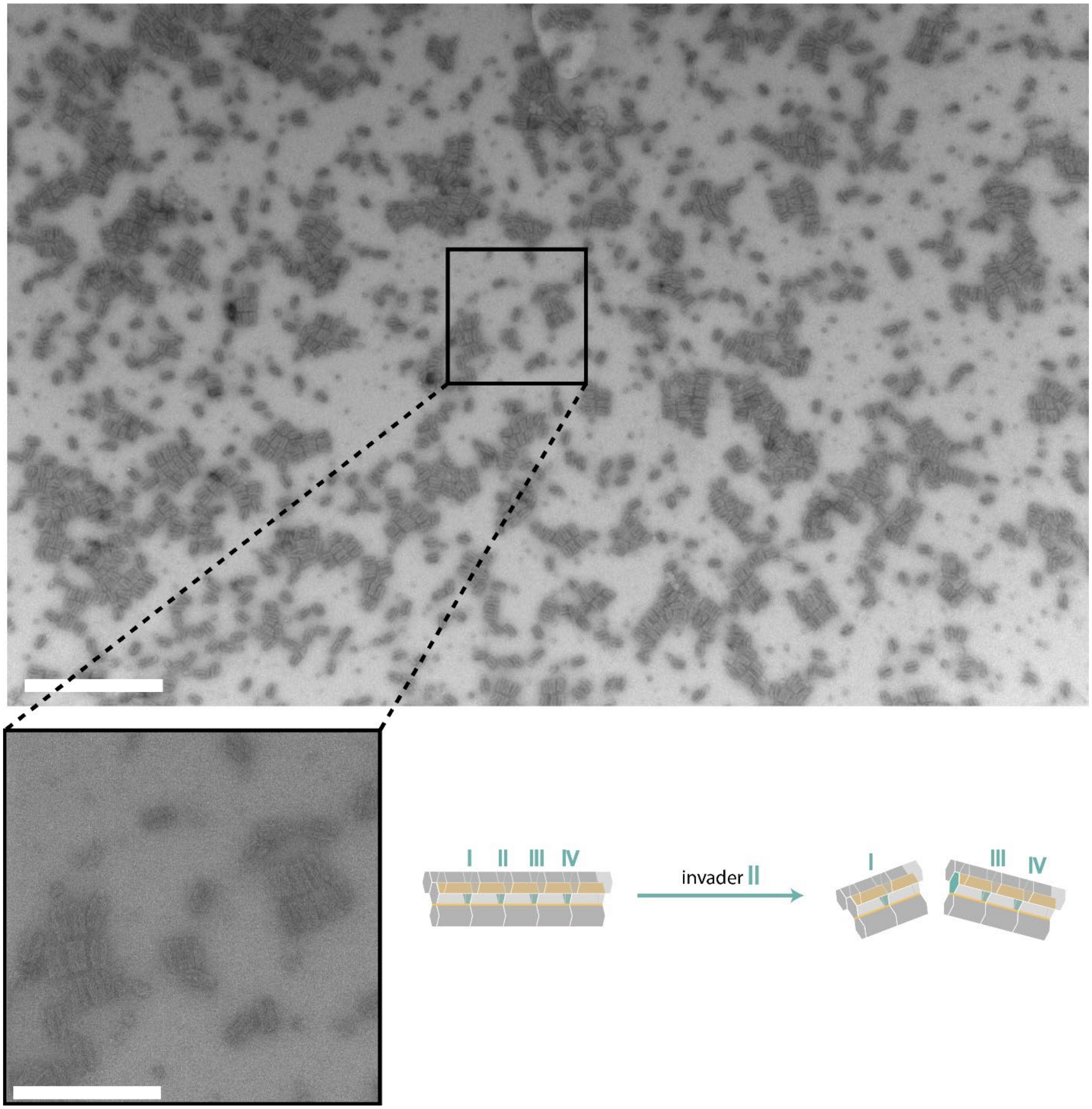


**Figure S86: TEM micrographs of tetrameric pentamer disassembly 3.** The addition of invader II leads to disassembly of the z-connection between the second and third tetramer resulting in a dimer and a trimer of tetramers. Scale bar is 500 nm for the overview picture and 200 nm for the enlarged part.

**a**

| constituent | | stock concencetration [µM] | end concentration [nM] |
|---|---|---|---|
| scaffold p8634 | | varying | 10 |
| core mix | | 0.820 | 50 |
| z connection mix: | left | 12.5 | 50 |
| | right | 11.111 | 50 |
| hinge helix mix: | 50 | 5.0 | 75 |
| | 55 | 5.0 | 75 |
| | 60 | 5.0 | 75 |
| | 65 | 5.0 | 75 |
| | 70 | 5.0 | 75 |
| | 75 | 5.0 | 75 |
| xy-connection mix: | 50-1 | 25.0 | 100 |
| | 50-2 | 25.0 | 100 |
| | 55-1 | 25.0 | 100 |
| | 55-2 | 25.0 | 100 |
| | 60-1 | 25.0 | 100 |
| | 60-2 | 25.0 | 100 |
| | 65-1 | 25.0 | 100 |
| | 65-2 | 25.0 | 100 |
| | 70-1 | 25.0 | 100 |
| | 70-2 | 25.0 | 100 |
| | 75-1 | 25.0 | 100 |
| | 75-2 | 25.0 | 100 |

**b**

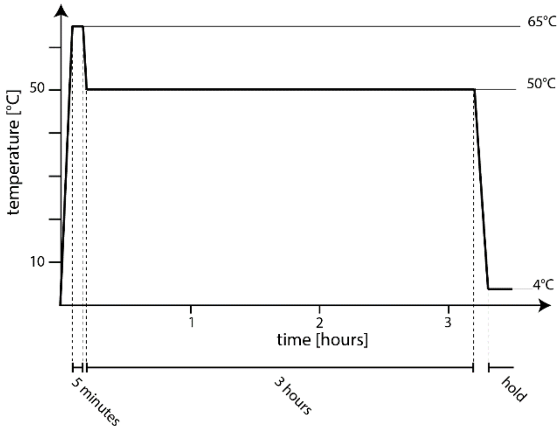


**Figure S87: folding conditions for the moDON monomers** (**a**) stock concentrations and end concentrations used in the folding mixture of all constituents. Concentrations of hinge helix mixes were adjusted to 5 µM and of xy-connection mixes to 25 µM manually. Most staple mixtures were stored in a 96 well plate and pipetted with a multichannel pipette for ease of use. (**b**) temperature graph of the optimized folding program used for folding moDON monomers.

**Table S1: Staple connection sites and the respective staple mixes**: Configuration 1 and configuration 2 differ only in one half of the total connection site. Each belongs to a different hinge helix. Since the distribution of changed helices is not point-, but mirror-symmetrically (cf. Figure S7), HH50 has four different conformations, HH65, on the opposite side has only one, and the others have two each. This results in the use of the following staples mixes for the modular parts, ordered by hinge helix. Similar colors indicate the same staple mix.

| hinge helix | connection site | staple mix |
|---|---|---|
| 50 | γ* α | γ* α |
| 50 | γ* δ | γ* δ |
| 50 | ζ* α | ζ* α |
| 50 | ζ* δ | ζ* δ |
| 55 | α β* | α β* |
| 55 | α ε* | δ ε* |
| 55 | δ β* | α β* |
| 55 | δ ε* | δ ε* |
| 60 | β* γ | β* γ |
| 60 | β* ζ | ε* ζ |
| 60 | ε* γ | β* γ |
| 60 | ε* ζ | ε* ζ |
| 65 | γ α* | γ α* |
| 65 | γ δ* | γ α* |
| 65 | ζ α* | γ α* |
| 65 | ζ δ* | γ α* |
| 70 | α* β | α* β |
| 70 | α* ε | α* β |
| 70 | δ* β | δ* ε |
| 70 | δ* ε | δ* ε |
| 75 | β γ* | β γ* |
| 75 | β ζ* | β γ* |
| 75 | ε γ* | ε ζ* |
| 75 | ε ζ* | ε ζ* |

**Table S2: Core staples** are constant for all configurations.

| name | sequence 5* -> 3* |
|---|---|
| core_001 | GGAGAATGGATCCCGCCAGTGTGTGCTG |
| core_002 | TAAATGCATCCTCGGAGAAATGACTGATACCGTGAATATTA |
| core_003 | GGTTTTGTATTTTATCTGAACTCTTTTT |
| core_004 | CATAACAACTTCCGTCGCATTCACCCTCATTCAG |
| core_005 | TTTTTCCGCTCACAATTGAGTGAGCTAACTCACATTTTTTT |
| core_006 | TAAACCCCAAAATTATTATCAGGCCAACGGATTTA |
| core_007 | TCGACATTACTTCTAATAACATCACTTGATCTCGG |
| core_008 | GTAACTAGTTTTGTAAAAGATCTTCACAGAGTCTG |
| core_009 | AATCAGATATAATCCAATATTACCGCCATCGTCTG |
| core_010 | AGGATGCAGGTTAGCCTCGTGTATTAA |
| core_011 | AGGTCAAGATGTCTGACGCTGGTAGCGG |
| core_012 | GTAGCAACATTACGCATCGCTATTACGGGCAAATTAGAAGAA |
| core_013 | CTGAGAAGTGTTTTGCGGGAGCTAAACAGGAGGCC |
| core_014 | GTAAAAGCTTGCTGGACAGTCAAATCACATTTGGG |
| core_015 | GGAAGACATTGCTAAACTGGAATACATCGTACCCC |
| core_016 | ATGGGAGAGGAGAACGAGGATATTGCGCAGGTGTTCCTGAGTAACCGTT |
| core_017 | AAATGGATGGCAGAACAAAATAAACAGC |
| core_018 | GAGCAGCTATCGGCAGTCTGTCCATCACGGTTGG |
| core_019 | TTACGATAACAGTATCGATTAGTTGCTATTTTGCGCGAGGCAAAAA |
| core_020 | GCGACCTAAGCGTTCTTAGTTTGACTGTTATCAAGCACTGCATCCTG |
| core_021 | TTTTTAAGAAACCAGCAAAGCAAC |
| core_022 | TGAAACATCTGACCAATACCGAACGAAC |
| core_023 | TTTTTTAAAAGGGACATTCTGGTCACACCGCTCAAGCCATTG |
| core_024 | GCCAACAAGAAGATGAGAGCCTGCTGAA |
| core_025 | CACCAGCGAGATAGAACCCTGCTACATTTATTAACCAGAA |
| core_026 | TGATAGCCACAGACAATATTTTTGAATGTTTAATGCGAAGTGATGAACG |
| core_027 | CTCAGGCACTGCGTGATGCAACTTTTTC |
| core_028 | AACAGTGGCATCTGCCTTTTT |
| core_029 | AAGACGCACTAATAGATTAGAGATAATATTATTATAGTCACA |
| core_030 | TGCCACTCATTGTTGTGAGTGTGGCGATAGAAATA |
| core_031 | ACTTGTGGGAGGATTGGGATAGGTCACGATGAGAA |
| core_032 | GAAGAATATCATTGATGCGTATTAACCATTTAACA |
| core_033 | GCAAATCAACAGTTGAAAGGAATCACCTAGCAGCAAGATGGG |
| core_034 | AAGGTTAAATTCGACAACTCGGGGAAACTTCACCGGTTCCG |
| core_035 | CTAACACTGGTCGTAAACAGAGAGGTTCGCGAAC |
| core_036 | TTTTTCAAATCCCCACCGAACTGTTTTT |
| core_037 | CACAATAGCCGTTCCCGATAGAGCGAAATTAACT |
| core_038 | TTTTTTTAATGAAAGATTAATGAAGATTTTT |
| core_039 | GAAGTATAACGACGCGGGTAC |
| core_040 | TTTTTTTTAAAAGTTTGAGTACCCGAACCTCACTG |
| core_041 | CATTTTGGAAACCACGCCATTAGCCAGCTCGGCCTCAGGAAG |
| core_042 | TTTTTAAATTAATTACAACAGTTCAGGGATTTTT |
| core_043 | CAGTACATCTGTAAAGGTTGGGTTATATAACTATTTTT |
| core_044 | TTTTTTGCCGGAAACCACCTCAAA |
| core_045 | CGCAACTGATTAAGGAGTCAATAGTGAATTTATCAAAATCA |
| core_046 | ATCGTCGCTATTAATTAATTTTACCTTT |
| core_047 | TTTTTAGTGAATAACCTTGCTTAAATCA |
| core_048 | ACGCTCGCACTCCCGCCATTTAAAGCATTGAGG |
| core_049 | TTTTTAGTTTGAGGGGACGACTAACCGTCCACGCTAAAACAG |
| core_050 | TTTTTTATGTAAATGCTGAAGCGGGACGCAGACCTTTTAACTGCT |
| core_051 | CGCATCGGACAGTATTTCCGGCACCGCTTCTGGTTTTT |
| core_052 | TTGGTGTAATGAAAAATGCCACAAGTTCCAGGCTG |
| core_053 | CGGATTGACCGTAACTGGAACTTATCAGTTCAGGGAGGGCGACAAGGC |

| | |
|---|---|
| core_054 | AAATATATTTTAGTTATTTTT |
| core_055 | TTTTTCTTATCATTCCATTTATTTTCATCGTAGGAATTTTT |
| core_056 | CCAGCTTTAATTCGGTAAGAATACGTGGCCTAAAACATCGCCATTAAA |
| core_057 | AAATCAGATCGATTGTGCTGGCCATGAA |
| core_058 | TTTTTAAAATTCGCGGATGAACGGGATTTTT |
| core_059 | AGTTAAAATATGTTTTGAAGCCTTAAATCCCGACTTTAACCGAGTAAC |
| core_060 | CCAATAGGAGCGTCTTTCCTTTTT |
| core_061 | GTCCATGATATTATTTGTGCACATAAACATTGCTAAGAAAG |
| core_062 | ATAAGTCGGCAGACTACAGCGCAACACA |
| core_063 | TTTTTAATTGCGTTGCGGTTATTAATTTTTT |
| core_064 | TGCCAGCTACAAACTCTAAAATATCTTTAGGAGCA |
| core_065 | TGCATTAGACGGGCAACAGCTGATTGCCCCTGTCG |
| core_066 | GCGCGGGGTTTTTCAAAGATTGGGCGTTATCAATGTGGGCGC |
| core_067 | TTTTTAGCCCCAAAAACAGGAGGTTGATAATCAGAAATTTTT |
| core_068 | TTTATCCTGAATCTTACCAA |
| core_069 | AATCGTAAAACTAGCATGTCAGAGCCG |
| core_070 | TTTTTAGAGCCTAATTTGCCAGTTTTCACCA |
| core_071 | TTTTTTCATATGGTAACCGATTGAGGTTTTT |
| core_072 | CAAAGGCCAAGAGAAGGGAAACTGCGTG |
| core_073 | TTAATCATATTCATATAGCAGCACCGTGCGTCAG |
| core_074 | TTTTTAGTAATGTGTAGGTAATTAAATGCAATGCCTGTTTTT |
| core_075 | GGCCGGAGTAATATAATCAAAACTCAACTTGAGCT |
| core_076 | ATGATATTCAACCGCACCGTCACGTCAC |
| core_077 | TTTTTGAGGGAAGGTAAATATTGACGGAAATAAAGGGC |
| core_078 | TTTTTGCGGGAGAAGCCTTTATTTCAACGCAAAACATTATGACCC |
| core_079 | AGGATAAAACCCTCATATATTAGATTCAAAAGGGTTCCAAAT |
| core_080 | CACCAGTAGCTAAACCACCGA |
| core_081 | GCCGGAAACCGACTTGAGCCCATCAATCTCAAACGTGAGTA |
| core_082 | TTTTTATCAAGTTTCGGCATTTTCGGTTTTT |
| core_083 | TAGCGCGTCGCAAATGGTCAATAACCTGTTTAGC |
| core_084 | TTTTTGCCCGTATATGTAATACTTTTTTTTT |
| core_085 | TTTTTTTAGTTTGACCATTAGCTGCGAACGAGTAGATTTTTT |
| core_086 | GGGCGCGAGCTGAACGAGAGG |
| core_087 | CCCAATTATACATTTTTTCATGCCTTTAAATCAGTAGCGACAGATTTTT |
| core_088 | TAAGTATTCATTTGCTAATAGTAGTAGCATTAACATCCAAT |
| core_089 | CAGGGTGGAGAGGCGGTTTGC |
| core_090 | AGATATAGAGTCGGCATACAAAAGGTTT |
| core_091 | GAGTTGCGCGAAAAAATAGCCCGAGATATCCACTA |
| core_092 | CAGTGAATGAATCCGAGTACACATATAGATGAT |
| core_093 | CTCAGAGGCTCAGTGAGGCTGAGACTCCGTATAACAATGCGC |
| core_094 | GAGCTTAATTGCTGAGGAGCGGAGATCG |
| core_095 | TTTTTAGCAAGCCCGGGCGCGTACTATTTTT |
| core_096 | AACTACAGAAGCAAGACCATATTGAATCCCCCTCAGATAGCG |
| core_097 | TTTTTCATAATGCCTCGCCTGATAAATTTAGCCGGGTGTCTT |
| core_098 | TTTTTACCCTGACTATTATAGTCAACGCCTGTAGCATTTTT |
| core_099 | TTTTTAAACGGGTAAAATACGTGAGGAACTTACTGTAGTGTC |
| core_100 | ACACTAAAACACTCTAAGAGGAAGCCCGGATTAGA |
| core_101 | TTTTTCATAAATATTCAAATCAAAAATCAGGTCTTTTTTTT |
| core_102 | TTTTTCCTGCTCCATGTTACTGTGTCGAAATCCGCGATTTTT |
| core_103 | TAGACTGAATGCTCAGAAAACGAGAATAGCGGATTTATAAACTCCAAC |
| core_104 | AAAAACCAAACGGCGCAGAATGTATCAACTACG |
| core_105 | TTTTTTTCAACTAATGCAGATACACTGCGGAATCGTTTTTT |
| core_106 | AAAAGGACTGGGGTTCCAGTCTATTAAATCCTTTGACATTAT |
| core_107 | GTCTCCAGTATTATGTTTCCATTTTTTT |
| core_108 | TGATGGTGCCTGGCCCTGAGACCCGCTT |
| core_109 | AATGAGTTGCAAGGAGTTTATAAGGCAAAAATCATAAATGTT |
| core_110 | AATCCCTGCATCAAAAAGAT |

| | |
|---|---|
| core_111 | CGTGCCAGCGAAAAATATAATGCTGTCTTTGAG |
| core_112 | TTAAAGAACGTGGATCAAAAGTCCTGTT |
| core_113 | GTCAAAGCACCCGCCGCGCTTGTGCTTT |
| core_114 | CGAGAAAGGGCGCTTGGCTTAAGGTTTAGTACCGC |
| core_115 | GCGTCGTAACGCTTAACAAGACCCGTTA |
| core_116 | TTTTTCTGAATTTCCATGTTTTAAATTTTTT |
| core_117 | GCGCGTAACCACCAGGCGAAAAACCGTCTATCAAGCCGGCGA |
| core_118 | TCACGCTATAAGAGGTCATTTAGGTCAGAAAGACT |
| core_119 | GGCAAGTGCATTCGCATCAAATATATTTAGCCCGGAATAGGTACTCAGG |
| core_120 | GGAGAGTGGCGCTAGGAAGGGGATACCGTTTAGCTGAAACGAACGTGG |
| core_121 | TTTTTTGGTTGCTTTGACGAGCACTCAAGAGAAGGATTTTT |
| core_122 | GAGTATCTGCATATTGGGTTC |
| core_123 | CAGAATCCAACAGGAAAAACGCTCATTTTTT |
| core_124 | AGGTGAGGCGGTCAGTATTTTTTT |
| core_125 | TTTTTTTTCTGCGGCAGTTAATCGGTGAAAATGTTTTT |
| core_126 | TTTTTGAAACCAGTTTCTTGTGAAGTCCGTGAAGACGTTTTT |
| core_127 | TTTTTTTTATGTAGATGAAGGTAT |
| core_128 | TTTGGAAATACCTACATTTTGAGACCAGTAATTTTT |
| core_129 | TTTAATATCTGGTCAGTTGTATCAAA |
| core_130 | TGTTCGCCACTGGTGACCTGGAAGAGTTT |
| core_131 | GCAATAAAAATGCGCCGCCTTT |
| core_132 | AGATGATGACCGTACTCAATTT |

**Table S3: Staples for HH50 modular part in configuration 1:** Staples also used in other configurations are printed in red, and on the right the respective other configuration is marked with a "y".

| name | sequence 5* -> 3* | δεζ | γ*δ | ζ*α |
|---|---|---|---|---|
| **hh50_Gamma*Alpha_shell_01** | **CGAGCTCGTACAAAGGTGGAAACGATACTTAAAGTAGCATGC** | | **y** | |
| **hh50_Gamma*Alpha_shell_02** | **CCTCTCTGAATTCGTAATCATGGTCATAGCCGGAGTAAAGC** | | **y** | |
| **hh50_Gamma*Alpha_shell_03** | **GCCTAATCCACACATAACGGAACAACATTATTATTTTT** | **y** | **y** | **y** |
| **hh50_Gamma*Alpha_shell_04** | **ATCAGTTTTAAACTTTGACCCAATAGTAGAGTATC** | | | **y** |
| hh50_Gamma*Alpha_shell_05 | TGCAAAAGAAGTTTTGAGCAATTTTCAC | | | |
| **hh50_Gamma*Alpha_shell_06** | **TCCAATATAACGCCTTCAGTTTTCATATACCAGTC** | | **y** | |
| hh50_Gamma*Alpha_shell_07 | TCAATCACAAATCATGACAAGAACCGGA | | | |
| **hh50_Gamma*Alpha_shell_08** | **AGGCGCAGGGGATTTTTTATGGAGATGA** | **y** | **y** | **y** |
| hh50_Gamma*Alpha_shell_09 | TTTTTTGAACGGTGTACAGACCTATTGAAAGAGGACAGATTTTT | | | |
| **hh50_Gamma*Alpha_shell_10** | **AACGAACACATACGAGCTGTTTCCTGTG** | | **y** | **y** |
| **hh50_Gamma*Alpha_shell_11** | **TTTTTCAGGTAGAAAGAGAGATTTAGGAATACCACATTTTT** | **y** | | |
| hh50_Gamma*Alpha_shell_12 | ACCAACTTTCATTACCTAAGGGAATTCTGC | | | |
| hh50_Gamma*Alpha_shell_13 | CCTTATGTAAGAGCAACACTAAGGGGGTCCAGCGA | | | |
| hh50_Gamma*Alpha_shell_14 | TTTCTTTAGATCCGAACGAGG | | | |
| hh50_Gamma*Alpha_shell_15 | CATACATTAGAGTCTGCCAGTCATAACATCATTGTGAATTA | | | |
| | | | | |
| hh50_Gamma*Alpha_passive_Gamma*_01 | AATGTGCCACTCGCTAGGCTGGCTGACCTTCATTTTTT | | | |
| **hh50_Gamma*Alpha_passive_Gamma*_02** | **ATTACGAGGCATAGCGATTTTTGGGAAGAATTTTT** | | **y** | |
| **hh50_Gamma*Alpha_passive_Gamma*_03** | **TTTTTAAATCTACGTTAATAAAGGACGTAAGAACTGGCTCA** | | **y** | |
| hh50_Gamma*Alpha_passive_Gamma*_04 | TTTTTCAAGAGTAATCTACGTAACAAAGCTGCTCATTC | | | |
| hh50_Gamma*Alpha_2nt_intrusion_Gamma*_01 | AATGTGCCACTCGCTAGGCTGGCTGACCTTCATGC | | | |
| **hh50_Gamma*Alpha_2nt_intrusion_Gamma*_02** | **ATTACGAGGCATAGCGATTTTTGGGAAGAAGC** | | **y** | |
| **hh50_Gamma*Alpha_2nt_intrusion_Gamma*_03** | **CCCAAGAGTAATCTACGTAACAAAGCTGCTCATTC** | | **y** | |
| hh50_Gamma*Alpha_2nt_intrusion_Gamma*_04 | TGAAATCTACGTTAATAAAGGACGTAAGAACTGGCTCA | | | |
| hh50_Gamma*Alpha_passive_Alpha_01 | GTACAACACCAGAAAATAAGGCTTGCCCTGTTTTT | | | |
| hh50_Gamma*Alpha_passive_Alpha_02 | AGATTTCGTTTATGCGAGTAGTAAATTGGGTTTTT | | | |
| hh50_Gamma*Alpha_passive_Alpha_03 | TTTTTCTTGAGATGAACTTTACCTCGTTTACCAGA | | | |
| hh50_Gamma*Alpha_passive_Alpha_04 | TTTTTACGAGAAACGGAGATTTAGCGAGAGGCTTTCGACGAT | | | |
| hh50_Gamma*Alpha_2nt_intrusion_Alpha_01 | GTACAACACCAGAAAATAAGGCTTGCCCTGTA | | | |
| hh50_Gamma*Alpha_2nt_intrusion_Alpha_02 | AGATTTCGTTTATGCGAGTAGTAAATTGGGTG | | | |
| hh50_Gamma*Alpha_2nt_intrusion_Alpha_03 | AAACGAGAAACGGAGATTTAGCGAGAGGCTTTCGACGAT | | | |
| hh50_Gamma*Alpha_2nt_intrusion_Alpha_04 | CCCTTGAGATGAACTTTACCTCGTTTACCAGA | | | |

**Table S4: Staples for HH55 modular part in configuration 1:** Staples also used in other configurations are printed in red, and to the right the other configuration is marked with a "y".

| **name** | **sequence 5* -> 3*** | **δεζ** |
|---|---|---|
| hh55_AlphaBeta*_shell_01 | ACCAGGCGGATAATCAGAACGTTTGCTTTTAATT | |
| **hh55_AlphaBeta*_shell_02** | **TTTTTATGCAACTAAAGAGGCCGCTTTTTTT** | **y** |
| hh55_AlphaBeta*_shell_03 | ACACTGACGCCACCTTCTGTATGGGATT | |
| **hh55_AlphaBeta*_shell_04** | **TTTTTGAGGGTAGCAACGGCTAAGACAGCATCGGAACTTTTT** | **y** |
| hh55_AlphaBeta*_shell_05 | CGTCACGAATAATAGAAAGGAACAACTATGAATT | |
| hh55_AlphaBeta*_shell_06 | ATCGCGTAAGCAAACCGACAA | |
| hh55_AlphaBeta*_shell_07 | AAAGAATTTATACCAAGCGCGAAACAAAAAACGAAAGCTTGC | |
| hh55_AlphaBeta*_shell_08 | CGATAGTTGGGCTCAAAACGCTCCAACTTGCGGA | |
| **hh55_AlphaBeta*_shell_09** | **GCAGCGAACAGAGGAGCTCAA** | **y** |
| **hh55_AlphaBeta*_shell_10** | **TTTTTTTCCACAGACAGTAGCGTAACGATCTAAAGTTTTTT** | **y** |
| **hh55_AlphaBeta*_shell_11** | **TTTTTTTGCGGGATCGTCACCGAGTTAATACGGTG** | **y** |
| hh55_AlphaBeta*_shell_12 | AAATAGTCCCTCAGGAATTGCCAGTACATACCGTA | |
| hh55_AlphaBeta*_shell_13 | TGAGAATAATTTTTTCACGTTGAGTACCCCTTTTG | |
| **hh55_AlphaBeta*_shell_14** | **TTTTTTTTGTCGTCTTTTTCAGGGATTTTTT** | **y** |
| **hh55_AlphaBeta*_shell_15** | **CGCTACAAATAGGACCTCATTCCAGACGTTAGTAA** | **y** |
| | | |
| hh55_AlphaBeta*_passive_Alpha_01 | TTTTTTTAATTGTATCGGTTTATCAGAGGCATCAAAT | |
| hh55_AlphaBeta*_passive_Alpha_02 | TTTTTTTAAACAGCAACCATCGCGAACCAGACCGGTTTAATTCAACCTA | |
| hh55_AlphaBeta*_passive_Alpha_03 | TGACAACTTGATACTTTCGAGGTGAATTTCTTTTT | |
| hh55_AlphaBeta*_passive_Alpha_04 | GAAAATCTCCAAAACAAAAGGAGCCTTTTTT | |
| hh55_AlphaBeta*_2nt_intrusion_Alpha_01 | TGACAACTTGATACTTTCGAGGTGAATTTCCA | |
| hh55_AlphaBeta*_2nt_intrusion_Alpha_02 | GAAAATCTCCAAAACAAAAGGAGCCTCG | |
| hh55_AlphaBeta*_2nt_intrusion_Alpha_03 | AATTAAACAGCAACCATCGCGAACCAGACCGGTTTAATTCAACCTA | |
| hh55_AlphaBeta*_2nt_intrusion_Alpha_04 | AGTTAATTGTATCGGTTTATCAGAGGCATCAAAT | |
| hh55_AlphaBeta*_passive_Beta_01 | GTTGATACACCCTCAGAACCACAACTTTCTTTTT | |
| hh55_AlphaBeta*_passive_Beta_02 | AAGGCACCGAGCTTCAAAGACGACTAAAGCCCACGATATTCGGTTTTTT | |
| hh55_AlphaBeta*_passive_Beta_03 | TTTTTAACAGTTTCAGCGGAGTTGCTAAGCCACCCGTGCCGT | |
| hh55_AlphaBeta*_passive_Beta_04 | TTTTTCGCTGAGGCTTGCAGGCTCCGATCATAACA | |
| hh55_AlphaBeta*_2nt_intrusion_Beta_01 | GTTGATACACCCTCAGAACCACAACTT | |
| hh55_AlphaBeta*_2nt_intrusion_Beta_02 | AAGGCACCGAGCTTCAAAGACGACTAAAGCCCACGATATTCGGTTC | |
| hh55_AlphaBeta*_2nt_intrusion_Beta_03 | CACGCTGAGGCTTGCAGGCTCCGATCATAACA | |
| hh55_AlphaBeta*_2nt_intrusion_Beta_04 | TCAACAGTTTCAGCGGAGTTGCTAAGCCACCCGTGCCGT | |

**Table S5: Staples for HH60 modular part in configuration 1:** Staples also used in other configurations are printed in red, and to the right the other configuration is marked with a "y".

| name | sequence 5* -> 3* | δεζ |
|---|---|---|
| **hh60_Beta*Gamma_shell_01** | **AGTGAGGTCGGTTATTTCGGTCTGAATTTACCGT** | y |
| **hh60_Beta*Gamma_shell_02** | **ACATACAAAATCTGTCAGAGGCCGCCACCCTCAG** | y |
| hh60_Beta*Gamma_shell_03 | AATGAAATATTCGGTGGCATCGCCAGAA | |
| **hh60_Beta*Gamma_shell_04** | **TTTTTTTAGGATTAGCGGGAGTGTACTGGTAATAAGTTTTT** | y |
| **hh60_Beta*Gamma_shell_05** | **CCGGAACGTTGATTTCTGGAAGTTTCATTCACCGTGTATCCA** | y |
| **hh60_Beta*Gamma_shell_06** | **TTTTTTTCATAATCAAAATCAAAGCGTTTATTACCGCCACCC** | y |
| **hh60_Beta*Gamma_shell_07** | **TTTTTTTTTAACGGGGTAGTAACAGTTTTTT** | y |
| hh60_Beta*Gamma_shell_08 | GCCGCCACCATCCTAATAAAGGGAGGTT | |
| **hh60_Beta*Gamma_shell_09** | **TTTTTTCATAGCCCCCTTGCCATCTTTTTTT** | y |
| **hh60_Beta*Gamma_shell_10** | **TCCAGTAAATGCCCCCTGCCTGTACCAA** | y |
| hh60_Beta*Gamma_shell_11 | TGGAAAGCGCAGTCAACCTATCATGAAA | |
| hh60_Beta*Gamma_shell_12 | GAGGCAGTAGCAAGCAATAAAGCCTCAGAGCATAA | |
| | | |
| **hh60_Beta*Gamma_passive_Beta*_01** | **AACAGTTAGCCTTGCAGTGCGTCATACATGGCTTTTTT** | y |
| **hh60_Beta*Gamma_passive_Beta*_02** | **TATAACACAGAGCCACCACCGGAACCTTTTT** | y |
| **hh60_Beta*Gamma_passive_Beta*_03** | **TTTTTTTTGATGATACAGGGTTTTCCACCACACCCATG** | y |
| hh60_Beta*Gamma_passive_Beta*_04 | TTTTTGCCTCCCTCAGACCACCACCCTCAGA | |
| **hh60_Beta*Gamma_2nt_intrusion_Beta*_01** | **AACAGTTAGCCTTGCAGTGCGTCATACATGG** | y |
| **hh60_Beta*Gamma_2nt_intrusion_Beta*_02** | **TATAACACAGAGCCACCACCGGAA** | y |
| **hh60_Beta*Gamma_2nt_intrusion_Beta*_03** | **TGATGATACAGGGTTTTCCACCACACCCATG** | y |
| hh60_Beta*Gamma_2nt_intrusion_Beta*_04 | CTCCCTCAGACCACCACCCTCAGA | |
| hh60_Beta*Gamma_passive_Gamma_01 | TTACCATGTCAGACGATTGTTTTT | |
| hh60_Beta*Gamma_passive_Gamma_02 | TTTTTGCCTTGATATTCACAAACACATTAAAAATTCTA | |
| hh60_Beta*Gamma_passive_Gamma_03 | ATTGACAAACCACCACCAGAGCCTTTTT | |
| hh60_Beta*Gamma_passive_Gamma_04 | TTTTTGCCGCCAGCCAATGAACAAAGAATTAGCAAAATTAAG | |
| hh60_Beta*Gamma_2nt_intrusion_Gamma_01 | TTACCATGTCAGACGAT | |
| hh60_Beta*Gamma_2nt_intrusion_Gamma_02 | CTTGATATTCACAAACACATTAAAAATTCTA | |
| hh60_Beta*Gamma_2nt_intrusion_Gamma_03 | ATTGACAAACCACCACCAGAG | |
| hh60_Beta*Gamma_2nt_intrusion_Gamma_04 | CGCCAGCCAATGAACAAAGAATTAGCAAAATTAAG | |

**Table S6: Staples for HH65 modular part in configuration 1:** This part is the same for both configurations and not changed. All staples are printed in red, and to the right the other configuration is marked with a "y".

| name | sequence 5* -> 3* | δεζ |
|---|---|---|
| hh65_GammaAlpha*_shell_01 | AGATTGTACGCAAAGACACCACGGAATTTTT | y |
| hh65_GammaAlpha*_shell_02 | GAGCAAATATCAGGTCATTGCAATAAGA | y |
| hh65_GammaAlpha*_shell_03 | TTTTGAGAGATCTAAATAGCA | y |
| hh65_GammaAlpha*_shell_04 | GAATTATTTCTAGCTGATAAAGAGAGGGTTAGCAAA | y |
| hh65_GammaAlpha*_shell_05 | GAGGAAACTATCTTACCGAAGACAATGA | y |
| hh65_GammaAlpha*_shell_06 | CGTAGAAAATACACGCCAAAATCATATAAACGCC | y |
| hh65_GammaAlpha*_shell_07 | TTTTTAAAATGAAAATAGGGAAGCGCTTTTT | y |
| hh65_GammaAlpha*_shell_08 | TTACAGAAAGAAACGATTTTTTGTTTAACGTCATTTTT | y |
| hh65_GammaAlpha*_shell_09 | GAAATTGACAAGGAATAACATAACTGAACGCTAACCAAGCAAAGCCGTT | y |
| hh65_GammaAlpha*_shell_10 | GCCCAATCTTATTTATCCCAAGAGAAA | y |
| hh65_GammaAlpha*_shell_11 | GCAAGAACCCTTTTACCAGAAGGAAACC | y |
| hh65_GammaAlpha*_shell_12 | ATTTACGCCTTAAGCGCAATAATAACGG | y |
| hh65_GammaAlpha*_shell_13 | TTTTTTAAGTTTATTTTATAGAAAATTTTTT | y |
| hh65_GammaAlpha*_shell_14 | TTTTTATTAGACGGGAGAATTAAAAACAGCAGCCT | y |
| hh65_GammaAlpha*_shell_15 | CACCCTGAACAAAGATAACCCAGTTAA | y |
| | | |
| hh65_GammaAlpha*_passive_Gamma_01 | AGCACCAAATTAGACAAAGTTTAAGAAAAGTAAGCAGTTTTT | y |
| hh65_GammaAlpha*_passive_Gamma_02 | GGCAAGGACCATCGTAAAGGTAATACCCAAAAGTTTTT | y |
| hh65_GammaAlpha*_passive_Gamma_03 | TTTTTATAGCCGAAGCCAGCAAATTTAGAAATTAT | y |
| hh65_GammaAlpha*_passive_Gamma_04 | TTTTTAACTGGCATGATTAAGACTCAGTATGTAGCTAT | y |
| hh65_GammaAlpha*_2nt_intrusion_Gamma_01 | AGCACCAAATTAGACAAAGTTTAAGAAAAGTAAGCAGCT | y |
| hh65_GammaAlpha*_2nt_intrusion_Gamma_02 | GGCAAGGACCATCGTAAAGGTAATACCCAAAAGAG | y |
| hh65_GammaAlpha*_2nt_intrusion_Gamma_03 | AGAACTGGCATGATTAAGACTCAGTATGTAGCTAT | y |
| hh65_GammaAlpha*_2nt_intrusion_Gamma_04 | TTATAGCCGAAGCCAGCAAATTTAGAAATTAT | y |
| hh65_GammaAlpha*_passive_Alpha*_01 | CATATGAGAGTCTGCTACAATGTAATTGAGTTTTT | y |
| hh65_GammaAlpha*_passive_Alpha*_02 | GACATTCTTACCAGTAAATCAGTCACCATAAAGGTGGCAATTTTT | y |
| hh65_GammaAlpha*_passive_Alpha*_03 | TTTTTCGCTAATATCAGAGAGTCAGAGG | y |
| hh65_GammaAlpha*_passive_Alpha*_04 | TTTTTCATATAAAAGAAATAAGCAATTGTAATTTTGTT | y |
| hh65_GammaAlpha*_2nt_intrusion_Alpha*_01 | CATATGAGAGTCTGCTACAATGTAATTG | y |
| hh65_GammaAlpha*_2nt_intrusion_Alpha*_02 | GACATTCTTACCAGTAAATCAGTCACCATAAAGGTGGC | y |
| hh65_GammaAlpha*_2nt_intrusion_Alpha*_03 | CTAATATCAGAGAGTCAGAGG | y |
| hh65_GammaAlpha*_2nt_intrusion_Alpha*_04 | TATAAAAGAAATAAGCAATTGTAATTTTGTT | y |

**Table S7: Staples for HH70 modular part in configuration 1:** Staples also used in other configurations are printed in red, and to the right the other configuration is marked with a “y”.

| **name** | **sequence 5* -> 3*** | **δεζ** |
|---|---|---|
| hh70_Alpha*Beta_shell_01 | TGAAAGCCGTCTGGCGGTATTATAGATAAGTCCTGCATGTTC | |
| hh70_Alpha*Beta_shell_02 | TTGGTAACATAGTCGCTATCCCTCATTTTTGCGGG | |
| hh70_Alpha*Beta_shell_03 | TTAACCTCGCAAGACGTCGGAACCCAAGCCTGTT | |
| hh70_Alpha*Beta_shell_04 | AGAACGGGTATTAAGTAATTCACGACAA | |
| hh70_Alpha*Beta_shell_05 | TCATCAACATTAAATTATACA | |
| hh70_Alpha*Beta_shell_06 | ATCGAGAACAAGCAATCAGATAAAATAA | |
| hh70_Alpha*Beta_shell_07 | ACGCCATCGTTTTAGCGAACCTCAAGATGAACGGT | |
| **hh70_Alpha*Beta_shell_08** | **TTTTTTCATTACCGCGCTTACGAGCATTTTT** | **y** |
| hh70_Alpha*Beta_shell_09 | TTTTTAATACCGACCGTGTATTTTGTTTTTTT | |
| hh70_Alpha*Beta_shell_10 | TATCCCATAAGACGTGTCCATCGGCTGTCTTTCTTTTT | |
| **hh70_Alpha*Beta_shell_11** | **TTTTTTGTAGAAACCAATCAATCCTAAT** | **y** |
| hh70_Alpha*Beta_shell_12 | TAAACAAAACAAGAATAGAAGGCTTATCCCCACTC | |
| hh70_Alpha*Beta_shell_13 | TAGTATCATATGCGTGTGAGCAATAGGA | |
| hh70_Alpha*Beta_shell_14 | AGAAAAAAGAACGCGAGAAAAATCCAATCCGGCTT | |
| hh70_Alpha*Beta_shell_15 | ATTTCATCTTCTGACCTAATCATCCGGAAATTTAATGGTTTGATTTTT | |
| hh70_Alpha*Beta_shell_16 | AATAAGAGTAATTGGCTTAATTGAGAA | |
| hh70_Alpha*Beta_shell_17 | TCGCCATGGCATTTTCGAGCCAGAATAT | |
| hh70_Alpha*Beta_passive_Alpha*_01 | CCTGTAGAAAGTACAGCTAATGCAGAACGCGCCTTTTT | |
| hh70_Alpha*Beta_passive_Alpha*_02 | ATTAAATACGTTAATGATAAATAAGGCGTTAAATTTTT | |
| hh70_Alpha*Beta_passive_Alpha*_03 | TTTTTTGTTTATCAACACTAAGAACACCCAG | |
| hh70_Alpha*Beta_passive_Alpha*_04 | TTTTTTAAGAATAAACAAATTACT | |
| hh70_Alpha*Beta_2nt_intrusion_Alpha*_01 | CCTGTAGAAAGTACAGCTAATGCAGAACGCG | |
| hh70_Alpha*Beta_2nt_intrusion_Alpha*_02 | ATTAAATACGTTAATGATAAATAAGGCGTTA | |
| hh70_Alpha*Beta_2nt_intrusion_Alpha*_03 | TTTATCAACACTAAGAACACCCAG | |
| hh70_Alpha*Beta_2nt_intrusion_Alpha*_04 | AGAATAAACAAATTACT | |
| hh70_Alpha*Beta_passive_Beta_01 | CGACAAAAGGCAGAATTTAACAACGCCAACTTTTT | |
| hh70_Alpha*Beta_passive_Beta_02 | TTTTTATGTAATTTAGGTAAAACCAAGTACCGTT | |
| hh70_Alpha*Beta_passive_Beta_03 | TTTTTCAACGCTCATAGGTCTGTGGGAACAAACGG | |
| hh70_Alpha*Beta_passive_Beta_04 | ACAGTAGCTTACCAGTATAAAGCTTTTT | |
| hh70_Alpha*Beta_2nt_intrusion_Beta_01 | CCCAACGCTCATAGGTCTGTGGGAACAAACGG | |
| hh70_Alpha*Beta_2nt_intrusion_Beta_02 | CTATGTAATTTAGGTAAAACCAAGTACCGTT | |
| hh70_Alpha*Beta_2nt_intrusion_Beta_03 | CGACAAAAGGCAGAATTTAACAACGCCAACTT | |
| hh70_Alpha*Beta_2nt_intrusion_Beta_04 | ACAGTAGCTTACCAGTATAAAGCGC | |

**Table S8: Staples for HH75 modular part in configuration 1:** Staples also used in other configurations are printed in red, and to the right the other configuration is marked with a "y".

| name | sequence 5* -> 3* | δεζ |
|---|---|---|
| hh75_BetaGamma*_shell_01 | TTTTTGCTACGGCGCCTGAGCAATTTTT | y |
| hh75_BetaGamma*_shell_02 | TCACGACTTGGGTAACGCCAGATTATTT | y |
| hh75_BetaGamma*_shell_03 | TTCGCTATGGCGAACGGATTCGCCTGATTGCTTTTTGAATTACCCAGC | y |
| hh75_BetaGamma*_shell_04 | GGCAAAGCCAGAAGGATAGAAAGGGTTGATGGCAATTCATTTTTT | y |
| hh75_BetaGamma*_shell_05 | TCGGTGCTGAAAACATAGCGATAGCTTAGTTGGGATTGTCGG | |
| hh75_BetaGamma*_shell_06 | TTTTTATATTCCTGATTATCAGAGCGGAATTATCATCTTTTT | y |
| hh75_BetaGamma*_shell_07 | TATCAAAGGACAAACGGATTTTCCCAG | y |
| hh75_BetaGamma*_shell_08 | GCACGTAAAACAGAGATTAAGGTTGTAATAGACTT | |
| hh75_BetaGamma*_shell_09 | TTTTTAAGAAGATGATGAAACTCAATTA | y |
| hh75_BetaGamma*_shell_10 | TTTTTCAATATAATCCTTGAAATTGTTATTTTTT | y |
| | | |
| hh75_BetaGamma*_passive_Beta_01 | ATTCTCCGAGAGACTCCCTTAGTACCTTTTACATTTTT | |
| hh75_BetaGamma*_passive_Beta_02 | TTTTTAGATGAATATACAGTAACAGAATCCTGGGCCTC | |
| hh75_BetaGamma*_passive_Beta_03 | TTTTTTCGGGAGAAACAATTTTTCGTAGAAAAGGGGGACCAAGCTCATTTGA | |
| hh75_BetaGamma*_passive_Beta_04 | AATAAAGAAATTGCAGGTTTAACGTCTTTTT | |
| hh75_BetaGamma*_2nt_intrusion_Beta_01 | ATTCTCCGAGAGACTCCCTTAGTACCTTTTA | |
| hh75_BetaGamma*_2nt_intrusion_Beta_02 | TCAGATGAATATACAGTAACAGAATCCTGGGCCTC | |
| hh75_BetaGamma*_2nt_intrusion_Beta_03 | GGGAGAAACAATTTTTCGTAGAAAAGGGGGACCAAGCTCATTTGA | |
| hh75_BetaGamma*_2nt_intrusion_Beta_04 | AATAAAGAAATTGCAGGTTTAACG | |
| hh75_BetaGamma*_passive_Gamma*_01 | TTACGCTTTTTTAATGGAAAATTTCATGAATACCATCGCGCAGTTTTT | y |
| hh75_BetaGamma*_passive_Gamma*_02 | TTTTTCTGAATAATGGACCTACCA | y |
| hh75_BetaGamma*_passive_Gamma*_03 | TTTTTAGGCGAATTATTCATTAAACAAAAAGTTACATCAAGAAAACATTTTT | y |
| hh75_BetaGamma*_passive_Gamma*_04 | GATTGTTTGGATTATACTTTTTTT | y |
| hh75_BetaGamma*_2nt_intrusion_Gamma*_01 | TTACGCTTTTTTAATGGAAAATTTCATGAATACCATCGCGC | y |
| hh75_BetaGamma*_2nt_intrusion_Gamma*_02 | GAATAATGGACCTACCA | y |
| hh75_BetaGamma*_2nt_intrusion_Gamma*_03 | GCGAATTATTCATTAAACAAAAAGTTACATCAAGAAAACATTTTT | y |
| hh75_BetaGamma*_2nt_intrusion_Gamma*_04 | GATTGTTTGGATTATAC | y |

**Table S9: Staples for HH50 modular part in configuration 2:** Staples also used in other configurations are printed in red, and to the right the other configuration is marked with a “y”. Some staples from configuration 1 are also needed, *cf.* Table S3

| name | sequence 5* -> 3* | γ*δ | ζ*α |
|---|---|---|---|
| hh50_Zeta*Delta_shell_01 | TAATCATGGTCATAGCCGGAGTAAAGC | | |
| **hh50_Zeta*Delta_shell_02** | **CGAGCTCGTACAAAGGTGGAAACGATACTTAAAGTAGCATGCATCTACG** | | **y** |
| hh50_Zeta*Delta_shell_03 | GTACAACGGAGATTTAGCGAGAGGCTTTCGACGAT | | |
| hh50_Zeta*Delta_shell_04 | TCCAATATAACGCCCGCAGTTTTCATATTTTAAGA | | |
| hh50_Zeta*Delta_shell_05 | TACCAGATGCAAAAGAAGTTTTGAGCAATTTTCAC | | |
| hh50_Zeta*Delta_shell_06 | TTTTTGAACGGTGTACAGACCAGATTTGAAAGAGGACAGATTTTTT | | |
| **hh50_Zeta*Delta_shell_07** | **TTAATAAACTGGCTGAATTACCTTATG** | | **y** |
| hh50_Zeta*Delta_shell_08 | TATTCATTACCCAAATCAACGTAACAAAGCTGCTCCCTCGTT | | |
| hh50_Zeta*Delta_shell_09 | TTTTTACCAACTCTTGACAAGTAAGGGAATTCTGC | | |
| hh50_Zeta*Delta_shell_10 | GCTTGAATAAGAGCAACACTAAGGGGGTCCAGCGA | | |
| hh50_Zeta*Delta_shell_11 | TTTCTTTAGATCCGAACGAGGTCAATCAAACCGGA | | |
| hh50_Zeta*Delta_shell_12 | CATACATTAGAGTCTGCCAGTCATAACATTCAGTGAATAAG | | |
| | | | |
| hh50_Zeta*Delta_passive_Zeta*_01 | AATGTGCCACTCGCGGCTGGCTGTTTTT | | |
| hh50_Zeta*Delta_passive_Zeta*_02 | ATTACGAGGCATAGTCATTGTCATTATACCAGTCAGGACGTTTTT | | |
| hh50_Zeta*Delta_passive_Zeta*_03 | TTTTTTTGGGAAGAAAACCTCTCTGAATTCG | | |
| hh50_Zeta*Delta_passive_Zeta*_04 | TTTTTACCTTCATCAAGAGTAGCGCATAGGGGATTTTTTATGGAGATGA | | |
| hh50_Zeta*Delta_2nt_intrusion_Zeta*_01 | AATGTGCCACTCGCGGCTGGCTGAC | | |
| hh50_Zeta*Delta_2nt_intrusion_Zeta*_02 | ATTACGAGGCATAGTCATTGTCATTATACCAGTCAGGACGGT | | |
| hh50_Zeta*Delta_2nt_intrusion_Zeta*_03 | CCACCTTCATCAAGAGTAGCGCATAGGGGATTTTTTATGGAGATGA | | |
| hh50_Zeta*Delta_2nt_intrusion_Zeta*_04 | AGTTGGGAAGAAAACCTCTCTGAATTCG | | |
| hh50_Zeta*Delta_passive_Delta_01 | ATCAGTTTTAAACTGCTTGAGATGGTTTTTT | | |
| hh50_Zeta*Delta_passive_Delta_02 | TTTTTTTAATTTCAACTTTCCCTGACGAGAAACACCTTTTT | | |
| hh50_Zeta*Delta_passive_Delta_03 | TTTTTAGAACGAGTAGTAAATTGGTTGACCCAATAGTAGAGTATC | | |
| hh50_Zeta*Delta_2nt_intrusion_Delta_01 | ATCAGTTTTAAACTGCTTGAGATGGTGA | | |
| hh50_Zeta*Delta_2nt_intrusion_Delta_02 | TAAGAACGAGTAGTAAATTGGTTGACCCAATAGTAGAGTATC | | |
| hh50_Zeta*Delta_2nt_intrusion_Delta_03 | AATTAATTTCAACTTTCCCTGACGAGAAACACCGA | | |

**Table S10: Staples for HH55 modular part in configuration 2:** Some staples from configuration 1 are also needed, *cf.* Table S4

| name | sequence 5* -> 3* |
|---|---|
| hh55_DeltaEpsilon*_shell_01 | ACCAGGCGGATAATCAGAAC |
| hh55_DeltaEpsilon*_shell_02 | GTTGATACACCCTCAGAACCGCCACCCGTGCCGT |
| hh55_DeltaEpsilon*_shell_03 | GTTTGCTTTTAATTCGTCACTTCAGCG |
| hh55_DeltaEpsilon*_shell_04 | AAGGCACCGAGCTTCAAAGACGACTAAAAGCTTGATGCGCCG |
| hh55_DeltaEpsilon*_shell_05 | AAAGAATTTATACCAAGCGCGAAACAAAAAACGAAAAAAAAG |
| hh55_DeltaEpsilon*_shell_06 | ATCAGCTTGTGCGAGAATCTCTCCAACTTGCGGA |
| hh55_DeltaEpsilon*_shell_07 | GAGTGAGAATAGAAGAGTACCCCTTTTG |
| hh55_DeltaEpsilon*_shell_08 | ACAATGATCGGTCGCTGAGGCTTGCAGGCTATAGTTACCGCA |
| hh55_DeltaEpsilon*_shell_09 | CTATAGTCCCTCTTCAACAGTCAGTACATACCGTA |
| | |
| hh55_DeltaEpsilon*_passive_Epsilon_01 | TTTTTTCACGTTGAAAATCTCCAAAGAGGCATCAAAT |
| hh55_DeltaEpsilon*_passive_Epsilon_02 | TTTTTTTAATTGTACTTAAACGCGAACCAGACCGGTTTAATTCAACCTA |
| hh55_DeltaEpsilon*_passive_Epsilon_03 | TGAATTTTCGGTTTGCTCCAAAAGGAGCCTTTTTT |
| hh55_DeltaEpsilon*_passive_Epsilon_04 | AGGAACAACTAAAGATAATAATTTTTTTTTT |
| hh55_DeltaEpsilon*_2nt_intrusion_Epsilon_01 | TGAATTTTCGGTTTGCTCCAAAAGGAGCCTCA |
| hh55_DeltaEpsilon*_2nt_intrusion_Epsilon_02 | AGGAACAACTAAAGATAATAATTTTTCG |
| hh55_DeltaEpsilon*_2nt_intrusion_Epsilon_03 | AATTAATTGTACTTAAACGCGAACCAGACCGGTTTAATTCAACCTA |
| hh55_DeltaEpsilon*_2nt_intrusion_Epsilon_04 | AGTCACGTTGAAAATCTCCAAAGAGGCATCAAAT |
| hh55_DeltaEpsilon*_passive_Delta*_02 | ATCGCGTAAGCAAATTCGAGGCATCGCCCATTTTT |
| hh55_DeltaEpsilon*_passive_Delta*_03 | TTTTTGGATTTTGCTAAACAAATGAATT |
| hh55_DeltaEpsilon*_passive_Delta*_04 | TTTTTCGCATAACCGATATATCAACAAC |
| hh55_DeltaEpsilon*_2nt_intrusion_Delta*_01 | ACACTGACGCCACCTTCTGTATGTA |
| hh55_DeltaEpsilon*_2nt_intrusion_Delta*_02 | ATCGCGTAAGCAAATTCGAGGCATCGCC |
| hh55_DeltaEpsilon*_2nt_intrusion_Delta*_03 | ATTTTGCTAAACAAATGAATT |
| hh55_DeltaEpsilon*_2nt_intrusion_Delta*_04 | ATCGCATAACCGATATATCAACAAC |

**Table S11: Staples for HH60 modular part in configuration 2:** Some staples from configuration 1 are also needed, cf. Table S5

| name | sequence 5* -> 3* |
|---|---|
| hh60_Epsilon*Zeta_shell_01 | TTACCATTAGCAAGCAATAAAGCCTCAGAGCATAA |
| hh60_Epsilon*Zeta_shell_02 | AATGAAATATTCGGTGGCATC |
| hh60_Epsilon*Zeta_shell_03 | GCCAGAATGGAAAGCGCAGTCAACCTATCATGAAA |
| | |
| hh60_Epsilon*Zeta_passive_Epsilon*_04 | TTTTTGCCTCCCTCAGACCACCACCCTCACA |
| hh60_Epsilon*Zeta_2nt_intrusion_Epsilon*_04 | CTCCCTCAGACCACCACCCTCACA |
| hh60_Epsilon*Zeta_passive_Zeta_01 | AACAAATAAATCCTTTGGCCTCAGGAGGTTGAGGCAGTTTTT |
| hh60_Epsilon*Zeta_passive_Zeta_02 | TTTTTGTCAGACGACATTAAAAATTCTA |
| hh60_Epsilon*Zeta_passive_Zeta_03 | GCATTGATGATATTCAGAGCCGCCACCAGAACCTTTTT |
| hh60_Epsilon*Zeta_passive_Zeta_04 | TTTTTACCACCAGAGCCGCCGCCACAATGAACAAAGAATTAGCAAAATTAAG |
| hh60_Epsilon*Zeta_2nt_intrusion_Zeta_01 | AACAAATAAATCCTTTGGCCTCAGGAGGTTGAGGC |
| hh60_Epsilon*Zeta_2nt_intrusion_Zeta_02 | CAGACGACATTAAAAATTCTA |
| hh60_Epsilon*Zeta_2nt_intrusion_Zeta_03 | GCATTGATGATATTCAGAGCCGCCACCAGAA |
| hh60_Epsilon*Zeta_2nt_intrusion_Zeta_04 | CACCAGAGCCGCCGCCACAATGAACAAAGAATTAGCAAAATTAAG |

**Table S12: Staples for HH70 modular part in configuration 2:** Some staples from configuration 1 are also needed, *cf.* Table 7

| name | sequence 5* -> 3* |
|---|---|
| hh70_Delta*Epsilon_shell_01 | TGAAAGCCGTCTGGCGGTATTCTAAGAACACCCAG |
| hh70_Delta*Epsilon_shell_02 | TTGGTAACATAGTCGCTATCCCTCATTTTTGCGGGTAAGAAT |
| hh70_Delta*Epsilon_shell_03 | TTAACCTCGCAAGACGTCGGAACCCAAAGGGCTT |
| hh70_Delta*Epsilon_shell_04 | TCATCAACATTAAAATATTTA |
| hh70_Delta*Epsilon_shell_05 | ATTAAATACGTTAATGTGATAAATAAGGCGTTAAA |
| hh70_Delta*Epsilon_shell_06 | ACAAGCAATCAGATATAGAAGGCTTATCCCCACTCATCGAGA |
| hh70_Delta*Epsilon_shell_07 | AAACACCTATCATAAATTGAGAATCGCCTGTGAGCAATAGGA |
| hh70_Delta*Epsilon_shell_08 | TTTTTAAATACCGACCGTATTTTGTTTTTTT |
| hh70_Delta*Epsilon_shell_09 | TGCGTTATACAAATTCTTACCCAACGCT |
| hh70_Delta*Epsilon_shell_10 | CAACAGTAGAACGCGAGAAAAATCCAATCCGGCTT |
| hh70_Delta*Epsilon_shell_11 | TTTTTATTTCATCTTCTGACCTAAAAGCAGTATAATTTAATGGTTTGTTTTT |
| hh70_Delta*Epsilon_shell_12 | ATAGAATCGGCTGTCTTTCTTTTT |
| hh70_Delta*Epsilon_shell_13 | ACACATAAACAAAGAGCCAGTAATAAG |
| hh70_Delta*Epsilon_shell_14 | AGAATATCAGACGACGACAATGTTCAGC |
| | |
| hh70_Delta*Epsilon_passive_Delta*_01 | AGAACGGGTATTAAATCAACACCTGAACAATTTTT |
| hh70_Delta*Epsilon_passive_Delta*_02 | ACGCCATCGTTTTATAATTACTATTTTT |
| hh70_Delta*Epsilon_passive_Delta*_03 | TTTTTGAAAAATAATATCCCATATAAGT |
| hh70_Delta*Epsilon_passive_Delta*_04 | TTTTTGAAAAAGCCTGTTTAGGGAATCAGCGAACCTCAAGATGAACGGT |
| hh70_Delta*Epsilon_2nt_intrusion_Delta*_01 | AGAACGGGTATTAAATCAACACCTGAAC |
| hh70_Delta*Epsilon_2nt_intrusion_Delta*_02 | ACGCCATCGTTTTATAATTAC |
| hh70_Delta*Epsilon_2nt_intrusion_Delta*_03 | AAAATAATATCCCATATAAGT |
| hh70_Delta*Epsilon_2nt_intrusion_Delta*_04 | AAAAGCCTGTTTAGGGAATCAGCGAACCTCAAGATGAACGGT |
| hh70_Delta*Epsilon_passive_Epsilon_01 | GAACGCGTTCTGTCAAAGTACCGACAAAAGTTTTT |
| hh70_Delta*Epsilon_passive_Epsilon_02 | TTTTTGTAAAGTAACCTGTTTACCAAGTACCGTTCCTGTAGTAATGCA |
| hh70_Delta*Epsilon_passive_Epsilon_03 | TTTTTAGGCAGAGGTAGGTCTGTGGGAACAAACGG |
| hh70_Delta*Epsilon_passive_Epsilon_04 | CATTTTCCGCCAACATGTAATTTTTTTT |
| hh70_Delta*Epsilon_2nt_intrusion_Epsilon_01 | CCAGGCAGAGGTAGGTCTGTGGGAACAAACGG |
| hh70_Delta*Epsilon_2nt_intrusion_Epsilon_02 | CTGTAAAGTAACCTGTTTACCAAGTACCGTTCCTGTAGTAATGCA |
| hh70_Delta*Epsilon_2nt_intrusion_Epsilon_03 | GAACGCGTTCTGTCAAAGTACCGACAAAAGTT |
| hh70_Delta*Epsilon_2nt_intrusion_Epsilon_04 | CATTTTCCGCCAACATGTAATTTGC |

**Table S13: Staples for HH75 modular part in configuration 2:** Some staples from configuration 1 are also needed, *cf.* Table S8

| **name** | **sequence 5* -> 3*** |
|---|---|
| hh75_EpsilonZeta*_shell_01 | ATTCTCCGAGAGACTCCCTTAGAATCCTGGGCCTC |
| hh75_EpsilonZeta*_shell_02 | TTACATCGGGAGAAACAATAAAGGGGGACCAAGCTCATTTGA |
| | |
| hh75_EpsilonZeta*_passive_Epsilon_01 | TCGGTGCTGAAAACATAGCGATCAGATGAATATTTTTT |
| hh75_EpsilonZeta*_passive_Epsilon_02 | TTTTTTAGATTTTCAGGTTTAACGTAGCTTAGTTGGGATTGTCGG |
| hh75_EpsilonZeta*_passive_Epsilon_03 | TTTTTACAGTAACAGTACCGAAATAAACATTGATTAAGGTTGTAATAGACTT |
| hh75_EpsilonZeta*_passive_Epsilon_04 | GCACGTAAAAGAAATTGCGTTTTT |
| hh75_EpsilonZeta*_2nt_intrusion_Epsilon_01 | TCGGTGCTGAAAACATAGCGATCAGATGAAT |
| hh75_EpsilonZeta*_2nt_intrusion_Epsilon_02 | CAACAGTAACAGTACCGAAATAAACATTGATTAAGGTTGTAATAGACTT |
| hh75_EpsilonZeta*_2nt_intrusion_Epsilon_03 | GATTTTCAGGTTTAACGTAGCTTAGTTGGGATTGTCGG |
| hh75_EpsilonZeta*_2nt_intrusion_Epsilon_04 | GCACGTAAAAGAAATTGCGGG |

**Table S14: Staples for cross-configurations of HH50:** Some staples from configuration 1 and 2 are also needed, *cf.* Table S3 and Table S9

| name | sequence 5* -> 3* |
|---|---|
| hh50_Gamma*Delta_shell_01 | TACCAGATGCAAAAGAAGTTTGAGCAATTTTCA |
| hh50_Gamma*Delta_shell_02 | TTTTTGAACGGTGTACAGACCAGTTTTGAAAGAGGACAGATTTTTT |
| hh50_Gamma*Delta_shell_03 | CCGGATAGCGCATAGGGGATTTTTTATGGAGATGA |
| hh50_Gamma*Delta_shell_04 | AGCTGCTCATTCAGTGAATAAGGCTTGCCCTCGTT |
| hh50_Gamma*Delta_shell_05 | TTTTTACCAACTCATTACCCATAAGGGAATTCTGC |
| hh50_Gamma*Delta_shell_06 | CCAGATGTAAGAGCAACACTAAGGGGGTCCAGCGA |
| hh50_Gamma*Delta_shell_07 | TTTCTTTAGATCCGAACGAGGTCAATCAAATCAACACAAGAA |
| hh50_Gamma*Delta_shell_08 | CATACATTAGAGTCTTGCCAGTCATAACCCTGACGAGAAACA |
| | |
| hh50_Gamma*Delta_passive_Gamma*_01 | AATGTGCCACTCGCGGCTGGCTGACCTTCATCATTTTT |
| hh50_Gamma*Delta_passive_Gamma*_04 | TTTTTAGAGTAATCTTGGTAACAA |
| hh50_Gamma*Delta_2nt_intrusion_Gamma*_01 | AATGTGCCACTCGCGGCTGGCTGACCTTCATCAGC |
| hh50_Gamma*Delta_2nt_intrusion_Gamma*_02 | ATTACGAGGCATAGCGATTTTTGGGAAGAAGC |
| hh50_Gamma*Delta_2nt_intrusion_Gamma*_03 | TGAAATCTACGTTAATAAAGGACGTAAGAACTGGCTCA |
| hh50_Gamma*Delta_2nt_intrusion_Gamma*_04 | CCAGAGTAATCTTGGTAACAA |
| hh50_Gamma*Delta_passive_Delta_01 | ATCAGTTTTAAACTAACTTTAATCATTTTTT |
| hh50_Gamma*Delta_passive_Delta_02 | TTTTTTGTGAATTACCTTAACGAGTAGTAAATTGGGTTTTT |
| hh50_Gamma*Delta_passive_Delta_03 | TTTTTCTTGAGATGGTTTAATTTCTTGACCCAATAGTAGAGTATC |
| hh50_Gamma*Delta_2nt_intrusion_Delta_01 | ATCAGTTTTAAACTAACTTTAATCATGA |
| hh50_Gamma*Delta_2nt_intrusion_Delta_02 | AATGTGAATTACCTTAACGAGTAGTAAATTGGGGA |
| hh50_Gamma*Delta_2nt_intrusion_Delta_03 | TACTTGAGATGGTTTAATTTCTTGACCCAATAGTAGAGTATC |
| | |
| hh50_Zeta*Alpha_shell_01 | TACCAGATGCAAAAGAAGTTTGAGCAATTTTCA |
| hh50_Zeta*Alpha_shell_02 | TTTTTGAACGGTGTACAGACCTATTGAAAGAGGACAGATTTTTT |
| hh50_Zeta*Alpha_shell_03 | GATATTCATTACCCAAATCAACGATTGGGCTTGAGCCTCGTT |
| hh50_Zeta*Alpha_shell_04 | TTTTTACCAACTATCTTGACATAAGGGAATTCTGC |
| hh50_Zeta*Alpha_shell_05 | ACTTTAATAAGAGCAACACTAAGGGGGTCCAGCGA |
| hh50_Zeta*Alpha_shell_06 | TTTCTTTAGATCCGAACGAGGTCAATCAAGAACCG |
| hh50_Zeta*Alpha_shell_07 | CATACATTAGAGTCTTGCCAGTCATAACATGGTTTAATTTCA |
| | |
| hh50_Zeta*Alpha_passive_Zeta*_01 | AATGTGCCACTCGCTAGGCTGGCTTTTT |
| hh50_Zeta*Alpha_passive_Zeta*_04 | TTTTTGACCTTCATCAAGAGAGGCGCAGGGGATTTTTTATGGAGATGA |
| hh50_Zeta*Alpha_2nt_intrusion_Zeta*_01 | AATGTGCCACTCGCTAGGCTGGCAC |
| hh50_Zeta*Alpha_2nt_intrusion_Zeta*_02 | ATTACGAGGCATAGTCATTGTCATTATACCAGTCAGGACGGT |
| hh50_Zeta*Alpha_2nt_intrusion_Zeta*_03 | AGTTGGGAAGAAAACCTCTCTGAATTCG |
| hh50_Zeta*Alpha_2nt_intrusion_Zeta*_04 | CCTGACCTTCATCAAGAGAGGCGCAGGGGATTTTTTATGGAGATGA |
| hh50_Zeta*Alpha_passive_Alpha_01 | GTACAACGGCTTGCCCTGACGAGAAACACCTTTTT |
| hh50_Zeta*Alpha_passive_Alpha_02 | TTTTTAGAACGAGTAGTAATAACAAAGCTGCTCATTCTTTTT |
| hh50_Zeta*Alpha_passive_Alpha_03 | TTTTTAGTGAATAAGGAGATTTAGCGAGAGGCTTTCGACGAT |
| hh50_Zeta*Alpha_2nt_intrusion_Alpha_01 | GTACAACGGCTTGCCCTGACGAGAAACACCTG |
| hh50_Zeta*Alpha_2nt_intrusion_Alpha_02 | CCAGAACGAGTAGTAATAACAAAGCTGCTCATTCTA |
| hh50_Zeta*Alpha_2nt_intrusion_Alpha_03 | AAAGTGAATAAGGAGATTTAGCGAGAGGCTTTCGACGAT |

**Table S15: z-connections staples** of all 4 configurations for the left and right attachment sites and the respective connectors with and without toeholds and the invaders

| name | sequence 5' -> 3' |
|---|---|
| left_passive_01 | TTTTTGACAGGAACGGTACGCGATTAAAGGGATTTTATTTTT |
| left_passive_02 | TTTTTGGAAATACCTACATTTTGAGACCAGTAATTTTT |
| left_passive_03 | TTTTTAACACCGCCTGCCCCTCAATCTTTTT |
| left_passive_04 | TTTTTAATATCTGGTCAGTTGTATCAAA |
| left_passive_05 | TTTTTTGGTTTGCCCCAGCAGAGCAAGCGGTCCACGCTTTTT |
| left_passive_06 | TTTTTCAGTTTGGAACAAGAGGGGTTGAGTGTTGTTCTTTTT |
| right_passive_01 | TGTTCGCCACTGGTGACCTGGAAGAGTTTTT |
| right_passive_02 | TTTTTTGACGACTGGGGATTTCAGAGCAGGCAATGCATTTTT |
| right_passive_03 | TTTTTTGAACCACCAGGCTATATCATATATGTGTTTTT |
| right_passive_04 | GCAATAAAAATGCGCCGCCTTTTT |
| right_passive_05 | TTTTTACATCGGGTTGATGCAGACATCACGAAGGTGTTTTT |
| right_passive_06 | AGATGATGACCGTACTCAATTTTT |
| | |
| zI_left_5'_01 | TGTAGTAATGGACAGGAACGGTACGCGATTAAAGGGATTTTATTT |
| zI_left_5'_02 | TGTAGTAATGGGAAATACCTACATTTTGAGACCAGTAATTTTT |
| zI_left_5'_03 | TGTAGTAATGAACACCGCCTGCCCCTCAATCTTT |
| zI_left_5'_04 | TGTAGTAATGAATATCTGGTCAGTTGTATCAAA |
| zI_left_5'_05 | TGTAGTAATGTGGTTTGCCCCAGCAGAGCAAGCGGTCCACGCTTT |
| zI_left_5'_06 | TGTAGTAATGCAGTTTGGAACAAGAGGGGTTGAGTGTTGTTCTTT |
| zI_right_3'_01 | TGTTCGCCACTGGTGACCTGGAAGAGGTGGTAGTAGA |
| zI_right_3'_02 | TTTGAAACCAGTTTCTTGTGAAGTCCGTGAAGACGGTGGTAGTAGA |
| zI_right_3'_03 | TTTTGACGACTGGGGATTTCAGAGCAGGCAATGCAGTGGTAGTAGA |
| zI_right_3'_04 | GCAATAAAAATGCGCCGCCGTGGTAGTAGA |
| zI_right_3'_05 | TTTACATCGGGTTGATGCAGACATCACGAAGGTGGTGGTAGTAGA |
| zI_right_3'_06 | AGATGATGACCGTACTCAAGTGGTAGTAGA |
| | |
| zII_left_5'_01 | AGTAGATTGAGACAGGAACGGTACGCGATTAAAGGGATTTTATTT |
| zII_left_5'_02 | AGTAGATTGAGGAAATACCTACATTTTGAGACCAGTAATTTTT |
| zII_left_5'_03 | AGTAGATTGAAACACCGCCTGCCCCTCAATCTTT |
| zII_left_5'_04 | AGTAGATTGAAATATCTGGTCAGTTGTATCAAA |
| zII_left_5'_05 | AGTAGATTGATGGTTTGCCCCAGCAGAGCAAGCGGTCCACGCTTT |
| zII_left_5'_06 | AGTAGATTGACAGTTTGGAACAAGAGGGGTTGAGTGTTGTTCTTT |
| zII_right_3'_01 | TGTTCGCCACTGGTGACCTGGAAGAGGTAGTAGTGAT |
| zII_right_3'_02 | TTTGAAACCAGTTTCTTGTGAAGTCCGTGAAGACGGTAGTAGTGAT |
| zII_right_3'_03 | TTTTGACGACTGGGGATTTCAGAGCAGGCAATGCAGTAGTAGTGAT |
| zII_right_3'_04 | GCAATAAAAATGCGCCGCCGTAGTAGTGAT |
| zII_right_3'_05 | TTTACATCGGGTTGATGCAGACATCACGAAGGTGGTAGTAGTGAT |
| zII_right_3'_06 | AGATGATGACCGTACTCAAGTAGTAGTGAT |
| | |
| zIII_left_5'_01 | GTTAGAAGTGGACAGGAACGGTACGCGATTAAAGGGATTTTATTT |
| zIII_left_5'_02 | GTTAGAAGTGGGAAATACCTACATTTTGAGACCAGTAATTTTT |
| zIII_left_5'_03 | GTTAGAAGTGAACACCGCCTGCCCCTCAATCTTT |
| zIII_left_5'_04 | GTTAGAAGTGAATATCTGGTCAGTTGTATCAAA |
| zIII_left_5'_05 | GTTAGAAGTGTGGTTTGCCCCAGCAGAGCAAGCGGTCCACGCTTT |
| zIII_left_5'_06 | GTTAGAAGTGCAGTTTGGAACAAGAGGGGTTGAGTGTTGTTCTTT |
| zIII_right_3'_01 | TGTTCGCCACTGGTGACCTGGAAGAGGATGGGAAGAT |
| zIII_right_3'_02 | TTTGAAACCAGTTTCTTGTGAAGTCCGTGAAGACGGATGGGAAGAT |
| zIII_right_3'_03 | TTTTGACGACTGGGGATTTCAGAGCAGGCAATGCAGATGGGAAGAT |
| zIII_right_3'_04 | GCAATAAAAATGCGCCGCCGATGGGAAGAT |
| zIII_right_3'_05 | TTTACATCGGGTTGATGCAGACATCACGAAGGTGGATGGGAAGAT |
| zIII_right_3'_06 | AGATGATGACCGTACTCAAGATGGGAAGAT |

| | |
|---|---|
| zIV_left_5’_01 | TGAGGTAGAAGACAGGAACGGTACGCGATTAAAGGGATTTTATTT |
| zIV_left_5’_02 | TGAGGTAGAAGGAAATACCTACATTTTGAGACCAGTAATTTTT |
| zIV_left_5’_03 | TGAGGTAGAAAACACCGCCTGCCCCTCAATCTTT |
| zIV_left_5’_04 | TGAGGTAGAAAATATCTGGTCAGTTGTATCAAA |
| zIV_left_5’_05 | TGAGGTAGAATGGTTTGCCCCAGCAGAGCAAGCGGTCCACGCTTT |
| zIV_left_5’_06 | TGAGGTAGAACAGTTTGGAACAAGAGGGGTTGAGTGTTGTTCTTT |
| zIV_right_3’_01 | TGTTCGCCACTGGTGACCTGGAAGAGGTAAAGAGATA |
| zIV_right_3’_02 | TTTGAAACCAGTTTCTTGTGAAGTCCGTGAAGACGGTAAAGAGATA |
| zIV_right_3’_03 | TTTTGACGACTGGGGATTTCAGAGCAGGCAATGCAGTAAAGAGATA |
| zIV_right_3’_04 | GCAATAAAAATGCGCCGCCGTAAAGAGATA |
| zIV_right_3’_05 | TTTACATCGGGTTGATGCAGACATCACGAAGGTGGTAAAGAGATA |
| zIV_right_3’_06 | AGATGATGACCGTACTCAAGTAAAGAGATA |
| | |
| zI_left_3’_01 | CAGAATCCAACAGGAAAAACGCTCATGTGGTAGTAGA |
| zI_left_3’_02 | TTTGACAGGAACGGTACGCGATTAAAGGGATTTTAGTGGTAGTAGA |
| zI_left_3’_03 | AGGTGAGGCGGTCAGTATTGTGGTAGTAGA |
| zI_left_3’_04 | TTTAACACCGCCTGCCCCTCAATCGTGGTAGTAGA |
| zI_left_3’_05 | TTTTGGTTTGCCCCAGCAGAGCAAGCGGTCCACGCGTGGTAGTAGA |
| zI_left_3’_06 | TTTCAGTTTGGAACAAGAGGGGTTGAGTGTTGTTCGTGGTAGTAGA |
| zI_right_5’_01 | TGTAGTAATGTTTCTGCGGCAGTTAATCGGTGAAAATGTTTTT |
| zI_ right_5’_02 | TGTAGTAATGGAAACCAGTTTCTTGTGAAGTCCGTGAAGACGTTT |
| zI_ right_5’_03 | TGTAGTAATGTGACGACTGGGGATTTCAGAGCAGGCAATGCATTT |
| zI_ right_5’_04 | TGTAGTAATGTGAACCACCAGGCTATATCATATATGTGTTTTT |
| zI_ right_5’_05 | TGTAGTAATGTTTATGTAGATGAAGGTAT |
| zI_ right_5’_06 | TGTAGTAATGACATCGGGTTGATGCAGACATCACGAAGGTGTTT |
| | |
| zII_left_3’_01 | CAGAATCCAACAGGAAAAACGCTCATGTAGTAGTGAT |
| zII_left_3’_02 | TTTGACAGGAACGGTACGCGATTAAAGGGATTTTAGTAGTAGTGAT |
| zII_left_3’_03 | AGGTGAGGCGGTCAGTATTGTAGTAGTGAT |
| zII_left_3’_04 | TTTAACACCGCCTGCCCCTCAATCGTAGTAGTGAT |
| zII_left_3’_05 | TTTTGGTTTGCCCCAGCAGAGCAAGCGGTCCACGCGTAGTAGTGAT |
| zII_left_3’_06 | TTTCAGTTTGGAACAAGAGGGGTTGAGTGTTGTTCGTAGTAGTGAT |
| zII_right_5’_01 | AGTAGATTGATTTCTGCGGCAGTTAATCGGTGAAAATGTTTTT |
| zII_ right_5’_02 | AGTAGATTGAGAAACCAGTTTCTTGTGAAGTCCGTGAAGACGTTT |
| zII_ right_5’_03 | AGTAGATTGATGACGACTGGGGATTTCAGAGCAGGCAATGCATTT |
| zII_ right_5’_04 | AGTAGATTGATGAACCACCAGGCTATATCATATATGTGTTTTT |
| zII_ right_5’_05 | AGTAGATTGATTTATGTAGATGAAGGTAT |
| zII_ right_5’_06 | AGTAGATTGAACATCGGGTTGATGCAGACATCACGAAGGTGTTT |
| | |
| zIII_left_3’_01 | CAGAATCCAACAGGAAAAACGCTCATGATGGGAAGAT |
| zIII_left_3’_02 | TTTGACAGGAACGGTACGCGATTAAAGGGATTTTAGATGGGAAGAT |
| zIII_left_3’_03 | AGGTGAGGCGGTCAGTATTGATGGGAAGAT |
| zIII_left_3’_04 | TTTAACACCGCCTGCCCCTCAATCGATGGGAAGAT |
| zIII_left_3’_05 | TTTTGGTTTGCCCCAGCAGAGCAAGCGGTCCACGCGATGGGAAGAT |
| zIII_left_3’_06 | TTTCAGTTTGGAACAAGAGGGGTTGAGTGTTGTTCGATGGGAAGAT |
| zIII_right_5’_01 | GTTAGAAGTGTTTCTGCGGCAGTTAATCGGTGAAAATGTTTTT |
| zIII_ right_5’_02 | GTTAGAAGTGGAAACCAGTTTCTTGTGAAGTCCGTGAAGACGTTT |
| zIII_ right_5’_03 | GTTAGAAGTGTGACGACTGGGGATTTCAGAGCAGGCAATGCATTT |
| zIII_ right_5’_04 | GTTAGAAGTGTGAACCACCAGGCTATATCATATATGTGTTTTT |
| zIII_ right_5’_05 | GTTAGAAGTGTTTATGTAGATGAAGGTAT |
| zIII_ right_5’_06 | GTTAGAAGTGACATCGGGTTGATGCAGACATCACGAAGGTGTTT |

| | |
|---|---|
| zIV_left_3’_01 | CAGAATCCAACAGGAAAAACGCTCATGTAAAGAGATA |
| zIV_left_3’_02 | TTTGACAGGAACGGTACGCGATTAAAGGGATTTTAGTAAAGAGATA |
| zIV_left_3’_03 | AGGTGAGGCGGTCAGTATTGTAAAGAGATA |
| zIV_left_3’_04 | TTTAACACCGCCTGCCCCTCAATCGTAAAGAGATA |
| zIV_left_3’_05 | TTTTGGTTTGCCCCAGCAGAGCAAGCGGTCCACGCGTAAAGAGATA |
| zIV_left_3’_06 | TTTCAGTTTGGAACAAGAGGGGTTGAGTGTTGTTCGTAAAGAGATA |
| zIV_right_5’_01 | TGAGGTAGAATTTCTGCGGCAGTTAATCGGTGAAAATGTTTTT |
| zIV_ right_5’_02 | TGAGGTAGAAGAAACCAGTTTCTTGTGAAGTCCGTGAAGACGTTT |
| zIV_ right_5’_03 | TGAGGTAGAATGACGACTGGGGATTTCAGAGCAGGCAATGCATTT |
| zIV_ right_5’_04 | TGAGGTAGAATGAACCACCAGGCTATATCATATATGTGTTTTT |
| zIV_ right_5’_05 | TGAGGTAGAATTTATGTAGATGAAGGTAT |
| zIV_ right_5’_06 | TGAGGTAGAAACATCGGGTTGATGCAGACATCACGAAGGTGTTT |

| | |
|---|---|
| z_connector_I | CATTACTACATCTACTACCAC |
| z_connector_II | TCAATCTACTATCACTACTAC |
| z_connector_III | CACTTCTAACATCTTCCCATC |
| z_connector_IV | TTCTACCTCATATCTCTTTAC |
| z_connector_I-th7 | CTACTATCATTACTACATCTACTACCAC |
| z_connector_II-th7 | ACACTGCTCAATCTACTATCACTACTAC |
| z_connector_III-th7 | CTACCAACACTTCTAACATCTTCCCATC |
| z_connector_IV-th7 | AACTCCATTCTACCTCATATCTCTTTAC |
| z_invader_I-th7 | GTGGTAGTAGATGTAGTAATGATAGTAG |
| z_invader_II-th7 | GTAGTAGTGATAGTAGATTGAGCAGTGT |
| z_invader_III-th7 | GATGGGAAGATGTTAGAAGTGTTGGTAG |
| z_invader_IV-th7 | GTAAAGAGATATGAGGTAGAATGGAGTT |

**Table S16: 4 nt and 6 nt overlap connections** for α and α* sites.

| name | sequence 5' -> 3' |
|---|---|
| hh50_Alpha_4nt_intrusion_01 | AGATTTCGTTTATGCGAGTAGTAAATTGGGTGTT |
| hh50_Alpha_4nt_intrusion_02 | CGCCCTTGAGATGAACTTTACCTCGTTTACCAGA |
| hh50_Alpha_4nt_intrusion_03 | TAAAACGAGAAACGGAGATTTAGCGAGAGGCTTTCGACGAT |
| hh50_Alpha_4nt_intrusion_04 | GTACAACACCAGAAAATAAGGCTTGCCCTGTAAG |
| hh55_Alpha_4nt_intrusion_01 | GAAAATCTCCAAAACAAAAGGAGCCTCGCT |
| hh55_Alpha_4nt_intrusion_02 | TGAGTTAATTGTATCGGTTTATCAGAGGCATCAAAT |
| hh55_Alpha_4nt_intrusion_03 | GCAATTAAACAGCAACCATCGCGAACCAGACCGGTTTAATTCAACCTA |
| hh55_Alpha_4nt_intrusion_04 | TGACAACTTGATACTTTCGAGGTGAATTTCCATA |
| hh65_Alpha*_4nt_intrusion_01 | AATATCAGAGAGTCAGAGG |
| hh65_Alpha*_4nt_intrusion_02 | CATATGAGAGTCTGCTACAATGTAAT |
| hh65_Alpha*_4nt_intrusion_03 | GACATTCTTACCAGTAAATCAGTCACCATAAAGGTG |
| hh65_Alpha*_4nt_intrusion_04 | TAAAAGAAATAAGCAATTGTAATTTTGTT |
| hh70_Alpha*_4nt_intrusion_01 | TATCAACACTAAGAACACCCAG |
| hh70_Alpha*_4nt_intrusion_02 | CCTGTAGAAAGTACAGCTAATGCAGAACG |
| hh70_Alpha*_4nt_intrusion_03 | ATTAAATACGTTAATGATAAATAAGGCGT |
| hh70_Alpha*_4nt_intrusion_04 | AATAAACAAATTACT |
| | |
| hh50_Alpha_6nt_intrusion_01 | AGATTTCGTTTATGCGAGTAGTAAATTGGGTGTTTA |
| hh50_Alpha_6nt_intrusion_02 | CGCGCCCTTGAGATGAACTTTACCTCGTTTACCAGA |
| hh50_Alpha_6nt_intrusion_03 | GTTAAAACGAGAAACGGAGATTTAGCGAGAGGCTTTCGACGAT |
| hh50_Alpha_6nt_intrusion_04 | GTACAACACCAGAAAATAAGGCTTGCCCTGTAAGAA |
| hh55_Alpha_6nt_intrusion_01 | GAAAATCTCCAAAACAAAAGGAGCCTCGCTAA |
| hh55_Alpha_6nt_intrusion_02 | ATTGAGTTAATTGTATCGGTTTATCAGAGGCATCAAAT |
| hh55_Alpha_6nt_intrusion_03 | TGGCAATTAAACAGCAACCATCGCGAACCAGACCGGTTTAATTCAACCTA |
| hh55_Alpha_6nt_intrusion_04 | TGACAACTTGATACTTTCGAGGTGAATTTCCATATA |
| hh65_Alpha*_6nt_intrusion_01 | TATCAGAGAGTCAGAGG |
| hh65_Alpha*_6nt_intrusion_02 | CATATGAGAGTCTGCTACAATGTA |
| hh65_Alpha*_6nt_intrusion_03 | GACATTCTTACCAGTAAATCAGTCACCATAAAGG |
| hh65_Alpha*_6nt_intrusion_04 | AAAGAAATAAGCAATTGTAATTTTGTT |
| hh70_Alpha*_6nt_intrusion_01 | TCAACACTAAGAACACCCAG |
| hh70_Alpha*_6nt_intrusion_02 | CCTGTAGAAAGTACAGCTAATGCAGAA |
| hh70_Alpha*_6nt_intrusion_03 | ATTAAATACGTTAATGATAAATAAGGC |
| hh70_Alpha*_6nt_intrusion_04 | TAAACAAATTACTAG |

**Table S17: Au NP handle sequences**: Three connection sites with two handles each were designed by 3’ end elongation of staples, which were otherwise used as part of connection sites. Two Ts were used as linkers between Au NP and its sequence as well as between staples and handles.

| name | sequence 5' -> 3' |
|---|---|
| AuNP_handle_hh50_Alpha | GTACAACACCAGAAAATAAGGCTTGCCCTG TT ATGTAGGTGGTAGAG |
| AuNP_handle_hh55_Alpha | TGACAACTTGATACTTTCGAGGTGAATTTC TT ATGTAGGTGGTAGAG |
| AuNP_handle_hh60_Gamma | ATTGACAAACCACCACCAGAGCC TT ATGTAGGTGGTAGAG |
| AuNP_handle_hh65_Gamma | GGCAAGGACCATCGTAAAGGTAATACCCAAAAG TT ATGTAGGTGGTAGAG |
| AuNP_handle_hh70_Beta | ACAGTAGCTTACCAGTATAAAGC TT ATGTAGGTGGTAGAG |
| AuNP_handle_hh75_Beta | ATTCTCCGAGAGACTCCCTTAGTACCTTTTACA TT ATGTAGGTGGTAGAG |
| | |
| AuNP_sequence | [thiol-C6] - TT CTCTACCACCTACAT |

**Table S18: Number of nt for specific configurations** in absolute numbers and as (*) percentage from a full, passivated structure (9408 nt as sum of core staples, ABC-shell, ABC-passive, and z-passive). Additionally, the absolute number (rounded up) and percentage of nt used per connection site. This divided the number of nt for xy-connections by six and z-connections by eight. The overall number of nt used was 17 875, which is less than the number of nt of staples needed for two whole structures.

| component | # of nt | %* | # nt / con. site | %* / con. site |
|---|---|---|---|---|
| | | | | |
| core | 4 572 | 48.60 | | |
| | | | | |
| ABC - shell | 2 798 | 29.74 | 467 | 4.96 |
| ABC - passivation | 1 750 | 18.60 | 292 | 3.10 |
| ABC - staple intrusions | 1 510 | 16.05 | 252 | 2.68 |
| | | | | |
| DEF - shell | 1 390 | 14.77 | 232 | 2.47 |
| DEF - passivation | 1 118 | 11.88 | 187 | 1.99 |
| DEF - staple intrusions | 1 026 | 10.91 | 171 | 1.82 |
| | | | | |
| cross config. - shell | 569 | 6.05 | | |
| cross config. - passivation | 375 | 3.99 | | |
| cross config. - staple intrusions | 479 | 5.09 | | |
| | | | | |
| z - passive | 423 | 4.50 | | |
| z - handles | 1 916 | 20.37 | 240 | 2.55 |
| z - handles (symmetric) | 1 956 | 20.79 | 245 | 2.60 |
| z - connectors | 84 | 0.89 | 21 | 0.22 |
| | | | | |
| Σ | 19 966 | 212.23 | | |
